# Reference genes for gene expression studies targeting sugarcane infected with *Sugarcane mosaic virus* (SCMV)

**DOI:** 10.1186/s13104-019-4168-5

**Published:** 2019-03-18

**Authors:** Marcel Fernando da Silva, Marcos Cesar Gonçalves, Michael dos Santos Brito, Paula Macedo Nóbile, Larissa Mara de Andrade, Cibele Nataliane Medeiros, Silvana Creste, Luciana Rossini Pinto

**Affiliations:** 1 Instituto Agronômico, Centro de Cana, CP 206, Ribeirão Preto, SP CEP 14001-970 Brazil; 2 Crop Protection Research Centre, Instituto Biológico, São Paulo, SP Brazil; 3 Instituto de Ciência e Tecnologia da Universidade Federal de São Paulo, São José dos Campos, SP Brazil

**Keywords:** *Saccharum* spp., Sugarcane mosaic disease, Normalization, BestKeeper, NormFinder, GeNorm, RefFinder

## Abstract

**Objective:**

The selection of reference genes in sugarcane under *Sugarcane mosaic virus* (SCMV) infection has not been reported and is indispensable to get reliable reverse transcription quantitative PCR (RT-qPCR) results for validation of transcriptome analysis. In this regard, seven potential reference genes were tested by RT-qPCR and ranked according to their stability using BestKeeper, NormFinder and GeNorm algorithms, and RefFinder WEB-based software in an experiment performed with samples from two sugarcane cultivars contrasting for SCMV resistance, when mechanically inoculated with a severe SCMV strain and using mock inoculated plant controls.

**Results:**

The genes Uridylate kinase (UK) and Ubiquitin-conjugating enzyme 18 (UBC18) were the most stable according to GeNorm algorithm and the Pearson correlation coefficients with the BestKeeper index. On the other hand, ribosomal protein L35-4 (RPL1), Actin (ACT) and Ubiquitin1 (UBQ1) were the least stable genes for all algorithms tested.

**Electronic supplementary material:**

The online version of this article (10.1186/s13104-019-4168-5) contains supplementary material, which is available to authorized users.

## Introduction

Sugarcane mosaic disease (SMD) is widely distributed among sugarcane-growing countries and may be caused by different virus species of the genera *Potyvirus* and *Poacevirus*, family *Potyviridae* [1]. In Brazil, *Sugarcane mosaic virus* (SCMV), *Potyvirus*, is one the main viruses affecting sugarcane and the only causal agent of SMD, to date [2, 3]. The disease is controlled by the use of resistant cultivars making the comprehension of molecular bases of resistance to these viruses of great concern for sugarcane breeding programs worldwide [1, 2]. Transcriptome analysis has been applied in sugarcane to identify differentially expressed genes associated with biological traits [4–6] yet, few studies have investigated changes in the sugarcane transcriptome under infection by mosaic-causing viruses [7, 8]. The validation of transcriptome results via reverse transcription quantitative PCR (RT-qPCR) requires a normalization step for reducing its uncertainties [9, 10], commonly attained by the use of endogenous reference genes [11, 12]. The choice of appropriate reference genes is an essential step, since improper selection of references genes may result in unreliable RT-qPCR results [13]. Several algorithms are available for identification of reliable candidate reference genes [10], which is a necessary procedure whenever different experimental conditions and genotypes are involved [13, 14], however such studies for SMD are yet to be reported. In this regard, the present study aimed to evaluate seven candidate reference genes based on previous reports in sugarcane under drought stress [15] and in closely related monocot species under viral infection [16].

## Main text

### Methods

#### Plant material and experimental design

The biological samples used in this study proceed from a previous experiment performed by Medeiros et al. [7]. It was used a completely randomized factorial design with three factors, under greenhouse conditions: (a) two sugarcane cultivars, IACSP95-5000, resistant to SCMV, and IAC91-1099, susceptible to SCMV, both from the “Sugarcane Breeding Program, Instituto Agronômico de Campinas, IAC”, Brazil (b) two treatments (SCMV inoculated, s.i; and mock inoculated, m.i), and (c) a time course experiment with three sampling time points of the + 1 leaf, 24, 48 and 72 h post inoculation (hpi). Briefly, 36 sugarcane plantlets of each cultivar were obtained by meristem tip culture and indexed as virus-free by reverse transcription PCR (RT-PCR) using specific primers for the SCMV capsid protein [17]. At 1-month-old, 18 plantlets of each cultivar were submitted to the s.i treatment using a severe strain of SCMV (SCMV Rib-1) [18] and the remaining 18 were submitted to the m.i treatment, according to Bain method [19]. Therefore, six biological replicates were used for each combination of experimental factors. Among the s.i and m.i samples indexed by RT-PCR respectively as virus infected and virus free, three biological replicates from 24 and 72 hpi for each genotype × treatment were selected for the stability assessment of the seven candidate reference genes. This choice was based on the higher number of differentially transcribed fragments (DTFs) observed in cDNA-AFLP analysis at these sampling time points [7].

#### Total RNA isolation and cDNA synthesis

Total RNA was extracted from the sugarcane + 1 leaf of each biological replicate with Trizol reagent (Invitrogen, Carlsbad, USA) following the manufacturer’s instructions, and stored at − 80 °C. RNA concentration was estimated in a spectrophotometer NanoDrop2000 (Thermo Fischer Scientific, Wilmington DE, USA), and RNA integrity was checked in 1.5% agarose’s gel. Firstly, 1 μg of total RNA was treated with DNase I, following manufacturer’s instructions (Promega, Fitchburg WI, USA), to remove genomic DNA. Reverse transcription of DNase treated RNA was then performed using the GoScript Reverse Transcription System (Promega) kit, according to manufacturer’s instructions.

#### Candidate reference genes and primer design

The sequence of reference genes reported in sorghum (*Sorghum bicolor*) infected with *Brome mosaic virus* (BMV, *Bromovirus*) and in maize (*Zea mays*) with *Barley stripe mosaic virus* (BSMV, *Hordeivirus*), *Rice black*-*streaked dwarf virus* (RBSDV, *Fijivirus*) and SCMV, namely Uridylate kinase (UK), SAND protein family (SAND), and Ubiquitin-conjugating enzyme 18 gene (UBC18) [16], were obtained in the DFCI gene index database [20]. These sequences were used as queries to search within the SUCEST-FUN (Sugarcane Expressed Sequence Tag Functional Analysis) database [21] by using BlastN tool and adopting an E-value of 1e−5 as inferior threshold. The primer design was performed using PrimerQuest tool [22] and analyzed using Netprimer software [23]. The other four candidate reference genes were selected based on sugarcane gene expression studies under drought stress described by Andrade et al. [15]: glyceraldehyde-3-phosphate dehydrogenase (GAPDH), 60S ribosomal protein L35-4 (RPL1), Actin (ACT) and Ubiquitin1 (UBQ1).

#### Quantitative PCR conditions

The RT-qPCR reactions were performed on an Applied Biosystems StepOnePlus System (Foster City CA, USA). The reaction mixture consisted in 5 μL of SYBR Green Power Master Mix (Applied Biosystems), 3 μL of 1:10 diluted cDNA and 0.2 μM of each forward and reverse primers in a total volume of 10 μL. The reaction thermal profile consisted in an initial denaturation step at 95 °C for 20 s, followed by 40 cycles of denaturation 95 °C for 3 s; 60 °C for 30 s. At the end of RT-qPCR reaction, dissociation curve profiles (melting curves) were carried out for amplicon specificity analysis.

#### Stability evaluation and selection of reference genes

The cDNA from the three aforementioned biological replicates were pooled together, resulting in eight cDNA samples, which were used in three technical replicates for the gene stability assessment (n = 24 for each gene): IAC91-1099 24 hpi (m.i), IAC91-1099 24 hpi (s.i), IAC91-1099 72 hpi (m.i), IAC91-1099 72 hpi (s.i), IACSP95-5000 24 hpi (m.i), IACSP95-5000 24 hpi (s.i), IACSP95-5000 72 hpi (m.i) and IACSP95-5000 72 hpi (s.i). PCR product threshold cycle (Ct) and PCR reaction efficiency data provided by LinReg PCR analysis [24] were used to seek for the best reference gene or best gene pair with NormFinder [25], BestKeeper [26] free Excel based software packages and GeNorm from NormqPCR R package [27], whilst RefFinder WEB-based software [28] identified the best reference gene based on Ct data.

### Results

#### Homology of sugarcane ESTs to maize and sorghum candidate reference genes and efficiency of RT-qPCR

The candidate reference genes UBC18, SAND and UK showed identity ranging from 93 to 96% and a highly significant alignment (E-value = 0) with the sugarcane ESTs (Additional file 1: Table S1). The ranking of the mean Ct-value was GAPDH > UK > SAND > UBC18 > RPL1 > ACT > UBQ1, whilst the ranking of coefficient of variation (CV%) values was SAND > UK > GAPDH > UBC18 > RPL1 > UBQ1 > ACT (Additional file 2: Figure S1; Additional file 3: Tables S2 and S3).

The LinReg PCR program, which detects the exponential phase of amplification by fluorescence data plotting in a logarithmical scale [24], showed amplification efficiency ranging from 90.2 to 98.2%. The matching degree of the plotted data to the standard curve in the PCR reaction, revealed by correlation coefficients (R^2^), ranged from 0.998 to 0.999 (Additional file 3: Tables S2 and S4). Each pair of primers showed a unique peak of fluorescence in the melting curves (Additional file 4: Figure S2), indicating single fragment amplification during RT-qPCR. The newly designed pairs of primers for genes SAND, UK and UBC18 showed the predicted amplicon size in 1% agarose gel (Additional file 5: Figure S3).

#### Gene expression stability

According to NormFinder the seven candidate reference genes showed the following ranking, from the most to the least stable gene: SAND > UK > GAPDH > UBC18 > RPL1 > UBQ1 > ACT. Moreover, with stability value of 0.181, SAND/GAPDH is the best combination of two genes, which represents the minimal combined intra- and intergroup variation in gene expression. The BestKeeper analysis involved two approaches, with the first, the BestKeeper standard deviation (SD) statistics, presenting the same stability ranking from above. The second approach was performed stepwise, with successive exclusion of candidate reference genes based on the SD threshold of 1.0 established by Pfaffl et al. [26], and on low Pearson correlation coefficient (r) values with significance cutoff at 5% level (P < 0.05). The selected genes were further ranked based on the Pearson correlation coefficients with the BestKeeper index, leading to the statement of UBC18 and UK as the most stable genes, all significant at 1% of probability. Results generated by GeNorm algorithm analysis showed the following gene ranking: UBC18/UK > SAND > GAPDH > RPL1 > UBQ1 > ACT (Table 1).

**Table 1:** Analyses of candidate reference genes by BestKeeper, GeNorm and NormFinder algorithms

BestKeeper^a^							
Gene ranking^b^	SAND	UK	GAPDH	UBC18	RPL1	UBQ1	ACT
N	24	24	24	24	24	24	24
SD [± CP]	0.21	0.25	0.28	0.38	0.59	1.02	1.04
CV [% CP]	0.72	0.97	1.18	1.28	1.98	2.99	3.24
BestKeeper^c^							
Gene ranking^b^	SAND	UK	GAPDH	UBC18	RPL1	UBQ1	ACT
UK	0.456	–	–	–	–	–	–
	0.025	–	–	–	–	–	–
GAPDH	− 0.563	− 0.611	–	–	–	–	–
	0.004	0.002	–	–	–	–	–
UBC18	0.473	0.690	− 0.367	–	–	–	–
	0.019	0.001	0.078	–	–	–	–
RPL1	0.259	0.163	− 0.393	0.320	–	–	–
	0.221	0.444	0.058	0.128	–	–	–
UBQ1	0.151	− 0.126	0.036	− 0.232	− 0.345	–	–
	0.479	0.555	0.867	0.275	0.099	–	–
ACT	− 0.330	− 0.419	0.781	− 0.353	− 0.773	0.419	–
	0.115	0.041	0.001	0.091	0.001	0.041	–
Coeff. of corr. [r]^d^	0.211	− 0.004	0.365	0.130	− 0.299	0.760	0.636
P-value	0.323	0.984	0.079	0.542	0.156	0.001	0.001
BestKeeper^e^	SAND	UK		UBC18			
Coeff. of corr. [r]	0.705	0.867		0.910			
P-value	0.001	0.001		0.001			
GeNorm							
Gene ranking^b^	UBC18	UK	SAND	GAPDH	RPL1	UBQ1	ACT
Stablity (M)	0.224	0.224	0.276	0.578	0.691	1.296	1.343
NormFinder							
Gene ranking^b^	SAND	UK	GAPDH	UBC18	RPL1	UBQ1	ACT
Stability	0.227	0.274	0.279	0.381	0.539	0.677	0.709
Best gene combination of two genes	SAND		GAPDH				
Stability	0.181		0.181				

^a^BestKeeper SD statistics of all seven candidate reference genes

^b^Ranking from the most to the least stable gene (left to right)

^c^Repeated pair-wise correlation analysis among candidate reference genes

^d^Coefficient of correlation with the BestKeeper index

^e^Coefficient of correlation with the BestKeeper index after the exclusion of candidate reference genes with standard dev (SD) above 1.0 and with low Pearson correlation (r) values

**Fig. 1:**
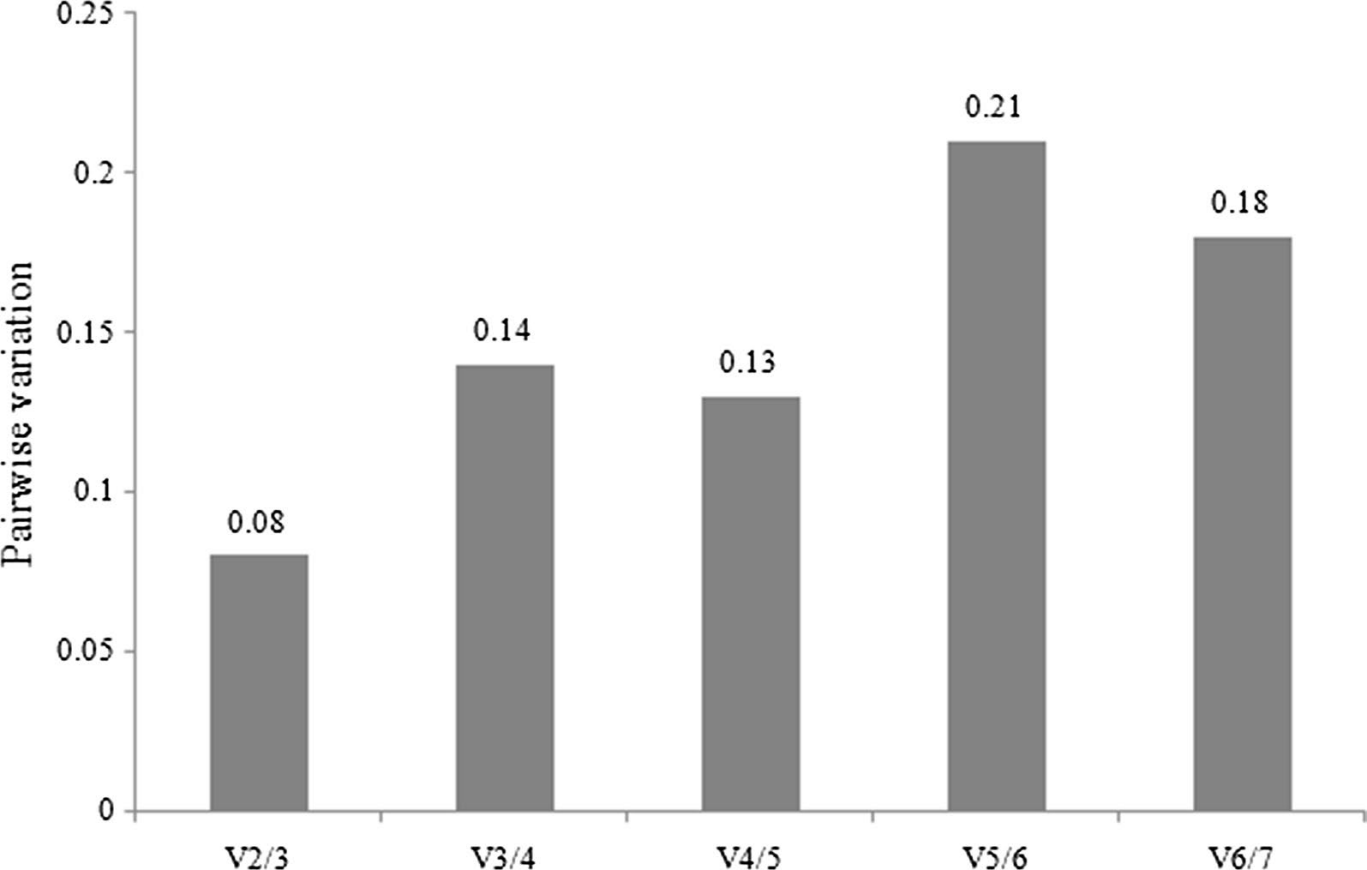
Pairwise variation analysis between the normalization factors NF_n_ and NF_n+1_ of candidate reference genes calculated by GeNorm in order to determine the optimal number of control genes for normalization

The pairwise variation V_n_/V_n+1_ of two sequential normalization factors NF_n_ and NF_n+1_ calculated by GeNorm showed a V_2/3_ value of 0.08, indicating that the inclusion of a third reference gene has no significant effect for normalization, considering a threshold value below 0.15 [29] (Fig. 1).

Among the algorithms tested in RefFinder, SAND and GAPDH were the most stable genes according to NormFinder. UBC18 and UK were the best pair of genes according to GeNorm while SAND and UK were indicated as the most stable by DeltaCt and BestKeeper algoritms. The comprehensive ranking presented the same ranking of the DeltaCt algorithm: SAND > UK > UBC18 > GAPDH > RPL1 > UBQ1 > ACT (Table 2).

**Table 2:** Analyses of candidate reference genes according to RefFinder tool

Comprehensive ranking^a^		DeltaCt^a^		BestKeeper^a^		NormFinder^a^		GeNorm^a^	
Gene	Stability	Gene	Stability	Gene	Stability	Gene	Stability	Gene	Stability
SAND	1.32	SAND	0.74	SAND	0.171	SAND	0.100	UBC18	0.243
UK	1.86	UK	0.77	UK	0.224	GAPDH	0.232	UK	0.243
UBC18	2.63	UBC18	0.80	GAPDH	0.274	UK	0.336	SAND	0.275
GAPDH	3.13	GAPDH	0.84	UBC18	0.316	UBC18	0.456	GAPDH	0.439
RPL1	5.00	RPL1	1.08	RPL1	0.585	RPL1	0.913	RPL1	0.559
UBQ1	6.00	UBQ1	1.38	UBQ1	1.014	UBQ1	1.198	UBQ1	0.835
ACT	7.00	ACT	1.43	ACT	1.030	ACT	1.298	ACT	1.006

^a^Ranking from the most to the least stable gene (top to bottom)

### Discussion

The most commonly employed algorithms for reference gene expression stability analysis are based on different mathematical approaches [30], and often result in dissimilar outcomes. In the present study, this was observed by different statements of the most stable genes, i.e. SAND and UK, SAND/GAPDH, UBC18/UK. The comprehensive rank provided by RefFinder, allows an overall assessment of gene stability based on these different mathematical approaches [31], but should be restricted as a complementary tool for reference gene stability assessment taking into account the strengths and weaknesses of each algorithm [32]. Among the tested algorithms, BestKeeper is addressed as a “common sense” between the need of reference genes with low SD values and good correlation among them, assuming that the reference genes are not co-regulated [30]. Considering this, the Bestkeeper algorithm indicates that UK and UBC18 genes have an acceptable low SD and high correlation between them, being in agreement with GeNorm output. On the other hand, the best combination of two genes SAND/GAPDH calculated by NormFinder had low Pearson correlation coefficient (c) values according to BestKeeper, which favors the choice of UK and UBC18 genes.

The statement of genes UK and UBC18 as the most stable in SCMV-infected sugarcane by GeNorm and BestKeeper algorithms resemble the reports of Zhang et al. [16], e.g. GeNorm and BestKeeper outputs for BMV and BSMV-infected barley (*Hordeum vulgare*), and NormFinder output for BMV-infected sorghum. The UBC18 gene stability is noteworthy since ubiquitin expression has been used for normalization in maize infected by different potyviruses [33]. The SAND and GAPDH genes also were reliably stable when subjected to all algorithms used in the present study. Similarly, SAND was reported as the most stable in wheat (*Triticum aestivum*) infected by BSMV and RBSDV according to NormFinder, and in BMV-infected Sorghum according to GeNorm, while GAPDH was ranked by NormFinder and GeNorm as the most stable in BSMV-infected Brachypodium (*Brachypodium distachyon*) [16]. Our results rank RPL1, UBQ1 and ACT genes amongst the least stable genes by all algorithms. The poor transcript stability of ACT is in agreement with previous studies [16, 34, 35], while the report of 60 s ribosomal protein in replication complexes during potyvirus infection [36] seem to corroborate our observations for RPL1. The sugarcane UBQ1 gene contrasted with UBC18, suggesting that potyviruses may interfere with pathways involving certain ubiquitin genes as reported by Cheng; Wang [37].

The results indicate that UBC18 and UK are the most stable sugarcane reference genes in leaves when the target is gene expression studies in search for resistance to SCMV by RT-qPCR approaches, and should also be considered as candidate reference genes for accurate normalization for other expression studies involving SMD.

## Limitations

It is necessary to reassess expression stability of candidate reference genes when different experimental conditions and genotypes are involved in SMD studies. In addition, an important step for the selection of reference genes is the validation by RT-qPCR analysis of a well-studied sugarcane gene responsive to SCMV infection, which information is lacking in literature. Therefore, studies with good candidate genes, e.g. recent reports in maize [38, 39] could provide useful data.

## Additional files

**Additional file 1: Table S1.** Sugarcane ESTs homologue to maize and sorghum candidate reference genes.

**Additional file 2: Figure S1.** Evaluation of Ct values of seven candidate reference genes across all leaf samples. The box indicates 25-75% while the line across the box represents the median and whiskers represent the range from minimum to maximum.

**Additional file 3: Table S2.** Primer pairs sequences, amplicon size (A) in basepairs (bp), melting temperature (Tm), coefficient of variation (CV), PCR reaction efficiency (E) and coefficient of determination (R^2^) of genes selected for stability assessment under SCMV infection. **Table S3.** Individual Ct values of each gene in sugarcane leaf samples. **Table S4.** RT-qPCR Cycles, Fluorescence (Rn) and change in Fluorescence (ΔRn) of each candidate reference gene in sugarcane leaf samples used as input in LinRegPCR for reaction efficiency assessment.

**Additional file 4: Figure S2.** Dissociation curve of seven candidate reference genes, with pictures taken using the qPCR instrument’s software. The dissociation curves for no template controls (NTCs) are indicated by an arrow.

**Additional file 5: Figure S3.** qRT-PCR amplicon size verification in agarose gel 1% of three newly designed primer pairs in cDNA bulks and genomic DNA from IACSP95-5000 and IAC91-1099 sugarcane cultivars.

## Abbreviations

SMD: sugarcane mosaic disease
hpi: hours post inoculation
m.i: mock-inoculated
s.i: SCMV-inoculated
