# Supplementary material for: Reference genes for gene expression studies targeting sugarcane infected with Sugarcane mosaic virus (SCMV)
Source: BMC Res Notes. 2019 Mar 18;12:149. doi: 10.1186/s13104-019-4168-5 (PMC6423880; doi:10.1186/s13104-019-4168-5)
Supplement: Supplementary file 1 — Additional file 1: Table S1. Sugarcane ESTs homologue to maize and sorghum candidate reference genes. [file 13104_2019_4168_MOESM1_ESM.docx]

Table S1. Sugarcane ESTs homologue to maize and sorghum candidate reference genes.

| Gene symbol | Accession DFCI^a^ | Homology in SUCEST^b^ | Accession  Number^c^ | E-value | Identity (%) | Gap (%) |
| --- | --- | --- | --- | --- | --- | --- |
| UBC18 | TC479874 | SCEZSD1083D06.g | CA285930.1 | 0 | 95 | 0 |
| SAND | TC131743 | SCSGFL4194B02.b | CA257083.1 | 0 | 93 | 0 |
| UK | TC123979 | SCQSST1039D09.g | CA177998 | 0 | 96 | 0 |

a: http://compbio.dfci.harvard.edu/tgi/

b: http://sucest-fun.org

c: NCBI database
