# Supplementary figures and images for: Reference genes for gene expression studies targeting sugarcane infected with Sugarcane mosaic virus (SCMV)
Source: BMC Res Notes. 2019 Mar 18;12:149. doi: 10.1186/s13104-019-4168-5 (PMC6423880; doi:10.1186/s13104-019-4168-5)

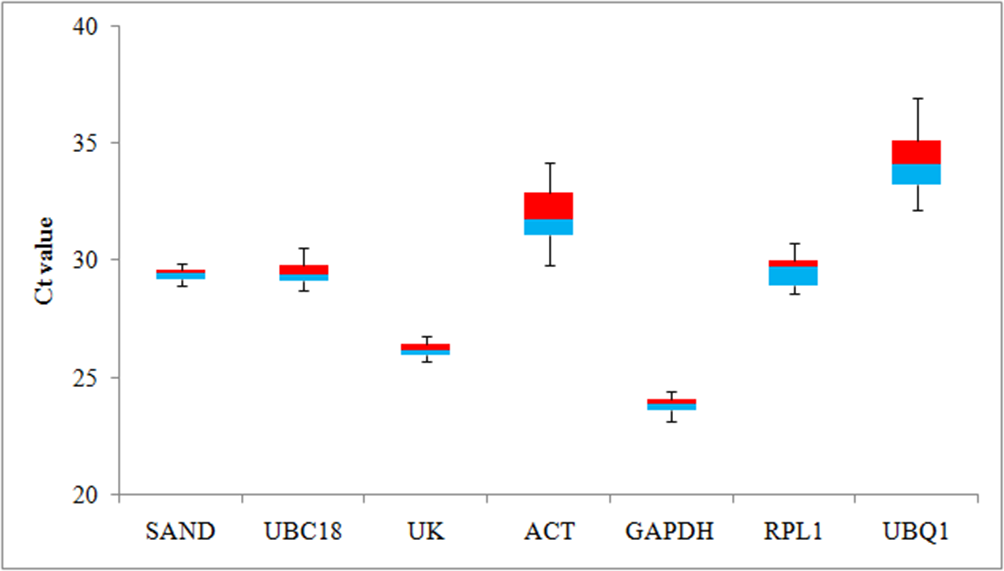

Supplement: Supplementary file 2 — Additional file 2: Figure S1. Evaluation of Ct values of seven candidate reference genes across all leaf samples. The box indicates 25-75% while the line across the box represents the median and whiskers represent the range from minimum to maximum. [file 13104_2019_4168_MOESM2_ESM.png]

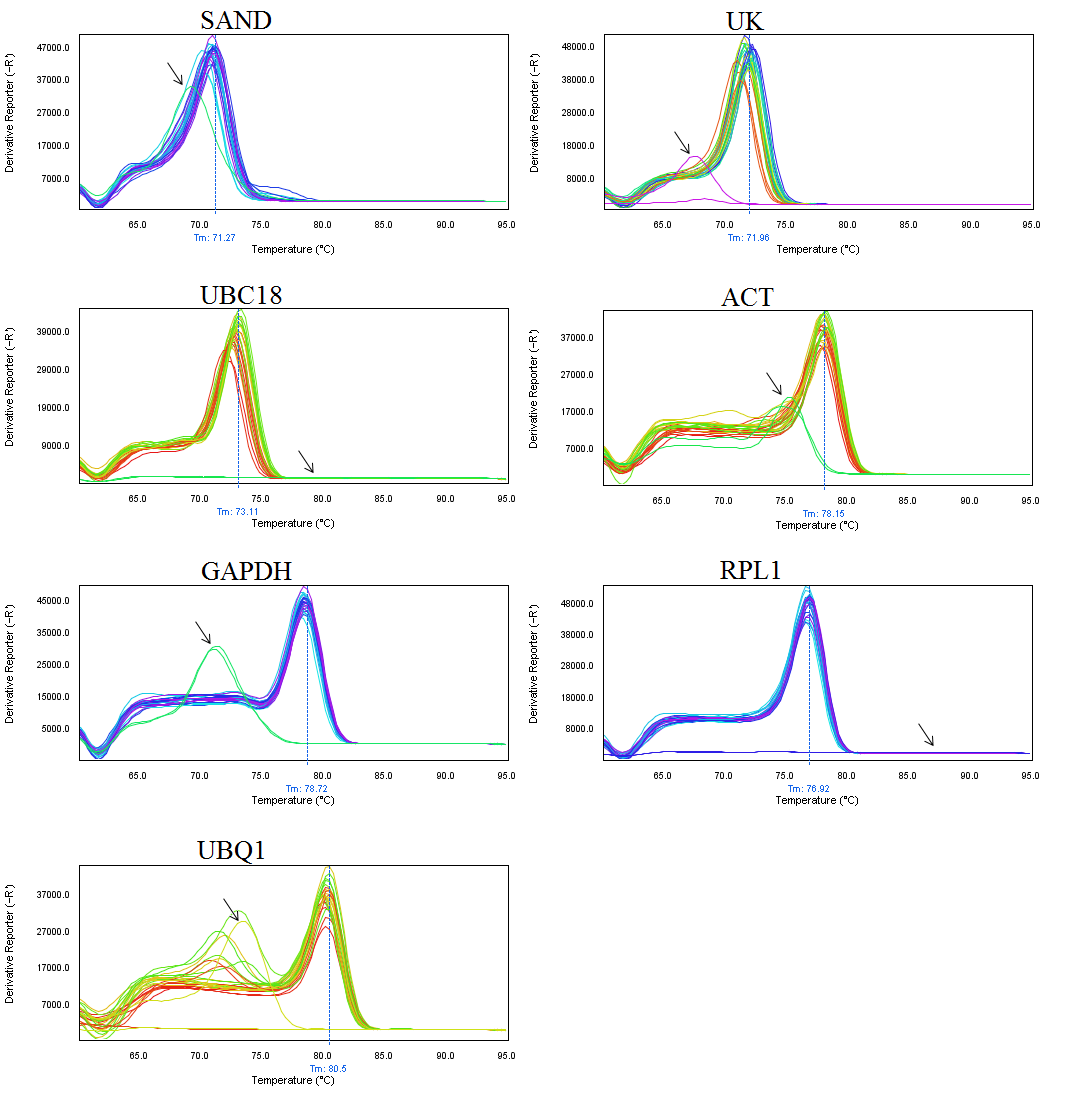

Supplement: Supplementary file 4 — Additional file 4: Figure S2. Dissociation curve of seven candidate reference genes, with pictures taken using the qPCR instrument’s software. The dissociation curves for no template controls (NTCs) are indicated by an arrow. [file 13104_2019_4168_MOESM4_ESM.png]
