# Supplementary material for: Reference genes for gene expression studies targeting sugarcane infected with Sugarcane mosaic virus (SCMV)
Source: BMC Res Notes. 2019 Mar 18;12:149. doi: 10.1186/s13104-019-4168-5 (PMC6423880; doi:10.1186/s13104-019-4168-5)
Supplement: Supplementary file 3 — Additional file 3: Table S2. Primer pairs sequences, amplicon size (A) in basepairs (bp), melting temperature (Tm), coefficient of variation (CV), PCR reaction efficiency (E) and coefficient of determination (R2) of genes selected for stability assessment under SCMV infection. Table S3. Individual Ct values of each gene in sugarcane leaf samples. Table S4. RT-qPCR Cycles, Fluorescence (Rn) and change in Fluorescence (ΔRn) of each candidate reference gene in sugarcane leaf samples used as input in LinRegPCR for reaction efficiency assessment. [file 13104_2019_4168_MOESM3_ESM.docx]

Table S2. Primer pairs sequences, amplicon size (A) in basepairs (bp), melting temperature (Tm), coefficient of variation (CV), PCR reaction efficiency (E), and coefficient of determination (R^2^) of genes selected for stability assessment under SCMV infection.

| Primers | Sequence F (5’-3’) | Sequence R (5’-3’) | A (bp) | Tm (ºC) | Mean Ct | SD | CV (%) | E (%) | R^2^ |
| --- | --- | --- | --- | --- | --- | --- | --- | --- | --- |
| SAND | CTGTGTGTAAGTTGATATGTCTATGTTG | CAACAACGATGGTACTGCCT | 94 | 70.9 | 29.4 | 0.26 | 0.88 | 97.0 | 0.999 |
| UBC18 | GCCTGTCAGCCTTCCTTAC | GGTAAGCTTCGCAAATCCAATAG | 100 | 72.8 | 29.4 | 0.46 | 1.55 | 98.2 | 0.999 |
| UK | GCAATCTAAGGGACATAATAAAGGTG | AATCGAATTGCCTACTGATATGTTG | 140 | 71.7 | 26.2 | 0.31 | 1.17 | 91.7 | 0.999 |
| ACT* | CTCAACCCCAAGGCTAACAG | GGCATGAGGAAGGGCATAA | 195 | 78.1 | 31.9 | 1.24 | 3.89 | 96.4 | 0.999 |
| GAPDH* | TTGGTTTCCACTGACTTCGTT | CTGTAGCCCCACTCGTTGT | 122 | 78.5 | 23.8 | 0.36 | 1.53 | 94.9 | 0.999 |
| RPL1* | CTGAAGACGGAGAGGGAAAA | GGCGAAGAGAAACTAACAC | 264 | 76.8 | 29.6 | 0.68 | 2.31 | 90.2 | 0.999 |
| UBQ1* | AGCCTCAGACCAGATTCCAA | AATCGCTGTCGAACTACTTGC | 110 | 79.5 | 34.2 | 1.26 | 3.67 | 92.1 | 0.998 |

*Primer pairs sequences reported by Andrade et al. [15].

Table S3. Individual Ct values of each gene in sugarcane leaf samples.

|  | Technical replicate | SAND | UBC18 | UK | ACT | GAPDH | RPL1 | UBQ1 |
| --- | --- | --- | --- | --- | --- | --- | --- | --- |
| IAC91-1099 24 hpi (m.i) | 1 | 30.56796 | 29.54655 | 26.61342 | 31.08426 | 24.0271 | 33.27314 | 30.74711 |
| IAC91-1099 24 hpi (m.i) | 2 | 29.64479 | 29.20693 | 26.15763 | 31.00998 | 23.91812 | 32.25776 | 30.65352 |
| IAC91-1099 24 hpi (m.i) | 3 | 29.77252 | 29.35209 | 26.16544 | 30.93571 | 23.9146 | 32.76926 | 30.43229 |
| IAC91-1099 24 hpi (s.i) | 1 | 29.47044 | 29.69482 | 26.56159 | 29.81182 | 23.21395 | 33.10108 | 29.73594 |
| IAC91-1099 24 hpi (s.i) | 2 | 29.96142 | 29.4563 | 26.36762 | 29.90277 | 23.16707 | 33.23085 | 29.78076 |
| IAC91-1099 24 hpi (s.i) | 3 | 29.92889 | 29.57556 | 26.75556 | 30.27352 | 23.10898 | 32.9713 | 29.75835 |
| IAC91-1099 72 hpi (m.i) | 1 | 29.66803 | 29.85129 | 26.42537 | 31.62352 | 23.57519 | 36.92767 | 30.32771 |
| IAC91-1099 72 hpi (m.i) | 2 | 29.05064 | 29.24616 | 26.02413 | 31.19548 | 23.51858 | 35.48368 | 30.24707 |
| IAC91-1099 72 hpi (m.i) | 3 | 28.88357 | 29.72104 | 26.22632 | 31.26264 | 23.44932 | 35.9382 | 29.98198 |
| IAC91-1099 72 hpi (s.i) | 1 | 29.9642 | 29.71221 | 26.63687 | 32.97979 | 23.60205 | 35.99268 | 28.71916 |
| IAC91-1099 72 hpi (s.i) | 2 | 29.86286 | 29.45235 | 26.61909 | 32.77672 | 23.70368 | 34.45903 | 28.59753 |
| IAC91-1099 72 hpi (s.i) | 3 | 29.53459 | 29.41702 | 26.65466 | 32.57365 | 23.85609 | 34.9776 | 29.00128 |
| IACSP95-5000 24 hpi (m.i) | 1 | 29.19224 | 29.46247 | 26.19417 | 31.24293 | 23.94404 | 33.39813 | 29.81638 |
| IACSP95-5000 24 hpi (m.i) | 2 | 29.24021 | 29.3891 | 26.18582 | 31.0277 | 23.85316 | 32.1547 | 29.95093 |
| IACSP95-5000 24 hpi (m.i) | 3 | 29.14426 | 28.96103 | 26.33602 | 31.41401 | 23.85373 | 33.28429 | 30.12449 |
| IACSP95-5000 24 hpi (s.i) | 1 | 29.91535 | 29.62702 | 26.05991 | 32.05695 | 23.80566 | 34.43314 | 29.89141 |
| IACSP95-5000 24 hpi (s.i) | 2 | 29.15786 | 29.53018 | 25.93169 | 31.86118 | 23.76799 | 34.39444 | 29.5534 |
| IACSP95-5000 24 hpi (s.i) | 3 | 29.04382 | 29.67727 | 25.76982 | 32.0737 | 23.88823 | 34.10696 | 29.80936 |
| IACSP95-5000 72 hpi (m.i) | 1 | 29.35568 | 29.17928 | 25.84801 | 33.00624 | 24.20568 | 35.64184 | 28.95149 |
| IACSP95-5000 72 hpi (m.i) | 2 | 28.74776 | 28.94528 | 25.92402 | 33.4669 | 24.33144 | 34.96577 | 28.58057 |
| IACSP95-5000 72 hpi (m.i) | 3 | 28.79636 | 28.92381 | 25.66535 | 32.85384 | 24.24599 | 35.30381 | 28.95483 |
| IACSP95-5000 72 hpi (s.i) | 1 | 29.4139 | 29.54389 | 25.98713 | 34.16151 | 24.39715 | 33.76658 | 28.77237 |
| IACSP95-5000 72 hpi (s.i) | 2 | 29.15899 | 29.15408 | 26.07737 | 33.83062 | 24.23381 | 33.94919 | 28.83875 |
| IACSP95-5000 72 hpi (s.i) | 3 | 28.95596 | 29.19102 | 25.98798 | 33.86447 | 24.29822 | 34.13179 | 28.84121 |

Table S4. RT-qPCR Cycles, Fluorescence (Rn) and change in Fluorescence (ΔRn) of each candidate reference gene in sugarcane leaf samples used as input in LinRegPCR for reaction efficiency assessment.

| cDNA bulk | Techinal replicate | RT-qPCR Cycle | Target Name | Rn | ΔRn |
| --- | --- | --- | --- | --- | --- |
| IAC91-1099 24 hpi (m.i) | 1 | 1 | SAND | -0.08006 | 0.004986 |
| IAC91-1099 24 hpi (m.i) | 1 | 2 | SAND | -0.08646 | 0.000623 |
| IAC91-1099 24 hpi (m.i) | 1 | 3 | SAND | -0.08997 | -0.00085 |
| IAC91-1099 24 hpi (m.i) | 1 | 4 | SAND | -0.09384 | -0.00267 |
| IAC91-1099 24 hpi (m.i) | 1 | 5 | SAND | -0.095 | -0.00179 |
| IAC91-1099 24 hpi (m.i) | 1 | 6 | SAND | -0.0949 | 0.000352 |
| IAC91-1099 24 hpi (m.i) | 1 | 7 | SAND | -0.09667 | 0.000621 |
| IAC91-1099 24 hpi (m.i) | 1 | 8 | SAND | -0.09795 | 0.001377 |
| IAC91-1099 24 hpi (m.i) | 1 | 9 | SAND | -0.10112 | 0.000256 |
| IAC91-1099 24 hpi (m.i) | 1 | 10 | SAND | -0.10332 | 9.15E-05 |
| IAC91-1099 24 hpi (m.i) | 1 | 11 | SAND | -0.104 | 0.00145 |
| IAC91-1099 24 hpi (m.i) | 1 | 12 | SAND | -0.10708 | 0.000411 |
| IAC91-1099 24 hpi (m.i) | 1 | 13 | SAND | -0.10801 | 0.001519 |
| IAC91-1099 24 hpi (m.i) | 1 | 14 | SAND | -0.10781 | 0.003768 |
| IAC91-1099 24 hpi (m.i) | 1 | 15 | SAND | -0.11234 | 0.00127 |
| IAC91-1099 24 hpi (m.i) | 1 | 16 | SAND | -0.1143 | 0.001354 |
| IAC91-1099 24 hpi (m.i) | 1 | 17 | SAND | -0.11774 | -4.5E-05 |
| IAC91-1099 24 hpi (m.i) | 1 | 18 | SAND | -0.1202 | -0.00047 |
| IAC91-1099 24 hpi (m.i) | 1 | 19 | SAND | -0.12478 | -0.003 |
| IAC91-1099 24 hpi (m.i) | 1 | 20 | SAND | -0.12742 | -0.0036 |
| IAC91-1099 24 hpi (m.i) | 1 | 21 | SAND | -0.12909 | -0.00324 |
| IAC91-1099 24 hpi (m.i) | 1 | 22 | SAND | -0.12812 | -0.00022 |
| IAC91-1099 24 hpi (m.i) | 1 | 23 | SAND | -0.12653 | 0.003413 |
| IAC91-1099 24 hpi (m.i) | 1 | 24 | SAND | -0.11921 | 0.01277 |
| IAC91-1099 24 hpi (m.i) | 1 | 25 | SAND | -0.10392 | 0.030097 |
| IAC91-1099 24 hpi (m.i) | 1 | 26 | SAND | -0.07038 | 0.065679 |
| IAC91-1099 24 hpi (m.i) | 1 | 27 | SAND | -0.00474 | 0.133364 |
| IAC91-1099 24 hpi (m.i) | 1 | 28 | SAND | 0.13348 | 0.273622 |
| IAC91-1099 24 hpi (m.i) | 1 | 29 | SAND | 0.379443 | 0.521625 |
| IAC91-1099 24 hpi (m.i) | 1 | 30 | SAND | 0.824368 | 0.968591 |
| IAC91-1099 24 hpi (m.i) | 1 | 31 | SAND | 1.494267 | 1.64053 |
| IAC91-1099 24 hpi (m.i) | 1 | 32 | SAND | 2.22298 | 2.371284 |
| IAC91-1099 24 hpi (m.i) | 1 | 33 | SAND | 2.937368 | 3.087713 |
| IAC91-1099 24 hpi (m.i) | 1 | 34 | SAND | 3.61695 | 3.769335 |
| IAC91-1099 24 hpi (m.i) | 1 | 35 | SAND | 4.219319 | 4.373745 |
| IAC91-1099 24 hpi (m.i) | 1 | 36 | SAND | 4.700764 | 4.85723 |
| IAC91-1099 24 hpi (m.i) | 1 | 37 | SAND | 5.084086 | 5.242593 |
| IAC91-1099 24 hpi (m.i) | 1 | 38 | SAND | 5.425779 | 5.586326 |
| IAC91-1099 24 hpi (m.i) | 1 | 39 | SAND | 5.640101 | 5.802689 |
| IAC91-1099 24 hpi (m.i) | 1 | 40 | SAND | 5.824191 | 5.988819 |
| IAC91-1099 24 hpi (m.i) | 2 | 1 | SAND | 0.007076 | 0.014959 |
| IAC91-1099 24 hpi (m.i) | 2 | 2 | SAND | -0.00502 | 0.004423 |
| IAC91-1099 24 hpi (m.i) | 2 | 3 | SAND | -0.01019 | 0.000821 |
| IAC91-1099 24 hpi (m.i) | 2 | 4 | SAND | -0.0127 | -0.00012 |
| IAC91-1099 24 hpi (m.i) | 2 | 5 | SAND | -0.01487 | -0.00073 |
| IAC91-1099 24 hpi (m.i) | 2 | 6 | SAND | -0.01635 | -0.00065 |
| IAC91-1099 24 hpi (m.i) | 2 | 7 | SAND | -0.01684 | 0.000424 |
| IAC91-1099 24 hpi (m.i) | 2 | 8 | SAND | -0.01817 | 0.000657 |
| IAC91-1099 24 hpi (m.i) | 2 | 9 | SAND | -0.01932 | 0.00107 |
| IAC91-1099 24 hpi (m.i) | 2 | 10 | SAND | -0.02113 | 0.00082 |
| IAC91-1099 24 hpi (m.i) | 2 | 11 | SAND | -0.0219 | 0.00162 |
| IAC91-1099 24 hpi (m.i) | 2 | 12 | SAND | -0.02617 | -0.00109 |
| IAC91-1099 24 hpi (m.i) | 2 | 13 | SAND | -0.02696 | -0.00032 |
| IAC91-1099 24 hpi (m.i) | 2 | 14 | SAND | -0.02807 | 0.000134 |
| IAC91-1099 24 hpi (m.i) | 2 | 15 | SAND | -0.03032 | -0.00055 |
| IAC91-1099 24 hpi (m.i) | 2 | 16 | SAND | -0.0324 | -0.00107 |
| IAC91-1099 24 hpi (m.i) | 2 | 17 | SAND | -0.03419 | -0.00129 |
| IAC91-1099 24 hpi (m.i) | 2 | 18 | SAND | -0.03651 | -0.00206 |
| IAC91-1099 24 hpi (m.i) | 2 | 19 | SAND | -0.03901 | -0.00299 |
| IAC91-1099 24 hpi (m.i) | 2 | 20 | SAND | -0.0385 | -0.00092 |
| IAC91-1099 24 hpi (m.i) | 2 | 21 | SAND | -0.03826 | 0.000886 |
| IAC91-1099 24 hpi (m.i) | 2 | 22 | SAND | -0.03535 | 0.00536 |
| IAC91-1099 24 hpi (m.i) | 2 | 23 | SAND | -0.03084 | 0.011438 |
| IAC91-1099 24 hpi (m.i) | 2 | 24 | SAND | -0.0205 | 0.023338 |
| IAC91-1099 24 hpi (m.i) | 2 | 25 | SAND | 0.000825 | 0.046229 |
| IAC91-1099 24 hpi (m.i) | 2 | 26 | SAND | 0.043877 | 0.090844 |
| IAC91-1099 24 hpi (m.i) | 2 | 27 | SAND | 0.127104 | 0.175634 |
| IAC91-1099 24 hpi (m.i) | 2 | 28 | SAND | 0.293377 | 0.34347 |
| IAC91-1099 24 hpi (m.i) | 2 | 29 | SAND | 0.594357 | 0.646014 |
| IAC91-1099 24 hpi (m.i) | 2 | 30 | SAND | 1.121448 | 1.174668 |
| IAC91-1099 24 hpi (m.i) | 2 | 31 | SAND | 1.916003 | 1.970786 |
| IAC91-1099 24 hpi (m.i) | 2 | 32 | SAND | 2.778945 | 2.835292 |
| IAC91-1099 24 hpi (m.i) | 2 | 33 | SAND | 3.588418 | 3.646328 |
| IAC91-1099 24 hpi (m.i) | 2 | 34 | SAND | 4.320098 | 4.379572 |
| IAC91-1099 24 hpi (m.i) | 2 | 35 | SAND | 4.943229 | 5.004266 |
| IAC91-1099 24 hpi (m.i) | 2 | 36 | SAND | 5.41925 | 5.48185 |
| IAC91-1099 24 hpi (m.i) | 2 | 37 | SAND | 5.777455 | 5.841619 |
| IAC91-1099 24 hpi (m.i) | 2 | 38 | SAND | 6.09901 | 6.164736 |
| IAC91-1099 24 hpi (m.i) | 2 | 39 | SAND | 6.289782 | 6.357072 |
| IAC91-1099 24 hpi (m.i) | 2 | 40 | SAND | 6.443903 | 6.512757 |
| IAC91-1099 24 hpi (m.i) | 3 | 1 | SAND | 0.040831 | 0.025587 |
| IAC91-1099 24 hpi (m.i) | 3 | 2 | SAND | 0.022945 | 0.008928 |
| IAC91-1099 24 hpi (m.i) | 3 | 3 | SAND | 0.01731 | 0.004519 |
| IAC91-1099 24 hpi (m.i) | 3 | 4 | SAND | 0.015457 | 0.003892 |
| IAC91-1099 24 hpi (m.i) | 3 | 5 | SAND | 0.012315 | 0.001976 |
| IAC91-1099 24 hpi (m.i) | 3 | 6 | SAND | 0.011312 | 0.002199 |
| IAC91-1099 24 hpi (m.i) | 3 | 7 | SAND | 0.00931 | 0.001423 |
| IAC91-1099 24 hpi (m.i) | 3 | 8 | SAND | 0.007568 | 0.000908 |
| IAC91-1099 24 hpi (m.i) | 3 | 9 | SAND | 0.005297 | -0.00014 |
| IAC91-1099 24 hpi (m.i) | 3 | 10 | SAND | 0.001928 | -0.00228 |
| IAC91-1099 24 hpi (m.i) | 3 | 11 | SAND | -0.00041 | -0.00339 |
| IAC91-1099 24 hpi (m.i) | 3 | 12 | SAND | -0.00131 | -0.00307 |
| IAC91-1099 24 hpi (m.i) | 3 | 13 | SAND | -0.00207 | -0.0026 |
| IAC91-1099 24 hpi (m.i) | 3 | 14 | SAND | -0.00346 | -0.00276 |
| IAC91-1099 24 hpi (m.i) | 3 | 15 | SAND | -0.0077 | -0.00578 |
| IAC91-1099 24 hpi (m.i) | 3 | 16 | SAND | -0.00779 | -0.00464 |
| IAC91-1099 24 hpi (m.i) | 3 | 17 | SAND | -0.00847 | -0.00409 |
| IAC91-1099 24 hpi (m.i) | 3 | 18 | SAND | -0.00805 | -0.00244 |
| IAC91-1099 24 hpi (m.i) | 3 | 19 | SAND | -0.00909 | -0.00226 |
| IAC91-1099 24 hpi (m.i) | 3 | 20 | SAND | -0.00856 | -0.00051 |
| IAC91-1099 24 hpi (m.i) | 3 | 21 | SAND | -0.00774 | 0.001543 |
| IAC91-1099 24 hpi (m.i) | 3 | 22 | SAND | -0.00434 | 0.00617 |
| IAC91-1099 24 hpi (m.i) | 3 | 23 | SAND | -0.0004 | 0.011333 |
| IAC91-1099 24 hpi (m.i) | 3 | 24 | SAND | 0.009038 | 0.021997 |
| IAC91-1099 24 hpi (m.i) | 3 | 25 | SAND | 0.030255 | 0.04444 |
| IAC91-1099 24 hpi (m.i) | 3 | 26 | SAND | 0.067773 | 0.083184 |
| IAC91-1099 24 hpi (m.i) | 3 | 27 | SAND | 0.141968 | 0.158605 |
| IAC91-1099 24 hpi (m.i) | 3 | 28 | SAND | 0.297387 | 0.31525 |
| IAC91-1099 24 hpi (m.i) | 3 | 29 | SAND | 0.574381 | 0.593471 |
| IAC91-1099 24 hpi (m.i) | 3 | 30 | SAND | 1.070363 | 1.090679 |
| IAC91-1099 24 hpi (m.i) | 3 | 31 | SAND | 1.840917 | 1.862459 |
| IAC91-1099 24 hpi (m.i) | 3 | 32 | SAND | 2.731667 | 2.754435 |
| IAC91-1099 24 hpi (m.i) | 3 | 33 | SAND | 3.56471 | 3.588705 |
| IAC91-1099 24 hpi (m.i) | 3 | 34 | SAND | 4.310773 | 4.335994 |
| IAC91-1099 24 hpi (m.i) | 3 | 35 | SAND | 4.94688 | 4.973327 |
| IAC91-1099 24 hpi (m.i) | 3 | 36 | SAND | 5.426028 | 5.453701 |
| IAC91-1099 24 hpi (m.i) | 3 | 37 | SAND | 5.784332 | 5.813232 |
| IAC91-1099 24 hpi (m.i) | 3 | 38 | SAND | 6.095498 | 6.125624 |
| IAC91-1099 24 hpi (m.i) | 3 | 39 | SAND | 6.257948 | 6.2893 |
| IAC91-1099 24 hpi (m.i) | 3 | 40 | SAND | 6.404635 | 6.437213 |
| IAC91-1099 24 hpi (s.i) | 1 | 1 | SAND | -0.2838 | -0.01491 |
| IAC91-1099 24 hpi (s.i) | 1 | 2 | SAND | -0.27689 | -0.00727 |
| IAC91-1099 24 hpi (s.i) | 1 | 3 | SAND | -0.27313 | -0.00278 |
| IAC91-1099 24 hpi (s.i) | 1 | 4 | SAND | -0.27053 | 0.000548 |
| IAC91-1099 24 hpi (s.i) | 1 | 5 | SAND | -0.274 | -0.0022 |
| IAC91-1099 24 hpi (s.i) | 1 | 6 | SAND | -0.27264 | -0.00011 |
| IAC91-1099 24 hpi (s.i) | 1 | 7 | SAND | -0.27226 | 0.001006 |
| IAC91-1099 24 hpi (s.i) | 1 | 8 | SAND | -0.27064 | 0.003354 |
| IAC91-1099 24 hpi (s.i) | 1 | 9 | SAND | -0.27265 | 0.002076 |
| IAC91-1099 24 hpi (s.i) | 1 | 10 | SAND | -0.27562 | -0.00017 |
| IAC91-1099 24 hpi (s.i) | 1 | 11 | SAND | -0.27617 | 6.1E-06 |
| IAC91-1099 24 hpi (s.i) | 1 | 12 | SAND | -0.27844 | -0.00154 |
| IAC91-1099 24 hpi (s.i) | 1 | 13 | SAND | -0.27515 | 0.002485 |
| IAC91-1099 24 hpi (s.i) | 1 | 14 | SAND | -0.27799 | 0.000377 |
| IAC91-1099 24 hpi (s.i) | 1 | 15 | SAND | -0.27906 | 3.1E-05 |
| IAC91-1099 24 hpi (s.i) | 1 | 16 | SAND | -0.28126 | -0.00144 |
| IAC91-1099 24 hpi (s.i) | 1 | 17 | SAND | -0.2797 | 0.000853 |
| IAC91-1099 24 hpi (s.i) | 1 | 18 | SAND | -0.28132 | -4.4E-05 |
| IAC91-1099 24 hpi (s.i) | 1 | 19 | SAND | -0.28186 | 0.000152 |
| IAC91-1099 24 hpi (s.i) | 1 | 20 | SAND | -0.28693 | -0.0042 |
| IAC91-1099 24 hpi (s.i) | 1 | 21 | SAND | -0.28435 | -0.00088 |
| IAC91-1099 24 hpi (s.i) | 1 | 22 | SAND | -0.28367 | 0.000522 |
| IAC91-1099 24 hpi (s.i) | 1 | 23 | SAND | -0.28298 | 0.001943 |
| IAC91-1099 24 hpi (s.i) | 1 | 24 | SAND | -0.27644 | 0.009212 |
| IAC91-1099 24 hpi (s.i) | 1 | 25 | SAND | -0.25834 | 0.028036 |
| IAC91-1099 24 hpi (s.i) | 1 | 26 | SAND | -0.22763 | 0.059481 |
| IAC91-1099 24 hpi (s.i) | 1 | 27 | SAND | -0.16504 | 0.122802 |
| IAC91-1099 24 hpi (s.i) | 1 | 28 | SAND | -0.03918 | 0.249388 |
| IAC91-1099 24 hpi (s.i) | 1 | 29 | SAND | 0.188949 | 0.478244 |
| IAC91-1099 24 hpi (s.i) | 1 | 30 | SAND | 0.604868 | 0.894892 |
| IAC91-1099 24 hpi (s.i) | 1 | 31 | SAND | 1.260016 | 1.550769 |
| IAC91-1099 24 hpi (s.i) | 1 | 32 | SAND | 2.015392 | 2.306874 |
| IAC91-1099 24 hpi (s.i) | 1 | 33 | SAND | 2.737288 | 3.029498 |
| IAC91-1099 24 hpi (s.i) | 1 | 34 | SAND | 3.404944 | 3.697883 |
| IAC91-1099 24 hpi (s.i) | 1 | 35 | SAND | 3.98986 | 4.283527 |
| IAC91-1099 24 hpi (s.i) | 1 | 36 | SAND | 4.454472 | 4.748868 |
| IAC91-1099 24 hpi (s.i) | 1 | 37 | SAND | 4.817955 | 5.113081 |
| IAC91-1099 24 hpi (s.i) | 1 | 38 | SAND | 5.154089 | 5.449943 |
| IAC91-1099 24 hpi (s.i) | 1 | 39 | SAND | 5.362101 | 5.658683 |
| IAC91-1099 24 hpi (s.i) | 1 | 40 | SAND | 5.553543 | 5.850854 |
| IAC91-1099 24 hpi (s.i) | 2 | 1 | SAND | -0.27253 | -0.01213 |
| IAC91-1099 24 hpi (s.i) | 2 | 2 | SAND | -0.27215 | -0.01111 |
| IAC91-1099 24 hpi (s.i) | 2 | 3 | SAND | -0.26567 | -0.00398 |
| IAC91-1099 24 hpi (s.i) | 2 | 4 | SAND | -0.26153 | 0.000811 |
| IAC91-1099 24 hpi (s.i) | 2 | 5 | SAND | -0.26442 | -0.00144 |
| IAC91-1099 24 hpi (s.i) | 2 | 6 | SAND | -0.26395 | -0.00032 |
| IAC91-1099 24 hpi (s.i) | 2 | 7 | SAND | -0.26333 | 0.000951 |
| IAC91-1099 24 hpi (s.i) | 2 | 8 | SAND | -0.26327 | 0.001655 |
| IAC91-1099 24 hpi (s.i) | 2 | 9 | SAND | -0.26341 | 0.002165 |
| IAC91-1099 24 hpi (s.i) | 2 | 10 | SAND | -0.26582 | 0.0004 |
| IAC91-1099 24 hpi (s.i) | 2 | 11 | SAND | -0.26363 | 0.003232 |
| IAC91-1099 24 hpi (s.i) | 2 | 12 | SAND | -0.2689 | -0.00138 |
| IAC91-1099 24 hpi (s.i) | 2 | 13 | SAND | -0.267 | 0.001157 |
| IAC91-1099 24 hpi (s.i) | 2 | 14 | SAND | -0.26818 | 0.000622 |
| IAC91-1099 24 hpi (s.i) | 2 | 15 | SAND | -0.26933 | 0.00012 |
| IAC91-1099 24 hpi (s.i) | 2 | 16 | SAND | -0.27259 | -0.00249 |
| IAC91-1099 24 hpi (s.i) | 2 | 17 | SAND | -0.26942 | 0.001321 |
| IAC91-1099 24 hpi (s.i) | 2 | 18 | SAND | -0.2717 | -0.00031 |
| IAC91-1099 24 hpi (s.i) | 2 | 19 | SAND | -0.27253 | -0.00049 |
| IAC91-1099 24 hpi (s.i) | 2 | 20 | SAND | -0.2754 | -0.00271 |
| IAC91-1099 24 hpi (s.i) | 2 | 21 | SAND | -0.2747 | -0.00137 |
| IAC91-1099 24 hpi (s.i) | 2 | 22 | SAND | -0.27193 | 0.002053 |
| IAC91-1099 24 hpi (s.i) | 2 | 23 | SAND | -0.27215 | 0.002471 |
| IAC91-1099 24 hpi (s.i) | 2 | 24 | SAND | -0.2618 | 0.013476 |
| IAC91-1099 24 hpi (s.i) | 2 | 25 | SAND | -0.23908 | 0.036841 |
| IAC91-1099 24 hpi (s.i) | 2 | 26 | SAND | -0.20323 | 0.073332 |
| IAC91-1099 24 hpi (s.i) | 2 | 27 | SAND | -0.12899 | 0.148226 |
| IAC91-1099 24 hpi (s.i) | 2 | 28 | SAND | 0.012048 | 0.289907 |
| IAC91-1099 24 hpi (s.i) | 2 | 29 | SAND | 0.277394 | 0.555899 |
| IAC91-1099 24 hpi (s.i) | 2 | 30 | SAND | 0.764966 | 1.044117 |
| IAC91-1099 24 hpi (s.i) | 2 | 31 | SAND | 1.558405 | 1.838204 |
| IAC91-1099 24 hpi (s.i) | 2 | 32 | SAND | 2.548294 | 2.828739 |
| IAC91-1099 24 hpi (s.i) | 2 | 33 | SAND | 3.472469 | 3.753561 |
| IAC91-1099 24 hpi (s.i) | 2 | 34 | SAND | 4.289726 | 4.571465 |
| IAC91-1099 24 hpi (s.i) | 2 | 35 | SAND | 4.994552 | 5.276937 |
| IAC91-1099 24 hpi (s.i) | 2 | 36 | SAND | 5.533924 | 5.816955 |
| IAC91-1099 24 hpi (s.i) | 2 | 37 | SAND | 5.941278 | 6.224957 |
| IAC91-1099 24 hpi (s.i) | 2 | 38 | SAND | 6.27387 | 6.558195 |
| IAC91-1099 24 hpi (s.i) | 2 | 39 | SAND | 6.475358 | 6.76033 |
| IAC91-1099 24 hpi (s.i) | 2 | 40 | SAND | 6.641099 | 6.926718 |
| IAC91-1099 24 hpi (s.i) | 3 | 1 | SAND | -0.27168 | -0.01043 |
| IAC91-1099 24 hpi (s.i) | 3 | 2 | SAND | -0.26671 | -0.00468 |
| IAC91-1099 24 hpi (s.i) | 3 | 3 | SAND | -0.26449 | -0.00168 |
| IAC91-1099 24 hpi (s.i) | 3 | 4 | SAND | -0.26479 | -0.00119 |
| IAC91-1099 24 hpi (s.i) | 3 | 5 | SAND | -0.26069 | 0.003686 |
| IAC91-1099 24 hpi (s.i) | 3 | 6 | SAND | -0.26324 | 0.001914 |
| IAC91-1099 24 hpi (s.i) | 3 | 7 | SAND | -0.2665 | -0.00057 |
| IAC91-1099 24 hpi (s.i) | 3 | 8 | SAND | -0.26573 | 0.000986 |
| IAC91-1099 24 hpi (s.i) | 3 | 9 | SAND | -0.26622 | 0.001277 |
| IAC91-1099 24 hpi (s.i) | 3 | 10 | SAND | -0.26694 | 0.001343 |
| IAC91-1099 24 hpi (s.i) | 3 | 11 | SAND | -0.26957 | -0.00051 |
| IAC91-1099 24 hpi (s.i) | 3 | 12 | SAND | -0.27007 | -0.00023 |
| IAC91-1099 24 hpi (s.i) | 3 | 13 | SAND | -0.2732 | -0.00257 |
| IAC91-1099 24 hpi (s.i) | 3 | 14 | SAND | -0.27257 | -0.00117 |
| IAC91-1099 24 hpi (s.i) | 3 | 15 | SAND | -0.27354 | -0.00135 |
| IAC91-1099 24 hpi (s.i) | 3 | 16 | SAND | -0.27374 | -0.00077 |
| IAC91-1099 24 hpi (s.i) | 3 | 17 | SAND | -0.2779 | -0.00415 |
| IAC91-1099 24 hpi (s.i) | 3 | 18 | SAND | -0.27698 | -0.00245 |
| IAC91-1099 24 hpi (s.i) | 3 | 19 | SAND | -0.27713 | -0.00182 |
| IAC91-1099 24 hpi (s.i) | 3 | 20 | SAND | -0.27271 | 0.003387 |
| IAC91-1099 24 hpi (s.i) | 3 | 21 | SAND | -0.27101 | 0.005864 |
| IAC91-1099 24 hpi (s.i) | 3 | 22 | SAND | -0.26499 | 0.012669 |
| IAC91-1099 24 hpi (s.i) | 3 | 23 | SAND | -0.2517 | 0.026736 |
| IAC91-1099 24 hpi (s.i) | 3 | 24 | SAND | -0.22922 | 0.05 |
| IAC91-1099 24 hpi (s.i) | 3 | 25 | SAND | -0.19061 | 0.089391 |
| IAC91-1099 24 hpi (s.i) | 3 | 26 | SAND | -0.10306 | 0.177723 |
| IAC91-1099 24 hpi (s.i) | 3 | 27 | SAND | 0.05807 | 0.339634 |
| IAC91-1099 24 hpi (s.i) | 3 | 28 | SAND | 0.374489 | 0.656834 |
| IAC91-1099 24 hpi (s.i) | 3 | 29 | SAND | 0.922685 | 1.205811 |
| IAC91-1099 24 hpi (s.i) | 3 | 30 | SAND | 1.795426 | 2.079334 |
| IAC91-1099 24 hpi (s.i) | 3 | 31 | SAND | 2.82201 | 3.106699 |
| IAC91-1099 24 hpi (s.i) | 3 | 32 | SAND | 3.704513 | 3.989984 |
| IAC91-1099 24 hpi (s.i) | 3 | 33 | SAND | 4.450263 | 4.736515 |
| IAC91-1099 24 hpi (s.i) | 3 | 34 | SAND | 5.045685 | 5.332718 |
| IAC91-1099 24 hpi (s.i) | 3 | 35 | SAND | 5.512719 | 5.800534 |
| IAC91-1099 24 hpi (s.i) | 3 | 36 | SAND | 5.836533 | 6.125129 |
| IAC91-1099 24 hpi (s.i) | 3 | 37 | SAND | 6.049426 | 6.338804 |
| IAC91-1099 24 hpi (s.i) | 3 | 38 | SAND | 6.223155 | 6.513314 |
| IAC91-1099 24 hpi (s.i) | 3 | 39 | SAND | 6.290576 | 6.581516 |
| IAC91-1099 24 hpi (s.i) | 3 | 40 | SAND | 6.365135 | 6.656857 |
| IAC91-1099 72 hpi (m.i) | 1 | 1 | SAND | -0.29542 | -0.00964 |
| IAC91-1099 72 hpi (m.i) | 1 | 2 | SAND | -0.29229 | -0.0061 |
| IAC91-1099 72 hpi (m.i) | 1 | 3 | SAND | -0.28946 | -0.00286 |
| IAC91-1099 72 hpi (m.i) | 1 | 4 | SAND | -0.287 | 4.48E-06 |
| IAC91-1099 72 hpi (m.i) | 1 | 5 | SAND | -0.28824 | -0.00083 |
| IAC91-1099 72 hpi (m.i) | 1 | 6 | SAND | -0.28656 | 0.001257 |
| IAC91-1099 72 hpi (m.i) | 1 | 7 | SAND | -0.28576 | 0.002466 |
| IAC91-1099 72 hpi (m.i) | 1 | 8 | SAND | -0.28759 | 0.001047 |
| IAC91-1099 72 hpi (m.i) | 1 | 9 | SAND | -0.28822 | 0.000822 |
| IAC91-1099 72 hpi (m.i) | 1 | 10 | SAND | -0.28962 | -0.00017 |
| IAC91-1099 72 hpi (m.i) | 1 | 11 | SAND | -0.28864 | 0.001225 |
| IAC91-1099 72 hpi (m.i) | 1 | 12 | SAND | -0.29017 | 9.41E-05 |
| IAC91-1099 72 hpi (m.i) | 1 | 13 | SAND | -0.29009 | 0.000583 |
| IAC91-1099 72 hpi (m.i) | 1 | 14 | SAND | -0.29 | 0.001079 |
| IAC91-1099 72 hpi (m.i) | 1 | 15 | SAND | -0.29267 | -0.00118 |
| IAC91-1099 72 hpi (m.i) | 1 | 16 | SAND | -0.29313 | -0.00123 |
| IAC91-1099 72 hpi (m.i) | 1 | 17 | SAND | -0.29215 | 0.000158 |
| IAC91-1099 72 hpi (m.i) | 1 | 18 | SAND | -0.29396 | -0.00124 |
| IAC91-1099 72 hpi (m.i) | 1 | 19 | SAND | -0.29614 | -0.00302 |
| IAC91-1099 72 hpi (m.i) | 1 | 20 | SAND | -0.29599 | -0.00246 |
| IAC91-1099 72 hpi (m.i) | 1 | 21 | SAND | -0.29547 | -0.00153 |
| IAC91-1099 72 hpi (m.i) | 1 | 22 | SAND | -0.29366 | 0.000691 |
| IAC91-1099 72 hpi (m.i) | 1 | 23 | SAND | -0.28967 | 0.005085 |
| IAC91-1099 72 hpi (m.i) | 1 | 24 | SAND | -0.28244 | 0.012724 |
| IAC91-1099 72 hpi (m.i) | 1 | 25 | SAND | -0.26849 | 0.027078 |
| IAC91-1099 72 hpi (m.i) | 1 | 26 | SAND | -0.23984 | 0.056142 |
| IAC91-1099 72 hpi (m.i) | 1 | 27 | SAND | -0.18413 | 0.112253 |
| IAC91-1099 72 hpi (m.i) | 1 | 28 | SAND | -0.06809 | 0.228708 |
| IAC91-1099 72 hpi (m.i) | 1 | 29 | SAND | 0.141814 | 0.439016 |
| IAC91-1099 72 hpi (m.i) | 1 | 30 | SAND | 0.535249 | 0.832859 |
| IAC91-1099 72 hpi (m.i) | 1 | 31 | SAND | 1.194769 | 1.492787 |
| IAC91-1099 72 hpi (m.i) | 1 | 32 | SAND | 2.115054 | 2.413479 |
| IAC91-1099 72 hpi (m.i) | 1 | 33 | SAND | 3.043104 | 3.341937 |
| IAC91-1099 72 hpi (m.i) | 1 | 34 | SAND | 3.87413 | 4.173371 |
| IAC91-1099 72 hpi (m.i) | 1 | 35 | SAND | 4.614429 | 4.914078 |
| IAC91-1099 72 hpi (m.i) | 1 | 36 | SAND | 5.18667 | 5.486727 |
| IAC91-1099 72 hpi (m.i) | 1 | 37 | SAND | 5.63916 | 5.939624 |
| IAC91-1099 72 hpi (m.i) | 1 | 38 | SAND | 6.026624 | 6.327496 |
| IAC91-1099 72 hpi (m.i) | 1 | 39 | SAND | 6.2701 | 6.57138 |
| IAC91-1099 72 hpi (m.i) | 1 | 40 | SAND | 6.481534 | 6.783221 |
| IAC91-1099 72 hpi (m.i) | 2 | 1 | SAND | -0.29702 | -0.00851 |
| IAC91-1099 72 hpi (m.i) | 2 | 2 | SAND | -0.29425 | -0.00562 |
| IAC91-1099 72 hpi (m.i) | 2 | 3 | SAND | -0.29093 | -0.00218 |
| IAC91-1099 72 hpi (m.i) | 2 | 4 | SAND | -0.2886 | 0.00027 |
| IAC91-1099 72 hpi (m.i) | 2 | 5 | SAND | -0.2879 | 0.001093 |
| IAC91-1099 72 hpi (m.i) | 2 | 6 | SAND | -0.28741 | 0.001702 |
| IAC91-1099 72 hpi (m.i) | 2 | 7 | SAND | -0.2872 | 0.002039 |
| IAC91-1099 72 hpi (m.i) | 2 | 8 | SAND | -0.28878 | 0.000581 |
| IAC91-1099 72 hpi (m.i) | 2 | 9 | SAND | -0.28875 | 0.000736 |
| IAC91-1099 72 hpi (m.i) | 2 | 10 | SAND | -0.28922 | 0.000386 |
| IAC91-1099 72 hpi (m.i) | 2 | 11 | SAND | -0.28938 | 0.000342 |
| IAC91-1099 72 hpi (m.i) | 2 | 12 | SAND | -0.29079 | -0.00094 |
| IAC91-1099 72 hpi (m.i) | 2 | 13 | SAND | -0.29114 | -0.00117 |
| IAC91-1099 72 hpi (m.i) | 2 | 14 | SAND | -0.29098 | -0.00089 |
| IAC91-1099 72 hpi (m.i) | 2 | 15 | SAND | -0.2919 | -0.00169 |
| IAC91-1099 72 hpi (m.i) | 2 | 16 | SAND | -0.29229 | -0.00195 |
| IAC91-1099 72 hpi (m.i) | 2 | 17 | SAND | -0.29058 | -0.00013 |
| IAC91-1099 72 hpi (m.i) | 2 | 18 | SAND | -0.29211 | -0.00153 |
| IAC91-1099 72 hpi (m.i) | 2 | 19 | SAND | -0.29319 | -0.00248 |
| IAC91-1099 72 hpi (m.i) | 2 | 20 | SAND | -0.29207 | -0.00124 |
| IAC91-1099 72 hpi (m.i) | 2 | 21 | SAND | -0.28971 | 0.001237 |
| IAC91-1099 72 hpi (m.i) | 2 | 22 | SAND | -0.28526 | 0.005807 |
| IAC91-1099 72 hpi (m.i) | 2 | 23 | SAND | -0.28158 | 0.009613 |
| IAC91-1099 72 hpi (m.i) | 2 | 24 | SAND | -0.27022 | 0.021094 |
| IAC91-1099 72 hpi (m.i) | 2 | 25 | SAND | -0.25015 | 0.041286 |
| IAC91-1099 72 hpi (m.i) | 2 | 26 | SAND | -0.20706 | 0.084497 |
| IAC91-1099 72 hpi (m.i) | 2 | 27 | SAND | -0.124 | 0.167679 |
| IAC91-1099 72 hpi (m.i) | 2 | 28 | SAND | 0.041218 | 0.333018 |
| IAC91-1099 72 hpi (m.i) | 2 | 29 | SAND | 0.342581 | 0.634503 |
| IAC91-1099 72 hpi (m.i) | 2 | 30 | SAND | 0.883256 | 1.1753 |
| IAC91-1099 72 hpi (m.i) | 2 | 31 | SAND | 1.729251 | 2.021418 |
| IAC91-1099 72 hpi (m.i) | 2 | 32 | SAND | 2.745531 | 3.037819 |
| IAC91-1099 72 hpi (m.i) | 2 | 33 | SAND | 3.654102 | 3.946512 |
| IAC91-1099 72 hpi (m.i) | 2 | 34 | SAND | 4.437976 | 4.730508 |
| IAC91-1099 72 hpi (m.i) | 2 | 35 | SAND | 5.094284 | 5.386938 |
| IAC91-1099 72 hpi (m.i) | 2 | 36 | SAND | 5.574841 | 5.867618 |
| IAC91-1099 72 hpi (m.i) | 2 | 37 | SAND | 5.943509 | 6.236408 |
| IAC91-1099 72 hpi (m.i) | 2 | 38 | SAND | 6.242287 | 6.535308 |
| IAC91-1099 72 hpi (m.i) | 2 | 39 | SAND | 6.416018 | 6.70916 |
| IAC91-1099 72 hpi (m.i) | 2 | 40 | SAND | 6.561111 | 6.854375 |
| IAC91-1099 72 hpi (m.i) | 3 | 1 | SAND | -0.36779 | -0.0107 |
| IAC91-1099 72 hpi (m.i) | 3 | 2 | SAND | -0.3634 | -0.00609 |
| IAC91-1099 72 hpi (m.i) | 3 | 3 | SAND | -0.3602 | -0.00268 |
| IAC91-1099 72 hpi (m.i) | 3 | 4 | SAND | -0.35701 | 0.000732 |
| IAC91-1099 72 hpi (m.i) | 3 | 5 | SAND | -0.35711 | 0.000844 |
| IAC91-1099 72 hpi (m.i) | 3 | 6 | SAND | -0.35691 | 0.001259 |
| IAC91-1099 72 hpi (m.i) | 3 | 7 | SAND | -0.357 | 0.001386 |
| IAC91-1099 72 hpi (m.i) | 3 | 8 | SAND | -0.35742 | 0.00118 |
| IAC91-1099 72 hpi (m.i) | 3 | 9 | SAND | -0.35724 | 0.001572 |
| IAC91-1099 72 hpi (m.i) | 3 | 10 | SAND | -0.35829 | 0.000739 |
| IAC91-1099 72 hpi (m.i) | 3 | 11 | SAND | -0.3593 | -5.2E-05 |
| IAC91-1099 72 hpi (m.i) | 3 | 12 | SAND | -0.36101 | -0.00154 |
| IAC91-1099 72 hpi (m.i) | 3 | 13 | SAND | -0.35995 | -0.00027 |
| IAC91-1099 72 hpi (m.i) | 3 | 14 | SAND | -0.35987 | 2.24E-05 |
| IAC91-1099 72 hpi (m.i) | 3 | 15 | SAND | -0.36159 | -0.00148 |
| IAC91-1099 72 hpi (m.i) | 3 | 16 | SAND | -0.36099 | -0.00066 |
| IAC91-1099 72 hpi (m.i) | 3 | 17 | SAND | -0.36283 | -0.00229 |
| IAC91-1099 72 hpi (m.i) | 3 | 18 | SAND | -0.36198 | -0.00122 |
| IAC91-1099 72 hpi (m.i) | 3 | 19 | SAND | -0.36308 | -0.00211 |
| IAC91-1099 72 hpi (m.i) | 3 | 20 | SAND | -0.36272 | -0.00153 |
| IAC91-1099 72 hpi (m.i) | 3 | 21 | SAND | -0.36209 | -0.00068 |
| IAC91-1099 72 hpi (m.i) | 3 | 22 | SAND | -0.35914 | 0.002484 |
| IAC91-1099 72 hpi (m.i) | 3 | 23 | SAND | -0.35753 | 0.004308 |
| IAC91-1099 72 hpi (m.i) | 3 | 24 | SAND | -0.34953 | 0.012522 |
| IAC91-1099 72 hpi (m.i) | 3 | 25 | SAND | -0.33422 | 0.028047 |
| IAC91-1099 72 hpi (m.i) | 3 | 26 | SAND | -0.30379 | 0.058694 |
| IAC91-1099 72 hpi (m.i) | 3 | 27 | SAND | -0.24297 | 0.119724 |
| IAC91-1099 72 hpi (m.i) | 3 | 28 | SAND | -0.12034 | 0.242568 |
| IAC91-1099 72 hpi (m.i) | 3 | 29 | SAND | 0.108953 | 0.472081 |
| IAC91-1099 72 hpi (m.i) | 3 | 30 | SAND | 0.531212 | 0.894556 |
| IAC91-1099 72 hpi (m.i) | 3 | 31 | SAND | 1.228048 | 1.591608 |
| IAC91-1099 72 hpi (m.i) | 3 | 32 | SAND | 2.134152 | 2.497928 |
| IAC91-1099 72 hpi (m.i) | 3 | 33 | SAND | 3.002227 | 3.366218 |
| IAC91-1099 72 hpi (m.i) | 3 | 34 | SAND | 3.781838 | 4.146044 |
| IAC91-1099 72 hpi (m.i) | 3 | 35 | SAND | 4.475596 | 4.840018 |
| IAC91-1099 72 hpi (m.i) | 3 | 36 | SAND | 5.008441 | 5.373079 |
| IAC91-1099 72 hpi (m.i) | 3 | 37 | SAND | 5.427428 | 5.792281 |
| IAC91-1099 72 hpi (m.i) | 3 | 38 | SAND | 5.783162 | 6.148232 |
| IAC91-1099 72 hpi (m.i) | 3 | 39 | SAND | 5.993293 | 6.358578 |
| IAC91-1099 72 hpi (m.i) | 3 | 40 | SAND | 6.185423 | 6.550923 |
| IAC91-1099 72 hpi (s.i) | 1 | 1 | SAND | -0.28475 | -0.00458 |
| IAC91-1099 72 hpi (s.i) | 1 | 2 | SAND | -0.2836 | -0.00339 |
| IAC91-1099 72 hpi (s.i) | 1 | 3 | SAND | -0.28598 | -0.00574 |
| IAC91-1099 72 hpi (s.i) | 1 | 4 | SAND | -0.28067 | -0.0004 |
| IAC91-1099 72 hpi (s.i) | 1 | 5 | SAND | -0.28257 | -0.00227 |
| IAC91-1099 72 hpi (s.i) | 1 | 6 | SAND | -0.28137 | -0.00104 |
| IAC91-1099 72 hpi (s.i) | 1 | 7 | SAND | -0.27795 | 0.002414 |
| IAC91-1099 72 hpi (s.i) | 1 | 8 | SAND | -0.2763 | 0.004091 |
| IAC91-1099 72 hpi (s.i) | 1 | 9 | SAND | -0.27764 | 0.002783 |
| IAC91-1099 72 hpi (s.i) | 1 | 10 | SAND | -0.27511 | 0.005345 |
| IAC91-1099 72 hpi (s.i) | 1 | 11 | SAND | -0.28074 | -0.00025 |
| IAC91-1099 72 hpi (s.i) | 1 | 12 | SAND | -0.2785 | 0.002025 |
| IAC91-1099 72 hpi (s.i) | 1 | 13 | SAND | -0.28003 | 0.000528 |
| IAC91-1099 72 hpi (s.i) | 1 | 14 | SAND | -0.28119 | -0.0006 |
| IAC91-1099 72 hpi (s.i) | 1 | 15 | SAND | -0.2784 | 0.002217 |
| IAC91-1099 72 hpi (s.i) | 1 | 16 | SAND | -0.28173 | -0.00108 |
| IAC91-1099 72 hpi (s.i) | 1 | 17 | SAND | -0.28516 | -0.00448 |
| IAC91-1099 72 hpi (s.i) | 1 | 18 | SAND | -0.28253 | -0.00181 |
| IAC91-1099 72 hpi (s.i) | 1 | 19 | SAND | -0.28355 | -0.00281 |
| IAC91-1099 72 hpi (s.i) | 1 | 20 | SAND | -0.28348 | -0.00271 |
| IAC91-1099 72 hpi (s.i) | 1 | 21 | SAND | -0.28247 | -0.00167 |
| IAC91-1099 72 hpi (s.i) | 1 | 22 | SAND | -0.27905 | 0.001792 |
| IAC91-1099 72 hpi (s.i) | 1 | 23 | SAND | -0.27721 | 0.003656 |
| IAC91-1099 72 hpi (s.i) | 1 | 24 | SAND | -0.26815 | 0.012746 |
| IAC91-1099 72 hpi (s.i) | 1 | 25 | SAND | -0.25245 | 0.028482 |
| IAC91-1099 72 hpi (s.i) | 1 | 26 | SAND | -0.22143 | 0.059533 |
| IAC91-1099 72 hpi (s.i) | 1 | 27 | SAND | -0.16244 | 0.118558 |
| IAC91-1099 72 hpi (s.i) | 1 | 28 | SAND | -0.03967 | 0.241361 |
| IAC91-1099 72 hpi (s.i) | 1 | 29 | SAND | 0.190575 | 0.471634 |
| IAC91-1099 72 hpi (s.i) | 1 | 30 | SAND | 0.607171 | 0.888262 |
| IAC91-1099 72 hpi (s.i) | 1 | 31 | SAND | 1.275977 | 1.557099 |
| IAC91-1099 72 hpi (s.i) | 1 | 32 | SAND | 2.061795 | 2.342948 |
| IAC91-1099 72 hpi (s.i) | 1 | 33 | SAND | 2.816875 | 3.09806 |
| IAC91-1099 72 hpi (s.i) | 1 | 34 | SAND | 3.536155 | 3.817372 |
| IAC91-1099 72 hpi (s.i) | 1 | 35 | SAND | 4.142858 | 4.424107 |
| IAC91-1099 72 hpi (s.i) | 1 | 36 | SAND | 4.627026 | 4.908306 |
| IAC91-1099 72 hpi (s.i) | 1 | 37 | SAND | 5.047666 | 5.328978 |
| IAC91-1099 72 hpi (s.i) | 1 | 38 | SAND | 5.387207 | 5.66855 |
| IAC91-1099 72 hpi (s.i) | 1 | 39 | SAND | 5.606438 | 5.887813 |
| IAC91-1099 72 hpi (s.i) | 1 | 40 | SAND | 5.837401 | 6.118808 |
| IAC91-1099 72 hpi (s.i) | 2 | 1 | SAND | -0.33453 | -0.00184 |
| IAC91-1099 72 hpi (s.i) | 2 | 2 | SAND | -0.33001 | 0.002465 |
| IAC91-1099 72 hpi (s.i) | 2 | 3 | SAND | -0.34 | -0.00773 |
| IAC91-1099 72 hpi (s.i) | 2 | 4 | SAND | -0.32715 | 0.004914 |
| IAC91-1099 72 hpi (s.i) | 2 | 5 | SAND | -0.33641 | -0.00456 |
| IAC91-1099 72 hpi (s.i) | 2 | 6 | SAND | -0.33624 | -0.0046 |
| IAC91-1099 72 hpi (s.i) | 2 | 7 | SAND | -0.32593 | 0.005505 |
| IAC91-1099 72 hpi (s.i) | 2 | 8 | SAND | -0.32632 | 0.004903 |
| IAC91-1099 72 hpi (s.i) | 2 | 9 | SAND | -0.32799 | 0.003031 |
| IAC91-1099 72 hpi (s.i) | 2 | 10 | SAND | -0.32635 | 0.004462 |
| IAC91-1099 72 hpi (s.i) | 2 | 11 | SAND | -0.33091 | -0.00031 |
| IAC91-1099 72 hpi (s.i) | 2 | 12 | SAND | -0.32872 | 0.00167 |
| IAC91-1099 72 hpi (s.i) | 2 | 13 | SAND | -0.33199 | -0.00181 |
| IAC91-1099 72 hpi (s.i) | 2 | 14 | SAND | -0.32596 | 0.00402 |
| IAC91-1099 72 hpi (s.i) | 2 | 15 | SAND | -0.32913 | 0.000638 |
| IAC91-1099 72 hpi (s.i) | 2 | 16 | SAND | -0.33615 | -0.00659 |
| IAC91-1099 72 hpi (s.i) | 2 | 17 | SAND | -0.33196 | -0.00261 |
| IAC91-1099 72 hpi (s.i) | 2 | 18 | SAND | -0.32922 | -7.8E-05 |
| IAC91-1099 72 hpi (s.i) | 2 | 19 | SAND | -0.33417 | -0.00523 |
| IAC91-1099 72 hpi (s.i) | 2 | 20 | SAND | -0.33037 | -0.00164 |
| IAC91-1099 72 hpi (s.i) | 2 | 21 | SAND | -0.32503 | 0.003486 |
| IAC91-1099 72 hpi (s.i) | 2 | 22 | SAND | -0.32578 | 0.002527 |
| IAC91-1099 72 hpi (s.i) | 2 | 23 | SAND | -0.32315 | 0.00495 |
| IAC91-1099 72 hpi (s.i) | 2 | 24 | SAND | -0.31141 | 0.016478 |
| IAC91-1099 72 hpi (s.i) | 2 | 25 | SAND | -0.29233 | 0.035352 |
| IAC91-1099 72 hpi (s.i) | 2 | 26 | SAND | -0.25563 | 0.071848 |
| IAC91-1099 72 hpi (s.i) | 2 | 27 | SAND | -0.18363 | 0.143641 |
| IAC91-1099 72 hpi (s.i) | 2 | 28 | SAND | -0.03925 | 0.287809 |
| IAC91-1099 72 hpi (s.i) | 2 | 29 | SAND | 0.224881 | 0.55173 |
| IAC91-1099 72 hpi (s.i) | 2 | 30 | SAND | 0.723476 | 1.050116 |
| IAC91-1099 72 hpi (s.i) | 2 | 31 | SAND | 1.498613 | 1.825045 |
| IAC91-1099 72 hpi (s.i) | 2 | 32 | SAND | 2.462486 | 2.788709 |
| IAC91-1099 72 hpi (s.i) | 2 | 33 | SAND | 3.345497 | 3.671512 |
| IAC91-1099 72 hpi (s.i) | 2 | 34 | SAND | 4.142742 | 4.468548 |
| IAC91-1099 72 hpi (s.i) | 2 | 35 | SAND | 4.820078 | 5.145677 |
| IAC91-1099 72 hpi (s.i) | 2 | 36 | SAND | 5.3454 | 5.67079 |
| IAC91-1099 72 hpi (s.i) | 2 | 37 | SAND | 5.75913 | 6.084312 |
| IAC91-1099 72 hpi (s.i) | 2 | 38 | SAND | 6.119473 | 6.444447 |
| IAC91-1099 72 hpi (s.i) | 2 | 39 | SAND | 6.33958 | 6.664345 |
| IAC91-1099 72 hpi (s.i) | 2 | 40 | SAND | 6.552635 | 6.877191 |
| IAC91-1099 72 hpi (s.i) | 3 | 1 | SAND | -0.27467 | -0.0146 |
| IAC91-1099 72 hpi (s.i) | 3 | 2 | SAND | -0.27116 | -0.01029 |
| IAC91-1099 72 hpi (s.i) | 3 | 3 | SAND | -0.26148 | 0.000185 |
| IAC91-1099 72 hpi (s.i) | 3 | 4 | SAND | -0.26605 | -0.0036 |
| IAC91-1099 72 hpi (s.i) | 3 | 5 | SAND | -0.25924 | 0.004005 |
| IAC91-1099 72 hpi (s.i) | 3 | 6 | SAND | -0.25888 | 0.005165 |
| IAC91-1099 72 hpi (s.i) | 3 | 7 | SAND | -0.26589 | -0.00106 |
| IAC91-1099 72 hpi (s.i) | 3 | 8 | SAND | -0.26501 | 0.000617 |
| IAC91-1099 72 hpi (s.i) | 3 | 9 | SAND | -0.26708 | -0.00066 |
| IAC91-1099 72 hpi (s.i) | 3 | 10 | SAND | -0.27028 | -0.00307 |
| IAC91-1099 72 hpi (s.i) | 3 | 11 | SAND | -0.26811 | -0.0001 |
| IAC91-1099 72 hpi (s.i) | 3 | 12 | SAND | -0.27015 | -0.00135 |
| IAC91-1099 72 hpi (s.i) | 3 | 13 | SAND | -0.26948 | 0.000112 |
| IAC91-1099 72 hpi (s.i) | 3 | 14 | SAND | -0.26967 | 0.000714 |
| IAC91-1099 72 hpi (s.i) | 3 | 15 | SAND | -0.27394 | -0.00276 |
| IAC91-1099 72 hpi (s.i) | 3 | 16 | SAND | -0.26713 | 0.004844 |
| IAC91-1099 72 hpi (s.i) | 3 | 17 | SAND | -0.27382 | -0.00105 |
| IAC91-1099 72 hpi (s.i) | 3 | 18 | SAND | -0.27693 | -0.00337 |
| IAC91-1099 72 hpi (s.i) | 3 | 19 | SAND | -0.27761 | -0.00326 |
| IAC91-1099 72 hpi (s.i) | 3 | 20 | SAND | -0.2778 | -0.00266 |
| IAC91-1099 72 hpi (s.i) | 3 | 21 | SAND | -0.27564 | 0.000298 |
| IAC91-1099 72 hpi (s.i) | 3 | 22 | SAND | -0.2749 | 0.001831 |
| IAC91-1099 72 hpi (s.i) | 3 | 23 | SAND | -0.27236 | 0.005169 |
| IAC91-1099 72 hpi (s.i) | 3 | 24 | SAND | -0.26379 | 0.014522 |
| IAC91-1099 72 hpi (s.i) | 3 | 25 | SAND | -0.24464 | 0.034472 |
| IAC91-1099 72 hpi (s.i) | 3 | 26 | SAND | -0.20565 | 0.074257 |
| IAC91-1099 72 hpi (s.i) | 3 | 27 | SAND | -0.13379 | 0.146909 |
| IAC91-1099 72 hpi (s.i) | 3 | 28 | SAND | 0.015928 | 0.297417 |
| IAC91-1099 72 hpi (s.i) | 3 | 29 | SAND | 0.291619 | 0.573902 |
| IAC91-1099 72 hpi (s.i) | 3 | 30 | SAND | 0.76607 | 1.049146 |
| IAC91-1099 72 hpi (s.i) | 3 | 31 | SAND | 1.519093 | 1.802962 |
| IAC91-1099 72 hpi (s.i) | 3 | 32 | SAND | 2.389518 | 2.67418 |
| IAC91-1099 72 hpi (s.i) | 3 | 33 | SAND | 3.201558 | 3.487013 |
| IAC91-1099 72 hpi (s.i) | 3 | 34 | SAND | 3.91406 | 4.200308 |
| IAC91-1099 72 hpi (s.i) | 3 | 35 | SAND | 4.540481 | 4.827522 |
| IAC91-1099 72 hpi (s.i) | 3 | 36 | SAND | 5.029315 | 5.317149 |
| IAC91-1099 72 hpi (s.i) | 3 | 37 | SAND | 5.395578 | 5.684206 |
| IAC91-1099 72 hpi (s.i) | 3 | 38 | SAND | 5.729131 | 6.018552 |
| IAC91-1099 72 hpi (s.i) | 3 | 39 | SAND | 5.940438 | 6.230652 |
| IAC91-1099 72 hpi (s.i) | 3 | 40 | SAND | 6.126747 | 6.417754 |
| IACSP95-5000 24 hpi (m.i) | 1 | 1 | SAND | -0.28046 | -0.00671 |
| IACSP95-5000 24 hpi (m.i) | 1 | 2 | SAND | -0.27759 | -0.00359 |
| IACSP95-5000 24 hpi (m.i) | 1 | 3 | SAND | -0.27704 | -0.00278 |
| IACSP95-5000 24 hpi (m.i) | 1 | 4 | SAND | -0.27512 | -0.0006 |
| IACSP95-5000 24 hpi (m.i) | 1 | 5 | SAND | -0.27457 | 0.000207 |
| IACSP95-5000 24 hpi (m.i) | 1 | 6 | SAND | -0.27476 | 0.000283 |
| IACSP95-5000 24 hpi (m.i) | 1 | 7 | SAND | -0.27489 | 0.000405 |
| IACSP95-5000 24 hpi (m.i) | 1 | 8 | SAND | -0.27589 | -0.00034 |
| IACSP95-5000 24 hpi (m.i) | 1 | 9 | SAND | -0.27436 | 0.001453 |
| IACSP95-5000 24 hpi (m.i) | 1 | 10 | SAND | -0.27598 | 9.31E-05 |
| IACSP95-5000 24 hpi (m.i) | 1 | 11 | SAND | -0.27485 | 0.001481 |
| IACSP95-5000 24 hpi (m.i) | 1 | 12 | SAND | -0.2753 | 0.001291 |
| IACSP95-5000 24 hpi (m.i) | 1 | 13 | SAND | -0.27605 | 0.000805 |
| IACSP95-5000 24 hpi (m.i) | 1 | 14 | SAND | -0.27633 | 0.000783 |
| IACSP95-5000 24 hpi (m.i) | 1 | 15 | SAND | -0.27903 | -0.00166 |
| IACSP95-5000 24 hpi (m.i) | 1 | 16 | SAND | -0.27568 | 0.001952 |
| IACSP95-5000 24 hpi (m.i) | 1 | 17 | SAND | -0.27866 | -0.00077 |
| IACSP95-5000 24 hpi (m.i) | 1 | 18 | SAND | -0.27875 | -0.0006 |
| IACSP95-5000 24 hpi (m.i) | 1 | 19 | SAND | -0.27913 | -0.00072 |
| IACSP95-5000 24 hpi (m.i) | 1 | 20 | SAND | -0.27913 | -0.00046 |
| IACSP95-5000 24 hpi (m.i) | 1 | 21 | SAND | -0.2781 | 0.000822 |
| IACSP95-5000 24 hpi (m.i) | 1 | 22 | SAND | -0.28081 | -0.00163 |
| IACSP95-5000 24 hpi (m.i) | 1 | 23 | SAND | -0.27328 | 0.006162 |
| IACSP95-5000 24 hpi (m.i) | 1 | 24 | SAND | -0.26289 | 0.016809 |
| IACSP95-5000 24 hpi (m.i) | 1 | 25 | SAND | -0.2471 | 0.032863 |
| IACSP95-5000 24 hpi (m.i) | 1 | 26 | SAND | -0.20836 | 0.071856 |
| IACSP95-5000 24 hpi (m.i) | 1 | 27 | SAND | -0.13798 | 0.142498 |
| IACSP95-5000 24 hpi (m.i) | 1 | 28 | SAND | 0.008322 | 0.28906 |
| IACSP95-5000 24 hpi (m.i) | 1 | 29 | SAND | 0.276653 | 0.55765 |
| IACSP95-5000 24 hpi (m.i) | 1 | 30 | SAND | 0.756129 | 1.037385 |
| IACSP95-5000 24 hpi (m.i) | 1 | 31 | SAND | 1.519537 | 1.801052 |
| IACSP95-5000 24 hpi (m.i) | 1 | 32 | SAND | 2.47173 | 2.753505 |
| IACSP95-5000 24 hpi (m.i) | 1 | 33 | SAND | 3.352678 | 3.634711 |
| IACSP95-5000 24 hpi (m.i) | 1 | 34 | SAND | 4.125297 | 4.407589 |
| IACSP95-5000 24 hpi (m.i) | 1 | 35 | SAND | 4.801455 | 5.084007 |
| IACSP95-5000 24 hpi (m.i) | 1 | 36 | SAND | 5.34224 | 5.625051 |
| IACSP95-5000 24 hpi (m.i) | 1 | 37 | SAND | 5.749459 | 6.032528 |
| IACSP95-5000 24 hpi (m.i) | 1 | 38 | SAND | 6.061564 | 6.344893 |
| IACSP95-5000 24 hpi (m.i) | 1 | 39 | SAND | 6.319993 | 6.603581 |
| IACSP95-5000 24 hpi (m.i) | 1 | 40 | SAND | 6.49382 | 6.777667 |
| IACSP95-5000 24 hpi (m.i) | 2 | 1 | SAND | -0.25794 | -0.0057 |
| IACSP95-5000 24 hpi (m.i) | 2 | 2 | SAND | -0.2562 | -0.00354 |
| IACSP95-5000 24 hpi (m.i) | 2 | 3 | SAND | -0.25435 | -0.00128 |
| IACSP95-5000 24 hpi (m.i) | 2 | 4 | SAND | -0.25394 | -0.00045 |
| IACSP95-5000 24 hpi (m.i) | 2 | 5 | SAND | -0.25434 | -0.00044 |
| IACSP95-5000 24 hpi (m.i) | 2 | 6 | SAND | -0.25416 | 0.000144 |
| IACSP95-5000 24 hpi (m.i) | 2 | 7 | SAND | -0.25398 | 0.000734 |
| IACSP95-5000 24 hpi (m.i) | 2 | 8 | SAND | -0.25384 | 0.00129 |
| IACSP95-5000 24 hpi (m.i) | 2 | 9 | SAND | -0.25486 | 0.000678 |
| IACSP95-5000 24 hpi (m.i) | 2 | 10 | SAND | -0.25549 | 0.000469 |
| IACSP95-5000 24 hpi (m.i) | 2 | 11 | SAND | -0.2541 | 0.002263 |
| IACSP95-5000 24 hpi (m.i) | 2 | 12 | SAND | -0.25867 | -0.0019 |
| IACSP95-5000 24 hpi (m.i) | 2 | 13 | SAND | -0.25458 | 0.002609 |
| IACSP95-5000 24 hpi (m.i) | 2 | 14 | SAND | -0.25773 | -0.00013 |
| IACSP95-5000 24 hpi (m.i) | 2 | 15 | SAND | -0.25919 | -0.00118 |
| IACSP95-5000 24 hpi (m.i) | 2 | 16 | SAND | -0.2589 | -0.00048 |
| IACSP95-5000 24 hpi (m.i) | 2 | 17 | SAND | -0.26163 | -0.0028 |
| IACSP95-5000 24 hpi (m.i) | 2 | 18 | SAND | -0.25952 | -0.00028 |
| IACSP95-5000 24 hpi (m.i) | 2 | 19 | SAND | -0.26009 | -0.00043 |
| IACSP95-5000 24 hpi (m.i) | 2 | 20 | SAND | -0.26297 | -0.00289 |
| IACSP95-5000 24 hpi (m.i) | 2 | 21 | SAND | -0.26142 | -0.00094 |
| IACSP95-5000 24 hpi (m.i) | 2 | 22 | SAND | -0.25589 | 0.005007 |
| IACSP95-5000 24 hpi (m.i) | 2 | 23 | SAND | -0.25222 | 0.009091 |
| IACSP95-5000 24 hpi (m.i) | 2 | 24 | SAND | -0.24189 | 0.019831 |
| IACSP95-5000 24 hpi (m.i) | 2 | 25 | SAND | -0.22109 | 0.04104 |
| IACSP95-5000 24 hpi (m.i) | 2 | 26 | SAND | -0.18388 | 0.078657 |
| IACSP95-5000 24 hpi (m.i) | 2 | 27 | SAND | -0.10732 | 0.155628 |
| IACSP95-5000 24 hpi (m.i) | 2 | 28 | SAND | 0.043966 | 0.307331 |
| IACSP95-5000 24 hpi (m.i) | 2 | 29 | SAND | 0.322685 | 0.586462 |
| IACSP95-5000 24 hpi (m.i) | 2 | 30 | SAND | 0.81779 | 1.081979 |
| IACSP95-5000 24 hpi (m.i) | 2 | 31 | SAND | 1.61114 | 1.87574 |
| IACSP95-5000 24 hpi (m.i) | 2 | 32 | SAND | 2.604667 | 2.869679 |
| IACSP95-5000 24 hpi (m.i) | 2 | 33 | SAND | 3.527802 | 3.793226 |
| IACSP95-5000 24 hpi (m.i) | 2 | 34 | SAND | 4.338329 | 4.604164 |
| IACSP95-5000 24 hpi (m.i) | 2 | 35 | SAND | 5.004819 | 5.271066 |
| IACSP95-5000 24 hpi (m.i) | 2 | 36 | SAND | 5.527205 | 5.793863 |
| IACSP95-5000 24 hpi (m.i) | 2 | 37 | SAND | 5.913009 | 6.18008 |
| IACSP95-5000 24 hpi (m.i) | 2 | 38 | SAND | 6.275571 | 6.543053 |
| IACSP95-5000 24 hpi (m.i) | 2 | 39 | SAND | 6.443873 | 6.711767 |
| IACSP95-5000 24 hpi (m.i) | 2 | 40 | SAND | 6.627843 | 6.896149 |
| IACSP95-5000 24 hpi (m.i) | 3 | 1 | SAND | -0.29018 | -0.00892 |
| IACSP95-5000 24 hpi (m.i) | 3 | 2 | SAND | -0.28282 | -0.00139 |
| IACSP95-5000 24 hpi (m.i) | 3 | 3 | SAND | -0.28231 | -0.00071 |
| IACSP95-5000 24 hpi (m.i) | 3 | 4 | SAND | -0.28032 | 0.001444 |
| IACSP95-5000 24 hpi (m.i) | 3 | 5 | SAND | -0.28113 | 0.000796 |
| IACSP95-5000 24 hpi (m.i) | 3 | 6 | SAND | -0.28204 | 4.72E-05 |
| IACSP95-5000 24 hpi (m.i) | 3 | 7 | SAND | -0.2823 | -4.2E-05 |
| IACSP95-5000 24 hpi (m.i) | 3 | 8 | SAND | -0.28141 | 0.001018 |
| IACSP95-5000 24 hpi (m.i) | 3 | 9 | SAND | -0.28089 | 0.001704 |
| IACSP95-5000 24 hpi (m.i) | 3 | 10 | SAND | -0.28138 | 0.001373 |
| IACSP95-5000 24 hpi (m.i) | 3 | 11 | SAND | -0.28444 | -0.00151 |
| IACSP95-5000 24 hpi (m.i) | 3 | 12 | SAND | -0.28286 | 0.000231 |
| IACSP95-5000 24 hpi (m.i) | 3 | 13 | SAND | -0.28459 | -0.00134 |
| IACSP95-5000 24 hpi (m.i) | 3 | 14 | SAND | -0.28375 | -0.00033 |
| IACSP95-5000 24 hpi (m.i) | 3 | 15 | SAND | -0.28502 | -0.00143 |
| IACSP95-5000 24 hpi (m.i) | 3 | 16 | SAND | -0.28713 | -0.00338 |
| IACSP95-5000 24 hpi (m.i) | 3 | 17 | SAND | -0.28516 | -0.00124 |
| IACSP95-5000 24 hpi (m.i) | 3 | 18 | SAND | -0.28576 | -0.00168 |
| IACSP95-5000 24 hpi (m.i) | 3 | 19 | SAND | -0.28794 | -0.00369 |
| IACSP95-5000 24 hpi (m.i) | 3 | 20 | SAND | -0.28346 | 0.000952 |
| IACSP95-5000 24 hpi (m.i) | 3 | 21 | SAND | -0.28218 | 0.002399 |
| IACSP95-5000 24 hpi (m.i) | 3 | 22 | SAND | -0.27935 | 0.005391 |
| IACSP95-5000 24 hpi (m.i) | 3 | 23 | SAND | -0.27444 | 0.010467 |
| IACSP95-5000 24 hpi (m.i) | 3 | 24 | SAND | -0.26011 | 0.024965 |
| IACSP95-5000 24 hpi (m.i) | 3 | 25 | SAND | -0.23264 | 0.052602 |
| IACSP95-5000 24 hpi (m.i) | 3 | 26 | SAND | -0.17902 | 0.106387 |
| IACSP95-5000 24 hpi (m.i) | 3 | 27 | SAND | -0.07427 | 0.211303 |
| IACSP95-5000 24 hpi (m.i) | 3 | 28 | SAND | 0.12769 | 0.413431 |
| IACSP95-5000 24 hpi (m.i) | 3 | 29 | SAND | 0.495351 | 0.781258 |
| IACSP95-5000 24 hpi (m.i) | 3 | 30 | SAND | 1.138841 | 1.424914 |
| IACSP95-5000 24 hpi (m.i) | 3 | 31 | SAND | 2.095263 | 2.381502 |
| IACSP95-5000 24 hpi (m.i) | 3 | 32 | SAND | 3.13247 | 3.418875 |
| IACSP95-5000 24 hpi (m.i) | 3 | 33 | SAND | 4.029394 | 4.315965 |
| IACSP95-5000 24 hpi (m.i) | 3 | 34 | SAND | 4.775742 | 5.062478 |
| IACSP95-5000 24 hpi (m.i) | 3 | 35 | SAND | 5.397134 | 5.684037 |
| IACSP95-5000 24 hpi (m.i) | 3 | 36 | SAND | 5.841942 | 6.12901 |
| IACSP95-5000 24 hpi (m.i) | 3 | 37 | SAND | 6.179049 | 6.466283 |
| IACSP95-5000 24 hpi (m.i) | 3 | 38 | SAND | 6.435419 | 6.722819 |
| IACSP95-5000 24 hpi (m.i) | 3 | 39 | SAND | 6.624606 | 6.912171 |
| IACSP95-5000 24 hpi (m.i) | 3 | 40 | SAND | 6.748523 | 7.036254 |
| IACSP95-5000 24 hpi (s.i) | 1 | 1 | SAND | -0.22326 | -0.00366 |
| IACSP95-5000 24 hpi (s.i) | 1 | 2 | SAND | -0.22393 | -0.00407 |
| IACSP95-5000 24 hpi (s.i) | 1 | 3 | SAND | -0.22159 | -0.00147 |
| IACSP95-5000 24 hpi (s.i) | 1 | 4 | SAND | -0.22046 | -9.1E-05 |
| IACSP95-5000 24 hpi (s.i) | 1 | 5 | SAND | -0.22002 | 0.000603 |
| IACSP95-5000 24 hpi (s.i) | 1 | 6 | SAND | -0.21972 | 0.001158 |
| IACSP95-5000 24 hpi (s.i) | 1 | 7 | SAND | -0.21927 | 0.001856 |
| IACSP95-5000 24 hpi (s.i) | 1 | 8 | SAND | -0.21889 | 0.002495 |
| IACSP95-5000 24 hpi (s.i) | 1 | 9 | SAND | -0.22131 | 0.000328 |
| IACSP95-5000 24 hpi (s.i) | 1 | 10 | SAND | -0.22189 | -4.5E-07 |
| IACSP95-5000 24 hpi (s.i) | 1 | 11 | SAND | -0.22207 | 8.02E-05 |
| IACSP95-5000 24 hpi (s.i) | 1 | 12 | SAND | -0.22018 | 0.002224 |
| IACSP95-5000 24 hpi (s.i) | 1 | 13 | SAND | -0.22291 | -0.00025 |
| IACSP95-5000 24 hpi (s.i) | 1 | 14 | SAND | -0.22439 | -0.00148 |
| IACSP95-5000 24 hpi (s.i) | 1 | 15 | SAND | -0.22639 | -0.00322 |
| IACSP95-5000 24 hpi (s.i) | 1 | 16 | SAND | -0.22502 | -0.00159 |
| IACSP95-5000 24 hpi (s.i) | 1 | 17 | SAND | -0.22658 | -0.00291 |
| IACSP95-5000 24 hpi (s.i) | 1 | 18 | SAND | -0.22512 | -0.00119 |
| IACSP95-5000 24 hpi (s.i) | 1 | 19 | SAND | -0.22811 | -0.00392 |
| IACSP95-5000 24 hpi (s.i) | 1 | 20 | SAND | -0.22596 | -0.00152 |
| IACSP95-5000 24 hpi (s.i) | 1 | 21 | SAND | -0.22611 | -0.00142 |
| IACSP95-5000 24 hpi (s.i) | 1 | 22 | SAND | -0.2222 | 0.002746 |
| IACSP95-5000 24 hpi (s.i) | 1 | 23 | SAND | -0.21763 | 0.007577 |
| IACSP95-5000 24 hpi (s.i) | 1 | 24 | SAND | -0.20825 | 0.017209 |
| IACSP95-5000 24 hpi (s.i) | 1 | 25 | SAND | -0.1913 | 0.034414 |
| IACSP95-5000 24 hpi (s.i) | 1 | 26 | SAND | -0.15774 | 0.068229 |
| IACSP95-5000 24 hpi (s.i) | 1 | 27 | SAND | -0.09315 | 0.133072 |
| IACSP95-5000 24 hpi (s.i) | 1 | 28 | SAND | 0.038797 | 0.265277 |
| IACSP95-5000 24 hpi (s.i) | 1 | 29 | SAND | 0.279938 | 0.506672 |
| IACSP95-5000 24 hpi (s.i) | 1 | 30 | SAND | 0.726325 | 0.953313 |
| IACSP95-5000 24 hpi (s.i) | 1 | 31 | SAND | 1.466694 | 1.693937 |
| IACSP95-5000 24 hpi (s.i) | 1 | 32 | SAND | 2.480875 | 2.708373 |
| IACSP95-5000 24 hpi (s.i) | 1 | 33 | SAND | 3.492981 | 3.720734 |
| IACSP95-5000 24 hpi (s.i) | 1 | 34 | SAND | 4.345482 | 4.573489 |
| IACSP95-5000 24 hpi (s.i) | 1 | 35 | SAND | 5.063959 | 5.292221 |
| IACSP95-5000 24 hpi (s.i) | 1 | 36 | SAND | 5.61549 | 5.844007 |
| IACSP95-5000 24 hpi (s.i) | 1 | 37 | SAND | 6.039059 | 6.267831 |
| IACSP95-5000 24 hpi (s.i) | 1 | 38 | SAND | 6.392772 | 6.621798 |
| IACSP95-5000 24 hpi (s.i) | 1 | 39 | SAND | 6.611845 | 6.841126 |
| IACSP95-5000 24 hpi (s.i) | 1 | 40 | SAND | 6.801118 | 7.030654 |
| IACSP95-5000 24 hpi (s.i) | 2 | 1 | SAND | -0.26791 | -0.01397 |
| IACSP95-5000 24 hpi (s.i) | 2 | 2 | SAND | -0.26117 | -0.00762 |
| IACSP95-5000 24 hpi (s.i) | 2 | 3 | SAND | -0.255 | -0.00185 |
| IACSP95-5000 24 hpi (s.i) | 2 | 4 | SAND | -0.25177 | 0.00099 |
| IACSP95-5000 24 hpi (s.i) | 2 | 5 | SAND | -0.25083 | 0.001534 |
| IACSP95-5000 24 hpi (s.i) | 2 | 6 | SAND | -0.25083 | 0.001138 |
| IACSP95-5000 24 hpi (s.i) | 2 | 7 | SAND | -0.25215 | -0.00058 |
| IACSP95-5000 24 hpi (s.i) | 2 | 8 | SAND | -0.24946 | 0.001712 |
| IACSP95-5000 24 hpi (s.i) | 2 | 9 | SAND | -0.25065 | 0.000134 |
| IACSP95-5000 24 hpi (s.i) | 2 | 10 | SAND | -0.2507 | -0.00031 |
| IACSP95-5000 24 hpi (s.i) | 2 | 11 | SAND | -0.24946 | 0.000529 |
| IACSP95-5000 24 hpi (s.i) | 2 | 12 | SAND | -0.24813 | 0.001466 |
| IACSP95-5000 24 hpi (s.i) | 2 | 13 | SAND | -0.24932 | -0.00012 |
| IACSP95-5000 24 hpi (s.i) | 2 | 14 | SAND | -0.25076 | -0.00195 |
| IACSP95-5000 24 hpi (s.i) | 2 | 15 | SAND | -0.25068 | -0.00227 |
| IACSP95-5000 24 hpi (s.i) | 2 | 16 | SAND | -0.25048 | -0.00246 |
| IACSP95-5000 24 hpi (s.i) | 2 | 17 | SAND | -0.24976 | -0.00214 |
| IACSP95-5000 24 hpi (s.i) | 2 | 18 | SAND | -0.24642 | 0.000798 |
| IACSP95-5000 24 hpi (s.i) | 2 | 19 | SAND | -0.2485 | -0.00167 |
| IACSP95-5000 24 hpi (s.i) | 2 | 20 | SAND | -0.24754 | -0.00111 |
| IACSP95-5000 24 hpi (s.i) | 2 | 21 | SAND | -0.24458 | 0.001452 |
| IACSP95-5000 24 hpi (s.i) | 2 | 22 | SAND | -0.24092 | 0.004723 |
| IACSP95-5000 24 hpi (s.i) | 2 | 23 | SAND | -0.23606 | 0.009186 |
| IACSP95-5000 24 hpi (s.i) | 2 | 24 | SAND | -0.22488 | 0.019973 |
| IACSP95-5000 24 hpi (s.i) | 2 | 25 | SAND | -0.20765 | 0.0368 |
| IACSP95-5000 24 hpi (s.i) | 2 | 26 | SAND | -0.16995 | 0.074104 |
| IACSP95-5000 24 hpi (s.i) | 2 | 27 | SAND | -0.09966 | 0.144005 |
| IACSP95-5000 24 hpi (s.i) | 2 | 28 | SAND | 0.037686 | 0.280953 |
| IACSP95-5000 24 hpi (s.i) | 2 | 29 | SAND | 0.29897 | 0.541842 |
| IACSP95-5000 24 hpi (s.i) | 2 | 30 | SAND | 0.771681 | 1.014158 |
| IACSP95-5000 24 hpi (s.i) | 2 | 31 | SAND | 1.561377 | 1.803458 |
| IACSP95-5000 24 hpi (s.i) | 2 | 32 | SAND | 2.656913 | 2.898599 |
| IACSP95-5000 24 hpi (s.i) | 2 | 33 | SAND | 3.755789 | 3.997079 |
| IACSP95-5000 24 hpi (s.i) | 2 | 34 | SAND | 4.664278 | 4.905172 |
| IACSP95-5000 24 hpi (s.i) | 2 | 35 | SAND | 5.417605 | 5.658104 |
| IACSP95-5000 24 hpi (s.i) | 2 | 36 | SAND | 5.982959 | 6.223063 |
| IACSP95-5000 24 hpi (s.i) | 2 | 37 | SAND | 6.405487 | 6.645195 |
| IACSP95-5000 24 hpi (s.i) | 2 | 38 | SAND | 6.753746 | 6.993059 |
| IACSP95-5000 24 hpi (s.i) | 2 | 39 | SAND | 6.959723 | 7.198641 |
| IACSP95-5000 24 hpi (s.i) | 2 | 40 | SAND | 7.127249 | 7.365771 |
| IACSP95-5000 24 hpi (s.i) | 3 | 1 | SAND | -0.26312 | -0.00349 |
| IACSP95-5000 24 hpi (s.i) | 3 | 2 | SAND | -0.26139 | -0.0016 |
| IACSP95-5000 24 hpi (s.i) | 3 | 3 | SAND | -0.26001 | -6.9E-05 |
| IACSP95-5000 24 hpi (s.i) | 3 | 4 | SAND | -0.25869 | 0.001407 |
| IACSP95-5000 24 hpi (s.i) | 3 | 5 | SAND | -0.25984 | 0.000421 |
| IACSP95-5000 24 hpi (s.i) | 3 | 6 | SAND | -0.25943 | 0.000984 |
| IACSP95-5000 24 hpi (s.i) | 3 | 7 | SAND | -0.26025 | 0.000321 |
| IACSP95-5000 24 hpi (s.i) | 3 | 8 | SAND | -0.2592 | 0.001532 |
| IACSP95-5000 24 hpi (s.i) | 3 | 9 | SAND | -0.26043 | 0.000456 |
| IACSP95-5000 24 hpi (s.i) | 3 | 10 | SAND | -0.25979 | 0.001251 |
| IACSP95-5000 24 hpi (s.i) | 3 | 11 | SAND | -0.2605 | 0.000699 |
| IACSP95-5000 24 hpi (s.i) | 3 | 12 | SAND | -0.26304 | -0.00169 |
| IACSP95-5000 24 hpi (s.i) | 3 | 13 | SAND | -0.26279 | -0.00128 |
| IACSP95-5000 24 hpi (s.i) | 3 | 14 | SAND | -0.26353 | -0.00186 |
| IACSP95-5000 24 hpi (s.i) | 3 | 15 | SAND | -0.26397 | -0.00215 |
| IACSP95-5000 24 hpi (s.i) | 3 | 16 | SAND | -0.26437 | -0.00239 |
| IACSP95-5000 24 hpi (s.i) | 3 | 17 | SAND | -0.26245 | -0.00031 |
| IACSP95-5000 24 hpi (s.i) | 3 | 18 | SAND | -0.26371 | -0.00142 |
| IACSP95-5000 24 hpi (s.i) | 3 | 19 | SAND | -0.26622 | -0.00377 |
| IACSP95-5000 24 hpi (s.i) | 3 | 20 | SAND | -0.26491 | -0.00231 |
| IACSP95-5000 24 hpi (s.i) | 3 | 21 | SAND | -0.26251 | 0.000257 |
| IACSP95-5000 24 hpi (s.i) | 3 | 22 | SAND | -0.25932 | 0.003598 |
| IACSP95-5000 24 hpi (s.i) | 3 | 23 | SAND | -0.25677 | 0.006306 |
| IACSP95-5000 24 hpi (s.i) | 3 | 24 | SAND | -0.24681 | 0.016426 |
| IACSP95-5000 24 hpi (s.i) | 3 | 25 | SAND | -0.23014 | 0.033246 |
| IACSP95-5000 24 hpi (s.i) | 3 | 26 | SAND | -0.19947 | 0.064075 |
| IACSP95-5000 24 hpi (s.i) | 3 | 27 | SAND | -0.13474 | 0.128969 |
| IACSP95-5000 24 hpi (s.i) | 3 | 28 | SAND | -0.0078 | 0.256063 |
| IACSP95-5000 24 hpi (s.i) | 3 | 29 | SAND | 0.22881 | 0.492827 |
| IACSP95-5000 24 hpi (s.i) | 3 | 30 | SAND | 0.65712 | 0.921294 |
| IACSP95-5000 24 hpi (s.i) | 3 | 31 | SAND | 1.362111 | 1.626442 |
| IACSP95-5000 24 hpi (s.i) | 3 | 32 | SAND | 2.313204 | 2.577691 |
| IACSP95-5000 24 hpi (s.i) | 3 | 33 | SAND | 3.25223 | 3.516874 |
| IACSP95-5000 24 hpi (s.i) | 3 | 34 | SAND | 4.039205 | 4.304006 |
| IACSP95-5000 24 hpi (s.i) | 3 | 35 | SAND | 4.714571 | 4.979529 |
| IACSP95-5000 24 hpi (s.i) | 3 | 36 | SAND | 5.218962 | 5.484076 |
| IACSP95-5000 24 hpi (s.i) | 3 | 37 | SAND | 5.608695 | 5.873966 |
| IACSP95-5000 24 hpi (s.i) | 3 | 38 | SAND | 5.938821 | 6.204248 |
| IACSP95-5000 24 hpi (s.i) | 3 | 39 | SAND | 6.136969 | 6.402554 |
| IACSP95-5000 24 hpi (s.i) | 3 | 40 | SAND | 6.307718 | 6.573459 |
| IACSP95-5000 72 hpi (m.i) | 1 | 1 | SAND | -0.27887 | -0.00884 |
| IACSP95-5000 72 hpi (m.i) | 1 | 2 | SAND | -0.27501 | -0.00436 |
| IACSP95-5000 72 hpi (m.i) | 1 | 3 | SAND | -0.27364 | -0.00236 |
| IACSP95-5000 72 hpi (m.i) | 1 | 4 | SAND | -0.27249 | -0.0006 |
| IACSP95-5000 72 hpi (m.i) | 1 | 5 | SAND | -0.27267 | -0.00015 |
| IACSP95-5000 72 hpi (m.i) | 1 | 6 | SAND | -0.27126 | 0.001878 |
| IACSP95-5000 72 hpi (m.i) | 1 | 7 | SAND | -0.27292 | 0.000844 |
| IACSP95-5000 72 hpi (m.i) | 1 | 8 | SAND | -0.27222 | 0.002169 |
| IACSP95-5000 72 hpi (m.i) | 1 | 9 | SAND | -0.27524 | -0.00023 |
| IACSP95-5000 72 hpi (m.i) | 1 | 10 | SAND | -0.27422 | 0.001409 |
| IACSP95-5000 72 hpi (m.i) | 1 | 11 | SAND | -0.27426 | 0.001995 |
| IACSP95-5000 72 hpi (m.i) | 1 | 12 | SAND | -0.2759 | 0.000977 |
| IACSP95-5000 72 hpi (m.i) | 1 | 13 | SAND | -0.27881 | -0.00131 |
| IACSP95-5000 72 hpi (m.i) | 1 | 14 | SAND | -0.27784 | 0.000284 |
| IACSP95-5000 72 hpi (m.i) | 1 | 15 | SAND | -0.28118 | -0.00243 |
| IACSP95-5000 72 hpi (m.i) | 1 | 16 | SAND | -0.28122 | -0.00185 |
| IACSP95-5000 72 hpi (m.i) | 1 | 17 | SAND | -0.28176 | -0.00176 |
| IACSP95-5000 72 hpi (m.i) | 1 | 18 | SAND | -0.28243 | -0.00182 |
| IACSP95-5000 72 hpi (m.i) | 1 | 19 | SAND | -0.2831 | -0.00186 |
| IACSP95-5000 72 hpi (m.i) | 1 | 20 | SAND | -0.28268 | -0.00082 |
| IACSP95-5000 72 hpi (m.i) | 1 | 21 | SAND | -0.28192 | 0.000568 |
| IACSP95-5000 72 hpi (m.i) | 1 | 22 | SAND | -0.27803 | 0.005074 |
| IACSP95-5000 72 hpi (m.i) | 1 | 23 | SAND | -0.2738 | 0.009933 |
| IACSP95-5000 72 hpi (m.i) | 1 | 24 | SAND | -0.26276 | 0.021593 |
| IACSP95-5000 72 hpi (m.i) | 1 | 25 | SAND | -0.24055 | 0.044431 |
| IACSP95-5000 72 hpi (m.i) | 1 | 26 | SAND | -0.19727 | 0.08833 |
| IACSP95-5000 72 hpi (m.i) | 1 | 27 | SAND | -0.11215 | 0.174067 |
| IACSP95-5000 72 hpi (m.i) | 1 | 28 | SAND | 0.060035 | 0.34688 |
| IACSP95-5000 72 hpi (m.i) | 1 | 29 | SAND | 0.373116 | 0.660584 |
| IACSP95-5000 72 hpi (m.i) | 1 | 30 | SAND | 0.932336 | 1.220427 |
| IACSP95-5000 72 hpi (m.i) | 1 | 31 | SAND | 1.794009 | 2.082723 |
| IACSP95-5000 72 hpi (m.i) | 1 | 32 | SAND | 2.813348 | 3.102684 |
| IACSP95-5000 72 hpi (m.i) | 1 | 33 | SAND | 3.724259 | 4.014219 |
| IACSP95-5000 72 hpi (m.i) | 1 | 34 | SAND | 4.480224 | 4.770806 |
| IACSP95-5000 72 hpi (m.i) | 1 | 35 | SAND | 5.115106 | 5.406311 |
| IACSP95-5000 72 hpi (m.i) | 1 | 36 | SAND | 5.600379 | 5.892208 |
| IACSP95-5000 72 hpi (m.i) | 1 | 37 | SAND | 5.971458 | 6.263909 |
| IACSP95-5000 72 hpi (m.i) | 1 | 38 | SAND | 6.29824 | 6.591314 |
| IACSP95-5000 72 hpi (m.i) | 1 | 39 | SAND | 6.502398 | 6.796095 |
| IACSP95-5000 72 hpi (m.i) | 1 | 40 | SAND | 6.661664 | 6.955984 |
| IACSP95-5000 72 hpi (m.i) | 2 | 1 | SAND | -0.26391 | -0.00299 |
| IACSP95-5000 72 hpi (m.i) | 2 | 2 | SAND | -0.26306 | -0.00188 |
| IACSP95-5000 72 hpi (m.i) | 2 | 3 | SAND | -0.26139 | 5.35E-05 |
| IACSP95-5000 72 hpi (m.i) | 2 | 4 | SAND | -0.26143 | 0.000282 |
| IACSP95-5000 72 hpi (m.i) | 2 | 5 | SAND | -0.26198 | -1.2E-05 |
| IACSP95-5000 72 hpi (m.i) | 2 | 6 | SAND | -0.26103 | 0.0012 |
| IACSP95-5000 72 hpi (m.i) | 2 | 7 | SAND | -0.26187 | 0.000618 |
| IACSP95-5000 72 hpi (m.i) | 2 | 8 | SAND | -0.26232 | 0.000435 |
| IACSP95-5000 72 hpi (m.i) | 2 | 9 | SAND | -0.26241 | 0.000604 |
| IACSP95-5000 72 hpi (m.i) | 2 | 10 | SAND | -0.26278 | 0.000489 |
| IACSP95-5000 72 hpi (m.i) | 2 | 11 | SAND | -0.2636 | -7.1E-05 |
| IACSP95-5000 72 hpi (m.i) | 2 | 12 | SAND | -0.26541 | -0.00162 |
| IACSP95-5000 72 hpi (m.i) | 2 | 13 | SAND | -0.2653 | -0.00124 |
| IACSP95-5000 72 hpi (m.i) | 2 | 14 | SAND | -0.26439 | -7.5E-05 |
| IACSP95-5000 72 hpi (m.i) | 2 | 15 | SAND | -0.26552 | -0.00094 |
| IACSP95-5000 72 hpi (m.i) | 2 | 16 | SAND | -0.26516 | -0.00033 |
| IACSP95-5000 72 hpi (m.i) | 2 | 17 | SAND | -0.26545 | -0.00035 |
| IACSP95-5000 72 hpi (m.i) | 2 | 18 | SAND | -0.26625 | -0.00089 |
| IACSP95-5000 72 hpi (m.i) | 2 | 19 | SAND | -0.26915 | -0.00354 |
| IACSP95-5000 72 hpi (m.i) | 2 | 20 | SAND | -0.26664 | -0.00076 |
| IACSP95-5000 72 hpi (m.i) | 2 | 21 | SAND | -0.26546 | 0.000678 |
| IACSP95-5000 72 hpi (m.i) | 2 | 22 | SAND | -0.26092 | 0.005479 |
| IACSP95-5000 72 hpi (m.i) | 2 | 23 | SAND | -0.25499 | 0.011671 |
| IACSP95-5000 72 hpi (m.i) | 2 | 24 | SAND | -0.24145 | 0.025473 |
| IACSP95-5000 72 hpi (m.i) | 2 | 25 | SAND | -0.21513 | 0.052052 |
| IACSP95-5000 72 hpi (m.i) | 2 | 26 | SAND | -0.16216 | 0.105285 |
| IACSP95-5000 72 hpi (m.i) | 2 | 27 | SAND | -0.06091 | 0.206794 |
| IACSP95-5000 72 hpi (m.i) | 2 | 28 | SAND | 0.136085 | 0.404047 |
| IACSP95-5000 72 hpi (m.i) | 2 | 29 | SAND | 0.495842 | 0.764065 |
| IACSP95-5000 72 hpi (m.i) | 2 | 30 | SAND | 1.117581 | 1.386065 |
| IACSP95-5000 72 hpi (m.i) | 2 | 31 | SAND | 2.055087 | 2.323831 |
| IACSP95-5000 72 hpi (m.i) | 2 | 32 | SAND | 3.098473 | 3.367478 |
| IACSP95-5000 72 hpi (m.i) | 2 | 33 | SAND | 4.007661 | 4.276927 |
| IACSP95-5000 72 hpi (m.i) | 2 | 34 | SAND | 4.766473 | 5.036 |
| IACSP95-5000 72 hpi (m.i) | 2 | 35 | SAND | 5.374512 | 5.644299 |
| IACSP95-5000 72 hpi (m.i) | 2 | 36 | SAND | 5.847472 | 6.117519 |
| IACSP95-5000 72 hpi (m.i) | 2 | 37 | SAND | 6.186025 | 6.456333 |
| IACSP95-5000 72 hpi (m.i) | 2 | 38 | SAND | 6.483352 | 6.753921 |
| IACSP95-5000 72 hpi (m.i) | 2 | 39 | SAND | 6.66649 | 6.937319 |
| IACSP95-5000 72 hpi (m.i) | 2 | 40 | SAND | 6.80015 | 7.07124 |
| IACSP95-5000 72 hpi (m.i) | 3 | 1 | SAND | -0.32874 | -0.00279 |
| IACSP95-5000 72 hpi (m.i) | 3 | 2 | SAND | -0.32731 | -0.00057 |
| IACSP95-5000 72 hpi (m.i) | 3 | 3 | SAND | -0.33009 | -0.00256 |
| IACSP95-5000 72 hpi (m.i) | 3 | 4 | SAND | -0.32918 | -0.00085 |
| IACSP95-5000 72 hpi (m.i) | 3 | 5 | SAND | -0.32844 | 0.000679 |
| IACSP95-5000 72 hpi (m.i) | 3 | 6 | SAND | -0.32755 | 0.00236 |
| IACSP95-5000 72 hpi (m.i) | 3 | 7 | SAND | -0.32821 | 0.002499 |
| IACSP95-5000 72 hpi (m.i) | 3 | 8 | SAND | -0.32893 | 0.002569 |
| IACSP95-5000 72 hpi (m.i) | 3 | 9 | SAND | -0.33202 | 0.000267 |
| IACSP95-5000 72 hpi (m.i) | 3 | 10 | SAND | -0.33305 | 3.21E-05 |
| IACSP95-5000 72 hpi (m.i) | 3 | 11 | SAND | -0.33356 | 0.000315 |
| IACSP95-5000 72 hpi (m.i) | 3 | 12 | SAND | -0.33458 | 9.11E-05 |
| IACSP95-5000 72 hpi (m.i) | 3 | 13 | SAND | -0.33688 | -0.00142 |
| IACSP95-5000 72 hpi (m.i) | 3 | 14 | SAND | -0.33706 | -0.00081 |
| IACSP95-5000 72 hpi (m.i) | 3 | 15 | SAND | -0.33904 | -0.00199 |
| IACSP95-5000 72 hpi (m.i) | 3 | 16 | SAND | -0.34006 | -0.00222 |
| IACSP95-5000 72 hpi (m.i) | 3 | 17 | SAND | -0.34024 | -0.0016 |
| IACSP95-5000 72 hpi (m.i) | 3 | 18 | SAND | -0.34084 | -0.00142 |
| IACSP95-5000 72 hpi (m.i) | 3 | 19 | SAND | -0.34298 | -0.00277 |
| IACSP95-5000 72 hpi (m.i) | 3 | 20 | SAND | -0.34085 | 0.000158 |
| IACSP95-5000 72 hpi (m.i) | 3 | 21 | SAND | -0.34059 | 0.001212 |
| IACSP95-5000 72 hpi (m.i) | 3 | 22 | SAND | -0.33714 | 0.005458 |
| IACSP95-5000 72 hpi (m.i) | 3 | 23 | SAND | -0.3299 | 0.013486 |
| IACSP95-5000 72 hpi (m.i) | 3 | 24 | SAND | -0.31685 | 0.027329 |
| IACSP95-5000 72 hpi (m.i) | 3 | 25 | SAND | -0.28838 | 0.056593 |
| IACSP95-5000 72 hpi (m.i) | 3 | 26 | SAND | -0.23524 | 0.110527 |
| IACSP95-5000 72 hpi (m.i) | 3 | 27 | SAND | -0.13051 | 0.216048 |
| IACSP95-5000 72 hpi (m.i) | 3 | 28 | SAND | 0.077286 | 0.424638 |
| IACSP95-5000 72 hpi (m.i) | 3 | 29 | SAND | 0.451194 | 0.799339 |
| IACSP95-5000 72 hpi (m.i) | 3 | 30 | SAND | 1.094809 | 1.443747 |
| IACSP95-5000 72 hpi (m.i) | 3 | 31 | SAND | 2.035671 | 2.385401 |
| IACSP95-5000 72 hpi (m.i) | 3 | 32 | SAND | 3.044116 | 3.394639 |
| IACSP95-5000 72 hpi (m.i) | 3 | 33 | SAND | 3.910361 | 4.261677 |
| IACSP95-5000 72 hpi (m.i) | 3 | 34 | SAND | 4.609482 | 4.961591 |
| IACSP95-5000 72 hpi (m.i) | 3 | 35 | SAND | 5.189045 | 5.541947 |
| IACSP95-5000 72 hpi (m.i) | 3 | 36 | SAND | 5.601752 | 5.955446 |
| IACSP95-5000 72 hpi (m.i) | 3 | 37 | SAND | 5.922689 | 6.277176 |
| IACSP95-5000 72 hpi (m.i) | 3 | 38 | SAND | 6.183904 | 6.539184 |
| IACSP95-5000 72 hpi (m.i) | 3 | 39 | SAND | 6.335488 | 6.691561 |
| IACSP95-5000 72 hpi (m.i) | 3 | 40 | SAND | 6.478568 | 6.835434 |
| IACSP95-5000 72 hpi (s.i) | 1 | 1 | SAND | -0.38241 | -0.01933 |
| IACSP95-5000 72 hpi (s.i) | 1 | 2 | SAND | -0.37375 | -0.01051 |
| IACSP95-5000 72 hpi (s.i) | 1 | 3 | SAND | -0.36705 | -0.00364 |
| IACSP95-5000 72 hpi (s.i) | 1 | 4 | SAND | -0.36357 | 7.34E-06 |
| IACSP95-5000 72 hpi (s.i) | 1 | 5 | SAND | -0.36491 | -0.00116 |
| IACSP95-5000 72 hpi (s.i) | 1 | 6 | SAND | -0.36148 | 0.002438 |
| IACSP95-5000 72 hpi (s.i) | 1 | 7 | SAND | -0.36195 | 0.002135 |
| IACSP95-5000 72 hpi (s.i) | 1 | 8 | SAND | -0.36163 | 0.002626 |
| IACSP95-5000 72 hpi (s.i) | 1 | 9 | SAND | -0.3615 | 0.002932 |
| IACSP95-5000 72 hpi (s.i) | 1 | 10 | SAND | -0.36497 | -0.00038 |
| IACSP95-5000 72 hpi (s.i) | 1 | 11 | SAND | -0.36355 | 0.001217 |
| IACSP95-5000 72 hpi (s.i) | 1 | 12 | SAND | -0.36589 | -0.00095 |
| IACSP95-5000 72 hpi (s.i) | 1 | 13 | SAND | -0.36452 | 0.000587 |
| IACSP95-5000 72 hpi (s.i) | 1 | 14 | SAND | -0.36652 | -0.00124 |
| IACSP95-5000 72 hpi (s.i) | 1 | 15 | SAND | -0.36754 | -0.0021 |
| IACSP95-5000 72 hpi (s.i) | 1 | 16 | SAND | -0.36758 | -0.00197 |
| IACSP95-5000 72 hpi (s.i) | 1 | 17 | SAND | -0.36741 | -0.00163 |
| IACSP95-5000 72 hpi (s.i) | 1 | 18 | SAND | -0.36621 | -0.00026 |
| IACSP95-5000 72 hpi (s.i) | 1 | 19 | SAND | -0.36894 | -0.00282 |
| IACSP95-5000 72 hpi (s.i) | 1 | 20 | SAND | -0.36745 | -0.00117 |
| IACSP95-5000 72 hpi (s.i) | 1 | 21 | SAND | -0.36681 | -0.00035 |
| IACSP95-5000 72 hpi (s.i) | 1 | 22 | SAND | -0.36593 | 0.0007 |
| IACSP95-5000 72 hpi (s.i) | 1 | 23 | SAND | -0.36176 | 0.005032 |
| IACSP95-5000 72 hpi (s.i) | 1 | 24 | SAND | -0.35203 | 0.014929 |
| IACSP95-5000 72 hpi (s.i) | 1 | 25 | SAND | -0.33683 | 0.030299 |
| IACSP95-5000 72 hpi (s.i) | 1 | 26 | SAND | -0.30011 | 0.06719 |
| IACSP95-5000 72 hpi (s.i) | 1 | 27 | SAND | -0.232 | 0.135471 |
| IACSP95-5000 72 hpi (s.i) | 1 | 28 | SAND | -0.09133 | 0.276313 |
| IACSP95-5000 72 hpi (s.i) | 1 | 29 | SAND | 0.165171 | 0.53298 |
| IACSP95-5000 72 hpi (s.i) | 1 | 30 | SAND | 0.63308 | 1.001058 |
| IACSP95-5000 72 hpi (s.i) | 1 | 31 | SAND | 1.398625 | 1.766773 |
| IACSP95-5000 72 hpi (s.i) | 1 | 32 | SAND | 2.428925 | 2.797241 |
| IACSP95-5000 72 hpi (s.i) | 1 | 33 | SAND | 3.415461 | 3.783946 |
| IACSP95-5000 72 hpi (s.i) | 1 | 34 | SAND | 4.232459 | 4.601114 |
| IACSP95-5000 72 hpi (s.i) | 1 | 35 | SAND | 4.901618 | 5.270441 |
| IACSP95-5000 72 hpi (s.i) | 1 | 36 | SAND | 5.416709 | 5.785702 |
| IACSP95-5000 72 hpi (s.i) | 1 | 37 | SAND | 5.802643 | 6.171804 |
| IACSP95-5000 72 hpi (s.i) | 1 | 38 | SAND | 6.132001 | 6.501332 |
| IACSP95-5000 72 hpi (s.i) | 1 | 39 | SAND | 6.334282 | 6.703782 |
| IACSP95-5000 72 hpi (s.i) | 1 | 40 | SAND | 6.505873 | 6.875542 |
| IACSP95-5000 72 hpi (s.i) | 2 | 1 | SAND | -0.37096 | -0.01744 |
| IACSP95-5000 72 hpi (s.i) | 2 | 2 | SAND | -0.36138 | -0.00733 |
| IACSP95-5000 72 hpi (s.i) | 2 | 3 | SAND | -0.35805 | -0.00347 |
| IACSP95-5000 72 hpi (s.i) | 2 | 4 | SAND | -0.35471 | 0.000398 |
| IACSP95-5000 72 hpi (s.i) | 2 | 5 | SAND | -0.35761 | -0.00198 |
| IACSP95-5000 72 hpi (s.i) | 2 | 6 | SAND | -0.35356 | 0.002602 |
| IACSP95-5000 72 hpi (s.i) | 2 | 7 | SAND | -0.35499 | 0.001699 |
| IACSP95-5000 72 hpi (s.i) | 2 | 8 | SAND | -0.35427 | 0.002944 |
| IACSP95-5000 72 hpi (s.i) | 2 | 9 | SAND | -0.35716 | 0.000584 |
| IACSP95-5000 72 hpi (s.i) | 2 | 10 | SAND | -0.35568 | 0.002595 |
| IACSP95-5000 72 hpi (s.i) | 2 | 11 | SAND | -0.35791 | 0.000892 |
| IACSP95-5000 72 hpi (s.i) | 2 | 12 | SAND | -0.36157 | -0.00224 |
| IACSP95-5000 72 hpi (s.i) | 2 | 13 | SAND | -0.35911 | 0.000744 |
| IACSP95-5000 72 hpi (s.i) | 2 | 14 | SAND | -0.36127 | -0.00088 |
| IACSP95-5000 72 hpi (s.i) | 2 | 15 | SAND | -0.362 | -0.00108 |
| IACSP95-5000 72 hpi (s.i) | 2 | 16 | SAND | -0.36529 | -0.00385 |
| IACSP95-5000 72 hpi (s.i) | 2 | 17 | SAND | -0.36225 | -0.00028 |
| IACSP95-5000 72 hpi (s.i) | 2 | 18 | SAND | -0.36335 | -0.00085 |
| IACSP95-5000 72 hpi (s.i) | 2 | 19 | SAND | -0.36499 | -0.00196 |
| IACSP95-5000 72 hpi (s.i) | 2 | 20 | SAND | -0.36469 | -0.00113 |
| IACSP95-5000 72 hpi (s.i) | 2 | 21 | SAND | -0.36218 | 0.001909 |
| IACSP95-5000 72 hpi (s.i) | 2 | 22 | SAND | -0.36126 | 0.00335 |
| IACSP95-5000 72 hpi (s.i) | 2 | 23 | SAND | -0.35578 | 0.009359 |
| IACSP95-5000 72 hpi (s.i) | 2 | 24 | SAND | -0.34139 | 0.024275 |
| IACSP95-5000 72 hpi (s.i) | 2 | 25 | SAND | -0.31812 | 0.048074 |
| IACSP95-5000 72 hpi (s.i) | 2 | 26 | SAND | -0.27479 | 0.091939 |
| IACSP95-5000 72 hpi (s.i) | 2 | 27 | SAND | -0.18833 | 0.178922 |
| IACSP95-5000 72 hpi (s.i) | 2 | 28 | SAND | -0.01256 | 0.355221 |
| IACSP95-5000 72 hpi (s.i) | 2 | 29 | SAND | 0.309742 | 0.678051 |
| IACSP95-5000 72 hpi (s.i) | 2 | 30 | SAND | 0.87595 | 1.244788 |
| IACSP95-5000 72 hpi (s.i) | 2 | 31 | SAND | 1.761243 | 2.130609 |
| IACSP95-5000 72 hpi (s.i) | 2 | 32 | SAND | 2.850271 | 3.220166 |
| IACSP95-5000 72 hpi (s.i) | 2 | 33 | SAND | 3.814761 | 4.185183 |
| IACSP95-5000 72 hpi (s.i) | 2 | 34 | SAND | 4.61112 | 4.98207 |
| IACSP95-5000 72 hpi (s.i) | 2 | 35 | SAND | 5.248648 | 5.620127 |
| IACSP95-5000 72 hpi (s.i) | 2 | 36 | SAND | 5.727099 | 6.099106 |
| IACSP95-5000 72 hpi (s.i) | 2 | 37 | SAND | 6.091022 | 6.463558 |
| IACSP95-5000 72 hpi (s.i) | 2 | 38 | SAND | 6.391072 | 6.764135 |
| IACSP95-5000 72 hpi (s.i) | 2 | 39 | SAND | 6.592683 | 6.966274 |
| IACSP95-5000 72 hpi (s.i) | 2 | 40 | SAND | 6.738431 | 7.112551 |
| IACSP95-5000 72 hpi (s.i) | 3 | 1 | SAND | -0.3124 | -0.01143 |
| IACSP95-5000 72 hpi (s.i) | 3 | 2 | SAND | -0.30685 | -0.00562 |
| IACSP95-5000 72 hpi (s.i) | 3 | 3 | SAND | -0.30399 | -0.00249 |
| IACSP95-5000 72 hpi (s.i) | 3 | 4 | SAND | -0.30283 | -0.00107 |
| IACSP95-5000 72 hpi (s.i) | 3 | 5 | SAND | -0.30185 | 0.000173 |
| IACSP95-5000 72 hpi (s.i) | 3 | 6 | SAND | -0.29992 | 0.00237 |
| IACSP95-5000 72 hpi (s.i) | 3 | 7 | SAND | -0.30169 | 0.000857 |
| IACSP95-5000 72 hpi (s.i) | 3 | 8 | SAND | -0.30141 | 0.001401 |
| IACSP95-5000 72 hpi (s.i) | 3 | 9 | SAND | -0.30214 | 0.00094 |
| IACSP95-5000 72 hpi (s.i) | 3 | 10 | SAND | -0.3012 | 0.002141 |
| IACSP95-5000 72 hpi (s.i) | 3 | 11 | SAND | -0.30306 | 0.000547 |
| IACSP95-5000 72 hpi (s.i) | 3 | 12 | SAND | -0.3037 | 0.000169 |
| IACSP95-5000 72 hpi (s.i) | 3 | 13 | SAND | -0.30471 | -0.00058 |
| IACSP95-5000 72 hpi (s.i) | 3 | 14 | SAND | -0.30454 | -0.00015 |
| IACSP95-5000 72 hpi (s.i) | 3 | 15 | SAND | -0.30615 | -0.0015 |
| IACSP95-5000 72 hpi (s.i) | 3 | 16 | SAND | -0.30746 | -0.00254 |
| IACSP95-5000 72 hpi (s.i) | 3 | 17 | SAND | -0.30654 | -0.00136 |
| IACSP95-5000 72 hpi (s.i) | 3 | 18 | SAND | -0.30616 | -0.00071 |
| IACSP95-5000 72 hpi (s.i) | 3 | 19 | SAND | -0.30823 | -0.00252 |
| IACSP95-5000 72 hpi (s.i) | 3 | 20 | SAND | -0.30818 | -0.0022 |
| IACSP95-5000 72 hpi (s.i) | 3 | 21 | SAND | -0.30481 | 0.001428 |
| IACSP95-5000 72 hpi (s.i) | 3 | 22 | SAND | -0.30141 | 0.005097 |
| IACSP95-5000 72 hpi (s.i) | 3 | 23 | SAND | -0.29723 | 0.009537 |
| IACSP95-5000 72 hpi (s.i) | 3 | 24 | SAND | -0.28547 | 0.021562 |
| IACSP95-5000 72 hpi (s.i) | 3 | 25 | SAND | -0.26295 | 0.044343 |
| IACSP95-5000 72 hpi (s.i) | 3 | 26 | SAND | -0.2185 | 0.089053 |
| IACSP95-5000 72 hpi (s.i) | 3 | 27 | SAND | -0.13414 | 0.173683 |
| IACSP95-5000 72 hpi (s.i) | 3 | 28 | SAND | 0.038828 | 0.346913 |
| IACSP95-5000 72 hpi (s.i) | 3 | 29 | SAND | 0.354582 | 0.662931 |
| IACSP95-5000 72 hpi (s.i) | 3 | 30 | SAND | 0.917414 | 1.226026 |
| IACSP95-5000 72 hpi (s.i) | 3 | 31 | SAND | 1.809715 | 2.118591 |
| IACSP95-5000 72 hpi (s.i) | 3 | 32 | SAND | 2.931896 | 3.241035 |
| IACSP95-5000 72 hpi (s.i) | 3 | 33 | SAND | 3.955224 | 4.264627 |
| IACSP95-5000 72 hpi (s.i) | 3 | 34 | SAND | 4.783091 | 5.092757 |
| IACSP95-5000 72 hpi (s.i) | 3 | 35 | SAND | 5.457943 | 5.767874 |
| IACSP95-5000 72 hpi (s.i) | 3 | 36 | SAND | 5.963825 | 6.274019 |
| IACSP95-5000 72 hpi (s.i) | 3 | 37 | SAND | 6.346471 | 6.656929 |
| IACSP95-5000 72 hpi (s.i) | 3 | 38 | SAND | 6.659596 | 6.970318 |
| IACSP95-5000 72 hpi (s.i) | 3 | 39 | SAND | 6.860445 | 7.171429 |
| IACSP95-5000 72 hpi (s.i) | 3 | 40 | SAND | 7.013254 | 7.324502 |
| IAC91-1099 24 hpi (m.i) | 1 | 1 | UBC18 | -0.16578 | 0.001057 |
| IAC91-1099 24 hpi (m.i) | 1 | 2 | UBC18 | -0.16972 | -0.00118 |
| IAC91-1099 24 hpi (m.i) | 1 | 3 | UBC18 | -0.17357 | -0.00333 |
| IAC91-1099 24 hpi (m.i) | 1 | 4 | UBC18 | -0.17608 | -0.00413 |
| IAC91-1099 24 hpi (m.i) | 1 | 5 | UBC18 | -0.17762 | -0.00396 |
| IAC91-1099 24 hpi (m.i) | 1 | 6 | UBC18 | -0.17576 | -0.0004 |
| IAC91-1099 24 hpi (m.i) | 1 | 7 | UBC18 | -0.17591 | 0.001158 |
| IAC91-1099 24 hpi (m.i) | 1 | 8 | UBC18 | -0.17742 | 0.001352 |
| IAC91-1099 24 hpi (m.i) | 1 | 9 | UBC18 | -0.17622 | 0.004257 |
| IAC91-1099 24 hpi (m.i) | 1 | 10 | UBC18 | -0.18005 | 0.002135 |
| IAC91-1099 24 hpi (m.i) | 1 | 11 | UBC18 | -0.17985 | 0.004034 |
| IAC91-1099 24 hpi (m.i) | 1 | 12 | UBC18 | -0.18343 | 0.002161 |
| IAC91-1099 24 hpi (m.i) | 1 | 13 | UBC18 | -0.18398 | 0.003322 |
| IAC91-1099 24 hpi (m.i) | 1 | 14 | UBC18 | -0.1847 | 0.004307 |
| IAC91-1099 24 hpi (m.i) | 1 | 15 | UBC18 | -0.18984 | 0.000869 |
| IAC91-1099 24 hpi (m.i) | 1 | 16 | UBC18 | -0.19233 | 8.73E-05 |
| IAC91-1099 24 hpi (m.i) | 1 | 17 | UBC18 | -0.19507 | -0.00095 |
| IAC91-1099 24 hpi (m.i) | 1 | 18 | UBC18 | -0.19774 | -0.00192 |
| IAC91-1099 24 hpi (m.i) | 1 | 19 | UBC18 | -0.20149 | -0.00396 |
| IAC91-1099 24 hpi (m.i) | 1 | 20 | UBC18 | -0.2028 | -0.00357 |
| IAC91-1099 24 hpi (m.i) | 1 | 21 | UBC18 | -0.2044 | -0.00346 |
| IAC91-1099 24 hpi (m.i) | 1 | 22 | UBC18 | -0.20432 | -0.00168 |
| IAC91-1099 24 hpi (m.i) | 1 | 23 | UBC18 | -0.20496 | -0.00061 |
| IAC91-1099 24 hpi (m.i) | 1 | 24 | UBC18 | -0.20177 | 0.00428 |
| IAC91-1099 24 hpi (m.i) | 1 | 25 | UBC18 | -0.19511 | 0.012646 |
| IAC91-1099 24 hpi (m.i) | 1 | 26 | UBC18 | -0.17943 | 0.03003 |
| IAC91-1099 24 hpi (m.i) | 1 | 27 | UBC18 | -0.14993 | 0.061244 |
| IAC91-1099 24 hpi (m.i) | 1 | 28 | UBC18 | -0.08162 | 0.13126 |
| IAC91-1099 24 hpi (m.i) | 1 | 29 | UBC18 | 0.038228 | 0.252809 |
| IAC91-1099 24 hpi (m.i) | 1 | 30 | UBC18 | 0.267695 | 0.483981 |
| IAC91-1099 24 hpi (m.i) | 1 | 31 | UBC18 | 0.646113 | 0.864104 |
| IAC91-1099 24 hpi (m.i) | 1 | 32 | UBC18 | 1.149291 | 1.368987 |
| IAC91-1099 24 hpi (m.i) | 1 | 33 | UBC18 | 1.709292 | 1.930694 |
| IAC91-1099 24 hpi (m.i) | 1 | 34 | UBC18 | 2.26281 | 2.485917 |
| IAC91-1099 24 hpi (m.i) | 1 | 35 | UBC18 | 2.783686 | 3.008498 |
| IAC91-1099 24 hpi (m.i) | 1 | 36 | UBC18 | 3.234317 | 3.460834 |
| IAC91-1099 24 hpi (m.i) | 1 | 37 | UBC18 | 3.620068 | 3.84829 |
| IAC91-1099 24 hpi (m.i) | 1 | 38 | UBC18 | 3.984559 | 4.214487 |
| IAC91-1099 24 hpi (m.i) | 1 | 39 | UBC18 | 4.245552 | 4.477185 |
| IAC91-1099 24 hpi (m.i) | 1 | 40 | UBC18 | 4.486349 | 4.719687 |
| IAC91-1099 24 hpi (m.i) | 2 | 1 | UBC18 | -0.09578 | 0.003379 |
| IAC91-1099 24 hpi (m.i) | 2 | 2 | UBC18 | -0.10166 | -0.00031 |
| IAC91-1099 24 hpi (m.i) | 2 | 3 | UBC18 | -0.1023 | 0.001239 |
| IAC91-1099 24 hpi (m.i) | 2 | 4 | UBC18 | -0.10321 | 0.002523 |
| IAC91-1099 24 hpi (m.i) | 2 | 5 | UBC18 | -0.10544 | 0.002482 |
| IAC91-1099 24 hpi (m.i) | 2 | 6 | UBC18 | -0.10934 | 0.00077 |
| IAC91-1099 24 hpi (m.i) | 2 | 7 | UBC18 | -0.10937 | 0.002929 |
| IAC91-1099 24 hpi (m.i) | 2 | 8 | UBC18 | -0.11435 | 0.000138 |
| IAC91-1099 24 hpi (m.i) | 2 | 9 | UBC18 | -0.11744 | -0.00077 |
| IAC91-1099 24 hpi (m.i) | 2 | 10 | UBC18 | -0.11768 | 0.001181 |
| IAC91-1099 24 hpi (m.i) | 2 | 11 | UBC18 | -0.12061 | 0.00044 |
| IAC91-1099 24 hpi (m.i) | 2 | 12 | UBC18 | -0.12586 | -0.00262 |
| IAC91-1099 24 hpi (m.i) | 2 | 13 | UBC18 | -0.12694 | -0.0015 |
| IAC91-1099 24 hpi (m.i) | 2 | 14 | UBC18 | -0.13139 | -0.00377 |
| IAC91-1099 24 hpi (m.i) | 2 | 15 | UBC18 | -0.13592 | -0.00611 |
| IAC91-1099 24 hpi (m.i) | 2 | 16 | UBC18 | -0.13834 | -0.00634 |
| IAC91-1099 24 hpi (m.i) | 2 | 17 | UBC18 | -0.13805 | -0.00387 |
| IAC91-1099 24 hpi (m.i) | 2 | 18 | UBC18 | -0.13653 | -0.00015 |
| IAC91-1099 24 hpi (m.i) | 2 | 19 | UBC18 | -0.1381 | 0.000465 |
| IAC91-1099 24 hpi (m.i) | 2 | 20 | UBC18 | -0.13992 | 0.000837 |
| IAC91-1099 24 hpi (m.i) | 2 | 21 | UBC18 | -0.14425 | -0.0013 |
| IAC91-1099 24 hpi (m.i) | 2 | 22 | UBC18 | -0.14119 | 0.003945 |
| IAC91-1099 24 hpi (m.i) | 2 | 23 | UBC18 | -0.13784 | 0.009483 |
| IAC91-1099 24 hpi (m.i) | 2 | 24 | UBC18 | -0.13239 | 0.017126 |
| IAC91-1099 24 hpi (m.i) | 2 | 25 | UBC18 | -0.1222 | 0.029504 |
| IAC91-1099 24 hpi (m.i) | 2 | 26 | UBC18 | -0.08943 | 0.06446 |
| IAC91-1099 24 hpi (m.i) | 2 | 27 | UBC18 | -0.03595 | 0.120134 |
| IAC91-1099 24 hpi (m.i) | 2 | 28 | UBC18 | 0.084621 | 0.24289 |
| IAC91-1099 24 hpi (m.i) | 2 | 29 | UBC18 | 0.294121 | 0.454579 |
| IAC91-1099 24 hpi (m.i) | 2 | 30 | UBC18 | 0.673423 | 0.836071 |
| IAC91-1099 24 hpi (m.i) | 2 | 31 | UBC18 | 1.249094 | 1.41393 |
| IAC91-1099 24 hpi (m.i) | 2 | 32 | UBC18 | 1.937031 | 2.104057 |
| IAC91-1099 24 hpi (m.i) | 2 | 33 | UBC18 | 2.634029 | 2.803244 |
| IAC91-1099 24 hpi (m.i) | 2 | 34 | UBC18 | 3.300573 | 3.471977 |
| IAC91-1099 24 hpi (m.i) | 2 | 35 | UBC18 | 3.89945 | 4.073043 |
| IAC91-1099 24 hpi (m.i) | 2 | 36 | UBC18 | 4.403104 | 4.578887 |
| IAC91-1099 24 hpi (m.i) | 2 | 37 | UBC18 | 4.813978 | 4.99195 |
| IAC91-1099 24 hpi (m.i) | 2 | 38 | UBC18 | 5.203048 | 5.383209 |
| IAC91-1099 24 hpi (m.i) | 2 | 39 | UBC18 | 5.486987 | 5.669337 |
| IAC91-1099 24 hpi (m.i) | 2 | 40 | UBC18 | 5.743414 | 5.927954 |
| IAC91-1099 24 hpi (m.i) | 3 | 1 | UBC18 | -0.0734 | 0.008999 |
| IAC91-1099 24 hpi (m.i) | 3 | 2 | UBC18 | -0.0808 | 0.003891 |
| IAC91-1099 24 hpi (m.i) | 3 | 3 | UBC18 | -0.08543 | 0.001548 |
| IAC91-1099 24 hpi (m.i) | 3 | 4 | UBC18 | -0.08908 | 0.000186 |
| IAC91-1099 24 hpi (m.i) | 3 | 5 | UBC18 | -0.09172 | -0.00015 |
| IAC91-1099 24 hpi (m.i) | 3 | 6 | UBC18 | -0.09217 | 0.00168 |
| IAC91-1099 24 hpi (m.i) | 3 | 7 | UBC18 | -0.09417 | 0.001973 |
| IAC91-1099 24 hpi (m.i) | 3 | 8 | UBC18 | -0.09839 | 3.89E-05 |
| IAC91-1099 24 hpi (m.i) | 3 | 9 | UBC18 | -0.10061 | 0.000111 |
| IAC91-1099 24 hpi (m.i) | 3 | 10 | UBC18 | -0.10679 | -0.00378 |
| IAC91-1099 24 hpi (m.i) | 3 | 11 | UBC18 | -0.1064 | -0.0011 |
| IAC91-1099 24 hpi (m.i) | 3 | 12 | UBC18 | -0.10887 | -0.00128 |
| IAC91-1099 24 hpi (m.i) | 3 | 13 | UBC18 | -0.1108 | -0.00092 |
| IAC91-1099 24 hpi (m.i) | 3 | 14 | UBC18 | -0.10975 | 0.002424 |
| IAC91-1099 24 hpi (m.i) | 3 | 15 | UBC18 | -0.1162 | -0.00174 |
| IAC91-1099 24 hpi (m.i) | 3 | 16 | UBC18 | -0.11495 | 0.001799 |
| IAC91-1099 24 hpi (m.i) | 3 | 17 | UBC18 | -0.12045 | -0.00141 |
| IAC91-1099 24 hpi (m.i) | 3 | 18 | UBC18 | -0.12215 | -0.00082 |
| IAC91-1099 24 hpi (m.i) | 3 | 19 | UBC18 | -0.1258 | -0.00218 |
| IAC91-1099 24 hpi (m.i) | 3 | 20 | UBC18 | -0.12734 | -0.00143 |
| IAC91-1099 24 hpi (m.i) | 3 | 21 | UBC18 | -0.13014 | -0.00194 |
| IAC91-1099 24 hpi (m.i) | 3 | 22 | UBC18 | -0.12993 | 0.000567 |
| IAC91-1099 24 hpi (m.i) | 3 | 23 | UBC18 | -0.12636 | 0.006424 |
| IAC91-1099 24 hpi (m.i) | 3 | 24 | UBC18 | -0.12098 | 0.014089 |
| IAC91-1099 24 hpi (m.i) | 3 | 25 | UBC18 | -0.10904 | 0.02832 |
| IAC91-1099 24 hpi (m.i) | 3 | 26 | UBC18 | -0.08355 | 0.056105 |
| IAC91-1099 24 hpi (m.i) | 3 | 27 | UBC18 | -0.03143 | 0.110512 |
| IAC91-1099 24 hpi (m.i) | 3 | 28 | UBC18 | 0.079507 | 0.223739 |
| IAC91-1099 24 hpi (m.i) | 3 | 29 | UBC18 | 0.276144 | 0.422666 |
| IAC91-1099 24 hpi (m.i) | 3 | 30 | UBC18 | 0.628992 | 0.777805 |
| IAC91-1099 24 hpi (m.i) | 3 | 31 | UBC18 | 1.169804 | 1.320907 |
| IAC91-1099 24 hpi (m.i) | 3 | 32 | UBC18 | 1.815884 | 1.969278 |
| IAC91-1099 24 hpi (m.i) | 3 | 33 | UBC18 | 2.473049 | 2.628732 |
| IAC91-1099 24 hpi (m.i) | 3 | 34 | UBC18 | 3.080104 | 3.238078 |
| IAC91-1099 24 hpi (m.i) | 3 | 35 | UBC18 | 3.628773 | 3.789037 |
| IAC91-1099 24 hpi (m.i) | 3 | 36 | UBC18 | 4.087606 | 4.25016 |
| IAC91-1099 24 hpi (m.i) | 3 | 37 | UBC18 | 4.459916 | 4.62476 |
| IAC91-1099 24 hpi (m.i) | 3 | 38 | UBC18 | 4.802756 | 4.96989 |
| IAC91-1099 24 hpi (m.i) | 3 | 39 | UBC18 | 5.054236 | 5.22366 |
| IAC91-1099 24 hpi (m.i) | 3 | 40 | UBC18 | 5.266858 | 5.438572 |
| IAC91-1099 24 hpi (s.i) | 1 | 1 | UBC18 | -0.27335 | -0.01337 |
| IAC91-1099 24 hpi (s.i) | 1 | 2 | UBC18 | -0.27122 | -0.01101 |
| IAC91-1099 24 hpi (s.i) | 1 | 3 | UBC18 | -0.26545 | -0.00502 |
| IAC91-1099 24 hpi (s.i) | 1 | 4 | UBC18 | -0.26273 | -0.00207 |
| IAC91-1099 24 hpi (s.i) | 1 | 5 | UBC18 | -0.25975 | 0.001125 |
| IAC91-1099 24 hpi (s.i) | 1 | 6 | UBC18 | -0.25929 | 0.001816 |
| IAC91-1099 24 hpi (s.i) | 1 | 7 | UBC18 | -0.25912 | 0.002203 |
| IAC91-1099 24 hpi (s.i) | 1 | 8 | UBC18 | -0.25951 | 0.002035 |
| IAC91-1099 24 hpi (s.i) | 1 | 9 | UBC18 | -0.25891 | 0.002862 |
| IAC91-1099 24 hpi (s.i) | 1 | 10 | UBC18 | -0.26061 | 0.001383 |
| IAC91-1099 24 hpi (s.i) | 1 | 11 | UBC18 | -0.26042 | 0.001802 |
| IAC91-1099 24 hpi (s.i) | 1 | 12 | UBC18 | -0.26128 | 0.001159 |
| IAC91-1099 24 hpi (s.i) | 1 | 13 | UBC18 | -0.26171 | 0.000957 |
| IAC91-1099 24 hpi (s.i) | 1 | 14 | UBC18 | -0.26189 | 0.001002 |
| IAC91-1099 24 hpi (s.i) | 1 | 15 | UBC18 | -0.26536 | -0.00224 |
| IAC91-1099 24 hpi (s.i) | 1 | 16 | UBC18 | -0.26577 | -0.00244 |
| IAC91-1099 24 hpi (s.i) | 1 | 17 | UBC18 | -0.26656 | -0.003 |
| IAC91-1099 24 hpi (s.i) | 1 | 18 | UBC18 | -0.26638 | -0.0026 |
| IAC91-1099 24 hpi (s.i) | 1 | 19 | UBC18 | -0.26763 | -0.00362 |
| IAC91-1099 24 hpi (s.i) | 1 | 20 | UBC18 | -0.26629 | -0.00207 |
| IAC91-1099 24 hpi (s.i) | 1 | 21 | UBC18 | -0.26607 | -0.00162 |
| IAC91-1099 24 hpi (s.i) | 1 | 22 | UBC18 | -0.26278 | 0.001898 |
| IAC91-1099 24 hpi (s.i) | 1 | 23 | UBC18 | -0.25846 | 0.006442 |
| IAC91-1099 24 hpi (s.i) | 1 | 24 | UBC18 | -0.25052 | 0.014603 |
| IAC91-1099 24 hpi (s.i) | 1 | 25 | UBC18 | -0.23298 | 0.032365 |
| IAC91-1099 24 hpi (s.i) | 1 | 26 | UBC18 | -0.20023 | 0.065334 |
| IAC91-1099 24 hpi (s.i) | 1 | 27 | UBC18 | -0.13485 | 0.130942 |
| IAC91-1099 24 hpi (s.i) | 1 | 28 | UBC18 | -0.00115 | 0.264864 |
| IAC91-1099 24 hpi (s.i) | 1 | 29 | UBC18 | 0.236214 | 0.502451 |
| IAC91-1099 24 hpi (s.i) | 1 | 30 | UBC18 | 0.657612 | 0.924073 |
| IAC91-1099 24 hpi (s.i) | 1 | 31 | UBC18 | 1.298105 | 1.564789 |
| IAC91-1099 24 hpi (s.i) | 1 | 32 | UBC18 | 2.055937 | 2.322845 |
| IAC91-1099 24 hpi (s.i) | 1 | 33 | UBC18 | 2.81157 | 3.078701 |
| IAC91-1099 24 hpi (s.i) | 1 | 34 | UBC18 | 3.499307 | 3.766661 |
| IAC91-1099 24 hpi (s.i) | 1 | 35 | UBC18 | 4.110293 | 4.377871 |
| IAC91-1099 24 hpi (s.i) | 1 | 36 | UBC18 | 4.611528 | 4.879329 |
| IAC91-1099 24 hpi (s.i) | 1 | 37 | UBC18 | 5.024688 | 5.292712 |
| IAC91-1099 24 hpi (s.i) | 1 | 38 | UBC18 | 5.392951 | 5.661198 |
| IAC91-1099 24 hpi (s.i) | 1 | 39 | UBC18 | 5.652915 | 5.921386 |
| IAC91-1099 24 hpi (s.i) | 1 | 40 | UBC18 | 5.904838 | 6.173532 |
| IAC91-1099 24 hpi (s.i) | 2 | 1 | UBC18 | -0.28785 | -0.01103 |
| IAC91-1099 24 hpi (s.i) | 2 | 2 | UBC18 | -0.28342 | -0.00584 |
| IAC91-1099 24 hpi (s.i) | 2 | 3 | UBC18 | -0.28076 | -0.00242 |
| IAC91-1099 24 hpi (s.i) | 2 | 4 | UBC18 | -0.27987 | -0.00077 |
| IAC91-1099 24 hpi (s.i) | 2 | 5 | UBC18 | -0.28019 | -0.00031 |
| IAC91-1099 24 hpi (s.i) | 2 | 6 | UBC18 | -0.2781 | 0.002539 |
| IAC91-1099 24 hpi (s.i) | 2 | 7 | UBC18 | -0.28156 | -0.00015 |
| IAC91-1099 24 hpi (s.i) | 2 | 8 | UBC18 | -0.2817 | 0.000464 |
| IAC91-1099 24 hpi (s.i) | 2 | 9 | UBC18 | -0.28123 | 0.001705 |
| IAC91-1099 24 hpi (s.i) | 2 | 10 | UBC18 | -0.28312 | 0.000572 |
| IAC91-1099 24 hpi (s.i) | 2 | 11 | UBC18 | -0.28461 | -0.00015 |
| IAC91-1099 24 hpi (s.i) | 2 | 12 | UBC18 | -0.28618 | -0.00095 |
| IAC91-1099 24 hpi (s.i) | 2 | 13 | UBC18 | -0.28651 | -0.00053 |
| IAC91-1099 24 hpi (s.i) | 2 | 14 | UBC18 | -0.28563 | 0.001126 |
| IAC91-1099 24 hpi (s.i) | 2 | 15 | UBC18 | -0.28866 | -0.00114 |
| IAC91-1099 24 hpi (s.i) | 2 | 16 | UBC18 | -0.28814 | 0.000144 |
| IAC91-1099 24 hpi (s.i) | 2 | 17 | UBC18 | -0.28826 | 0.000786 |
| IAC91-1099 24 hpi (s.i) | 2 | 18 | UBC18 | -0.28862 | 0.001196 |
| IAC91-1099 24 hpi (s.i) | 2 | 19 | UBC18 | -0.29104 | -0.00046 |
| IAC91-1099 24 hpi (s.i) | 2 | 20 | UBC18 | -0.29173 | -0.00039 |
| IAC91-1099 24 hpi (s.i) | 2 | 21 | UBC18 | -0.29333 | -0.00123 |
| IAC91-1099 24 hpi (s.i) | 2 | 22 | UBC18 | -0.29306 | -0.00019 |
| IAC91-1099 24 hpi (s.i) | 2 | 23 | UBC18 | -0.29348 | 0.000154 |
| IAC91-1099 24 hpi (s.i) | 2 | 24 | UBC18 | -0.28805 | 0.006346 |
| IAC91-1099 24 hpi (s.i) | 2 | 25 | UBC18 | -0.27721 | 0.017956 |
| IAC91-1099 24 hpi (s.i) | 2 | 26 | UBC18 | -0.25296 | 0.042965 |
| IAC91-1099 24 hpi (s.i) | 2 | 27 | UBC18 | -0.20716 | 0.08953 |
| IAC91-1099 24 hpi (s.i) | 2 | 28 | UBC18 | -0.10977 | 0.187688 |
| IAC91-1099 24 hpi (s.i) | 2 | 29 | UBC18 | 0.06684 | 0.365061 |
| IAC91-1099 24 hpi (s.i) | 2 | 30 | UBC18 | 0.384508 | 0.683493 |
| IAC91-1099 24 hpi (s.i) | 2 | 31 | UBC18 | 0.881319 | 1.181068 |
| IAC91-1099 24 hpi (s.i) | 2 | 32 | UBC18 | 1.490239 | 1.790753 |
| IAC91-1099 24 hpi (s.i) | 2 | 33 | UBC18 | 2.121341 | 2.422619 |
| IAC91-1099 24 hpi (s.i) | 2 | 34 | UBC18 | 2.717148 | 3.019191 |
| IAC91-1099 24 hpi (s.i) | 2 | 35 | UBC18 | 3.265883 | 3.56869 |
| IAC91-1099 24 hpi (s.i) | 2 | 36 | UBC18 | 3.726152 | 4.029724 |
| IAC91-1099 24 hpi (s.i) | 2 | 37 | UBC18 | 4.113328 | 4.417665 |
| IAC91-1099 24 hpi (s.i) | 2 | 38 | UBC18 | 4.469856 | 4.774956 |
| IAC91-1099 24 hpi (s.i) | 2 | 39 | UBC18 | 4.734125 | 5.03999 |
| IAC91-1099 24 hpi (s.i) | 2 | 40 | UBC18 | 4.965105 | 5.271735 |
| IAC91-1099 24 hpi (s.i) | 3 | 1 | UBC18 | -0.27102 | -0.01438 |
| IAC91-1099 24 hpi (s.i) | 3 | 2 | UBC18 | -0.26525 | -0.00803 |
| IAC91-1099 24 hpi (s.i) | 3 | 3 | UBC18 | -0.26092 | -0.00314 |
| IAC91-1099 24 hpi (s.i) | 3 | 4 | UBC18 | -0.26011 | -0.00177 |
| IAC91-1099 24 hpi (s.i) | 3 | 5 | UBC18 | -0.25929 | -0.00038 |
| IAC91-1099 24 hpi (s.i) | 3 | 6 | UBC18 | -0.25945 | 2.37E-05 |
| IAC91-1099 24 hpi (s.i) | 3 | 7 | UBC18 | -0.26001 | 3.29E-05 |
| IAC91-1099 24 hpi (s.i) | 3 | 8 | UBC18 | -0.26048 | 0.000127 |
| IAC91-1099 24 hpi (s.i) | 3 | 9 | UBC18 | -0.26003 | 0.001145 |
| IAC91-1099 24 hpi (s.i) | 3 | 10 | UBC18 | -0.26134 | 0.000402 |
| IAC91-1099 24 hpi (s.i) | 3 | 11 | UBC18 | -0.26129 | 0.001021 |
| IAC91-1099 24 hpi (s.i) | 3 | 12 | UBC18 | -0.26222 | 0.000659 |
| IAC91-1099 24 hpi (s.i) | 3 | 13 | UBC18 | -0.26051 | 0.002929 |
| IAC91-1099 24 hpi (s.i) | 3 | 14 | UBC18 | -0.26118 | 0.002826 |
| IAC91-1099 24 hpi (s.i) | 3 | 15 | UBC18 | -0.26332 | 0.001256 |
| IAC91-1099 24 hpi (s.i) | 3 | 16 | UBC18 | -0.26292 | 0.002222 |
| IAC91-1099 24 hpi (s.i) | 3 | 17 | UBC18 | -0.26493 | 0.000776 |
| IAC91-1099 24 hpi (s.i) | 3 | 18 | UBC18 | -0.26626 | 1.13E-05 |
| IAC91-1099 24 hpi (s.i) | 3 | 19 | UBC18 | -0.27025 | -0.00341 |
| IAC91-1099 24 hpi (s.i) | 3 | 20 | UBC18 | -0.27001 | -0.00261 |
| IAC91-1099 24 hpi (s.i) | 3 | 21 | UBC18 | -0.26905 | -0.00108 |
| IAC91-1099 24 hpi (s.i) | 3 | 22 | UBC18 | -0.27035 | -0.00182 |
| IAC91-1099 24 hpi (s.i) | 3 | 23 | UBC18 | -0.26833 | 0.000778 |
| IAC91-1099 24 hpi (s.i) | 3 | 24 | UBC18 | -0.26217 | 0.007497 |
| IAC91-1099 24 hpi (s.i) | 3 | 25 | UBC18 | -0.24965 | 0.020584 |
| IAC91-1099 24 hpi (s.i) | 3 | 26 | UBC18 | -0.22361 | 0.047192 |
| IAC91-1099 24 hpi (s.i) | 3 | 27 | UBC18 | -0.17447 | 0.096903 |
| IAC91-1099 24 hpi (s.i) | 3 | 28 | UBC18 | -0.07753 | 0.194401 |
| IAC91-1099 24 hpi (s.i) | 3 | 29 | UBC18 | 0.102189 | 0.374691 |
| IAC91-1099 24 hpi (s.i) | 3 | 30 | UBC18 | 0.424157 | 0.697226 |
| IAC91-1099 24 hpi (s.i) | 3 | 31 | UBC18 | 0.924445 | 1.198079 |
| IAC91-1099 24 hpi (s.i) | 3 | 32 | UBC18 | 1.538837 | 1.813038 |
| IAC91-1099 24 hpi (s.i) | 3 | 33 | UBC18 | 2.170794 | 2.445562 |
| IAC91-1099 24 hpi (s.i) | 3 | 34 | UBC18 | 2.762237 | 3.037571 |
| IAC91-1099 24 hpi (s.i) | 3 | 35 | UBC18 | 3.304017 | 3.579917 |
| IAC91-1099 24 hpi (s.i) | 3 | 36 | UBC18 | 3.756691 | 4.033157 |
| IAC91-1099 24 hpi (s.i) | 3 | 37 | UBC18 | 4.126881 | 4.403913 |
| IAC91-1099 24 hpi (s.i) | 3 | 38 | UBC18 | 4.477593 | 4.755192 |
| IAC91-1099 24 hpi (s.i) | 3 | 39 | UBC18 | 4.729102 | 5.007267 |
| IAC91-1099 24 hpi (s.i) | 3 | 40 | UBC18 | 4.957556 | 5.236288 |
| IAC91-1099 72 hpi (m.i) | 1 | 1 | UBC18 | -0.3193 | -0.01481 |
| IAC91-1099 72 hpi (m.i) | 1 | 2 | UBC18 | -0.31453 | -0.00998 |
| IAC91-1099 72 hpi (m.i) | 1 | 3 | UBC18 | -0.31045 | -0.00584 |
| IAC91-1099 72 hpi (m.i) | 1 | 4 | UBC18 | -0.30477 | -9.9E-05 |
| IAC91-1099 72 hpi (m.i) | 1 | 5 | UBC18 | -0.306 | -0.00127 |
| IAC91-1099 72 hpi (m.i) | 1 | 6 | UBC18 | -0.30321 | 0.001584 |
| IAC91-1099 72 hpi (m.i) | 1 | 7 | UBC18 | -0.30359 | 0.001266 |
| IAC91-1099 72 hpi (m.i) | 1 | 8 | UBC18 | -0.30246 | 0.002462 |
| IAC91-1099 72 hpi (m.i) | 1 | 9 | UBC18 | -0.30231 | 0.002677 |
| IAC91-1099 72 hpi (m.i) | 1 | 10 | UBC18 | -0.30232 | 0.002727 |
| IAC91-1099 72 hpi (m.i) | 1 | 11 | UBC18 | -0.30285 | 0.002256 |
| IAC91-1099 72 hpi (m.i) | 1 | 12 | UBC18 | -0.30426 | 0.00091 |
| IAC91-1099 72 hpi (m.i) | 1 | 13 | UBC18 | -0.30384 | 0.00139 |
| IAC91-1099 72 hpi (m.i) | 1 | 14 | UBC18 | -0.30527 | 2.48E-05 |
| IAC91-1099 72 hpi (m.i) | 1 | 15 | UBC18 | -0.30769 | -0.00233 |
| IAC91-1099 72 hpi (m.i) | 1 | 16 | UBC18 | -0.30697 | -0.00156 |
| IAC91-1099 72 hpi (m.i) | 1 | 17 | UBC18 | -0.30804 | -0.00256 |
| IAC91-1099 72 hpi (m.i) | 1 | 18 | UBC18 | -0.30748 | -0.00194 |
| IAC91-1099 72 hpi (m.i) | 1 | 19 | UBC18 | -0.30693 | -0.00133 |
| IAC91-1099 72 hpi (m.i) | 1 | 20 | UBC18 | -0.30731 | -0.00165 |
| IAC91-1099 72 hpi (m.i) | 1 | 21 | UBC18 | -0.30771 | -0.00199 |
| IAC91-1099 72 hpi (m.i) | 1 | 22 | UBC18 | -0.3037 | 0.002086 |
| IAC91-1099 72 hpi (m.i) | 1 | 23 | UBC18 | -0.30267 | 0.003179 |
| IAC91-1099 72 hpi (m.i) | 1 | 24 | UBC18 | -0.29452 | 0.011392 |
| IAC91-1099 72 hpi (m.i) | 1 | 25 | UBC18 | -0.27898 | 0.02699 |
| IAC91-1099 72 hpi (m.i) | 1 | 26 | UBC18 | -0.24979 | 0.056249 |
| IAC91-1099 72 hpi (m.i) | 1 | 27 | UBC18 | -0.1917 | 0.114396 |
| IAC91-1099 72 hpi (m.i) | 1 | 28 | UBC18 | -0.07347 | 0.232695 |
| IAC91-1099 72 hpi (m.i) | 1 | 29 | UBC18 | 0.140938 | 0.447161 |
| IAC91-1099 72 hpi (m.i) | 1 | 30 | UBC18 | 0.522334 | 0.828618 |
| IAC91-1099 72 hpi (m.i) | 1 | 31 | UBC18 | 1.1144 | 1.420746 |
| IAC91-1099 72 hpi (m.i) | 1 | 32 | UBC18 | 1.839895 | 2.146303 |
| IAC91-1099 72 hpi (m.i) | 1 | 33 | UBC18 | 2.5629 | 2.86937 |
| IAC91-1099 72 hpi (m.i) | 1 | 34 | UBC18 | 3.221148 | 3.52768 |
| IAC91-1099 72 hpi (m.i) | 1 | 35 | UBC18 | 3.81371 | 4.120305 |
| IAC91-1099 72 hpi (m.i) | 1 | 36 | UBC18 | 4.288143 | 4.594799 |
| IAC91-1099 72 hpi (m.i) | 1 | 37 | UBC18 | 4.685314 | 4.992033 |
| IAC91-1099 72 hpi (m.i) | 1 | 38 | UBC18 | 5.047476 | 5.354257 |
| IAC91-1099 72 hpi (m.i) | 1 | 39 | UBC18 | 5.294808 | 5.601651 |
| IAC91-1099 72 hpi (m.i) | 1 | 40 | UBC18 | 5.529799 | 5.836703 |
| IAC91-1099 72 hpi (m.i) | 2 | 1 | UBC18 | -0.27766 | -0.01063 |
| IAC91-1099 72 hpi (m.i) | 2 | 2 | UBC18 | -0.27267 | -0.00541 |
| IAC91-1099 72 hpi (m.i) | 2 | 3 | UBC18 | -0.27034 | -0.00285 |
| IAC91-1099 72 hpi (m.i) | 2 | 4 | UBC18 | -0.2676 | 0.000124 |
| IAC91-1099 72 hpi (m.i) | 2 | 5 | UBC18 | -0.26711 | 0.000836 |
| IAC91-1099 72 hpi (m.i) | 2 | 6 | UBC18 | -0.26664 | 0.00154 |
| IAC91-1099 72 hpi (m.i) | 2 | 7 | UBC18 | -0.26621 | 0.002205 |
| IAC91-1099 72 hpi (m.i) | 2 | 8 | UBC18 | -0.26731 | 0.001338 |
| IAC91-1099 72 hpi (m.i) | 2 | 9 | UBC18 | -0.26547 | 0.003404 |
| IAC91-1099 72 hpi (m.i) | 2 | 10 | UBC18 | -0.26751 | 0.001593 |
| IAC91-1099 72 hpi (m.i) | 2 | 11 | UBC18 | -0.26868 | 0.00066 |
| IAC91-1099 72 hpi (m.i) | 2 | 12 | UBC18 | -0.27183 | -0.00226 |
| IAC91-1099 72 hpi (m.i) | 2 | 13 | UBC18 | -0.27208 | -0.00228 |
| IAC91-1099 72 hpi (m.i) | 2 | 14 | UBC18 | -0.27181 | -0.00178 |
| IAC91-1099 72 hpi (m.i) | 2 | 15 | UBC18 | -0.27234 | -0.00208 |
| IAC91-1099 72 hpi (m.i) | 2 | 16 | UBC18 | -0.2733 | -0.00281 |
| IAC91-1099 72 hpi (m.i) | 2 | 17 | UBC18 | -0.27233 | -0.00161 |
| IAC91-1099 72 hpi (m.i) | 2 | 18 | UBC18 | -0.2731 | -0.00215 |
| IAC91-1099 72 hpi (m.i) | 2 | 19 | UBC18 | -0.27322 | -0.00203 |
| IAC91-1099 72 hpi (m.i) | 2 | 20 | UBC18 | -0.27125 | 0.000164 |
| IAC91-1099 72 hpi (m.i) | 2 | 21 | UBC18 | -0.26971 | 0.001932 |
| IAC91-1099 72 hpi (m.i) | 2 | 22 | UBC18 | -0.2658 | 0.006071 |
| IAC91-1099 72 hpi (m.i) | 2 | 23 | UBC18 | -0.26162 | 0.010488 |
| IAC91-1099 72 hpi (m.i) | 2 | 24 | UBC18 | -0.24965 | 0.022688 |
| IAC91-1099 72 hpi (m.i) | 2 | 25 | UBC18 | -0.22563 | 0.046941 |
| IAC91-1099 72 hpi (m.i) | 2 | 26 | UBC18 | -0.17972 | 0.093074 |
| IAC91-1099 72 hpi (m.i) | 2 | 27 | UBC18 | -0.09636 | 0.176667 |
| IAC91-1099 72 hpi (m.i) | 2 | 28 | UBC18 | 0.074584 | 0.347845 |
| IAC91-1099 72 hpi (m.i) | 2 | 29 | UBC18 | 0.373985 | 0.647477 |
| IAC91-1099 72 hpi (m.i) | 2 | 30 | UBC18 | 0.880999 | 1.154721 |
| IAC91-1099 72 hpi (m.i) | 2 | 31 | UBC18 | 1.600879 | 1.874832 |
| IAC91-1099 72 hpi (m.i) | 2 | 32 | UBC18 | 2.384833 | 2.659017 |
| IAC91-1099 72 hpi (m.i) | 2 | 33 | UBC18 | 3.13019 | 3.404604 |
| IAC91-1099 72 hpi (m.i) | 2 | 34 | UBC18 | 3.790201 | 4.064847 |
| IAC91-1099 72 hpi (m.i) | 2 | 35 | UBC18 | 4.365472 | 4.640348 |
| IAC91-1099 72 hpi (m.i) | 2 | 36 | UBC18 | 4.82658 | 5.101687 |
| IAC91-1099 72 hpi (m.i) | 2 | 37 | UBC18 | 5.213233 | 5.488571 |
| IAC91-1099 72 hpi (m.i) | 2 | 38 | UBC18 | 5.548573 | 5.824142 |
| IAC91-1099 72 hpi (m.i) | 2 | 39 | UBC18 | 5.796268 | 6.072068 |
| IAC91-1099 72 hpi (m.i) | 2 | 40 | UBC18 | 6.015983 | 6.292014 |
| IAC91-1099 72 hpi (m.i) | 3 | 1 | UBC18 | -0.32091 | -0.00943 |
| IAC91-1099 72 hpi (m.i) | 3 | 2 | UBC18 | -0.31617 | -0.00477 |
| IAC91-1099 72 hpi (m.i) | 3 | 3 | UBC18 | -0.31364 | -0.00233 |
| IAC91-1099 72 hpi (m.i) | 3 | 4 | UBC18 | -0.30999 | 0.001237 |
| IAC91-1099 72 hpi (m.i) | 3 | 5 | UBC18 | -0.31114 | -5.2E-06 |
| IAC91-1099 72 hpi (m.i) | 3 | 6 | UBC18 | -0.31054 | 0.00051 |
| IAC91-1099 72 hpi (m.i) | 3 | 7 | UBC18 | -0.30985 | 0.001115 |
| IAC91-1099 72 hpi (m.i) | 3 | 8 | UBC18 | -0.31099 | -0.0001 |
| IAC91-1099 72 hpi (m.i) | 3 | 9 | UBC18 | -0.30998 | 0.000821 |
| IAC91-1099 72 hpi (m.i) | 3 | 10 | UBC18 | -0.31029 | 0.000419 |
| IAC91-1099 72 hpi (m.i) | 3 | 11 | UBC18 | -0.3109 | -0.00027 |
| IAC91-1099 72 hpi (m.i) | 3 | 12 | UBC18 | -0.31045 | 9.17E-05 |
| IAC91-1099 72 hpi (m.i) | 3 | 13 | UBC18 | -0.30938 | 0.001078 |
| IAC91-1099 72 hpi (m.i) | 3 | 14 | UBC18 | -0.31035 | 2.25E-05 |
| IAC91-1099 72 hpi (m.i) | 3 | 15 | UBC18 | -0.31093 | -0.00064 |
| IAC91-1099 72 hpi (m.i) | 3 | 16 | UBC18 | -0.31135 | -0.00115 |
| IAC91-1099 72 hpi (m.i) | 3 | 17 | UBC18 | -0.31174 | -0.00162 |
| IAC91-1099 72 hpi (m.i) | 3 | 18 | UBC18 | -0.30963 | 0.000407 |
| IAC91-1099 72 hpi (m.i) | 3 | 19 | UBC18 | -0.31097 | -0.00102 |
| IAC91-1099 72 hpi (m.i) | 3 | 20 | UBC18 | -0.31192 | -0.00206 |
| IAC91-1099 72 hpi (m.i) | 3 | 21 | UBC18 | -0.30948 | 0.000296 |
| IAC91-1099 72 hpi (m.i) | 3 | 22 | UBC18 | -0.30647 | 0.003218 |
| IAC91-1099 72 hpi (m.i) | 3 | 23 | UBC18 | -0.29945 | 0.010159 |
| IAC91-1099 72 hpi (m.i) | 3 | 24 | UBC18 | -0.28517 | 0.024352 |
| IAC91-1099 72 hpi (m.i) | 3 | 25 | UBC18 | -0.25956 | 0.049874 |
| IAC91-1099 72 hpi (m.i) | 3 | 26 | UBC18 | -0.20737 | 0.101984 |
| IAC91-1099 72 hpi (m.i) | 3 | 27 | UBC18 | -0.10931 | 0.19996 |
| IAC91-1099 72 hpi (m.i) | 3 | 28 | UBC18 | 0.086653 | 0.395835 |
| IAC91-1099 72 hpi (m.i) | 3 | 29 | UBC18 | 0.43033 | 0.739427 |
| IAC91-1099 72 hpi (m.i) | 3 | 30 | UBC18 | 1.012388 | 1.321399 |
| IAC91-1099 72 hpi (m.i) | 3 | 31 | UBC18 | 1.831468 | 2.140395 |
| IAC91-1099 72 hpi (m.i) | 3 | 32 | UBC18 | 2.703494 | 3.012335 |
| IAC91-1099 72 hpi (m.i) | 3 | 33 | UBC18 | 3.525467 | 3.834223 |
| IAC91-1099 72 hpi (m.i) | 3 | 34 | UBC18 | 4.252452 | 4.561123 |
| IAC91-1099 72 hpi (m.i) | 3 | 35 | UBC18 | 4.864071 | 5.172657 |
| IAC91-1099 72 hpi (m.i) | 3 | 36 | UBC18 | 5.352696 | 5.661198 |
| IAC91-1099 72 hpi (m.i) | 3 | 37 | UBC18 | 5.769276 | 6.077692 |
| IAC91-1099 72 hpi (m.i) | 3 | 38 | UBC18 | 6.110977 | 6.419308 |
| IAC91-1099 72 hpi (m.i) | 3 | 39 | UBC18 | 6.360242 | 6.668488 |
| IAC91-1099 72 hpi (m.i) | 3 | 40 | UBC18 | 6.597758 | 6.905919 |
| IAC91-1099 72 hpi (s.i) | 1 | 1 | UBC18 | -0.34249 | -0.02064 |
| IAC91-1099 72 hpi (s.i) | 1 | 2 | UBC18 | -0.33514 | -0.01288 |
| IAC91-1099 72 hpi (s.i) | 1 | 3 | UBC18 | -0.32554 | -0.00288 |
| IAC91-1099 72 hpi (s.i) | 1 | 4 | UBC18 | -0.32855 | -0.00549 |
| IAC91-1099 72 hpi (s.i) | 1 | 5 | UBC18 | -0.3218 | 0.001662 |
| IAC91-1099 72 hpi (s.i) | 1 | 6 | UBC18 | -0.32019 | 0.003669 |
| IAC91-1099 72 hpi (s.i) | 1 | 7 | UBC18 | -0.32367 | 0.000588 |
| IAC91-1099 72 hpi (s.i) | 1 | 8 | UBC18 | -0.32356 | 0.001108 |
| IAC91-1099 72 hpi (s.i) | 1 | 9 | UBC18 | -0.32266 | 0.0024 |
| IAC91-1099 72 hpi (s.i) | 1 | 10 | UBC18 | -0.32364 | 0.00183 |
| IAC91-1099 72 hpi (s.i) | 1 | 11 | UBC18 | -0.32418 | 0.001685 |
| IAC91-1099 72 hpi (s.i) | 1 | 12 | UBC18 | -0.32708 | -0.00081 |
| IAC91-1099 72 hpi (s.i) | 1 | 13 | UBC18 | -0.32623 | 0.000435 |
| IAC91-1099 72 hpi (s.i) | 1 | 14 | UBC18 | -0.32709 | -1.8E-05 |
| IAC91-1099 72 hpi (s.i) | 1 | 15 | UBC18 | -0.32858 | -0.00111 |
| IAC91-1099 72 hpi (s.i) | 1 | 16 | UBC18 | -0.32509 | 0.002782 |
| IAC91-1099 72 hpi (s.i) | 1 | 17 | UBC18 | -0.32947 | -0.00119 |
| IAC91-1099 72 hpi (s.i) | 1 | 18 | UBC18 | -0.3314 | -0.00273 |
| IAC91-1099 72 hpi (s.i) | 1 | 19 | UBC18 | -0.33172 | -0.00265 |
| IAC91-1099 72 hpi (s.i) | 1 | 20 | UBC18 | -0.33147 | -0.00199 |
| IAC91-1099 72 hpi (s.i) | 1 | 21 | UBC18 | -0.33208 | -0.0022 |
| IAC91-1099 72 hpi (s.i) | 1 | 22 | UBC18 | -0.32835 | 0.001925 |
| IAC91-1099 72 hpi (s.i) | 1 | 23 | UBC18 | -0.32769 | 0.00299 |
| IAC91-1099 72 hpi (s.i) | 1 | 24 | UBC18 | -0.32012 | 0.010964 |
| IAC91-1099 72 hpi (s.i) | 1 | 25 | UBC18 | -0.3084 | 0.023086 |
| IAC91-1099 72 hpi (s.i) | 1 | 26 | UBC18 | -0.28413 | 0.047758 |
| IAC91-1099 72 hpi (s.i) | 1 | 27 | UBC18 | -0.23653 | 0.095754 |
| IAC91-1099 72 hpi (s.i) | 1 | 28 | UBC18 | -0.13687 | 0.195818 |
| IAC91-1099 72 hpi (s.i) | 1 | 29 | UBC18 | 0.044297 | 0.377384 |
| IAC91-1099 72 hpi (s.i) | 1 | 30 | UBC18 | 0.367325 | 0.700814 |
| IAC91-1099 72 hpi (s.i) | 1 | 31 | UBC18 | 0.889472 | 1.223361 |
| IAC91-1099 72 hpi (s.i) | 1 | 32 | UBC18 | 1.562614 | 1.896905 |
| IAC91-1099 72 hpi (s.i) | 1 | 33 | UBC18 | 2.268752 | 2.603444 |
| IAC91-1099 72 hpi (s.i) | 1 | 34 | UBC18 | 2.916123 | 3.251216 |
| IAC91-1099 72 hpi (s.i) | 1 | 35 | UBC18 | 3.523848 | 3.859343 |
| IAC91-1099 72 hpi (s.i) | 1 | 36 | UBC18 | 4.035011 | 4.370907 |
| IAC91-1099 72 hpi (s.i) | 1 | 37 | UBC18 | 4.445478 | 4.781775 |
| IAC91-1099 72 hpi (s.i) | 1 | 38 | UBC18 | 4.832716 | 5.169413 |
| IAC91-1099 72 hpi (s.i) | 1 | 39 | UBC18 | 5.119105 | 5.456204 |
| IAC91-1099 72 hpi (s.i) | 1 | 40 | UBC18 | 5.373438 | 5.710938 |
| IAC91-1099 72 hpi (s.i) | 2 | 1 | UBC18 | -0.34579 | -0.00703 |
| IAC91-1099 72 hpi (s.i) | 2 | 2 | UBC18 | -0.33693 | 0.001608 |
| IAC91-1099 72 hpi (s.i) | 2 | 3 | UBC18 | -0.34419 | -0.00586 |
| IAC91-1099 72 hpi (s.i) | 2 | 4 | UBC18 | -0.33216 | 0.005959 |
| IAC91-1099 72 hpi (s.i) | 2 | 5 | UBC18 | -0.34008 | -0.00217 |
| IAC91-1099 72 hpi (s.i) | 2 | 6 | UBC18 | -0.34001 | -0.00232 |
| IAC91-1099 72 hpi (s.i) | 2 | 7 | UBC18 | -0.33165 | 0.005832 |
| IAC91-1099 72 hpi (s.i) | 2 | 8 | UBC18 | -0.33423 | 0.003044 |
| IAC91-1099 72 hpi (s.i) | 2 | 9 | UBC18 | -0.33351 | 0.003551 |
| IAC91-1099 72 hpi (s.i) | 2 | 10 | UBC18 | -0.33687 | -1.6E-05 |
| IAC91-1099 72 hpi (s.i) | 2 | 11 | UBC18 | -0.3365 | 0.000138 |
| IAC91-1099 72 hpi (s.i) | 2 | 12 | UBC18 | -0.33596 | 0.00047 |
| IAC91-1099 72 hpi (s.i) | 2 | 13 | UBC18 | -0.33921 | -0.00299 |
| IAC91-1099 72 hpi (s.i) | 2 | 14 | UBC18 | -0.33521 | 0.000802 |
| IAC91-1099 72 hpi (s.i) | 2 | 15 | UBC18 | -0.33745 | -0.00165 |
| IAC91-1099 72 hpi (s.i) | 2 | 16 | UBC18 | -0.34706 | -0.01147 |
| IAC91-1099 72 hpi (s.i) | 2 | 17 | UBC18 | -0.33594 | -0.00056 |
| IAC91-1099 72 hpi (s.i) | 2 | 18 | UBC18 | -0.33384 | 0.001327 |
| IAC91-1099 72 hpi (s.i) | 2 | 19 | UBC18 | -0.33551 | -0.00056 |
| IAC91-1099 72 hpi (s.i) | 2 | 20 | UBC18 | -0.33391 | 0.000835 |
| IAC91-1099 72 hpi (s.i) | 2 | 21 | UBC18 | -0.33521 | -0.00068 |
| IAC91-1099 72 hpi (s.i) | 2 | 22 | UBC18 | -0.33318 | 0.001135 |
| IAC91-1099 72 hpi (s.i) | 2 | 23 | UBC18 | -0.32892 | 0.005185 |
| IAC91-1099 72 hpi (s.i) | 2 | 24 | UBC18 | -0.32554 | 0.008355 |
| IAC91-1099 72 hpi (s.i) | 2 | 25 | UBC18 | -0.3102 | 0.02349 |
| IAC91-1099 72 hpi (s.i) | 2 | 26 | UBC18 | -0.28256 | 0.050914 |
| IAC91-1099 72 hpi (s.i) | 2 | 27 | UBC18 | -0.22971 | 0.103553 |
| IAC91-1099 72 hpi (s.i) | 2 | 28 | UBC18 | -0.12761 | 0.205442 |
| IAC91-1099 72 hpi (s.i) | 2 | 29 | UBC18 | 0.057119 | 0.38996 |
| IAC91-1099 72 hpi (s.i) | 2 | 30 | UBC18 | 0.418193 | 0.750822 |
| IAC91-1099 72 hpi (s.i) | 2 | 31 | UBC18 | 0.974968 | 1.307386 |
| IAC91-1099 72 hpi (s.i) | 2 | 32 | UBC18 | 1.714663 | 2.04687 |
| IAC91-1099 72 hpi (s.i) | 2 | 33 | UBC18 | 2.486102 | 2.818098 |
| IAC91-1099 72 hpi (s.i) | 2 | 34 | UBC18 | 3.195139 | 3.526924 |
| IAC91-1099 72 hpi (s.i) | 2 | 35 | UBC18 | 3.839783 | 4.171356 |
| IAC91-1099 72 hpi (s.i) | 2 | 36 | UBC18 | 4.364377 | 4.695739 |
| IAC91-1099 72 hpi (s.i) | 2 | 37 | UBC18 | 4.804811 | 5.135962 |
| IAC91-1099 72 hpi (s.i) | 2 | 38 | UBC18 | 5.200474 | 5.531415 |
| IAC91-1099 72 hpi (s.i) | 2 | 39 | UBC18 | 5.4842 | 5.814929 |
| IAC91-1099 72 hpi (s.i) | 2 | 40 | UBC18 | 5.754196 | 6.084714 |
| IAC91-1099 72 hpi (s.i) | 3 | 1 | UBC18 | -0.24234 | -0.01065 |
| IAC91-1099 72 hpi (s.i) | 3 | 2 | UBC18 | -0.24635 | -0.01342 |
| IAC91-1099 72 hpi (s.i) | 3 | 3 | UBC18 | -0.22981 | 0.004362 |
| IAC91-1099 72 hpi (s.i) | 3 | 4 | UBC18 | -0.24327 | -0.00786 |
| IAC91-1099 72 hpi (s.i) | 3 | 5 | UBC18 | -0.2296 | 0.007052 |
| IAC91-1099 72 hpi (s.i) | 3 | 6 | UBC18 | -0.22877 | 0.009128 |
| IAC91-1099 72 hpi (s.i) | 3 | 7 | UBC18 | -0.24583 | -0.0067 |
| IAC91-1099 72 hpi (s.i) | 3 | 8 | UBC18 | -0.24417 | -0.0038 |
| IAC91-1099 72 hpi (s.i) | 3 | 9 | UBC18 | -0.24429 | -0.00267 |
| IAC91-1099 72 hpi (s.i) | 3 | 10 | UBC18 | -0.24553 | -0.00267 |
| IAC91-1099 72 hpi (s.i) | 3 | 11 | UBC18 | -0.24476 | -0.00066 |
| IAC91-1099 72 hpi (s.i) | 3 | 12 | UBC18 | -0.24746 | -0.00212 |
| IAC91-1099 72 hpi (s.i) | 3 | 13 | UBC18 | -0.24343 | 0.003156 |
| IAC91-1099 72 hpi (s.i) | 3 | 14 | UBC18 | -0.24943 | -0.00161 |
| IAC91-1099 72 hpi (s.i) | 3 | 15 | UBC18 | -0.25207 | -0.003 |
| IAC91-1099 72 hpi (s.i) | 3 | 16 | UBC18 | -0.2379 | 0.012401 |
| IAC91-1099 72 hpi (s.i) | 3 | 17 | UBC18 | -0.2524 | -0.00085 |
| IAC91-1099 72 hpi (s.i) | 3 | 18 | UBC18 | -0.2567 | -0.00392 |
| IAC91-1099 72 hpi (s.i) | 3 | 19 | UBC18 | -0.25543 | -0.0014 |
| IAC91-1099 72 hpi (s.i) | 3 | 20 | UBC18 | -0.25707 | -0.0018 |
| IAC91-1099 72 hpi (s.i) | 3 | 21 | UBC18 | -0.25621 | 0.000301 |
| IAC91-1099 72 hpi (s.i) | 3 | 22 | UBC18 | -0.2551 | 0.002654 |
| IAC91-1099 72 hpi (s.i) | 3 | 23 | UBC18 | -0.25275 | 0.00624 |
| IAC91-1099 72 hpi (s.i) | 3 | 24 | UBC18 | -0.24267 | 0.017564 |
| IAC91-1099 72 hpi (s.i) | 3 | 25 | UBC18 | -0.22796 | 0.033518 |
| IAC91-1099 72 hpi (s.i) | 3 | 26 | UBC18 | -0.19495 | 0.067771 |
| IAC91-1099 72 hpi (s.i) | 3 | 27 | UBC18 | -0.1361 | 0.127862 |
| IAC91-1099 72 hpi (s.i) | 3 | 28 | UBC18 | -0.00509 | 0.260109 |
| IAC91-1099 72 hpi (s.i) | 3 | 29 | UBC18 | 0.227944 | 0.494386 |
| IAC91-1099 72 hpi (s.i) | 3 | 30 | UBC18 | 0.609451 | 0.877134 |
| IAC91-1099 72 hpi (s.i) | 3 | 31 | UBC18 | 1.198676 | 1.4676 |
| IAC91-1099 72 hpi (s.i) | 3 | 32 | UBC18 | 1.865657 | 2.135823 |
| IAC91-1099 72 hpi (s.i) | 3 | 33 | UBC18 | 2.525999 | 2.797406 |
| IAC91-1099 72 hpi (s.i) | 3 | 34 | UBC18 | 3.12503 | 3.397678 |
| IAC91-1099 72 hpi (s.i) | 3 | 35 | UBC18 | 3.667625 | 3.941514 |
| IAC91-1099 72 hpi (s.i) | 3 | 36 | UBC18 | 4.115895 | 4.391026 |
| IAC91-1099 72 hpi (s.i) | 3 | 37 | UBC18 | 4.477787 | 4.754159 |
| IAC91-1099 72 hpi (s.i) | 3 | 38 | UBC18 | 4.818162 | 5.095776 |
| IAC91-1099 72 hpi (s.i) | 3 | 39 | UBC18 | 5.060145 | 5.338999 |
| IAC91-1099 72 hpi (s.i) | 3 | 40 | UBC18 | 5.28849 | 5.568586 |
| IACSP95-5000 24 hpi (m.i) | 1 | 1 | UBC18 | -0.29926 | -0.00491 |
| IACSP95-5000 24 hpi (m.i) | 1 | 2 | UBC18 | -0.30353 | -0.00881 |
| IACSP95-5000 24 hpi (m.i) | 1 | 3 | UBC18 | -0.29715 | -0.00206 |
| IACSP95-5000 24 hpi (m.i) | 1 | 4 | UBC18 | -0.29583 | -0.00037 |
| IACSP95-5000 24 hpi (m.i) | 1 | 5 | UBC18 | -0.29692 | -0.00108 |
| IACSP95-5000 24 hpi (m.i) | 1 | 6 | UBC18 | -0.29559 | 0.00062 |
| IACSP95-5000 24 hpi (m.i) | 1 | 7 | UBC18 | -0.29423 | 0.002345 |
| IACSP95-5000 24 hpi (m.i) | 1 | 8 | UBC18 | -0.29313 | 0.003819 |
| IACSP95-5000 24 hpi (m.i) | 1 | 9 | UBC18 | -0.29602 | 0.001304 |
| IACSP95-5000 24 hpi (m.i) | 1 | 10 | UBC18 | -0.29646 | 0.00123 |
| IACSP95-5000 24 hpi (m.i) | 1 | 11 | UBC18 | -0.29634 | 0.001729 |
| IACSP95-5000 24 hpi (m.i) | 1 | 12 | UBC18 | -0.29854 | -0.00011 |
| IACSP95-5000 24 hpi (m.i) | 1 | 13 | UBC18 | -0.29861 | 0.000203 |
| IACSP95-5000 24 hpi (m.i) | 1 | 14 | UBC18 | -0.29906 | 0.000123 |
| IACSP95-5000 24 hpi (m.i) | 1 | 15 | UBC18 | -0.30141 | -0.00186 |
| IACSP95-5000 24 hpi (m.i) | 1 | 16 | UBC18 | -0.30372 | -0.00379 |
| IACSP95-5000 24 hpi (m.i) | 1 | 17 | UBC18 | -0.30268 | -0.00239 |
| IACSP95-5000 24 hpi (m.i) | 1 | 18 | UBC18 | -0.30249 | -0.00182 |
| IACSP95-5000 24 hpi (m.i) | 1 | 19 | UBC18 | -0.30339 | -0.00236 |
| IACSP95-5000 24 hpi (m.i) | 1 | 20 | UBC18 | -0.30502 | -0.00361 |
| IACSP95-5000 24 hpi (m.i) | 1 | 21 | UBC18 | -0.30485 | -0.00307 |
| IACSP95-5000 24 hpi (m.i) | 1 | 22 | UBC18 | -0.29904 | 0.003111 |
| IACSP95-5000 24 hpi (m.i) | 1 | 23 | UBC18 | -0.30115 | 0.001374 |
| IACSP95-5000 24 hpi (m.i) | 1 | 24 | UBC18 | -0.29625 | 0.006646 |
| IACSP95-5000 24 hpi (m.i) | 1 | 25 | UBC18 | -0.28674 | 0.016528 |
| IACSP95-5000 24 hpi (m.i) | 1 | 26 | UBC18 | -0.27055 | 0.033094 |
| IACSP95-5000 24 hpi (m.i) | 1 | 27 | UBC18 | -0.23942 | 0.064591 |
| IACSP95-5000 24 hpi (m.i) | 1 | 28 | UBC18 | -0.17227 | 0.132108 |
| IACSP95-5000 24 hpi (m.i) | 1 | 29 | UBC18 | -0.0562 | 0.248558 |
| IACSP95-5000 24 hpi (m.i) | 1 | 30 | UBC18 | 0.171766 | 0.476892 |
| IACSP95-5000 24 hpi (m.i) | 1 | 31 | UBC18 | 0.543614 | 0.849112 |
| IACSP95-5000 24 hpi (m.i) | 1 | 32 | UBC18 | 1.047897 | 1.353766 |
| IACSP95-5000 24 hpi (m.i) | 1 | 33 | UBC18 | 1.601425 | 1.907666 |
| IACSP95-5000 24 hpi (m.i) | 1 | 34 | UBC18 | 2.129578 | 2.436191 |
| IACSP95-5000 24 hpi (m.i) | 1 | 35 | UBC18 | 2.615684 | 2.922668 |
| IACSP95-5000 24 hpi (m.i) | 1 | 36 | UBC18 | 3.034114 | 3.34147 |
| IACSP95-5000 24 hpi (m.i) | 1 | 37 | UBC18 | 3.381251 | 3.688979 |
| IACSP95-5000 24 hpi (m.i) | 1 | 38 | UBC18 | 3.725679 | 4.033778 |
| IACSP95-5000 24 hpi (m.i) | 1 | 39 | UBC18 | 3.954467 | 4.262938 |
| IACSP95-5000 24 hpi (m.i) | 1 | 40 | UBC18 | 4.1912 | 4.500042 |
| IACSP95-5000 24 hpi (m.i) | 2 | 1 | UBC18 | -0.25775 | -0.00537 |
| IACSP95-5000 24 hpi (m.i) | 2 | 2 | UBC18 | -0.25811 | -0.00504 |
| IACSP95-5000 24 hpi (m.i) | 2 | 3 | UBC18 | -0.25446 | -0.00068 |
| IACSP95-5000 24 hpi (m.i) | 2 | 4 | UBC18 | -0.25476 | -0.00029 |
| IACSP95-5000 24 hpi (m.i) | 2 | 5 | UBC18 | -0.2557 | -0.00053 |
| IACSP95-5000 24 hpi (m.i) | 2 | 6 | UBC18 | -0.25486 | 0.001 |
| IACSP95-5000 24 hpi (m.i) | 2 | 7 | UBC18 | -0.25517 | 0.001391 |
| IACSP95-5000 24 hpi (m.i) | 2 | 8 | UBC18 | -0.25558 | 0.001678 |
| IACSP95-5000 24 hpi (m.i) | 2 | 9 | UBC18 | -0.25897 | -0.00102 |
| IACSP95-5000 24 hpi (m.i) | 2 | 10 | UBC18 | -0.25852 | 0.000134 |
| IACSP95-5000 24 hpi (m.i) | 2 | 11 | UBC18 | -0.25978 | -0.00043 |
| IACSP95-5000 24 hpi (m.i) | 2 | 12 | UBC18 | -0.26052 | -0.00047 |
| IACSP95-5000 24 hpi (m.i) | 2 | 13 | UBC18 | -0.25969 | 0.001058 |
| IACSP95-5000 24 hpi (m.i) | 2 | 14 | UBC18 | -0.26144 | 5.09E-06 |
| IACSP95-5000 24 hpi (m.i) | 2 | 15 | UBC18 | -0.26041 | 0.00173 |
| IACSP95-5000 24 hpi (m.i) | 2 | 16 | UBC18 | -0.26483 | -0.00199 |
| IACSP95-5000 24 hpi (m.i) | 2 | 17 | UBC18 | -0.26566 | -0.00212 |
| IACSP95-5000 24 hpi (m.i) | 2 | 18 | UBC18 | -0.26452 | -0.00029 |
| IACSP95-5000 24 hpi (m.i) | 2 | 19 | UBC18 | -0.26663 | -0.0017 |
| IACSP95-5000 24 hpi (m.i) | 2 | 20 | UBC18 | -0.26763 | -0.002 |
| IACSP95-5000 24 hpi (m.i) | 2 | 21 | UBC18 | -0.26727 | -0.00095 |
| IACSP95-5000 24 hpi (m.i) | 2 | 22 | UBC18 | -0.26154 | 0.005481 |
| IACSP95-5000 24 hpi (m.i) | 2 | 23 | UBC18 | -0.26003 | 0.007689 |
| IACSP95-5000 24 hpi (m.i) | 2 | 24 | UBC18 | -0.24668 | 0.021738 |
| IACSP95-5000 24 hpi (m.i) | 2 | 25 | UBC18 | -0.22967 | 0.039441 |
| IACSP95-5000 24 hpi (m.i) | 2 | 26 | UBC18 | -0.18884 | 0.080968 |
| IACSP95-5000 24 hpi (m.i) | 2 | 27 | UBC18 | -0.11431 | 0.156196 |
| IACSP95-5000 24 hpi (m.i) | 2 | 28 | UBC18 | 0.034892 | 0.306094 |
| IACSP95-5000 24 hpi (m.i) | 2 | 29 | UBC18 | 0.305583 | 0.577482 |
| IACSP95-5000 24 hpi (m.i) | 2 | 30 | UBC18 | 0.771332 | 1.043929 |
| IACSP95-5000 24 hpi (m.i) | 2 | 31 | UBC18 | 1.460266 | 1.73356 |
| IACSP95-5000 24 hpi (m.i) | 2 | 32 | UBC18 | 2.230345 | 2.504336 |
| IACSP95-5000 24 hpi (m.i) | 2 | 33 | UBC18 | 2.959845 | 3.234534 |
| IACSP95-5000 24 hpi (m.i) | 2 | 34 | UBC18 | 3.598505 | 3.87389 |
| IACSP95-5000 24 hpi (m.i) | 2 | 35 | UBC18 | 4.140506 | 4.416589 |
| IACSP95-5000 24 hpi (m.i) | 2 | 36 | UBC18 | 4.600794 | 4.877574 |
| IACSP95-5000 24 hpi (m.i) | 2 | 37 | UBC18 | 4.965458 | 5.242935 |
| IACSP95-5000 24 hpi (m.i) | 2 | 38 | UBC18 | 5.311244 | 5.589419 |
| IACSP95-5000 24 hpi (m.i) | 2 | 39 | UBC18 | 5.528899 | 5.80777 |
| IACSP95-5000 24 hpi (m.i) | 2 | 40 | UBC18 | 5.753141 | 6.03271 |
| IACSP95-5000 24 hpi (m.i) | 3 | 1 | UBC18 | -0.31127 | -0.00684 |
| IACSP95-5000 24 hpi (m.i) | 3 | 2 | UBC18 | -0.3079 | -0.00348 |
| IACSP95-5000 24 hpi (m.i) | 3 | 3 | UBC18 | -0.30615 | -0.00173 |
| IACSP95-5000 24 hpi (m.i) | 3 | 4 | UBC18 | -0.30541 | -0.001 |
| IACSP95-5000 24 hpi (m.i) | 3 | 5 | UBC18 | -0.30253 | 0.001886 |
| IACSP95-5000 24 hpi (m.i) | 3 | 6 | UBC18 | -0.30297 | 0.00145 |
| IACSP95-5000 24 hpi (m.i) | 3 | 7 | UBC18 | -0.3026 | 0.001811 |
| IACSP95-5000 24 hpi (m.i) | 3 | 8 | UBC18 | -0.30629 | -0.00187 |
| IACSP95-5000 24 hpi (m.i) | 3 | 9 | UBC18 | -0.30034 | 0.004075 |
| IACSP95-5000 24 hpi (m.i) | 3 | 10 | UBC18 | -0.30348 | 0.000927 |
| IACSP95-5000 24 hpi (m.i) | 3 | 11 | UBC18 | -0.30163 | 0.002778 |
| IACSP95-5000 24 hpi (m.i) | 3 | 12 | UBC18 | -0.30254 | 0.001865 |
| IACSP95-5000 24 hpi (m.i) | 3 | 13 | UBC18 | -0.30426 | 0.000143 |
| IACSP95-5000 24 hpi (m.i) | 3 | 14 | UBC18 | -0.30621 | -0.00181 |
| IACSP95-5000 24 hpi (m.i) | 3 | 15 | UBC18 | -0.30991 | -0.00551 |
| IACSP95-5000 24 hpi (m.i) | 3 | 16 | UBC18 | -0.30694 | -0.00254 |
| IACSP95-5000 24 hpi (m.i) | 3 | 17 | UBC18 | -0.30908 | -0.00468 |
| IACSP95-5000 24 hpi (m.i) | 3 | 18 | UBC18 | -0.30613 | -0.00173 |
| IACSP95-5000 24 hpi (m.i) | 3 | 19 | UBC18 | -0.30629 | -0.00189 |
| IACSP95-5000 24 hpi (m.i) | 3 | 20 | UBC18 | -0.30628 | -0.00188 |
| IACSP95-5000 24 hpi (m.i) | 3 | 21 | UBC18 | -0.30672 | -0.00232 |
| IACSP95-5000 24 hpi (m.i) | 3 | 22 | UBC18 | -0.3021 | 0.002294 |
| IACSP95-5000 24 hpi (m.i) | 3 | 23 | UBC18 | -0.29468 | 0.009713 |
| IACSP95-5000 24 hpi (m.i) | 3 | 24 | UBC18 | -0.29068 | 0.013712 |
| IACSP95-5000 24 hpi (m.i) | 3 | 25 | UBC18 | -0.26391 | 0.04048 |
| IACSP95-5000 24 hpi (m.i) | 3 | 26 | UBC18 | -0.22344 | 0.080954 |
| IACSP95-5000 24 hpi (m.i) | 3 | 27 | UBC18 | -0.14338 | 0.161016 |
| IACSP95-5000 24 hpi (m.i) | 3 | 28 | UBC18 | 0.015835 | 0.320225 |
| IACSP95-5000 24 hpi (m.i) | 3 | 29 | UBC18 | 0.302216 | 0.606605 |
| IACSP95-5000 24 hpi (m.i) | 3 | 30 | UBC18 | 0.805019 | 1.109407 |
| IACSP95-5000 24 hpi (m.i) | 3 | 31 | UBC18 | 1.528536 | 1.832923 |
| IACSP95-5000 24 hpi (m.i) | 3 | 32 | UBC18 | 2.325929 | 2.630315 |
| IACSP95-5000 24 hpi (m.i) | 3 | 33 | UBC18 | 3.071518 | 3.375903 |
| IACSP95-5000 24 hpi (m.i) | 3 | 34 | UBC18 | 3.697067 | 4.001451 |
| IACSP95-5000 24 hpi (m.i) | 3 | 35 | UBC18 | 4.264128 | 4.56851 |
| IACSP95-5000 24 hpi (m.i) | 3 | 36 | UBC18 | 4.693151 | 4.997533 |
| IACSP95-5000 24 hpi (m.i) | 3 | 37 | UBC18 | 5.049363 | 5.353743 |
| IACSP95-5000 24 hpi (m.i) | 3 | 38 | UBC18 | 5.368227 | 5.672606 |
| IACSP95-5000 24 hpi (m.i) | 3 | 39 | UBC18 | 5.60815 | 5.912528 |
| IACSP95-5000 24 hpi (m.i) | 3 | 40 | UBC18 | 5.814108 | 6.118485 |
| IACSP95-5000 24 hpi (s.i) | 1 | 1 | UBC18 | -0.29154 | -0.00914 |
| IACSP95-5000 24 hpi (s.i) | 1 | 2 | UBC18 | -0.2912 | -0.00811 |
| IACSP95-5000 24 hpi (s.i) | 1 | 3 | UBC18 | -0.28644 | -0.00266 |
| IACSP95-5000 24 hpi (s.i) | 1 | 4 | UBC18 | -0.28475 | -0.00029 |
| IACSP95-5000 24 hpi (s.i) | 1 | 5 | UBC18 | -0.28537 | -0.00022 |
| IACSP95-5000 24 hpi (s.i) | 1 | 6 | UBC18 | -0.28515 | 0.000685 |
| IACSP95-5000 24 hpi (s.i) | 1 | 7 | UBC18 | -0.28428 | 0.002238 |
| IACSP95-5000 24 hpi (s.i) | 1 | 8 | UBC18 | -0.28497 | 0.002231 |
| IACSP95-5000 24 hpi (s.i) | 1 | 9 | UBC18 | -0.28622 | 0.001666 |
| IACSP95-5000 24 hpi (s.i) | 1 | 10 | UBC18 | -0.28856 | 6.08E-06 |
| IACSP95-5000 24 hpi (s.i) | 1 | 11 | UBC18 | -0.28797 | 0.001278 |
| IACSP95-5000 24 hpi (s.i) | 1 | 12 | UBC18 | -0.28942 | 0.00052 |
| IACSP95-5000 24 hpi (s.i) | 1 | 13 | UBC18 | -0.29094 | -0.00032 |
| IACSP95-5000 24 hpi (s.i) | 1 | 14 | UBC18 | -0.29148 | -0.00018 |
| IACSP95-5000 24 hpi (s.i) | 1 | 15 | UBC18 | -0.29377 | -0.00178 |
| IACSP95-5000 24 hpi (s.i) | 1 | 16 | UBC18 | -0.29447 | -0.0018 |
| IACSP95-5000 24 hpi (s.i) | 1 | 17 | UBC18 | -0.29566 | -0.0023 |
| IACSP95-5000 24 hpi (s.i) | 1 | 18 | UBC18 | -0.29454 | -0.0005 |
| IACSP95-5000 24 hpi (s.i) | 1 | 19 | UBC18 | -0.29708 | -0.00236 |
| IACSP95-5000 24 hpi (s.i) | 1 | 20 | UBC18 | -0.29723 | -0.00182 |
| IACSP95-5000 24 hpi (s.i) | 1 | 21 | UBC18 | -0.29717 | -0.00107 |
| IACSP95-5000 24 hpi (s.i) | 1 | 22 | UBC18 | -0.29455 | 0.002227 |
| IACSP95-5000 24 hpi (s.i) | 1 | 23 | UBC18 | -0.29302 | 0.004445 |
| IACSP95-5000 24 hpi (s.i) | 1 | 24 | UBC18 | -0.28625 | 0.011898 |
| IACSP95-5000 24 hpi (s.i) | 1 | 25 | UBC18 | -0.27318 | 0.02565 |
| IACSP95-5000 24 hpi (s.i) | 1 | 26 | UBC18 | -0.24821 | 0.051308 |
| IACSP95-5000 24 hpi (s.i) | 1 | 27 | UBC18 | -0.19783 | 0.102367 |
| IACSP95-5000 24 hpi (s.i) | 1 | 28 | UBC18 | -0.09955 | 0.20134 |
| IACSP95-5000 24 hpi (s.i) | 1 | 29 | UBC18 | 0.083578 | 0.385147 |
| IACSP95-5000 24 hpi (s.i) | 1 | 30 | UBC18 | 0.419668 | 0.721922 |
| IACSP95-5000 24 hpi (s.i) | 1 | 31 | UBC18 | 0.955177 | 1.258115 |
| IACSP95-5000 24 hpi (s.i) | 1 | 32 | UBC18 | 1.641282 | 1.944905 |
| IACSP95-5000 24 hpi (s.i) | 1 | 33 | UBC18 | 2.343659 | 2.647966 |
| IACSP95-5000 24 hpi (s.i) | 1 | 34 | UBC18 | 2.973812 | 3.278803 |
| IACSP95-5000 24 hpi (s.i) | 1 | 35 | UBC18 | 3.526424 | 3.8321 |
| IACSP95-5000 24 hpi (s.i) | 1 | 36 | UBC18 | 3.977587 | 4.283947 |
| IACSP95-5000 24 hpi (s.i) | 1 | 37 | UBC18 | 4.347219 | 4.654263 |
| IACSP95-5000 24 hpi (s.i) | 1 | 38 | UBC18 | 4.693853 | 5.001582 |
| IACSP95-5000 24 hpi (s.i) | 1 | 39 | UBC18 | 4.926602 | 5.235014 |
| IACSP95-5000 24 hpi (s.i) | 1 | 40 | UBC18 | 5.155926 | 5.465023 |
| IACSP95-5000 24 hpi (s.i) | 2 | 1 | UBC18 | -0.27695 | -0.00201 |
| IACSP95-5000 24 hpi (s.i) | 2 | 2 | UBC18 | -0.27679 | -0.00103 |
| IACSP95-5000 24 hpi (s.i) | 2 | 3 | UBC18 | -0.27452 | 0.002063 |
| IACSP95-5000 24 hpi (s.i) | 2 | 4 | UBC18 | -0.27568 | 0.001719 |
| IACSP95-5000 24 hpi (s.i) | 2 | 5 | UBC18 | -0.27871 | -0.00049 |
| IACSP95-5000 24 hpi (s.i) | 2 | 6 | UBC18 | -0.27768 | 0.001359 |
| IACSP95-5000 24 hpi (s.i) | 2 | 7 | UBC18 | -0.27916 | 0.0007 |
| IACSP95-5000 24 hpi (s.i) | 2 | 8 | UBC18 | -0.28073 | -4.9E-05 |
| IACSP95-5000 24 hpi (s.i) | 2 | 9 | UBC18 | -0.28125 | 0.000253 |
| IACSP95-5000 24 hpi (s.i) | 2 | 10 | UBC18 | -0.28319 | -0.00087 |
| IACSP95-5000 24 hpi (s.i) | 2 | 11 | UBC18 | -0.28364 | -0.0005 |
| IACSP95-5000 24 hpi (s.i) | 2 | 12 | UBC18 | -0.28534 | -0.00137 |
| IACSP95-5000 24 hpi (s.i) | 2 | 13 | UBC18 | -0.28787 | -0.00308 |
| IACSP95-5000 24 hpi (s.i) | 2 | 14 | UBC18 | -0.28802 | -0.00241 |
| IACSP95-5000 24 hpi (s.i) | 2 | 15 | UBC18 | -0.28801 | -0.00158 |
| IACSP95-5000 24 hpi (s.i) | 2 | 16 | UBC18 | -0.28824 | -0.001 |
| IACSP95-5000 24 hpi (s.i) | 2 | 17 | UBC18 | -0.28939 | -0.00133 |
| IACSP95-5000 24 hpi (s.i) | 2 | 18 | UBC18 | -0.28641 | 0.002478 |
| IACSP95-5000 24 hpi (s.i) | 2 | 19 | UBC18 | -0.2923 | -0.00259 |
| IACSP95-5000 24 hpi (s.i) | 2 | 20 | UBC18 | -0.28993 | 0.000599 |
| IACSP95-5000 24 hpi (s.i) | 2 | 21 | UBC18 | -0.29105 | 0.0003 |
| IACSP95-5000 24 hpi (s.i) | 2 | 22 | UBC18 | -0.28638 | 0.005792 |
| IACSP95-5000 24 hpi (s.i) | 2 | 23 | UBC18 | -0.28314 | 0.009855 |
| IACSP95-5000 24 hpi (s.i) | 2 | 24 | UBC18 | -0.27144 | 0.022373 |
| IACSP95-5000 24 hpi (s.i) | 2 | 25 | UBC18 | -0.25172 | 0.042914 |
| IACSP95-5000 24 hpi (s.i) | 2 | 26 | UBC18 | -0.20928 | 0.086176 |
| IACSP95-5000 24 hpi (s.i) | 2 | 27 | UBC18 | -0.13058 | 0.165692 |
| IACSP95-5000 24 hpi (s.i) | 2 | 28 | UBC18 | 0.027294 | 0.324389 |
| IACSP95-5000 24 hpi (s.i) | 2 | 29 | UBC18 | 0.30912 | 0.607035 |
| IACSP95-5000 24 hpi (s.i) | 2 | 30 | UBC18 | 0.798776 | 1.097512 |
| IACSP95-5000 24 hpi (s.i) | 2 | 31 | UBC18 | 1.519374 | 1.81893 |
| IACSP95-5000 24 hpi (s.i) | 2 | 32 | UBC18 | 2.336 | 2.636377 |
| IACSP95-5000 24 hpi (s.i) | 2 | 33 | UBC18 | 3.087619 | 3.388817 |
| IACSP95-5000 24 hpi (s.i) | 2 | 34 | UBC18 | 3.734396 | 4.036415 |
| IACSP95-5000 24 hpi (s.i) | 2 | 35 | UBC18 | 4.279651 | 4.582489 |
| IACSP95-5000 24 hpi (s.i) | 2 | 36 | UBC18 | 4.710642 | 5.014302 |
| IACSP95-5000 24 hpi (s.i) | 2 | 37 | UBC18 | 5.062138 | 5.366618 |
| IACSP95-5000 24 hpi (s.i) | 2 | 38 | UBC18 | 5.379087 | 5.684388 |
| IACSP95-5000 24 hpi (s.i) | 2 | 39 | UBC18 | 5.608431 | 5.914553 |
| IACSP95-5000 24 hpi (s.i) | 2 | 40 | UBC18 | 5.802175 | 6.109117 |
| IACSP95-5000 24 hpi (s.i) | 3 | 1 | UBC18 | -0.26059 | -0.00915 |
| IACSP95-5000 24 hpi (s.i) | 3 | 2 | UBC18 | -0.25673 | -0.00466 |
| IACSP95-5000 24 hpi (s.i) | 3 | 3 | UBC18 | -0.2524 | 0.000287 |
| IACSP95-5000 24 hpi (s.i) | 3 | 4 | UBC18 | -0.25336 | -4.8E-05 |
| IACSP95-5000 24 hpi (s.i) | 3 | 5 | UBC18 | -0.2535 | 0.000427 |
| IACSP95-5000 24 hpi (s.i) | 3 | 6 | UBC18 | -0.2538 | 0.000746 |
| IACSP95-5000 24 hpi (s.i) | 3 | 7 | UBC18 | -0.2543 | 0.000873 |
| IACSP95-5000 24 hpi (s.i) | 3 | 8 | UBC18 | -0.25417 | 0.001621 |
| IACSP95-5000 24 hpi (s.i) | 3 | 9 | UBC18 | -0.25554 | 0.000873 |
| IACSP95-5000 24 hpi (s.i) | 3 | 10 | UBC18 | -0.25852 | -0.00149 |
| IACSP95-5000 24 hpi (s.i) | 3 | 11 | UBC18 | -0.25728 | 0.000375 |
| IACSP95-5000 24 hpi (s.i) | 3 | 12 | UBC18 | -0.25772 | 0.000555 |
| IACSP95-5000 24 hpi (s.i) | 3 | 13 | UBC18 | -0.25906 | -0.00016 |
| IACSP95-5000 24 hpi (s.i) | 3 | 14 | UBC18 | -0.26043 | -0.0009 |
| IACSP95-5000 24 hpi (s.i) | 3 | 15 | UBC18 | -0.26265 | -0.00251 |
| IACSP95-5000 24 hpi (s.i) | 3 | 16 | UBC18 | -0.26252 | -0.00176 |
| IACSP95-5000 24 hpi (s.i) | 3 | 17 | UBC18 | -0.26409 | -0.0027 |
| IACSP95-5000 24 hpi (s.i) | 3 | 18 | UBC18 | -0.26423 | -0.00222 |
| IACSP95-5000 24 hpi (s.i) | 3 | 19 | UBC18 | -0.2644 | -0.00177 |
| IACSP95-5000 24 hpi (s.i) | 3 | 20 | UBC18 | -0.26359 | -0.00033 |
| IACSP95-5000 24 hpi (s.i) | 3 | 21 | UBC18 | -0.26148 | 0.002394 |
| IACSP95-5000 24 hpi (s.i) | 3 | 22 | UBC18 | -0.25876 | 0.005734 |
| IACSP95-5000 24 hpi (s.i) | 3 | 23 | UBC18 | -0.2521 | 0.013016 |
| IACSP95-5000 24 hpi (s.i) | 3 | 24 | UBC18 | -0.24156 | 0.024176 |
| IACSP95-5000 24 hpi (s.i) | 3 | 25 | UBC18 | -0.21699 | 0.049368 |
| IACSP95-5000 24 hpi (s.i) | 3 | 26 | UBC18 | -0.17304 | 0.09394 |
| IACSP95-5000 24 hpi (s.i) | 3 | 27 | UBC18 | -0.08868 | 0.178923 |
| IACSP95-5000 24 hpi (s.i) | 3 | 28 | UBC18 | 0.079446 | 0.347671 |
| IACSP95-5000 24 hpi (s.i) | 3 | 29 | UBC18 | 0.381195 | 0.650043 |
| IACSP95-5000 24 hpi (s.i) | 3 | 30 | UBC18 | 0.898452 | 1.167921 |
| IACSP95-5000 24 hpi (s.i) | 3 | 31 | UBC18 | 1.647372 | 1.917463 |
| IACSP95-5000 24 hpi (s.i) | 3 | 32 | UBC18 | 2.475673 | 2.746385 |
| IACSP95-5000 24 hpi (s.i) | 3 | 33 | UBC18 | 3.230848 | 3.502182 |
| IACSP95-5000 24 hpi (s.i) | 3 | 34 | UBC18 | 3.880469 | 4.152424 |
| IACSP95-5000 24 hpi (s.i) | 3 | 35 | UBC18 | 4.430963 | 4.70354 |
| IACSP95-5000 24 hpi (s.i) | 3 | 36 | UBC18 | 4.863718 | 5.136917 |
| IACSP95-5000 24 hpi (s.i) | 3 | 37 | UBC18 | 5.214167 | 5.487987 |
| IACSP95-5000 24 hpi (s.i) | 3 | 38 | UBC18 | 5.534824 | 5.809266 |
| IACSP95-5000 24 hpi (s.i) | 3 | 39 | UBC18 | 5.75513 | 6.030194 |
| IACSP95-5000 24 hpi (s.i) | 3 | 40 | UBC18 | 5.963081 | 6.238766 |
| IACSP95-5000 72 hpi (m.i) | 1 | 1 | UBC18 | -0.29217 | -0.00878 |
| IACSP95-5000 72 hpi (m.i) | 1 | 2 | UBC18 | -0.292 | -0.00812 |
| IACSP95-5000 72 hpi (m.i) | 1 | 3 | UBC18 | -0.28948 | -0.00511 |
| IACSP95-5000 72 hpi (m.i) | 1 | 4 | UBC18 | -0.28573 | -0.00086 |
| IACSP95-5000 72 hpi (m.i) | 1 | 5 | UBC18 | -0.28564 | -0.00028 |
| IACSP95-5000 72 hpi (m.i) | 1 | 6 | UBC18 | -0.28384 | 0.00201 |
| IACSP95-5000 72 hpi (m.i) | 1 | 7 | UBC18 | -0.28327 | 0.003066 |
| IACSP95-5000 72 hpi (m.i) | 1 | 8 | UBC18 | -0.28444 | 0.002392 |
| IACSP95-5000 72 hpi (m.i) | 1 | 9 | UBC18 | -0.28384 | 0.003476 |
| IACSP95-5000 72 hpi (m.i) | 1 | 10 | UBC18 | -0.28696 | 0.000847 |
| IACSP95-5000 72 hpi (m.i) | 1 | 11 | UBC18 | -0.2879 | 0.000398 |
| IACSP95-5000 72 hpi (m.i) | 1 | 12 | UBC18 | -0.28935 | -0.00056 |
| IACSP95-5000 72 hpi (m.i) | 1 | 13 | UBC18 | -0.28823 | 0.001049 |
| IACSP95-5000 72 hpi (m.i) | 1 | 14 | UBC18 | -0.28951 | 0.000262 |
| IACSP95-5000 72 hpi (m.i) | 1 | 15 | UBC18 | -0.29228 | -0.00201 |
| IACSP95-5000 72 hpi (m.i) | 1 | 16 | UBC18 | -0.2928 | -0.00205 |
| IACSP95-5000 72 hpi (m.i) | 1 | 17 | UBC18 | -0.2929 | -0.00165 |
| IACSP95-5000 72 hpi (m.i) | 1 | 18 | UBC18 | -0.29378 | -0.00204 |
| IACSP95-5000 72 hpi (m.i) | 1 | 19 | UBC18 | -0.29343 | -0.0012 |
| IACSP95-5000 72 hpi (m.i) | 1 | 20 | UBC18 | -0.29556 | -0.00284 |
| IACSP95-5000 72 hpi (m.i) | 1 | 21 | UBC18 | -0.295 | -0.00179 |
| IACSP95-5000 72 hpi (m.i) | 1 | 22 | UBC18 | -0.29288 | 0.000819 |
| IACSP95-5000 72 hpi (m.i) | 1 | 23 | UBC18 | -0.28812 | 0.006072 |
| IACSP95-5000 72 hpi (m.i) | 1 | 24 | UBC18 | -0.28059 | 0.014092 |
| IACSP95-5000 72 hpi (m.i) | 1 | 25 | UBC18 | -0.26137 | 0.033807 |
| IACSP95-5000 72 hpi (m.i) | 1 | 26 | UBC18 | -0.22423 | 0.071439 |
| IACSP95-5000 72 hpi (m.i) | 1 | 27 | UBC18 | -0.15352 | 0.142639 |
| IACSP95-5000 72 hpi (m.i) | 1 | 28 | UBC18 | -0.01145 | 0.285201 |
| IACSP95-5000 72 hpi (m.i) | 1 | 29 | UBC18 | 0.240903 | 0.538044 |
| IACSP95-5000 72 hpi (m.i) | 1 | 30 | UBC18 | 0.670878 | 0.96851 |
| IACSP95-5000 72 hpi (m.i) | 1 | 31 | UBC18 | 1.271849 | 1.569973 |
| IACSP95-5000 72 hpi (m.i) | 1 | 32 | UBC18 | 1.915825 | 2.214439 |
| IACSP95-5000 72 hpi (m.i) | 1 | 33 | UBC18 | 2.511665 | 2.810771 |
| IACSP95-5000 72 hpi (m.i) | 1 | 34 | UBC18 | 3.035704 | 3.3353 |
| IACSP95-5000 72 hpi (m.i) | 1 | 35 | UBC18 | 3.48789 | 3.787977 |
| IACSP95-5000 72 hpi (m.i) | 1 | 36 | UBC18 | 3.863836 | 4.164414 |
| IACSP95-5000 72 hpi (m.i) | 1 | 37 | UBC18 | 4.169361 | 4.47043 |
| IACSP95-5000 72 hpi (m.i) | 1 | 38 | UBC18 | 4.447188 | 4.748749 |
| IACSP95-5000 72 hpi (m.i) | 1 | 39 | UBC18 | 4.654818 | 4.95687 |
| IACSP95-5000 72 hpi (m.i) | 1 | 40 | UBC18 | 4.843462 | 5.146005 |
| IACSP95-5000 72 hpi (m.i) | 2 | 1 | UBC18 | -0.31499 | -0.01114 |
| IACSP95-5000 72 hpi (m.i) | 2 | 2 | UBC18 | -0.31181 | -0.00754 |
| IACSP95-5000 72 hpi (m.i) | 2 | 3 | UBC18 | -0.30676 | -0.00208 |
| IACSP95-5000 72 hpi (m.i) | 2 | 4 | UBC18 | -0.30568 | -0.00058 |
| IACSP95-5000 72 hpi (m.i) | 2 | 5 | UBC18 | -0.3054 | 0.000117 |
| IACSP95-5000 72 hpi (m.i) | 2 | 6 | UBC18 | -0.30387 | 0.002061 |
| IACSP95-5000 72 hpi (m.i) | 2 | 7 | UBC18 | -0.30649 | -0.00014 |
| IACSP95-5000 72 hpi (m.i) | 2 | 8 | UBC18 | -0.30616 | 0.000606 |
| IACSP95-5000 72 hpi (m.i) | 2 | 9 | UBC18 | -0.30542 | 0.001762 |
| IACSP95-5000 72 hpi (m.i) | 2 | 10 | UBC18 | -0.30596 | 0.001641 |
| IACSP95-5000 72 hpi (m.i) | 2 | 11 | UBC18 | -0.3075 | 0.000518 |
| IACSP95-5000 72 hpi (m.i) | 2 | 12 | UBC18 | -0.30798 | 0.000448 |
| IACSP95-5000 72 hpi (m.i) | 2 | 13 | UBC18 | -0.31004 | -0.00119 |
| IACSP95-5000 72 hpi (m.i) | 2 | 14 | UBC18 | -0.30888 | 0.000386 |
| IACSP95-5000 72 hpi (m.i) | 2 | 15 | UBC18 | -0.31055 | -0.00087 |
| IACSP95-5000 72 hpi (m.i) | 2 | 16 | UBC18 | -0.31241 | -0.00231 |
| IACSP95-5000 72 hpi (m.i) | 2 | 17 | UBC18 | -0.31235 | -0.00183 |
| IACSP95-5000 72 hpi (m.i) | 2 | 18 | UBC18 | -0.31149 | -0.00055 |
| IACSP95-5000 72 hpi (m.i) | 2 | 19 | UBC18 | -0.31291 | -0.00156 |
| IACSP95-5000 72 hpi (m.i) | 2 | 20 | UBC18 | -0.31264 | -0.00087 |
| IACSP95-5000 72 hpi (m.i) | 2 | 21 | UBC18 | -0.31111 | 0.001076 |
| IACSP95-5000 72 hpi (m.i) | 2 | 22 | UBC18 | -0.30923 | 0.003366 |
| IACSP95-5000 72 hpi (m.i) | 2 | 23 | UBC18 | -0.30306 | 0.009959 |
| IACSP95-5000 72 hpi (m.i) | 2 | 24 | UBC18 | -0.28829 | 0.025138 |
| IACSP95-5000 72 hpi (m.i) | 2 | 25 | UBC18 | -0.25826 | 0.055587 |
| IACSP95-5000 72 hpi (m.i) | 2 | 26 | UBC18 | -0.20256 | 0.111707 |
| IACSP95-5000 72 hpi (m.i) | 2 | 27 | UBC18 | -0.09438 | 0.220298 |
| IACSP95-5000 72 hpi (m.i) | 2 | 28 | UBC18 | 0.115182 | 0.43028 |
| IACSP95-5000 72 hpi (m.i) | 2 | 29 | UBC18 | 0.480359 | 0.795874 |
| IACSP95-5000 72 hpi (m.i) | 2 | 30 | UBC18 | 1.073993 | 1.389925 |
| IACSP95-5000 72 hpi (m.i) | 2 | 31 | UBC18 | 1.850138 | 2.166487 |
| IACSP95-5000 72 hpi (m.i) | 2 | 32 | UBC18 | 2.618315 | 2.93508 |
| IACSP95-5000 72 hpi (m.i) | 2 | 33 | UBC18 | 3.292366 | 3.609548 |
| IACSP95-5000 72 hpi (m.i) | 2 | 34 | UBC18 | 3.859981 | 4.177579 |
| IACSP95-5000 72 hpi (m.i) | 2 | 35 | UBC18 | 4.346526 | 4.664541 |
| IACSP95-5000 72 hpi (m.i) | 2 | 36 | UBC18 | 4.744395 | 5.062827 |
| IACSP95-5000 72 hpi (m.i) | 2 | 37 | UBC18 | 5.056815 | 5.375663 |
| IACSP95-5000 72 hpi (m.i) | 2 | 38 | UBC18 | 5.349882 | 5.669147 |
| IACSP95-5000 72 hpi (m.i) | 2 | 39 | UBC18 | 5.533236 | 5.852917 |
| IACSP95-5000 72 hpi (m.i) | 2 | 40 | UBC18 | 5.702622 | 6.02272 |
| IACSP95-5000 72 hpi (m.i) | 3 | 1 | UBC18 | -0.37474 | -0.00244 |
| IACSP95-5000 72 hpi (m.i) | 3 | 2 | UBC18 | -0.37267 | -0.00027 |
| IACSP95-5000 72 hpi (m.i) | 3 | 3 | UBC18 | -0.37189 | 0.000613 |
| IACSP95-5000 72 hpi (m.i) | 3 | 4 | UBC18 | -0.36991 | 0.002691 |
| IACSP95-5000 72 hpi (m.i) | 3 | 5 | UBC18 | -0.37303 | -0.00033 |
| IACSP95-5000 72 hpi (m.i) | 3 | 6 | UBC18 | -0.37338 | -0.00058 |
| IACSP95-5000 72 hpi (m.i) | 3 | 7 | UBC18 | -0.3697 | 0.003199 |
| IACSP95-5000 72 hpi (m.i) | 3 | 8 | UBC18 | -0.37217 | 0.000829 |
| IACSP95-5000 72 hpi (m.i) | 3 | 9 | UBC18 | -0.37219 | 0.000912 |
| IACSP95-5000 72 hpi (m.i) | 3 | 10 | UBC18 | -0.37689 | -0.00368 |
| IACSP95-5000 72 hpi (m.i) | 3 | 11 | UBC18 | -0.37326 | 4.15E-05 |
| IACSP95-5000 72 hpi (m.i) | 3 | 12 | UBC18 | -0.37473 | -0.00133 |
| IACSP95-5000 72 hpi (m.i) | 3 | 13 | UBC18 | -0.37529 | -0.00178 |
| IACSP95-5000 72 hpi (m.i) | 3 | 14 | UBC18 | -0.37482 | -0.00121 |
| IACSP95-5000 72 hpi (m.i) | 3 | 15 | UBC18 | -0.37632 | -0.00261 |
| IACSP95-5000 72 hpi (m.i) | 3 | 16 | UBC18 | -0.37488 | -0.00107 |
| IACSP95-5000 72 hpi (m.i) | 3 | 17 | UBC18 | -0.37436 | -0.00045 |
| IACSP95-5000 72 hpi (m.i) | 3 | 18 | UBC18 | -0.37527 | -0.00126 |
| IACSP95-5000 72 hpi (m.i) | 3 | 19 | UBC18 | -0.37648 | -0.00236 |
| IACSP95-5000 72 hpi (m.i) | 3 | 20 | UBC18 | -0.37217 | 0.002044 |
| IACSP95-5000 72 hpi (m.i) | 3 | 21 | UBC18 | -0.3733 | 0.001016 |
| IACSP95-5000 72 hpi (m.i) | 3 | 22 | UBC18 | -0.3691 | 0.005315 |
| IACSP95-5000 72 hpi (m.i) | 3 | 23 | UBC18 | -0.36338 | 0.011133 |
| IACSP95-5000 72 hpi (m.i) | 3 | 24 | UBC18 | -0.34633 | 0.028288 |
| IACSP95-5000 72 hpi (m.i) | 3 | 25 | UBC18 | -0.3206 | 0.054115 |
| IACSP95-5000 72 hpi (m.i) | 3 | 26 | UBC18 | -0.26575 | 0.109068 |
| IACSP95-5000 72 hpi (m.i) | 3 | 27 | UBC18 | -0.15957 | 0.215348 |
| IACSP95-5000 72 hpi (m.i) | 3 | 28 | UBC18 | 0.041714 | 0.416734 |
| IACSP95-5000 72 hpi (m.i) | 3 | 29 | UBC18 | 0.399952 | 0.775073 |
| IACSP95-5000 72 hpi (m.i) | 3 | 30 | UBC18 | 0.995896 | 1.371118 |
| IACSP95-5000 72 hpi (m.i) | 3 | 31 | UBC18 | 1.795274 | 2.170597 |
| IACSP95-5000 72 hpi (m.i) | 3 | 32 | UBC18 | 2.615282 | 2.990705 |
| IACSP95-5000 72 hpi (m.i) | 3 | 33 | UBC18 | 3.342859 | 3.718383 |
| IACSP95-5000 72 hpi (m.i) | 3 | 34 | UBC18 | 3.944728 | 4.320354 |
| IACSP95-5000 72 hpi (m.i) | 3 | 35 | UBC18 | 4.448674 | 4.8244 |
| IACSP95-5000 72 hpi (m.i) | 3 | 36 | UBC18 | 4.844905 | 5.220732 |
| IACSP95-5000 72 hpi (m.i) | 3 | 37 | UBC18 | 5.162704 | 5.538631 |
| IACSP95-5000 72 hpi (m.i) | 3 | 38 | UBC18 | 5.438544 | 5.814572 |
| IACSP95-5000 72 hpi (m.i) | 3 | 39 | UBC18 | 5.653664 | 6.029793 |
| IACSP95-5000 72 hpi (m.i) | 3 | 40 | UBC18 | 5.846056 | 6.222286 |
| IACSP95-5000 72 hpi (s.i) | 1 | 1 | UBC18 | -0.31418 | -0.01143 |
| IACSP95-5000 72 hpi (s.i) | 1 | 2 | UBC18 | -0.30928 | -0.00654 |
| IACSP95-5000 72 hpi (s.i) | 1 | 3 | UBC18 | -0.30555 | -0.00282 |
| IACSP95-5000 72 hpi (s.i) | 1 | 4 | UBC18 | -0.30278 | -5.9E-05 |
| IACSP95-5000 72 hpi (s.i) | 1 | 5 | UBC18 | -0.30278 | -7.1E-05 |
| IACSP95-5000 72 hpi (s.i) | 1 | 6 | UBC18 | -0.30012 | 0.002579 |
| IACSP95-5000 72 hpi (s.i) | 1 | 7 | UBC18 | -0.30109 | 0.001599 |
| IACSP95-5000 72 hpi (s.i) | 1 | 8 | UBC18 | -0.30169 | 0.000986 |
| IACSP95-5000 72 hpi (s.i) | 1 | 9 | UBC18 | -0.30084 | 0.001828 |
| IACSP95-5000 72 hpi (s.i) | 1 | 10 | UBC18 | -0.30159 | 0.001069 |
| IACSP95-5000 72 hpi (s.i) | 1 | 11 | UBC18 | -0.30181 | 0.000834 |
| IACSP95-5000 72 hpi (s.i) | 1 | 12 | UBC18 | -0.30396 | -0.00132 |
| IACSP95-5000 72 hpi (s.i) | 1 | 13 | UBC18 | -0.30306 | -0.00043 |
| IACSP95-5000 72 hpi (s.i) | 1 | 14 | UBC18 | -0.30207 | 0.00054 |
| IACSP95-5000 72 hpi (s.i) | 1 | 15 | UBC18 | -0.30371 | -0.00111 |
| IACSP95-5000 72 hpi (s.i) | 1 | 16 | UBC18 | -0.30478 | -0.00218 |
| IACSP95-5000 72 hpi (s.i) | 1 | 17 | UBC18 | -0.30462 | -0.00204 |
| IACSP95-5000 72 hpi (s.i) | 1 | 18 | UBC18 | -0.30538 | -0.00281 |
| IACSP95-5000 72 hpi (s.i) | 1 | 19 | UBC18 | -0.30326 | -0.0007 |
| IACSP95-5000 72 hpi (s.i) | 1 | 20 | UBC18 | -0.30452 | -0.00197 |
| IACSP95-5000 72 hpi (s.i) | 1 | 21 | UBC18 | -0.30437 | -0.00183 |
| IACSP95-5000 72 hpi (s.i) | 1 | 22 | UBC18 | -0.30063 | 0.001899 |
| IACSP95-5000 72 hpi (s.i) | 1 | 23 | UBC18 | -0.29652 | 0.006003 |
| IACSP95-5000 72 hpi (s.i) | 1 | 24 | UBC18 | -0.2877 | 0.014807 |
| IACSP95-5000 72 hpi (s.i) | 1 | 25 | UBC18 | -0.27136 | 0.031136 |
| IACSP95-5000 72 hpi (s.i) | 1 | 26 | UBC18 | -0.23403 | 0.068461 |
| IACSP95-5000 72 hpi (s.i) | 1 | 27 | UBC18 | -0.16598 | 0.136496 |
| IACSP95-5000 72 hpi (s.i) | 1 | 28 | UBC18 | -0.02962 | 0.272844 |
| IACSP95-5000 72 hpi (s.i) | 1 | 29 | UBC18 | 0.217835 | 0.520292 |
| IACSP95-5000 72 hpi (s.i) | 1 | 30 | UBC18 | 0.645237 | 0.947684 |
| IACSP95-5000 72 hpi (s.i) | 1 | 31 | UBC18 | 1.270255 | 1.572691 |
| IACSP95-5000 72 hpi (s.i) | 1 | 32 | UBC18 | 1.980001 | 2.282427 |
| IACSP95-5000 72 hpi (s.i) | 1 | 33 | UBC18 | 2.639956 | 2.942371 |
| IACSP95-5000 72 hpi (s.i) | 1 | 34 | UBC18 | 3.206788 | 3.509192 |
| IACSP95-5000 72 hpi (s.i) | 1 | 35 | UBC18 | 3.688854 | 3.991249 |
| IACSP95-5000 72 hpi (s.i) | 1 | 36 | UBC18 | 4.092269 | 4.394653 |
| IACSP95-5000 72 hpi (s.i) | 1 | 37 | UBC18 | 4.399633 | 4.702007 |
| IACSP95-5000 72 hpi (s.i) | 1 | 38 | UBC18 | 4.693372 | 4.995735 |
| IACSP95-5000 72 hpi (s.i) | 1 | 39 | UBC18 | 4.909738 | 5.21209 |
| IACSP95-5000 72 hpi (s.i) | 1 | 40 | UBC18 | 5.100621 | 5.402963 |
| IACSP95-5000 72 hpi (s.i) | 2 | 1 | UBC18 | -0.33993 | -0.01312 |
| IACSP95-5000 72 hpi (s.i) | 2 | 2 | UBC18 | -0.33605 | -0.00873 |
| IACSP95-5000 72 hpi (s.i) | 2 | 3 | UBC18 | -0.3335 | -0.00569 |
| IACSP95-5000 72 hpi (s.i) | 2 | 4 | UBC18 | -0.32936 | -0.00105 |
| IACSP95-5000 72 hpi (s.i) | 2 | 5 | UBC18 | -0.33001 | -0.00119 |
| IACSP95-5000 72 hpi (s.i) | 2 | 6 | UBC18 | -0.32755 | 0.001765 |
| IACSP95-5000 72 hpi (s.i) | 2 | 7 | UBC18 | -0.3286 | 0.00121 |
| IACSP95-5000 72 hpi (s.i) | 2 | 8 | UBC18 | -0.32809 | 0.002221 |
| IACSP95-5000 72 hpi (s.i) | 2 | 9 | UBC18 | -0.32845 | 0.002366 |
| IACSP95-5000 72 hpi (s.i) | 2 | 10 | UBC18 | -0.32895 | 0.002365 |
| IACSP95-5000 72 hpi (s.i) | 2 | 11 | UBC18 | -0.32853 | 0.003285 |
| IACSP95-5000 72 hpi (s.i) | 2 | 12 | UBC18 | -0.33214 | 0.000172 |
| IACSP95-5000 72 hpi (s.i) | 2 | 13 | UBC18 | -0.33171 | 0.001105 |
| IACSP95-5000 72 hpi (s.i) | 2 | 14 | UBC18 | -0.33265 | 0.000658 |
| IACSP95-5000 72 hpi (s.i) | 2 | 15 | UBC18 | -0.33472 | -0.00091 |
| IACSP95-5000 72 hpi (s.i) | 2 | 16 | UBC18 | -0.336 | -0.00169 |
| IACSP95-5000 72 hpi (s.i) | 2 | 17 | UBC18 | -0.33604 | -0.00123 |
| IACSP95-5000 72 hpi (s.i) | 2 | 18 | UBC18 | -0.33819 | -0.00288 |
| IACSP95-5000 72 hpi (s.i) | 2 | 19 | UBC18 | -0.3377 | -0.00189 |
| IACSP95-5000 72 hpi (s.i) | 2 | 20 | UBC18 | -0.33718 | -0.00087 |
| IACSP95-5000 72 hpi (s.i) | 2 | 21 | UBC18 | -0.3378 | -0.00099 |
| IACSP95-5000 72 hpi (s.i) | 2 | 22 | UBC18 | -0.33408 | 0.003229 |
| IACSP95-5000 72 hpi (s.i) | 2 | 23 | UBC18 | -0.33013 | 0.007683 |
| IACSP95-5000 72 hpi (s.i) | 2 | 24 | UBC18 | -0.31782 | 0.020485 |
| IACSP95-5000 72 hpi (s.i) | 2 | 25 | UBC18 | -0.29774 | 0.041069 |
| IACSP95-5000 72 hpi (s.i) | 2 | 26 | UBC18 | -0.25629 | 0.083014 |
| IACSP95-5000 72 hpi (s.i) | 2 | 27 | UBC18 | -0.17683 | 0.162975 |
| IACSP95-5000 72 hpi (s.i) | 2 | 28 | UBC18 | -0.01812 | 0.322192 |
| IACSP95-5000 72 hpi (s.i) | 2 | 29 | UBC18 | 0.265559 | 0.606366 |
| IACSP95-5000 72 hpi (s.i) | 2 | 30 | UBC18 | 0.755258 | 1.096566 |
| IACSP95-5000 72 hpi (s.i) | 2 | 31 | UBC18 | 1.46938 | 1.811187 |
| IACSP95-5000 72 hpi (s.i) | 2 | 32 | UBC18 | 2.275017 | 2.617323 |
| IACSP95-5000 72 hpi (s.i) | 2 | 33 | UBC18 | 3.013234 | 3.356041 |
| IACSP95-5000 72 hpi (s.i) | 2 | 34 | UBC18 | 3.637048 | 3.980355 |
| IACSP95-5000 72 hpi (s.i) | 2 | 35 | UBC18 | 4.155303 | 4.499109 |
| IACSP95-5000 72 hpi (s.i) | 2 | 36 | UBC18 | 4.571517 | 4.915823 |
| IACSP95-5000 72 hpi (s.i) | 2 | 37 | UBC18 | 4.900813 | 5.245618 |
| IACSP95-5000 72 hpi (s.i) | 2 | 38 | UBC18 | 5.198759 | 5.544064 |
| IACSP95-5000 72 hpi (s.i) | 2 | 39 | UBC18 | 5.402914 | 5.748719 |
| IACSP95-5000 72 hpi (s.i) | 2 | 40 | UBC18 | 5.586532 | 5.932837 |
| IACSP95-5000 72 hpi (s.i) | 3 | 1 | UBC18 | -0.32971 | -0.01583 |
| IACSP95-5000 72 hpi (s.i) | 3 | 2 | UBC18 | -0.31998 | -0.00633 |
| IACSP95-5000 72 hpi (s.i) | 3 | 3 | UBC18 | -0.3164 | -0.00298 |
| IACSP95-5000 72 hpi (s.i) | 3 | 4 | UBC18 | -0.31269 | 0.000504 |
| IACSP95-5000 72 hpi (s.i) | 3 | 5 | UBC18 | -0.31321 | -0.00024 |
| IACSP95-5000 72 hpi (s.i) | 3 | 6 | UBC18 | -0.31133 | 0.001415 |
| IACSP95-5000 72 hpi (s.i) | 3 | 7 | UBC18 | -0.30982 | 0.002694 |
| IACSP95-5000 72 hpi (s.i) | 3 | 8 | UBC18 | -0.31134 | 0.000941 |
| IACSP95-5000 72 hpi (s.i) | 3 | 9 | UBC18 | -0.31154 | 0.000518 |
| IACSP95-5000 72 hpi (s.i) | 3 | 10 | UBC18 | -0.31067 | 0.001159 |
| IACSP95-5000 72 hpi (s.i) | 3 | 11 | UBC18 | -0.30964 | 0.001963 |
| IACSP95-5000 72 hpi (s.i) | 3 | 12 | UBC18 | -0.31308 | -0.0017 |
| IACSP95-5000 72 hpi (s.i) | 3 | 13 | UBC18 | -0.31253 | -0.00138 |
| IACSP95-5000 72 hpi (s.i) | 3 | 14 | UBC18 | -0.31123 | -0.00031 |
| IACSP95-5000 72 hpi (s.i) | 3 | 15 | UBC18 | -0.31239 | -0.0017 |
| IACSP95-5000 72 hpi (s.i) | 3 | 16 | UBC18 | -0.31244 | -0.00197 |
| IACSP95-5000 72 hpi (s.i) | 3 | 17 | UBC18 | -0.31093 | -0.00069 |
| IACSP95-5000 72 hpi (s.i) | 3 | 18 | UBC18 | -0.31075 | -0.00073 |
| IACSP95-5000 72 hpi (s.i) | 3 | 19 | UBC18 | -0.3109 | -0.00112 |
| IACSP95-5000 72 hpi (s.i) | 3 | 20 | UBC18 | -0.31082 | -0.00127 |
| IACSP95-5000 72 hpi (s.i) | 3 | 21 | UBC18 | -0.30823 | 0.0011 |
| IACSP95-5000 72 hpi (s.i) | 3 | 22 | UBC18 | -0.3053 | 0.003805 |
| IACSP95-5000 72 hpi (s.i) | 3 | 23 | UBC18 | -0.29743 | 0.011445 |
| IACSP95-5000 72 hpi (s.i) | 3 | 24 | UBC18 | -0.28597 | 0.022678 |
| IACSP95-5000 72 hpi (s.i) | 3 | 25 | UBC18 | -0.26144 | 0.046981 |
| IACSP95-5000 72 hpi (s.i) | 3 | 26 | UBC18 | -0.21397 | 0.094224 |
| IACSP95-5000 72 hpi (s.i) | 3 | 27 | UBC18 | -0.11676 | 0.191207 |
| IACSP95-5000 72 hpi (s.i) | 3 | 28 | UBC18 | 0.068736 | 0.376474 |
| IACSP95-5000 72 hpi (s.i) | 3 | 29 | UBC18 | 0.399697 | 0.707207 |
| IACSP95-5000 72 hpi (s.i) | 3 | 30 | UBC18 | 0.959412 | 1.266694 |
| IACSP95-5000 72 hpi (s.i) | 3 | 31 | UBC18 | 1.743163 | 2.050218 |
| IACSP95-5000 72 hpi (s.i) | 3 | 32 | UBC18 | 2.579063 | 2.88589 |
| IACSP95-5000 72 hpi (s.i) | 3 | 33 | UBC18 | 3.323873 | 3.630474 |
| IACSP95-5000 72 hpi (s.i) | 3 | 34 | UBC18 | 3.943352 | 4.249725 |
| IACSP95-5000 72 hpi (s.i) | 3 | 35 | UBC18 | 4.47395 | 4.780096 |
| IACSP95-5000 72 hpi (s.i) | 3 | 36 | UBC18 | 4.898877 | 5.204795 |
| IACSP95-5000 72 hpi (s.i) | 3 | 37 | UBC18 | 5.232717 | 5.538408 |
| IACSP95-5000 72 hpi (s.i) | 3 | 38 | UBC18 | 5.541433 | 5.846897 |
| IACSP95-5000 72 hpi (s.i) | 3 | 39 | UBC18 | 5.747877 | 6.053113 |
| IACSP95-5000 72 hpi (s.i) | 3 | 40 | UBC18 | 5.945229 | 6.250237 |
| IAC91-1099 24 hpi (m.i) | 1 | 1 | UK | -0.14317 | -0.00803 |
| IAC91-1099 24 hpi (m.i) | 1 | 2 | UK | -0.14519 | -0.00832 |
| IAC91-1099 24 hpi (m.i) | 1 | 3 | UK | -0.14228 | -0.00368 |
| IAC91-1099 24 hpi (m.i) | 1 | 4 | UK | -0.14168 | -0.00135 |
| IAC91-1099 24 hpi (m.i) | 1 | 5 | UK | -0.14223 | -0.00017 |
| IAC91-1099 24 hpi (m.i) | 1 | 6 | UK | -0.14096 | 0.002819 |
| IAC91-1099 24 hpi (m.i) | 1 | 7 | UK | -0.14258 | 0.002926 |
| IAC91-1099 24 hpi (m.i) | 1 | 8 | UK | -0.14488 | 0.002357 |
| IAC91-1099 24 hpi (m.i) | 1 | 9 | UK | -0.14737 | 0.001596 |
| IAC91-1099 24 hpi (m.i) | 1 | 10 | UK | -0.15019 | 0.000499 |
| IAC91-1099 24 hpi (m.i) | 1 | 11 | UK | -0.15217 | 0.000252 |
| IAC91-1099 24 hpi (m.i) | 1 | 12 | UK | -0.1539 | 0.000251 |
| IAC91-1099 24 hpi (m.i) | 1 | 13 | UK | -0.15604 | -0.00016 |
| IAC91-1099 24 hpi (m.i) | 1 | 14 | UK | -0.15749 | 0.000113 |
| IAC91-1099 24 hpi (m.i) | 1 | 15 | UK | -0.16159 | -0.00226 |
| IAC91-1099 24 hpi (m.i) | 1 | 16 | UK | -0.16256 | -0.0015 |
| IAC91-1099 24 hpi (m.i) | 1 | 17 | UK | -0.16585 | -0.00306 |
| IAC91-1099 24 hpi (m.i) | 1 | 18 | UK | -0.16589 | -0.00137 |
| IAC91-1099 24 hpi (m.i) | 1 | 19 | UK | -0.16929 | -0.00305 |
| IAC91-1099 24 hpi (m.i) | 1 | 20 | UK | -0.16934 | -0.00138 |
| IAC91-1099 24 hpi (m.i) | 1 | 21 | UK | -0.1685 | 0.001196 |
| IAC91-1099 24 hpi (m.i) | 1 | 22 | UK | -0.16545 | 0.005977 |
| IAC91-1099 24 hpi (m.i) | 1 | 23 | UK | -0.157 | 0.016156 |
| IAC91-1099 24 hpi (m.i) | 1 | 24 | UK | -0.1389 | 0.035977 |
| IAC91-1099 24 hpi (m.i) | 1 | 25 | UK | -0.10081 | 0.075795 |
| IAC91-1099 24 hpi (m.i) | 1 | 26 | UK | -0.02623 | 0.152105 |
| IAC91-1099 24 hpi (m.i) | 1 | 27 | UK | 0.112707 | 0.292769 |
| IAC91-1099 24 hpi (m.i) | 1 | 28 | UK | 0.375547 | 0.557336 |
| IAC91-1099 24 hpi (m.i) | 1 | 29 | UK | 0.804417 | 0.987934 |
| IAC91-1099 24 hpi (m.i) | 1 | 30 | UK | 1.418166 | 1.60341 |
| IAC91-1099 24 hpi (m.i) | 1 | 31 | UK | 2.09939 | 2.286362 |
| IAC91-1099 24 hpi (m.i) | 1 | 32 | UK | 2.750748 | 2.939447 |
| IAC91-1099 24 hpi (m.i) | 1 | 33 | UK | 3.359882 | 3.550309 |
| IAC91-1099 24 hpi (m.i) | 1 | 34 | UK | 3.886337 | 4.078491 |
| IAC91-1099 24 hpi (m.i) | 1 | 35 | UK | 4.366326 | 4.560208 |
| IAC91-1099 24 hpi (m.i) | 1 | 36 | UK | 4.753543 | 4.949152 |
| IAC91-1099 24 hpi (m.i) | 1 | 37 | UK | 5.094803 | 5.29214 |
| IAC91-1099 24 hpi (m.i) | 1 | 38 | UK | 5.391255 | 5.59032 |
| IAC91-1099 24 hpi (m.i) | 1 | 39 | UK | 5.616921 | 5.817714 |
| IAC91-1099 24 hpi (m.i) | 1 | 40 | UK | 5.820949 | 6.023468 |
| IAC91-1099 24 hpi (m.i) | 2 | 1 | UK | -0.0548 | 0.002593 |
| IAC91-1099 24 hpi (m.i) | 2 | 2 | UK | -0.05589 | 0.003079 |
| IAC91-1099 24 hpi (m.i) | 2 | 3 | UK | -0.05686 | 0.003686 |
| IAC91-1099 24 hpi (m.i) | 2 | 4 | UK | -0.05762 | 0.004495 |
| IAC91-1099 24 hpi (m.i) | 2 | 5 | UK | -0.06056 | 0.003128 |
| IAC91-1099 24 hpi (m.i) | 2 | 6 | UK | -0.0624 | 0.00287 |
| IAC91-1099 24 hpi (m.i) | 2 | 7 | UK | -0.06558 | 0.001256 |
| IAC91-1099 24 hpi (m.i) | 2 | 8 | UK | -0.06941 | -0.001 |
| IAC91-1099 24 hpi (m.i) | 2 | 9 | UK | -0.0712 | -0.00122 |
| IAC91-1099 24 hpi (m.i) | 2 | 10 | UK | -0.07483 | -0.00327 |
| IAC91-1099 24 hpi (m.i) | 2 | 11 | UK | -0.07687 | -0.00373 |
| IAC91-1099 24 hpi (m.i) | 2 | 12 | UK | -0.07662 | -0.00191 |
| IAC91-1099 24 hpi (m.i) | 2 | 13 | UK | -0.07898 | -0.0027 |
| IAC91-1099 24 hpi (m.i) | 2 | 14 | UK | -0.08186 | -0.004 |
| IAC91-1099 24 hpi (m.i) | 2 | 15 | UK | -0.0843 | -0.00486 |
| IAC91-1099 24 hpi (m.i) | 2 | 16 | UK | -0.08518 | -0.00417 |
| IAC91-1099 24 hpi (m.i) | 2 | 17 | UK | -0.08672 | -0.00414 |
| IAC91-1099 24 hpi (m.i) | 2 | 18 | UK | -0.08591 | -0.00175 |
| IAC91-1099 24 hpi (m.i) | 2 | 19 | UK | -0.0873 | -0.00157 |
| IAC91-1099 24 hpi (m.i) | 2 | 20 | UK | -0.08533 | 0.001983 |
| IAC91-1099 24 hpi (m.i) | 2 | 21 | UK | -0.08493 | 0.003952 |
| IAC91-1099 24 hpi (m.i) | 2 | 22 | UK | -0.07752 | 0.012933 |
| IAC91-1099 24 hpi (m.i) | 2 | 23 | UK | -0.06756 | 0.024472 |
| IAC91-1099 24 hpi (m.i) | 2 | 24 | UK | -0.03768 | 0.055931 |
| IAC91-1099 24 hpi (m.i) | 2 | 25 | UK | 0.012805 | 0.107986 |
| IAC91-1099 24 hpi (m.i) | 2 | 26 | UK | 0.111919 | 0.208674 |
| IAC91-1099 24 hpi (m.i) | 2 | 27 | UK | 0.296443 | 0.394772 |
| IAC91-1099 24 hpi (m.i) | 2 | 28 | UK | 0.64427 | 0.744174 |
| IAC91-1099 24 hpi (m.i) | 2 | 29 | UK | 1.191918 | 1.293396 |
| IAC91-1099 24 hpi (m.i) | 2 | 30 | UK | 1.933947 | 2.037 |
| IAC91-1099 24 hpi (m.i) | 2 | 31 | UK | 2.710864 | 2.815491 |
| IAC91-1099 24 hpi (m.i) | 2 | 32 | UK | 3.41589 | 3.522092 |
| IAC91-1099 24 hpi (m.i) | 2 | 33 | UK | 4.059761 | 4.167537 |
| IAC91-1099 24 hpi (m.i) | 2 | 34 | UK | 4.591444 | 4.700795 |
| IAC91-1099 24 hpi (m.i) | 2 | 35 | UK | 5.058043 | 5.168968 |
| IAC91-1099 24 hpi (m.i) | 2 | 36 | UK | 5.445798 | 5.558298 |
| IAC91-1099 24 hpi (m.i) | 2 | 37 | UK | 5.772064 | 5.886138 |
| IAC91-1099 24 hpi (m.i) | 2 | 38 | UK | 6.039808 | 6.155457 |
| IAC91-1099 24 hpi (m.i) | 2 | 39 | UK | 6.252047 | 6.36927 |
| IAC91-1099 24 hpi (m.i) | 2 | 40 | UK | 6.441527 | 6.560324 |
| IAC91-1099 24 hpi (m.i) | 3 | 1 | UK | -0.0495 | -0.00065 |
| IAC91-1099 24 hpi (m.i) | 3 | 2 | UK | -0.05296 | -0.00288 |
| IAC91-1099 24 hpi (m.i) | 3 | 3 | UK | -0.05015 | 0.001164 |
| IAC91-1099 24 hpi (m.i) | 3 | 4 | UK | -0.05105 | 0.001503 |
| IAC91-1099 24 hpi (m.i) | 3 | 5 | UK | -0.05088 | 0.002904 |
| IAC91-1099 24 hpi (m.i) | 3 | 6 | UK | -0.05294 | 0.002083 |
| IAC91-1099 24 hpi (m.i) | 3 | 7 | UK | -0.05584 | 0.000414 |
| IAC91-1099 24 hpi (m.i) | 3 | 8 | UK | -0.05599 | 0.001497 |
| IAC91-1099 24 hpi (m.i) | 3 | 9 | UK | -0.05921 | -0.00049 |
| IAC91-1099 24 hpi (m.i) | 3 | 10 | UK | -0.06349 | -0.00353 |
| IAC91-1099 24 hpi (m.i) | 3 | 11 | UK | -0.06311 | -0.00191 |
| IAC91-1099 24 hpi (m.i) | 3 | 12 | UK | -0.06654 | -0.00411 |
| IAC91-1099 24 hpi (m.i) | 3 | 13 | UK | -0.06777 | -0.00411 |
| IAC91-1099 24 hpi (m.i) | 3 | 14 | UK | -0.06443 | 0.000467 |
| IAC91-1099 24 hpi (m.i) | 3 | 15 | UK | -0.067 | -0.00087 |
| IAC91-1099 24 hpi (m.i) | 3 | 16 | UK | -0.06988 | -0.00251 |
| IAC91-1099 24 hpi (m.i) | 3 | 17 | UK | -0.06913 | -0.00052 |
| IAC91-1099 24 hpi (m.i) | 3 | 18 | UK | -0.0694 | 0.000445 |
| IAC91-1099 24 hpi (m.i) | 3 | 19 | UK | -0.07251 | -0.00143 |
| IAC91-1099 24 hpi (m.i) | 3 | 20 | UK | -0.07001 | 0.002296 |
| IAC91-1099 24 hpi (m.i) | 3 | 21 | UK | -0.06684 | 0.006707 |
| IAC91-1099 24 hpi (m.i) | 3 | 22 | UK | -0.06292 | 0.011863 |
| IAC91-1099 24 hpi (m.i) | 3 | 23 | UK | -0.04836 | 0.027659 |
| IAC91-1099 24 hpi (m.i) | 3 | 24 | UK | -0.02456 | 0.052695 |
| IAC91-1099 24 hpi (m.i) | 3 | 25 | UK | 0.027914 | 0.106399 |
| IAC91-1099 24 hpi (m.i) | 3 | 26 | UK | 0.12876 | 0.20848 |
| IAC91-1099 24 hpi (m.i) | 3 | 27 | UK | 0.314775 | 0.39573 |
| IAC91-1099 24 hpi (m.i) | 3 | 28 | UK | 0.653826 | 0.736016 |
| IAC91-1099 24 hpi (m.i) | 3 | 29 | UK | 1.200419 | 1.283844 |
| IAC91-1099 24 hpi (m.i) | 3 | 30 | UK | 1.952958 | 2.037618 |
| IAC91-1099 24 hpi (m.i) | 3 | 31 | UK | 2.741863 | 2.827758 |
| IAC91-1099 24 hpi (m.i) | 3 | 32 | UK | 3.45875 | 3.54588 |
| IAC91-1099 24 hpi (m.i) | 3 | 33 | UK | 4.113074 | 4.201439 |
| IAC91-1099 24 hpi (m.i) | 3 | 34 | UK | 4.67714 | 4.76674 |
| IAC91-1099 24 hpi (m.i) | 3 | 35 | UK | 5.177524 | 5.268359 |
| IAC91-1099 24 hpi (m.i) | 3 | 36 | UK | 5.564455 | 5.656525 |
| IAC91-1099 24 hpi (m.i) | 3 | 37 | UK | 5.88438 | 5.977685 |
| IAC91-1099 24 hpi (m.i) | 3 | 38 | UK | 6.207006 | 6.301546 |
| IAC91-1099 24 hpi (m.i) | 3 | 39 | UK | 6.437106 | 6.532881 |
| IAC91-1099 24 hpi (m.i) | 3 | 40 | UK | 6.625518 | 6.722528 |
| IAC91-1099 24 hpi (s.i) | 1 | 1 | UK | -0.26491 | -0.01648 |
| IAC91-1099 24 hpi (s.i) | 1 | 2 | UK | -0.25877 | -0.01024 |
| IAC91-1099 24 hpi (s.i) | 1 | 3 | UK | -0.25561 | -0.00699 |
| IAC91-1099 24 hpi (s.i) | 1 | 4 | UK | -0.24852 | 0.000203 |
| IAC91-1099 24 hpi (s.i) | 1 | 5 | UK | -0.24718 | 0.001636 |
| IAC91-1099 24 hpi (s.i) | 1 | 6 | UK | -0.24495 | 0.003961 |
| IAC91-1099 24 hpi (s.i) | 1 | 7 | UK | -0.24868 | 0.000325 |
| IAC91-1099 24 hpi (s.i) | 1 | 8 | UK | -0.24616 | 0.002935 |
| IAC91-1099 24 hpi (s.i) | 1 | 9 | UK | -0.24779 | 0.001405 |
| IAC91-1099 24 hpi (s.i) | 1 | 10 | UK | -0.25018 | -0.0009 |
| IAC91-1099 24 hpi (s.i) | 1 | 11 | UK | -0.24882 | 0.000557 |
| IAC91-1099 24 hpi (s.i) | 1 | 12 | UK | -0.24715 | 0.002325 |
| IAC91-1099 24 hpi (s.i) | 1 | 13 | UK | -0.25032 | -0.00075 |
| IAC91-1099 24 hpi (s.i) | 1 | 14 | UK | -0.24681 | 0.002858 |
| IAC91-1099 24 hpi (s.i) | 1 | 15 | UK | -0.25193 | -0.00217 |
| IAC91-1099 24 hpi (s.i) | 1 | 16 | UK | -0.25163 | -0.00178 |
| IAC91-1099 24 hpi (s.i) | 1 | 17 | UK | -0.24964 | 0.00031 |
| IAC91-1099 24 hpi (s.i) | 1 | 18 | UK | -0.25288 | -0.00284 |
| IAC91-1099 24 hpi (s.i) | 1 | 19 | UK | -0.25456 | -0.00443 |
| IAC91-1099 24 hpi (s.i) | 1 | 20 | UK | -0.25197 | -0.00174 |
| IAC91-1099 24 hpi (s.i) | 1 | 21 | UK | -0.2514 | -0.00108 |
| IAC91-1099 24 hpi (s.i) | 1 | 22 | UK | -0.25168 | -0.00126 |
| IAC91-1099 24 hpi (s.i) | 1 | 23 | UK | -0.24309 | 0.00742 |
| IAC91-1099 24 hpi (s.i) | 1 | 24 | UK | -0.2356 | 0.015007 |
| IAC91-1099 24 hpi (s.i) | 1 | 25 | UK | -0.2137 | 0.037001 |
| IAC91-1099 24 hpi (s.i) | 1 | 26 | UK | -0.16942 | 0.081379 |
| IAC91-1099 24 hpi (s.i) | 1 | 27 | UK | -0.09301 | 0.157878 |
| IAC91-1099 24 hpi (s.i) | 1 | 28 | UK | 0.058199 | 0.309184 |
| IAC91-1099 24 hpi (s.i) | 1 | 29 | UK | 0.326611 | 0.577691 |
| IAC91-1099 24 hpi (s.i) | 1 | 30 | UK | 0.770368 | 1.021542 |
| IAC91-1099 24 hpi (s.i) | 1 | 31 | UK | 1.376783 | 1.628052 |
| IAC91-1099 24 hpi (s.i) | 1 | 32 | UK | 2.014369 | 2.265732 |
| IAC91-1099 24 hpi (s.i) | 1 | 33 | UK | 2.644593 | 2.89605 |
| IAC91-1099 24 hpi (s.i) | 1 | 34 | UK | 3.23297 | 3.484522 |
| IAC91-1099 24 hpi (s.i) | 1 | 35 | UK | 3.761153 | 4.012799 |
| IAC91-1099 24 hpi (s.i) | 1 | 36 | UK | 4.218435 | 4.470176 |
| IAC91-1099 24 hpi (s.i) | 1 | 37 | UK | 4.591262 | 4.843097 |
| IAC91-1099 24 hpi (s.i) | 1 | 38 | UK | 4.952375 | 5.204305 |
| IAC91-1099 24 hpi (s.i) | 1 | 39 | UK | 5.213292 | 5.465316 |
| IAC91-1099 24 hpi (s.i) | 1 | 40 | UK | 5.466942 | 5.719061 |
| IAC91-1099 24 hpi (s.i) | 2 | 1 | UK | -0.31845 | -0.00673 |
| IAC91-1099 24 hpi (s.i) | 2 | 2 | UK | -0.31604 | -0.00406 |
| IAC91-1099 24 hpi (s.i) | 2 | 3 | UK | -0.31191 | 0.000339 |
| IAC91-1099 24 hpi (s.i) | 2 | 4 | UK | -0.30989 | 0.002622 |
| IAC91-1099 24 hpi (s.i) | 2 | 5 | UK | -0.31085 | 0.001922 |
| IAC91-1099 24 hpi (s.i) | 2 | 6 | UK | -0.31091 | 0.002123 |
| IAC91-1099 24 hpi (s.i) | 2 | 7 | UK | -0.31052 | 0.002778 |
| IAC91-1099 24 hpi (s.i) | 2 | 8 | UK | -0.31295 | 0.000606 |
| IAC91-1099 24 hpi (s.i) | 2 | 9 | UK | -0.31187 | 0.001951 |
| IAC91-1099 24 hpi (s.i) | 2 | 10 | UK | -0.31294 | 0.001138 |
| IAC91-1099 24 hpi (s.i) | 2 | 11 | UK | -0.31546 | -0.00111 |
| IAC91-1099 24 hpi (s.i) | 2 | 12 | UK | -0.31897 | -0.00436 |
| IAC91-1099 24 hpi (s.i) | 2 | 13 | UK | -0.31774 | -0.00287 |
| IAC91-1099 24 hpi (s.i) | 2 | 14 | UK | -0.32054 | -0.00541 |
| IAC91-1099 24 hpi (s.i) | 2 | 15 | UK | -0.32076 | -0.00537 |
| IAC91-1099 24 hpi (s.i) | 2 | 16 | UK | -0.31911 | -0.00346 |
| IAC91-1099 24 hpi (s.i) | 2 | 17 | UK | -0.3201 | -0.00419 |
| IAC91-1099 24 hpi (s.i) | 2 | 18 | UK | -0.31758 | -0.0014 |
| IAC91-1099 24 hpi (s.i) | 2 | 19 | UK | -0.31749 | -0.00105 |
| IAC91-1099 24 hpi (s.i) | 2 | 20 | UK | -0.31649 | 0.000215 |
| IAC91-1099 24 hpi (s.i) | 2 | 21 | UK | -0.31383 | 0.003131 |
| IAC91-1099 24 hpi (s.i) | 2 | 22 | UK | -0.30483 | 0.012397 |
| IAC91-1099 24 hpi (s.i) | 2 | 23 | UK | -0.29531 | 0.022181 |
| IAC91-1099 24 hpi (s.i) | 2 | 24 | UK | -0.26862 | 0.049127 |
| IAC91-1099 24 hpi (s.i) | 2 | 25 | UK | -0.22258 | 0.095432 |
| IAC91-1099 24 hpi (s.i) | 2 | 26 | UK | -0.13627 | 0.182002 |
| IAC91-1099 24 hpi (s.i) | 2 | 27 | UK | 0.029201 | 0.347736 |
| IAC91-1099 24 hpi (s.i) | 2 | 28 | UK | 0.344865 | 0.663662 |
| IAC91-1099 24 hpi (s.i) | 2 | 29 | UK | 0.865768 | 1.184827 |
| IAC91-1099 24 hpi (s.i) | 2 | 30 | UK | 1.63202 | 1.951342 |
| IAC91-1099 24 hpi (s.i) | 2 | 31 | UK | 2.490587 | 2.810169 |
| IAC91-1099 24 hpi (s.i) | 2 | 32 | UK | 3.271136 | 3.590981 |
| IAC91-1099 24 hpi (s.i) | 2 | 33 | UK | 3.988492 | 4.308599 |
| IAC91-1099 24 hpi (s.i) | 2 | 34 | UK | 4.595418 | 4.915787 |
| IAC91-1099 24 hpi (s.i) | 2 | 35 | UK | 5.138819 | 5.45945 |
| IAC91-1099 24 hpi (s.i) | 2 | 36 | UK | 5.568032 | 5.888925 |
| IAC91-1099 24 hpi (s.i) | 2 | 37 | UK | 5.926073 | 6.247227 |
| IAC91-1099 24 hpi (s.i) | 2 | 38 | UK | 6.25254 | 6.573957 |
| IAC91-1099 24 hpi (s.i) | 2 | 39 | UK | 6.488286 | 6.809965 |
| IAC91-1099 24 hpi (s.i) | 2 | 40 | UK | 6.710462 | 7.032403 |
| IAC91-1099 24 hpi (s.i) | 3 | 1 | UK | -0.27887 | -0.00991 |
| IAC91-1099 24 hpi (s.i) | 3 | 2 | UK | -0.27504 | -0.00606 |
| IAC91-1099 24 hpi (s.i) | 3 | 3 | UK | -0.27 | -0.00099 |
| IAC91-1099 24 hpi (s.i) | 3 | 4 | UK | -0.26857 | 0.000457 |
| IAC91-1099 24 hpi (s.i) | 3 | 5 | UK | -0.26908 | -2.7E-05 |
| IAC91-1099 24 hpi (s.i) | 3 | 6 | UK | -0.26893 | 0.000137 |
| IAC91-1099 24 hpi (s.i) | 3 | 7 | UK | -0.26551 | 0.003577 |
| IAC91-1099 24 hpi (s.i) | 3 | 8 | UK | -0.26893 | 0.000186 |
| IAC91-1099 24 hpi (s.i) | 3 | 9 | UK | -0.26577 | 0.003368 |
| IAC91-1099 24 hpi (s.i) | 3 | 10 | UK | -0.26671 | 0.00245 |
| IAC91-1099 24 hpi (s.i) | 3 | 11 | UK | -0.26892 | 0.000258 |
| IAC91-1099 24 hpi (s.i) | 3 | 12 | UK | -0.27315 | -0.00395 |
| IAC91-1099 24 hpi (s.i) | 3 | 13 | UK | -0.27091 | -0.00169 |
| IAC91-1099 24 hpi (s.i) | 3 | 14 | UK | -0.27315 | -0.0039 |
| IAC91-1099 24 hpi (s.i) | 3 | 15 | UK | -0.27102 | -0.00176 |
| IAC91-1099 24 hpi (s.i) | 3 | 16 | UK | -0.27012 | -0.00083 |
| IAC91-1099 24 hpi (s.i) | 3 | 17 | UK | -0.2723 | -0.003 |
| IAC91-1099 24 hpi (s.i) | 3 | 18 | UK | -0.26901 | 0.000316 |
| IAC91-1099 24 hpi (s.i) | 3 | 19 | UK | -0.26989 | -0.00054 |
| IAC91-1099 24 hpi (s.i) | 3 | 20 | UK | -0.27155 | -0.00218 |
| IAC91-1099 24 hpi (s.i) | 3 | 21 | UK | -0.26975 | -0.00036 |
| IAC91-1099 24 hpi (s.i) | 3 | 22 | UK | -0.26093 | 0.008483 |
| IAC91-1099 24 hpi (s.i) | 3 | 23 | UK | -0.25491 | 0.014529 |
| IAC91-1099 24 hpi (s.i) | 3 | 24 | UK | -0.23308 | 0.036381 |
| IAC91-1099 24 hpi (s.i) | 3 | 25 | UK | -0.1984 | 0.071076 |
| IAC91-1099 24 hpi (s.i) | 3 | 26 | UK | -0.13148 | 0.138016 |
| IAC91-1099 24 hpi (s.i) | 3 | 27 | UK | -0.00266 | 0.266863 |
| IAC91-1099 24 hpi (s.i) | 3 | 28 | UK | 0.250793 | 0.520336 |
| IAC91-1099 24 hpi (s.i) | 3 | 29 | UK | 0.679753 | 0.949318 |
| IAC91-1099 24 hpi (s.i) | 3 | 30 | UK | 1.355531 | 1.625117 |
| IAC91-1099 24 hpi (s.i) | 3 | 31 | UK | 2.191303 | 2.460911 |
| IAC91-1099 24 hpi (s.i) | 3 | 32 | UK | 2.985958 | 3.255588 |
| IAC91-1099 24 hpi (s.i) | 3 | 33 | UK | 3.713834 | 3.983485 |
| IAC91-1099 24 hpi (s.i) | 3 | 34 | UK | 4.354705 | 4.624378 |
| IAC91-1099 24 hpi (s.i) | 3 | 35 | UK | 4.913186 | 5.182879 |
| IAC91-1099 24 hpi (s.i) | 3 | 36 | UK | 5.370465 | 5.640181 |
| IAC91-1099 24 hpi (s.i) | 3 | 37 | UK | 5.740789 | 6.010527 |
| IAC91-1099 24 hpi (s.i) | 3 | 38 | UK | 6.085498 | 6.355257 |
| IAC91-1099 24 hpi (s.i) | 3 | 39 | UK | 6.33237 | 6.60215 |
| IAC91-1099 24 hpi (s.i) | 3 | 40 | UK | 6.561966 | 6.831768 |
| IAC91-1099 72 hpi (m.i) | 1 | 1 | UK | -0.31029 | -0.00647 |
| IAC91-1099 72 hpi (m.i) | 1 | 2 | UK | -0.31046 | -0.00655 |
| IAC91-1099 72 hpi (m.i) | 1 | 3 | UK | -0.30437 | -0.00037 |
| IAC91-1099 72 hpi (m.i) | 1 | 4 | UK | -0.30363 | 0.000451 |
| IAC91-1099 72 hpi (m.i) | 1 | 5 | UK | -0.30453 | -0.00035 |
| IAC91-1099 72 hpi (m.i) | 1 | 6 | UK | -0.30378 | 0.000486 |
| IAC91-1099 72 hpi (m.i) | 1 | 7 | UK | -0.30427 | 7.73E-05 |
| IAC91-1099 72 hpi (m.i) | 1 | 8 | UK | -0.3041 | 0.000338 |
| IAC91-1099 72 hpi (m.i) | 1 | 9 | UK | -0.30261 | 0.001921 |
| IAC91-1099 72 hpi (m.i) | 1 | 10 | UK | -0.30417 | 0.000444 |
| IAC91-1099 72 hpi (m.i) | 1 | 11 | UK | -0.30514 | -0.00044 |
| IAC91-1099 72 hpi (m.i) | 1 | 12 | UK | -0.30548 | -0.00068 |
| IAC91-1099 72 hpi (m.i) | 1 | 13 | UK | -0.30478 | 0.000105 |
| IAC91-1099 72 hpi (m.i) | 1 | 14 | UK | -0.30519 | -0.00022 |
| IAC91-1099 72 hpi (m.i) | 1 | 15 | UK | -0.3076 | -0.00254 |
| IAC91-1099 72 hpi (m.i) | 1 | 16 | UK | -0.30577 | -0.00063 |
| IAC91-1099 72 hpi (m.i) | 1 | 17 | UK | -0.30634 | -0.0011 |
| IAC91-1099 72 hpi (m.i) | 1 | 18 | UK | -0.30577 | -0.00044 |
| IAC91-1099 72 hpi (m.i) | 1 | 19 | UK | -0.30595 | -0.00054 |
| IAC91-1099 72 hpi (m.i) | 1 | 20 | UK | -0.30499 | 0.000512 |
| IAC91-1099 72 hpi (m.i) | 1 | 21 | UK | -0.30259 | 0.002997 |
| IAC91-1099 72 hpi (m.i) | 1 | 22 | UK | -0.29618 | 0.009501 |
| IAC91-1099 72 hpi (m.i) | 1 | 23 | UK | -0.28315 | 0.022616 |
| IAC91-1099 72 hpi (m.i) | 1 | 24 | UK | -0.2625 | 0.043349 |
| IAC91-1099 72 hpi (m.i) | 1 | 25 | UK | -0.21689 | 0.089052 |
| IAC91-1099 72 hpi (m.i) | 1 | 26 | UK | -0.13203 | 0.173998 |
| IAC91-1099 72 hpi (m.i) | 1 | 27 | UK | 0.030736 | 0.336855 |
| IAC91-1099 72 hpi (m.i) | 1 | 28 | UK | 0.334704 | 0.640911 |
| IAC91-1099 72 hpi (m.i) | 1 | 29 | UK | 0.840773 | 1.147068 |
| IAC91-1099 72 hpi (m.i) | 1 | 30 | UK | 1.584329 | 1.890712 |
| IAC91-1099 72 hpi (m.i) | 1 | 31 | UK | 2.41589 | 2.722362 |
| IAC91-1099 72 hpi (m.i) | 1 | 32 | UK | 3.169816 | 3.476377 |
| IAC91-1099 72 hpi (m.i) | 1 | 33 | UK | 3.860267 | 4.166916 |
| IAC91-1099 72 hpi (m.i) | 1 | 34 | UK | 4.460546 | 4.767283 |
| IAC91-1099 72 hpi (m.i) | 1 | 35 | UK | 4.988993 | 5.295818 |
| IAC91-1099 72 hpi (m.i) | 1 | 36 | UK | 5.421716 | 5.72863 |
| IAC91-1099 72 hpi (m.i) | 1 | 37 | UK | 5.762239 | 6.069242 |
| IAC91-1099 72 hpi (m.i) | 1 | 38 | UK | 6.093606 | 6.400697 |
| IAC91-1099 72 hpi (m.i) | 1 | 39 | UK | 6.320493 | 6.627672 |
| IAC91-1099 72 hpi (m.i) | 1 | 40 | UK | 6.531766 | 6.839034 |
| IAC91-1099 72 hpi (m.i) | 2 | 1 | UK | -0.35647 | -0.00851 |
| IAC91-1099 72 hpi (m.i) | 2 | 2 | UK | -0.35408 | -0.00565 |
| IAC91-1099 72 hpi (m.i) | 2 | 3 | UK | -0.35306 | -0.00417 |
| IAC91-1099 72 hpi (m.i) | 2 | 4 | UK | -0.34912 | 0.000231 |
| IAC91-1099 72 hpi (m.i) | 2 | 5 | UK | -0.35106 | -0.00125 |
| IAC91-1099 72 hpi (m.i) | 2 | 6 | UK | -0.34747 | 0.002808 |
| IAC91-1099 72 hpi (m.i) | 2 | 7 | UK | -0.34789 | 0.002847 |
| IAC91-1099 72 hpi (m.i) | 2 | 8 | UK | -0.34844 | 0.002756 |
| IAC91-1099 72 hpi (m.i) | 2 | 9 | UK | -0.34982 | 0.00184 |
| IAC91-1099 72 hpi (m.i) | 2 | 10 | UK | -0.35155 | 0.000574 |
| IAC91-1099 72 hpi (m.i) | 2 | 11 | UK | -0.35151 | 0.00108 |
| IAC91-1099 72 hpi (m.i) | 2 | 12 | UK | -0.35311 | -5.4E-05 |
| IAC91-1099 72 hpi (m.i) | 2 | 13 | UK | -0.35453 | -0.00101 |
| IAC91-1099 72 hpi (m.i) | 2 | 14 | UK | -0.35562 | -0.00164 |
| IAC91-1099 72 hpi (m.i) | 2 | 15 | UK | -0.35715 | -0.00271 |
| IAC91-1099 72 hpi (m.i) | 2 | 16 | UK | -0.35727 | -0.00236 |
| IAC91-1099 72 hpi (m.i) | 2 | 17 | UK | -0.35845 | -0.00308 |
| IAC91-1099 72 hpi (m.i) | 2 | 18 | UK | -0.35731 | -0.00148 |
| IAC91-1099 72 hpi (m.i) | 2 | 19 | UK | -0.35758 | -0.00129 |
| IAC91-1099 72 hpi (m.i) | 2 | 20 | UK | -0.3552 | 0.001558 |
| IAC91-1099 72 hpi (m.i) | 2 | 21 | UK | -0.35187 | 0.005351 |
| IAC91-1099 72 hpi (m.i) | 2 | 22 | UK | -0.34628 | 0.011407 |
| IAC91-1099 72 hpi (m.i) | 2 | 23 | UK | -0.33014 | 0.028009 |
| IAC91-1099 72 hpi (m.i) | 2 | 24 | UK | -0.30074 | 0.057872 |
| IAC91-1099 72 hpi (m.i) | 2 | 25 | UK | -0.24461 | 0.114457 |
| IAC91-1099 72 hpi (m.i) | 2 | 26 | UK | -0.13445 | 0.225089 |
| IAC91-1099 72 hpi (m.i) | 2 | 27 | UK | 0.066609 | 0.426607 |
| IAC91-1099 72 hpi (m.i) | 2 | 28 | UK | 0.439875 | 0.800336 |
| IAC91-1099 72 hpi (m.i) | 2 | 29 | UK | 1.046516 | 1.407441 |
| IAC91-1099 72 hpi (m.i) | 2 | 30 | UK | 1.892213 | 2.253601 |
| IAC91-1099 72 hpi (m.i) | 2 | 31 | UK | 2.778465 | 3.140316 |
| IAC91-1099 72 hpi (m.i) | 2 | 32 | UK | 3.548586 | 3.910899 |
| IAC91-1099 72 hpi (m.i) | 2 | 33 | UK | 4.229705 | 4.592482 |
| IAC91-1099 72 hpi (m.i) | 2 | 34 | UK | 4.79876 | 5.162 |
| IAC91-1099 72 hpi (m.i) | 2 | 35 | UK | 5.29055 | 5.654253 |
| IAC91-1099 72 hpi (m.i) | 2 | 36 | UK | 5.692361 | 6.056527 |
| IAC91-1099 72 hpi (m.i) | 2 | 37 | UK | 6.014894 | 6.379524 |
| IAC91-1099 72 hpi (m.i) | 2 | 38 | UK | 6.312848 | 6.67794 |
| IAC91-1099 72 hpi (m.i) | 2 | 39 | UK | 6.523992 | 6.889547 |
| IAC91-1099 72 hpi (m.i) | 2 | 40 | UK | 6.707454 | 7.073472 |
| IAC91-1099 72 hpi (m.i) | 3 | 1 | UK | -0.33864 | -0.01378 |
| IAC91-1099 72 hpi (m.i) | 3 | 2 | UK | -0.33385 | -0.00855 |
| IAC91-1099 72 hpi (m.i) | 3 | 3 | UK | -0.32823 | -0.00249 |
| IAC91-1099 72 hpi (m.i) | 3 | 4 | UK | -0.32651 | -0.00033 |
| IAC91-1099 72 hpi (m.i) | 3 | 5 | UK | -0.32668 | -5.3E-05 |
| IAC91-1099 72 hpi (m.i) | 3 | 6 | UK | -0.32552 | 0.001549 |
| IAC91-1099 72 hpi (m.i) | 3 | 7 | UK | -0.32698 | 0.000536 |
| IAC91-1099 72 hpi (m.i) | 3 | 8 | UK | -0.32816 | -0.0002 |
| IAC91-1099 72 hpi (m.i) | 3 | 9 | UK | -0.32604 | 0.00236 |
| IAC91-1099 72 hpi (m.i) | 3 | 10 | UK | -0.32728 | 0.001561 |
| IAC91-1099 72 hpi (m.i) | 3 | 11 | UK | -0.32633 | 0.002952 |
| IAC91-1099 72 hpi (m.i) | 3 | 12 | UK | -0.3324 | -0.00267 |
| IAC91-1099 72 hpi (m.i) | 3 | 13 | UK | -0.33065 | -0.00049 |
| IAC91-1099 72 hpi (m.i) | 3 | 14 | UK | -0.32872 | 0.001888 |
| IAC91-1099 72 hpi (m.i) | 3 | 15 | UK | -0.33497 | -0.00392 |
| IAC91-1099 72 hpi (m.i) | 3 | 16 | UK | -0.33185 | -0.00036 |
| IAC91-1099 72 hpi (m.i) | 3 | 17 | UK | -0.334 | -0.00206 |
| IAC91-1099 72 hpi (m.i) | 3 | 18 | UK | -0.33274 | -0.00036 |
| IAC91-1099 72 hpi (m.i) | 3 | 19 | UK | -0.3336 | -0.00078 |
| IAC91-1099 72 hpi (m.i) | 3 | 20 | UK | -0.33408 | -0.00082 |
| IAC91-1099 72 hpi (m.i) | 3 | 21 | UK | -0.33 | 0.003704 |
| IAC91-1099 72 hpi (m.i) | 3 | 22 | UK | -0.3208 | 0.013348 |
| IAC91-1099 72 hpi (m.i) | 3 | 23 | UK | -0.30706 | 0.027529 |
| IAC91-1099 72 hpi (m.i) | 3 | 24 | UK | -0.28136 | 0.053669 |
| IAC91-1099 72 hpi (m.i) | 3 | 25 | UK | -0.23257 | 0.102907 |
| IAC91-1099 72 hpi (m.i) | 3 | 26 | UK | -0.13731 | 0.198602 |
| IAC91-1099 72 hpi (m.i) | 3 | 27 | UK | 0.050579 | 0.386936 |
| IAC91-1099 72 hpi (m.i) | 3 | 28 | UK | 0.398591 | 0.73539 |
| IAC91-1099 72 hpi (m.i) | 3 | 29 | UK | 0.970115 | 1.307357 |
| IAC91-1099 72 hpi (m.i) | 3 | 30 | UK | 1.803465 | 2.14115 |
| IAC91-1099 72 hpi (m.i) | 3 | 31 | UK | 2.71527 | 3.053396 |
| IAC91-1099 72 hpi (m.i) | 3 | 32 | UK | 3.521585 | 3.860153 |
| IAC91-1099 72 hpi (m.i) | 3 | 33 | UK | 4.240312 | 4.579323 |
| IAC91-1099 72 hpi (m.i) | 3 | 34 | UK | 4.843343 | 5.182796 |
| IAC91-1099 72 hpi (m.i) | 3 | 35 | UK | 5.37102 | 5.710915 |
| IAC91-1099 72 hpi (m.i) | 3 | 36 | UK | 5.78714 | 6.127478 |
| IAC91-1099 72 hpi (m.i) | 3 | 37 | UK | 6.134905 | 6.475685 |
| IAC91-1099 72 hpi (m.i) | 3 | 38 | UK | 6.454195 | 6.795418 |
| IAC91-1099 72 hpi (m.i) | 3 | 39 | UK | 6.686058 | 7.027722 |
| IAC91-1099 72 hpi (m.i) | 3 | 40 | UK | 6.915888 | 7.257995 |
| IAC91-1099 72 hpi (s.i) | 1 | 1 | UK | -0.40069 | -0.0163 |
| IAC91-1099 72 hpi (s.i) | 1 | 2 | UK | -0.39371 | -0.00937 |
| IAC91-1099 72 hpi (s.i) | 1 | 3 | UK | -0.38777 | -0.00349 |
| IAC91-1099 72 hpi (s.i) | 1 | 4 | UK | -0.38561 | -0.00139 |
| IAC91-1099 72 hpi (s.i) | 1 | 5 | UK | -0.38541 | -0.00125 |
| IAC91-1099 72 hpi (s.i) | 1 | 6 | UK | -0.38467 | -0.00056 |
| IAC91-1099 72 hpi (s.i) | 1 | 7 | UK | -0.38566 | -0.00161 |
| IAC91-1099 72 hpi (s.i) | 1 | 8 | UK | -0.38057 | 0.003422 |
| IAC91-1099 72 hpi (s.i) | 1 | 9 | UK | -0.38133 | 0.002602 |
| IAC91-1099 72 hpi (s.i) | 1 | 10 | UK | -0.38411 | -0.00023 |
| IAC91-1099 72 hpi (s.i) | 1 | 11 | UK | -0.37999 | 0.003828 |
| IAC91-1099 72 hpi (s.i) | 1 | 12 | UK | -0.38156 | 0.002203 |
| IAC91-1099 72 hpi (s.i) | 1 | 13 | UK | -0.38159 | 0.002115 |
| IAC91-1099 72 hpi (s.i) | 1 | 14 | UK | -0.38261 | 0.001038 |
| IAC91-1099 72 hpi (s.i) | 1 | 15 | UK | -0.38341 | 0.000186 |
| IAC91-1099 72 hpi (s.i) | 1 | 16 | UK | -0.3844 | -0.00087 |
| IAC91-1099 72 hpi (s.i) | 1 | 17 | UK | -0.38539 | -0.00191 |
| IAC91-1099 72 hpi (s.i) | 1 | 18 | UK | -0.38373 | -0.0003 |
| IAC91-1099 72 hpi (s.i) | 1 | 19 | UK | -0.38468 | -0.00132 |
| IAC91-1099 72 hpi (s.i) | 1 | 20 | UK | -0.38548 | -0.00218 |
| IAC91-1099 72 hpi (s.i) | 1 | 21 | UK | -0.38506 | -0.00181 |
| IAC91-1099 72 hpi (s.i) | 1 | 22 | UK | -0.38166 | 0.001528 |
| IAC91-1099 72 hpi (s.i) | 1 | 23 | UK | -0.37653 | 0.006603 |
| IAC91-1099 72 hpi (s.i) | 1 | 24 | UK | -0.36548 | 0.017593 |
| IAC91-1099 72 hpi (s.i) | 1 | 25 | UK | -0.34536 | 0.037662 |
| IAC91-1099 72 hpi (s.i) | 1 | 26 | UK | -0.30684 | 0.076125 |
| IAC91-1099 72 hpi (s.i) | 1 | 27 | UK | -0.23118 | 0.151726 |
| IAC91-1099 72 hpi (s.i) | 1 | 28 | UK | -0.08274 | 0.30011 |
| IAC91-1099 72 hpi (s.i) | 1 | 29 | UK | 0.177768 | 0.560559 |
| IAC91-1099 72 hpi (s.i) | 1 | 30 | UK | 0.619942 | 1.002675 |
| IAC91-1099 72 hpi (s.i) | 1 | 31 | UK | 1.246134 | 1.62881 |
| IAC91-1099 72 hpi (s.i) | 1 | 32 | UK | 1.922698 | 2.305317 |
| IAC91-1099 72 hpi (s.i) | 1 | 33 | UK | 2.575795 | 2.958356 |
| IAC91-1099 72 hpi (s.i) | 1 | 34 | UK | 3.168438 | 3.550941 |
| IAC91-1099 72 hpi (s.i) | 1 | 35 | UK | 3.705415 | 4.087861 |
| IAC91-1099 72 hpi (s.i) | 1 | 36 | UK | 4.160873 | 4.543262 |
| IAC91-1099 72 hpi (s.i) | 1 | 37 | UK | 4.540117 | 4.922449 |
| IAC91-1099 72 hpi (s.i) | 1 | 38 | UK | 4.892169 | 5.274444 |
| IAC91-1099 72 hpi (s.i) | 1 | 39 | UK | 5.156295 | 5.538512 |
| IAC91-1099 72 hpi (s.i) | 1 | 40 | UK | 5.394657 | 5.776817 |
| IAC91-1099 72 hpi (s.i) | 2 | 1 | UK | -0.36494 | -0.01444 |
| IAC91-1099 72 hpi (s.i) | 2 | 2 | UK | -0.35865 | -0.00774 |
| IAC91-1099 72 hpi (s.i) | 2 | 3 | UK | -0.35527 | -0.00397 |
| IAC91-1099 72 hpi (s.i) | 2 | 4 | UK | -0.35211 | -0.00041 |
| IAC91-1099 72 hpi (s.i) | 2 | 5 | UK | -0.3514 | 0.000699 |
| IAC91-1099 72 hpi (s.i) | 2 | 6 | UK | -0.35026 | 0.002236 |
| IAC91-1099 72 hpi (s.i) | 2 | 7 | UK | -0.35169 | 0.001202 |
| IAC91-1099 72 hpi (s.i) | 2 | 8 | UK | -0.35135 | 0.001941 |
| IAC91-1099 72 hpi (s.i) | 2 | 9 | UK | -0.35116 | 0.002529 |
| IAC91-1099 72 hpi (s.i) | 2 | 10 | UK | -0.35179 | 0.002302 |
| IAC91-1099 72 hpi (s.i) | 2 | 11 | UK | -0.35246 | 0.002025 |
| IAC91-1099 72 hpi (s.i) | 2 | 12 | UK | -0.35629 | -0.0014 |
| IAC91-1099 72 hpi (s.i) | 2 | 13 | UK | -0.35552 | -0.00024 |
| IAC91-1099 72 hpi (s.i) | 2 | 14 | UK | -0.35631 | -0.00063 |
| IAC91-1099 72 hpi (s.i) | 2 | 15 | UK | -0.35943 | -0.00336 |
| IAC91-1099 72 hpi (s.i) | 2 | 16 | UK | -0.36026 | -0.00378 |
| IAC91-1099 72 hpi (s.i) | 2 | 17 | UK | -0.35948 | -0.0026 |
| IAC91-1099 72 hpi (s.i) | 2 | 18 | UK | -0.35922 | -0.00195 |
| IAC91-1099 72 hpi (s.i) | 2 | 19 | UK | -0.35892 | -0.00125 |
| IAC91-1099 72 hpi (s.i) | 2 | 20 | UK | -0.35938 | -0.00131 |
| IAC91-1099 72 hpi (s.i) | 2 | 21 | UK | -0.35678 | 0.001688 |
| IAC91-1099 72 hpi (s.i) | 2 | 22 | UK | -0.35259 | 0.006274 |
| IAC91-1099 72 hpi (s.i) | 2 | 23 | UK | -0.34281 | 0.016451 |
| IAC91-1099 72 hpi (s.i) | 2 | 24 | UK | -0.32315 | 0.036515 |
| IAC91-1099 72 hpi (s.i) | 2 | 25 | UK | -0.28385 | 0.076213 |
| IAC91-1099 72 hpi (s.i) | 2 | 26 | UK | -0.20575 | 0.154704 |
| IAC91-1099 72 hpi (s.i) | 2 | 27 | UK | -0.06666 | 0.294196 |
| IAC91-1099 72 hpi (s.i) | 2 | 28 | UK | 0.208402 | 0.569655 |
| IAC91-1099 72 hpi (s.i) | 2 | 29 | UK | 0.676774 | 1.038425 |
| IAC91-1099 72 hpi (s.i) | 2 | 30 | UK | 1.415041 | 1.77709 |
| IAC91-1099 72 hpi (s.i) | 2 | 31 | UK | 2.328012 | 2.690459 |
| IAC91-1099 72 hpi (s.i) | 2 | 32 | UK | 3.169327 | 3.532172 |
| IAC91-1099 72 hpi (s.i) | 2 | 33 | UK | 3.91041 | 4.273654 |
| IAC91-1099 72 hpi (s.i) | 2 | 34 | UK | 4.539423 | 4.903065 |
| IAC91-1099 72 hpi (s.i) | 2 | 35 | UK | 5.081163 | 5.445202 |
| IAC91-1099 72 hpi (s.i) | 2 | 36 | UK | 5.51098 | 5.875417 |
| IAC91-1099 72 hpi (s.i) | 2 | 37 | UK | 5.868557 | 6.233393 |
| IAC91-1099 72 hpi (s.i) | 2 | 38 | UK | 6.192085 | 6.557319 |
| IAC91-1099 72 hpi (s.i) | 2 | 39 | UK | 6.432266 | 6.797898 |
| IAC91-1099 72 hpi (s.i) | 2 | 40 | UK | 6.635678 | 7.001708 |
| IAC91-1099 72 hpi (s.i) | 3 | 1 | UK | -0.29663 | -0.00961 |
| IAC91-1099 72 hpi (s.i) | 3 | 2 | UK | -0.29291 | -0.00591 |
| IAC91-1099 72 hpi (s.i) | 3 | 3 | UK | -0.28909 | -0.00211 |
| IAC91-1099 72 hpi (s.i) | 3 | 4 | UK | -0.28667 | 0.000286 |
| IAC91-1099 72 hpi (s.i) | 3 | 5 | UK | -0.28642 | 0.000517 |
| IAC91-1099 72 hpi (s.i) | 3 | 6 | UK | -0.28456 | 0.002355 |
| IAC91-1099 72 hpi (s.i) | 3 | 7 | UK | -0.28532 | 0.001573 |
| IAC91-1099 72 hpi (s.i) | 3 | 8 | UK | -0.28474 | 0.00213 |
| IAC91-1099 72 hpi (s.i) | 3 | 9 | UK | -0.28532 | 0.001525 |
| IAC91-1099 72 hpi (s.i) | 3 | 10 | UK | -0.28664 | 0.000185 |
| IAC91-1099 72 hpi (s.i) | 3 | 11 | UK | -0.28716 | -0.00036 |
| IAC91-1099 72 hpi (s.i) | 3 | 12 | UK | -0.28708 | -0.0003 |
| IAC91-1099 72 hpi (s.i) | 3 | 13 | UK | -0.28724 | -0.00048 |
| IAC91-1099 72 hpi (s.i) | 3 | 14 | UK | -0.28775 | -0.00102 |
| IAC91-1099 72 hpi (s.i) | 3 | 15 | UK | -0.29018 | -0.00346 |
| IAC91-1099 72 hpi (s.i) | 3 | 16 | UK | -0.28994 | -0.00325 |
| IAC91-1099 72 hpi (s.i) | 3 | 17 | UK | -0.29021 | -0.00353 |
| IAC91-1099 72 hpi (s.i) | 3 | 18 | UK | -0.28786 | -0.00121 |
| IAC91-1099 72 hpi (s.i) | 3 | 19 | UK | -0.28858 | -0.00195 |
| IAC91-1099 72 hpi (s.i) | 3 | 20 | UK | -0.28584 | 0.000762 |
| IAC91-1099 72 hpi (s.i) | 3 | 21 | UK | -0.28363 | 0.002959 |
| IAC91-1099 72 hpi (s.i) | 3 | 22 | UK | -0.28119 | 0.005378 |
| IAC91-1099 72 hpi (s.i) | 3 | 23 | UK | -0.27141 | 0.015128 |
| IAC91-1099 72 hpi (s.i) | 3 | 24 | UK | -0.25257 | 0.033948 |
| IAC91-1099 72 hpi (s.i) | 3 | 25 | UK | -0.21251 | 0.073991 |
| IAC91-1099 72 hpi (s.i) | 3 | 26 | UK | -0.13705 | 0.149421 |
| IAC91-1099 72 hpi (s.i) | 3 | 27 | UK | 0.002408 | 0.288862 |
| IAC91-1099 72 hpi (s.i) | 3 | 28 | UK | 0.269616 | 0.556048 |
| IAC91-1099 72 hpi (s.i) | 3 | 29 | UK | 0.732115 | 1.018525 |
| IAC91-1099 72 hpi (s.i) | 3 | 30 | UK | 1.450121 | 1.736509 |
| IAC91-1099 72 hpi (s.i) | 3 | 31 | UK | 2.324328 | 2.610694 |
| IAC91-1099 72 hpi (s.i) | 3 | 32 | UK | 3.135878 | 3.422222 |
| IAC91-1099 72 hpi (s.i) | 3 | 33 | UK | 3.851999 | 4.138322 |
| IAC91-1099 72 hpi (s.i) | 3 | 34 | UK | 4.46087 | 4.747171 |
| IAC91-1099 72 hpi (s.i) | 3 | 35 | UK | 4.985877 | 5.272156 |
| IAC91-1099 72 hpi (s.i) | 3 | 36 | UK | 5.413829 | 5.700086 |
| IAC91-1099 72 hpi (s.i) | 3 | 37 | UK | 5.753787 | 6.040022 |
| IAC91-1099 72 hpi (s.i) | 3 | 38 | UK | 6.074918 | 6.361131 |
| IAC91-1099 72 hpi (s.i) | 3 | 39 | UK | 6.292913 | 6.579104 |
| IAC91-1099 72 hpi (s.i) | 3 | 40 | UK | 6.507141 | 6.793311 |
| IACSP95-5000 24 hpi (m.i) | 1 | 1 | UK | -0.28538 | -0.00304 |
| IACSP95-5000 24 hpi (m.i) | 1 | 2 | UK | -0.28505 | -0.00284 |
| IACSP95-5000 24 hpi (m.i) | 1 | 3 | UK | -0.28071 | 0.00136 |
| IACSP95-5000 24 hpi (m.i) | 1 | 4 | UK | -0.27921 | 0.002727 |
| IACSP95-5000 24 hpi (m.i) | 1 | 5 | UK | -0.27789 | 0.003904 |
| IACSP95-5000 24 hpi (m.i) | 1 | 6 | UK | -0.2816 | 6.05E-05 |
| IACSP95-5000 24 hpi (m.i) | 1 | 7 | UK | -0.28266 | -0.00113 |
| IACSP95-5000 24 hpi (m.i) | 1 | 8 | UK | -0.28062 | 0.000771 |
| IACSP95-5000 24 hpi (m.i) | 1 | 9 | UK | -0.28207 | -0.00082 |
| IACSP95-5000 24 hpi (m.i) | 1 | 10 | UK | -0.28353 | -0.00242 |
| IACSP95-5000 24 hpi (m.i) | 1 | 11 | UK | -0.28126 | -0.00028 |
| IACSP95-5000 24 hpi (m.i) | 1 | 12 | UK | -0.28473 | -0.00389 |
| IACSP95-5000 24 hpi (m.i) | 1 | 13 | UK | -0.28427 | -0.00356 |
| IACSP95-5000 24 hpi (m.i) | 1 | 14 | UK | -0.28076 | -0.00019 |
| IACSP95-5000 24 hpi (m.i) | 1 | 15 | UK | -0.28201 | -0.00157 |
| IACSP95-5000 24 hpi (m.i) | 1 | 16 | UK | -0.28458 | -0.00428 |
| IACSP95-5000 24 hpi (m.i) | 1 | 17 | UK | -0.27985 | 0.000312 |
| IACSP95-5000 24 hpi (m.i) | 1 | 18 | UK | -0.27889 | 0.001134 |
| IACSP95-5000 24 hpi (m.i) | 1 | 19 | UK | -0.27957 | 0.000324 |
| IACSP95-5000 24 hpi (m.i) | 1 | 20 | UK | -0.27849 | 0.001265 |
| IACSP95-5000 24 hpi (m.i) | 1 | 21 | UK | -0.27333 | 0.006289 |
| IACSP95-5000 24 hpi (m.i) | 1 | 22 | UK | -0.26639 | 0.01309 |
| IACSP95-5000 24 hpi (m.i) | 1 | 23 | UK | -0.24903 | 0.030312 |
| IACSP95-5000 24 hpi (m.i) | 1 | 24 | UK | -0.22499 | 0.05422 |
| IACSP95-5000 24 hpi (m.i) | 1 | 25 | UK | -0.1717 | 0.107369 |
| IACSP95-5000 24 hpi (m.i) | 1 | 26 | UK | -0.07269 | 0.206249 |
| IACSP95-5000 24 hpi (m.i) | 1 | 27 | UK | 0.111781 | 0.390582 |
| IACSP95-5000 24 hpi (m.i) | 1 | 28 | UK | 0.450573 | 0.729237 |
| IACSP95-5000 24 hpi (m.i) | 1 | 29 | UK | 1.013908 | 1.292436 |
| IACSP95-5000 24 hpi (m.i) | 1 | 30 | UK | 1.814858 | 2.093249 |
| IACSP95-5000 24 hpi (m.i) | 1 | 31 | UK | 2.691246 | 2.969501 |
| IACSP95-5000 24 hpi (m.i) | 1 | 32 | UK | 3.484815 | 3.762935 |
| IACSP95-5000 24 hpi (m.i) | 1 | 33 | UK | 4.201261 | 4.479244 |
| IACSP95-5000 24 hpi (m.i) | 1 | 34 | UK | 4.832636 | 5.110483 |
| IACSP95-5000 24 hpi (m.i) | 1 | 35 | UK | 5.37677 | 5.654481 |
| IACSP95-5000 24 hpi (m.i) | 1 | 36 | UK | 5.810699 | 6.088273 |
| IACSP95-5000 24 hpi (m.i) | 1 | 37 | UK | 6.158525 | 6.435964 |
| IACSP95-5000 24 hpi (m.i) | 1 | 38 | UK | 6.48957 | 6.766872 |
| IACSP95-5000 24 hpi (m.i) | 1 | 39 | UK | 6.729269 | 7.006435 |
| IACSP95-5000 24 hpi (m.i) | 1 | 40 | UK | 6.951072 | 7.228102 |
| IACSP95-5000 24 hpi (m.i) | 2 | 1 | UK | -0.28556 | -0.00453 |
| IACSP95-5000 24 hpi (m.i) | 2 | 2 | UK | -0.28393 | -0.00266 |
| IACSP95-5000 24 hpi (m.i) | 2 | 3 | UK | -0.28112 | 0.000381 |
| IACSP95-5000 24 hpi (m.i) | 2 | 4 | UK | -0.27933 | 0.002409 |
| IACSP95-5000 24 hpi (m.i) | 2 | 5 | UK | -0.28041 | 0.001577 |
| IACSP95-5000 24 hpi (m.i) | 2 | 6 | UK | -0.28169 | 0.000532 |
| IACSP95-5000 24 hpi (m.i) | 2 | 7 | UK | -0.2824 | 6.14E-05 |
| IACSP95-5000 24 hpi (m.i) | 2 | 8 | UK | -0.28311 | -0.00041 |
| IACSP95-5000 24 hpi (m.i) | 2 | 9 | UK | -0.28292 | 1.75E-05 |
| IACSP95-5000 24 hpi (m.i) | 2 | 10 | UK | -0.28388 | -0.0007 |
| IACSP95-5000 24 hpi (m.i) | 2 | 11 | UK | -0.28367 | -0.00025 |
| IACSP95-5000 24 hpi (m.i) | 2 | 12 | UK | -0.28467 | -0.00102 |
| IACSP95-5000 24 hpi (m.i) | 2 | 13 | UK | -0.28601 | -0.00212 |
| IACSP95-5000 24 hpi (m.i) | 2 | 14 | UK | -0.28368 | 0.000458 |
| IACSP95-5000 24 hpi (m.i) | 2 | 15 | UK | -0.28739 | -0.00301 |
| IACSP95-5000 24 hpi (m.i) | 2 | 16 | UK | -0.28814 | -0.00352 |
| IACSP95-5000 24 hpi (m.i) | 2 | 17 | UK | -0.28644 | -0.00158 |
| IACSP95-5000 24 hpi (m.i) | 2 | 18 | UK | -0.28598 | -0.00088 |
| IACSP95-5000 24 hpi (m.i) | 2 | 19 | UK | -0.28571 | -0.00038 |
| IACSP95-5000 24 hpi (m.i) | 2 | 20 | UK | -0.28388 | 0.001689 |
| IACSP95-5000 24 hpi (m.i) | 2 | 21 | UK | -0.27907 | 0.006738 |
| IACSP95-5000 24 hpi (m.i) | 2 | 22 | UK | -0.27256 | 0.013488 |
| IACSP95-5000 24 hpi (m.i) | 2 | 23 | UK | -0.25838 | 0.027911 |
| IACSP95-5000 24 hpi (m.i) | 2 | 24 | UK | -0.23078 | 0.055749 |
| IACSP95-5000 24 hpi (m.i) | 2 | 25 | UK | -0.17905 | 0.107717 |
| IACSP95-5000 24 hpi (m.i) | 2 | 26 | UK | -0.07941 | 0.207598 |
| IACSP95-5000 24 hpi (m.i) | 2 | 27 | UK | 0.103788 | 0.391037 |
| IACSP95-5000 24 hpi (m.i) | 2 | 28 | UK | 0.447377 | 0.734865 |
| IACSP95-5000 24 hpi (m.i) | 2 | 29 | UK | 1.012007 | 1.299735 |
| IACSP95-5000 24 hpi (m.i) | 2 | 30 | UK | 1.823465 | 2.111432 |
| IACSP95-5000 24 hpi (m.i) | 2 | 31 | UK | 2.717728 | 3.005935 |
| IACSP95-5000 24 hpi (m.i) | 2 | 32 | UK | 3.517122 | 3.805568 |
| IACSP95-5000 24 hpi (m.i) | 2 | 33 | UK | 4.244397 | 4.533082 |
| IACSP95-5000 24 hpi (m.i) | 2 | 34 | UK | 4.867321 | 5.156246 |
| IACSP95-5000 24 hpi (m.i) | 2 | 35 | UK | 5.399664 | 5.688828 |
| IACSP95-5000 24 hpi (m.i) | 2 | 36 | UK | 5.834078 | 6.123482 |
| IACSP95-5000 24 hpi (m.i) | 2 | 37 | UK | 6.181378 | 6.471022 |
| IACSP95-5000 24 hpi (m.i) | 2 | 38 | UK | 6.504403 | 6.794285 |
| IACSP95-5000 24 hpi (m.i) | 2 | 39 | UK | 6.73694 | 7.027062 |
| IACSP95-5000 24 hpi (m.i) | 2 | 40 | UK | 6.955319 | 7.245681 |
| IACSP95-5000 24 hpi (m.i) | 3 | 1 | UK | -0.18169 | -0.01404 |
| IACSP95-5000 24 hpi (m.i) | 3 | 2 | UK | -0.17276 | -0.0051 |
| IACSP95-5000 24 hpi (m.i) | 3 | 3 | UK | -0.16994 | -0.00227 |
| IACSP95-5000 24 hpi (m.i) | 3 | 4 | UK | -0.16704 | 0.000643 |
| IACSP95-5000 24 hpi (m.i) | 3 | 5 | UK | -0.16844 | -0.00075 |
| IACSP95-5000 24 hpi (m.i) | 3 | 6 | UK | -0.16603 | 0.001676 |
| IACSP95-5000 24 hpi (m.i) | 3 | 7 | UK | -0.16776 | -4.3E-05 |
| IACSP95-5000 24 hpi (m.i) | 3 | 8 | UK | -0.16715 | 0.000568 |
| IACSP95-5000 24 hpi (m.i) | 3 | 9 | UK | -0.16613 | 0.001603 |
| IACSP95-5000 24 hpi (m.i) | 3 | 10 | UK | -0.16578 | 0.001965 |
| IACSP95-5000 24 hpi (m.i) | 3 | 11 | UK | -0.16752 | 0.000236 |
| IACSP95-5000 24 hpi (m.i) | 3 | 12 | UK | -0.168 | -0.00024 |
| IACSP95-5000 24 hpi (m.i) | 3 | 13 | UK | -0.16758 | 0.000188 |
| IACSP95-5000 24 hpi (m.i) | 3 | 14 | UK | -0.16648 | 0.0013 |
| IACSP95-5000 24 hpi (m.i) | 3 | 15 | UK | -0.16948 | -0.00169 |
| IACSP95-5000 24 hpi (m.i) | 3 | 16 | UK | -0.17057 | -0.00277 |
| IACSP95-5000 24 hpi (m.i) | 3 | 17 | UK | -0.17098 | -0.00316 |
| IACSP95-5000 24 hpi (m.i) | 3 | 18 | UK | -0.16854 | -0.00072 |
| IACSP95-5000 24 hpi (m.i) | 3 | 19 | UK | -0.1698 | -0.00196 |
| IACSP95-5000 24 hpi (m.i) | 3 | 20 | UK | -0.16686 | 0.000982 |
| IACSP95-5000 24 hpi (m.i) | 3 | 21 | UK | -0.16342 | 0.004435 |
| IACSP95-5000 24 hpi (m.i) | 3 | 22 | UK | -0.15622 | 0.011646 |
| IACSP95-5000 24 hpi (m.i) | 3 | 23 | UK | -0.14359 | 0.024279 |
| IACSP95-5000 24 hpi (m.i) | 3 | 24 | UK | -0.12007 | 0.04781 |
| IACSP95-5000 24 hpi (m.i) | 3 | 25 | UK | -0.07259 | 0.095299 |
| IACSP95-5000 24 hpi (m.i) | 3 | 26 | UK | 0.01867 | 0.186573 |
| IACSP95-5000 24 hpi (m.i) | 3 | 27 | UK | 0.188138 | 0.356052 |
| IACSP95-5000 24 hpi (m.i) | 3 | 28 | UK | 0.50626 | 0.674184 |
| IACSP95-5000 24 hpi (m.i) | 3 | 29 | UK | 1.031816 | 1.199749 |
| IACSP95-5000 24 hpi (m.i) | 3 | 30 | UK | 1.794848 | 1.962791 |
| IACSP95-5000 24 hpi (m.i) | 3 | 31 | UK | 2.646477 | 2.814431 |
| IACSP95-5000 24 hpi (m.i) | 3 | 32 | UK | 3.418901 | 3.586865 |
| IACSP95-5000 24 hpi (m.i) | 3 | 33 | UK | 4.128136 | 4.296109 |
| IACSP95-5000 24 hpi (m.i) | 3 | 34 | UK | 4.736509 | 4.904493 |
| IACSP95-5000 24 hpi (m.i) | 3 | 35 | UK | 5.263842 | 5.431836 |
| IACSP95-5000 24 hpi (m.i) | 3 | 36 | UK | 5.685982 | 5.853986 |
| IACSP95-5000 24 hpi (m.i) | 3 | 37 | UK | 6.027956 | 6.19597 |
| IACSP95-5000 24 hpi (m.i) | 3 | 38 | UK | 6.350281 | 6.518305 |
| IACSP95-5000 24 hpi (m.i) | 3 | 39 | UK | 6.577581 | 6.745615 |
| IACSP95-5000 24 hpi (m.i) | 3 | 40 | UK | 6.792778 | 6.960822 |
| IACSP95-5000 24 hpi (s.i) | 1 | 1 | UK | -0.28678 | -0.01104 |
| IACSP95-5000 24 hpi (s.i) | 1 | 2 | UK | -0.28329 | -0.00761 |
| IACSP95-5000 24 hpi (s.i) | 1 | 3 | UK | -0.27697 | -0.00136 |
| IACSP95-5000 24 hpi (s.i) | 1 | 4 | UK | -0.27416 | 0.001391 |
| IACSP95-5000 24 hpi (s.i) | 1 | 5 | UK | -0.27463 | 0.000854 |
| IACSP95-5000 24 hpi (s.i) | 1 | 6 | UK | -0.2764 | -0.00099 |
| IACSP95-5000 24 hpi (s.i) | 1 | 7 | UK | -0.27405 | 0.001297 |
| IACSP95-5000 24 hpi (s.i) | 1 | 8 | UK | -0.27637 | -0.00109 |
| IACSP95-5000 24 hpi (s.i) | 1 | 9 | UK | -0.27257 | 0.002648 |
| IACSP95-5000 24 hpi (s.i) | 1 | 10 | UK | -0.27443 | 0.000721 |
| IACSP95-5000 24 hpi (s.i) | 1 | 11 | UK | -0.2755 | -0.00041 |
| IACSP95-5000 24 hpi (s.i) | 1 | 12 | UK | -0.27664 | -0.00162 |
| IACSP95-5000 24 hpi (s.i) | 1 | 13 | UK | -0.27408 | 0.000878 |
| IACSP95-5000 24 hpi (s.i) | 1 | 14 | UK | -0.27578 | -0.00088 |
| IACSP95-5000 24 hpi (s.i) | 1 | 15 | UK | -0.27549 | -0.00066 |
| IACSP95-5000 24 hpi (s.i) | 1 | 16 | UK | -0.27607 | -0.00131 |
| IACSP95-5000 24 hpi (s.i) | 1 | 17 | UK | -0.27714 | -0.00244 |
| IACSP95-5000 24 hpi (s.i) | 1 | 18 | UK | -0.27536 | -0.00073 |
| IACSP95-5000 24 hpi (s.i) | 1 | 19 | UK | -0.27497 | -0.0004 |
| IACSP95-5000 24 hpi (s.i) | 1 | 20 | UK | -0.2752 | -0.0007 |
| IACSP95-5000 24 hpi (s.i) | 1 | 21 | UK | -0.26965 | 0.004792 |
| IACSP95-5000 24 hpi (s.i) | 1 | 22 | UK | -0.26112 | 0.013256 |
| IACSP95-5000 24 hpi (s.i) | 1 | 23 | UK | -0.24783 | 0.026472 |
| IACSP95-5000 24 hpi (s.i) | 1 | 24 | UK | -0.21613 | 0.058113 |
| IACSP95-5000 24 hpi (s.i) | 1 | 25 | UK | -0.15957 | 0.11461 |
| IACSP95-5000 24 hpi (s.i) | 1 | 26 | UK | -0.05016 | 0.223952 |
| IACSP95-5000 24 hpi (s.i) | 1 | 27 | UK | 0.155727 | 0.429773 |
| IACSP95-5000 24 hpi (s.i) | 1 | 28 | UK | 0.537115 | 0.811096 |
| IACSP95-5000 24 hpi (s.i) | 1 | 29 | UK | 1.153955 | 1.427871 |
| IACSP95-5000 24 hpi (s.i) | 1 | 30 | UK | 2.024658 | 2.298509 |
| IACSP95-5000 24 hpi (s.i) | 1 | 31 | UK | 2.951761 | 3.225546 |
| IACSP95-5000 24 hpi (s.i) | 1 | 32 | UK | 3.786121 | 4.059841 |
| IACSP95-5000 24 hpi (s.i) | 1 | 33 | UK | 4.532517 | 4.806171 |
| IACSP95-5000 24 hpi (s.i) | 1 | 34 | UK | 5.169887 | 5.443476 |
| IACSP95-5000 24 hpi (s.i) | 1 | 35 | UK | 5.728702 | 6.002225 |
| IACSP95-5000 24 hpi (s.i) | 1 | 36 | UK | 6.163954 | 6.437413 |
| IACSP95-5000 24 hpi (s.i) | 1 | 37 | UK | 6.533262 | 6.806655 |
| IACSP95-5000 24 hpi (s.i) | 1 | 38 | UK | 6.866175 | 7.139503 |
| IACSP95-5000 24 hpi (s.i) | 1 | 39 | UK | 7.1051 | 7.378363 |
| IACSP95-5000 24 hpi (s.i) | 1 | 40 | UK | 7.343058 | 7.616256 |
| IACSP95-5000 24 hpi (s.i) | 2 | 1 | UK | -0.24048 | -0.00768 |
| IACSP95-5000 24 hpi (s.i) | 2 | 2 | UK | -0.2378 | -0.00533 |
| IACSP95-5000 24 hpi (s.i) | 2 | 3 | UK | -0.23302 | -0.00089 |
| IACSP95-5000 24 hpi (s.i) | 2 | 4 | UK | -0.23061 | 0.001176 |
| IACSP95-5000 24 hpi (s.i) | 2 | 5 | UK | -0.22972 | 0.001733 |
| IACSP95-5000 24 hpi (s.i) | 2 | 6 | UK | -0.22986 | 0.001256 |
| IACSP95-5000 24 hpi (s.i) | 2 | 7 | UK | -0.23025 | 0.000519 |
| IACSP95-5000 24 hpi (s.i) | 2 | 8 | UK | -0.23011 | 0.000319 |
| IACSP95-5000 24 hpi (s.i) | 2 | 9 | UK | -0.2294 | 0.000694 |
| IACSP95-5000 24 hpi (s.i) | 2 | 10 | UK | -0.22799 | 0.001762 |
| IACSP95-5000 24 hpi (s.i) | 2 | 11 | UK | -0.23004 | -0.00063 |
| IACSP95-5000 24 hpi (s.i) | 2 | 12 | UK | -0.22974 | -0.00067 |
| IACSP95-5000 24 hpi (s.i) | 2 | 13 | UK | -0.22936 | -0.00063 |
| IACSP95-5000 24 hpi (s.i) | 2 | 14 | UK | -0.23124 | -0.00284 |
| IACSP95-5000 24 hpi (s.i) | 2 | 15 | UK | -0.23177 | -0.00372 |
| IACSP95-5000 24 hpi (s.i) | 2 | 16 | UK | -0.23183 | -0.00411 |
| IACSP95-5000 24 hpi (s.i) | 2 | 17 | UK | -0.23017 | -0.00279 |
| IACSP95-5000 24 hpi (s.i) | 2 | 18 | UK | -0.22833 | -0.00129 |
| IACSP95-5000 24 hpi (s.i) | 2 | 19 | UK | -0.2257 | 0.000997 |
| IACSP95-5000 24 hpi (s.i) | 2 | 20 | UK | -0.22303 | 0.003325 |
| IACSP95-5000 24 hpi (s.i) | 2 | 21 | UK | -0.22024 | 0.00578 |
| IACSP95-5000 24 hpi (s.i) | 2 | 22 | UK | -0.21216 | 0.013516 |
| IACSP95-5000 24 hpi (s.i) | 2 | 23 | UK | -0.19502 | 0.030322 |
| IACSP95-5000 24 hpi (s.i) | 2 | 24 | UK | -0.16279 | 0.062207 |
| IACSP95-5000 24 hpi (s.i) | 2 | 25 | UK | -0.10333 | 0.121332 |
| IACSP95-5000 24 hpi (s.i) | 2 | 26 | UK | 0.015401 | 0.239723 |
| IACSP95-5000 24 hpi (s.i) | 2 | 27 | UK | 0.233488 | 0.45747 |
| IACSP95-5000 24 hpi (s.i) | 2 | 28 | UK | 0.63002 | 0.853663 |
| IACSP95-5000 24 hpi (s.i) | 2 | 29 | UK | 1.281775 | 1.505079 |
| IACSP95-5000 24 hpi (s.i) | 2 | 30 | UK | 2.202822 | 2.425786 |
| IACSP95-5000 24 hpi (s.i) | 2 | 31 | UK | 3.183708 | 3.406332 |
| IACSP95-5000 24 hpi (s.i) | 2 | 32 | UK | 4.035181 | 4.257466 |
| IACSP95-5000 24 hpi (s.i) | 2 | 33 | UK | 4.796032 | 5.017978 |
| IACSP95-5000 24 hpi (s.i) | 2 | 34 | UK | 5.434058 | 5.655664 |
| IACSP95-5000 24 hpi (s.i) | 2 | 35 | UK | 5.974152 | 6.195418 |
| IACSP95-5000 24 hpi (s.i) | 2 | 36 | UK | 6.413889 | 6.634816 |
| IACSP95-5000 24 hpi (s.i) | 2 | 37 | UK | 6.775589 | 6.996177 |
| IACSP95-5000 24 hpi (s.i) | 2 | 38 | UK | 7.099792 | 7.32004 |
| IACSP95-5000 24 hpi (s.i) | 2 | 39 | UK | 7.328183 | 7.548092 |
| IACSP95-5000 24 hpi (s.i) | 2 | 40 | UK | 7.54104 | 7.76061 |
| IACSP95-5000 24 hpi (s.i) | 3 | 1 | UK | -0.26827 | 0.000292 |
| IACSP95-5000 24 hpi (s.i) | 3 | 2 | UK | -0.2708 | -0.00254 |
| IACSP95-5000 24 hpi (s.i) | 3 | 3 | UK | -0.26748 | 0.000481 |
| IACSP95-5000 24 hpi (s.i) | 3 | 4 | UK | -0.26769 | -2.7E-05 |
| IACSP95-5000 24 hpi (s.i) | 3 | 5 | UK | -0.26521 | 0.002146 |
| IACSP95-5000 24 hpi (s.i) | 3 | 6 | UK | -0.26621 | 0.000844 |
| IACSP95-5000 24 hpi (s.i) | 3 | 7 | UK | -0.26664 | 0.000114 |
| IACSP95-5000 24 hpi (s.i) | 3 | 8 | UK | -0.26659 | -0.00013 |
| IACSP95-5000 24 hpi (s.i) | 3 | 9 | UK | -0.26398 | 0.002182 |
| IACSP95-5000 24 hpi (s.i) | 3 | 10 | UK | -0.26292 | 0.002942 |
| IACSP95-5000 24 hpi (s.i) | 3 | 11 | UK | -0.26577 | -0.00022 |
| IACSP95-5000 24 hpi (s.i) | 3 | 12 | UK | -0.26806 | -0.0028 |
| IACSP95-5000 24 hpi (s.i) | 3 | 13 | UK | -0.26743 | -0.00247 |
| IACSP95-5000 24 hpi (s.i) | 3 | 14 | UK | -0.26668 | -0.00202 |
| IACSP95-5000 24 hpi (s.i) | 3 | 15 | UK | -0.26886 | -0.0045 |
| IACSP95-5000 24 hpi (s.i) | 3 | 16 | UK | -0.26669 | -0.00264 |
| IACSP95-5000 24 hpi (s.i) | 3 | 17 | UK | -0.26638 | -0.00262 |
| IACSP95-5000 24 hpi (s.i) | 3 | 18 | UK | -0.26445 | -0.00099 |
| IACSP95-5000 24 hpi (s.i) | 3 | 19 | UK | -0.26401 | -0.00085 |
| IACSP95-5000 24 hpi (s.i) | 3 | 20 | UK | -0.25984 | 0.003016 |
| IACSP95-5000 24 hpi (s.i) | 3 | 21 | UK | -0.25502 | 0.007541 |
| IACSP95-5000 24 hpi (s.i) | 3 | 22 | UK | -0.24815 | 0.014106 |
| IACSP95-5000 24 hpi (s.i) | 3 | 23 | UK | -0.22776 | 0.034197 |
| IACSP95-5000 24 hpi (s.i) | 3 | 24 | UK | -0.1908 | 0.070856 |
| IACSP95-5000 24 hpi (s.i) | 3 | 25 | UK | -0.12263 | 0.138732 |
| IACSP95-5000 24 hpi (s.i) | 3 | 26 | UK | 0.008115 | 0.269175 |
| IACSP95-5000 24 hpi (s.i) | 3 | 27 | UK | 0.248966 | 0.509725 |
| IACSP95-5000 24 hpi (s.i) | 3 | 28 | UK | 0.689543 | 0.950003 |
| IACSP95-5000 24 hpi (s.i) | 3 | 29 | UK | 1.403571 | 1.663731 |
| IACSP95-5000 24 hpi (s.i) | 3 | 30 | UK | 2.385299 | 2.645159 |
| IACSP95-5000 24 hpi (s.i) | 3 | 31 | UK | 3.413836 | 3.673395 |
| IACSP95-5000 24 hpi (s.i) | 3 | 32 | UK | 4.294329 | 4.553589 |
| IACSP95-5000 24 hpi (s.i) | 3 | 33 | UK | 5.076011 | 5.334971 |
| IACSP95-5000 24 hpi (s.i) | 3 | 34 | UK | 5.729823 | 5.988483 |
| IACSP95-5000 24 hpi (s.i) | 3 | 35 | UK | 6.284463 | 6.542823 |
| IACSP95-5000 24 hpi (s.i) | 3 | 36 | UK | 6.729412 | 6.987472 |
| IACSP95-5000 24 hpi (s.i) | 3 | 37 | UK | 7.09551 | 7.35327 |
| IACSP95-5000 24 hpi (s.i) | 3 | 38 | UK | 7.424776 | 7.682236 |
| IACSP95-5000 24 hpi (s.i) | 3 | 39 | UK | 7.659338 | 7.916498 |
| IACSP95-5000 24 hpi (s.i) | 3 | 40 | UK | 7.880971 | 8.137832 |
| IACSP95-5000 72 hpi (m.i) | 1 | 1 | UK | -0.22943 | -0.00957 |
| IACSP95-5000 72 hpi (m.i) | 1 | 2 | UK | -0.22295 | -0.00279 |
| IACSP95-5000 72 hpi (m.i) | 1 | 3 | UK | -0.22049 | -4.1E-05 |
| IACSP95-5000 72 hpi (m.i) | 1 | 4 | UK | -0.22121 | -0.00047 |
| IACSP95-5000 72 hpi (m.i) | 1 | 5 | UK | -0.21903 | 0.002006 |
| IACSP95-5000 72 hpi (m.i) | 1 | 6 | UK | -0.21967 | 0.001659 |
| IACSP95-5000 72 hpi (m.i) | 1 | 7 | UK | -0.22113 | 0.000487 |
| IACSP95-5000 72 hpi (m.i) | 1 | 8 | UK | -0.22099 | 0.000922 |
| IACSP95-5000 72 hpi (m.i) | 1 | 9 | UK | -0.21942 | 0.002784 |
| IACSP95-5000 72 hpi (m.i) | 1 | 10 | UK | -0.22329 | -0.0008 |
| IACSP95-5000 72 hpi (m.i) | 1 | 11 | UK | -0.22642 | -0.00364 |
| IACSP95-5000 72 hpi (m.i) | 1 | 12 | UK | -0.2271 | -0.00402 |
| IACSP95-5000 72 hpi (m.i) | 1 | 13 | UK | -0.22349 | -0.00012 |
| IACSP95-5000 72 hpi (m.i) | 1 | 14 | UK | -0.22543 | -0.00177 |
| IACSP95-5000 72 hpi (m.i) | 1 | 15 | UK | -0.22453 | -0.00057 |
| IACSP95-5000 72 hpi (m.i) | 1 | 16 | UK | -0.22495 | -0.0007 |
| IACSP95-5000 72 hpi (m.i) | 1 | 17 | UK | -0.22684 | -0.0023 |
| IACSP95-5000 72 hpi (m.i) | 1 | 18 | UK | -0.22394 | 0.000894 |
| IACSP95-5000 72 hpi (m.i) | 1 | 19 | UK | -0.2231 | 0.002024 |
| IACSP95-5000 72 hpi (m.i) | 1 | 20 | UK | -0.22176 | 0.003657 |
| IACSP95-5000 72 hpi (m.i) | 1 | 21 | UK | -0.21582 | 0.009885 |
| IACSP95-5000 72 hpi (m.i) | 1 | 22 | UK | -0.20499 | 0.021013 |
| IACSP95-5000 72 hpi (m.i) | 1 | 23 | UK | -0.19048 | 0.035813 |
| IACSP95-5000 72 hpi (m.i) | 1 | 24 | UK | -0.15807 | 0.068511 |
| IACSP95-5000 72 hpi (m.i) | 1 | 25 | UK | -0.09396 | 0.132916 |
| IACSP95-5000 72 hpi (m.i) | 1 | 26 | UK | 0.027621 | 0.254788 |
| IACSP95-5000 72 hpi (m.i) | 1 | 27 | UK | 0.252107 | 0.479566 |
| IACSP95-5000 72 hpi (m.i) | 1 | 28 | UK | 0.668965 | 0.896716 |
| IACSP95-5000 72 hpi (m.i) | 1 | 29 | UK | 1.334457 | 1.5625 |
| IACSP95-5000 72 hpi (m.i) | 1 | 30 | UK | 2.269048 | 2.497383 |
| IACSP95-5000 72 hpi (m.i) | 1 | 31 | UK | 3.234966 | 3.463593 |
| IACSP95-5000 72 hpi (m.i) | 1 | 32 | UK | 4.089031 | 4.31795 |
| IACSP95-5000 72 hpi (m.i) | 1 | 33 | UK | 4.834036 | 5.063248 |
| IACSP95-5000 72 hpi (m.i) | 1 | 34 | UK | 5.463578 | 5.693081 |
| IACSP95-5000 72 hpi (m.i) | 1 | 35 | UK | 6.020291 | 6.250086 |
| IACSP95-5000 72 hpi (m.i) | 1 | 36 | UK | 6.440176 | 6.670263 |
| IACSP95-5000 72 hpi (m.i) | 1 | 37 | UK | 6.819416 | 7.049795 |
| IACSP95-5000 72 hpi (m.i) | 1 | 38 | UK | 7.135202 | 7.365874 |
| IACSP95-5000 72 hpi (m.i) | 1 | 39 | UK | 7.38264 | 7.613604 |
| IACSP95-5000 72 hpi (m.i) | 1 | 40 | UK | 7.586446 | 7.817702 |
| IACSP95-5000 72 hpi (m.i) | 2 | 1 | UK | -0.29557 | -0.01249 |
| IACSP95-5000 72 hpi (m.i) | 2 | 2 | UK | -0.28917 | -0.00577 |
| IACSP95-5000 72 hpi (m.i) | 2 | 3 | UK | -0.28452 | -0.0008 |
| IACSP95-5000 72 hpi (m.i) | 2 | 4 | UK | -0.28234 | 0.001694 |
| IACSP95-5000 72 hpi (m.i) | 2 | 5 | UK | -0.28166 | 0.002689 |
| IACSP95-5000 72 hpi (m.i) | 2 | 6 | UK | -0.28103 | 0.003627 |
| IACSP95-5000 72 hpi (m.i) | 2 | 7 | UK | -0.28203 | 0.002942 |
| IACSP95-5000 72 hpi (m.i) | 2 | 8 | UK | -0.28487 | 0.000417 |
| IACSP95-5000 72 hpi (m.i) | 2 | 9 | UK | -0.28519 | 0.000416 |
| IACSP95-5000 72 hpi (m.i) | 2 | 10 | UK | -0.28859 | -0.00266 |
| IACSP95-5000 72 hpi (m.i) | 2 | 11 | UK | -0.2872 | -0.00096 |
| IACSP95-5000 72 hpi (m.i) | 2 | 12 | UK | -0.29099 | -0.00444 |
| IACSP95-5000 72 hpi (m.i) | 2 | 13 | UK | -0.28931 | -0.00244 |
| IACSP95-5000 72 hpi (m.i) | 2 | 14 | UK | -0.29106 | -0.00387 |
| IACSP95-5000 72 hpi (m.i) | 2 | 15 | UK | -0.29009 | -0.00259 |
| IACSP95-5000 72 hpi (m.i) | 2 | 16 | UK | -0.29018 | -0.00236 |
| IACSP95-5000 72 hpi (m.i) | 2 | 17 | UK | -0.29083 | -0.0027 |
| IACSP95-5000 72 hpi (m.i) | 2 | 18 | UK | -0.29019 | -0.00174 |
| IACSP95-5000 72 hpi (m.i) | 2 | 19 | UK | -0.28799 | 0.000769 |
| IACSP95-5000 72 hpi (m.i) | 2 | 20 | UK | -0.28506 | 0.004016 |
| IACSP95-5000 72 hpi (m.i) | 2 | 21 | UK | -0.28138 | 0.008013 |
| IACSP95-5000 72 hpi (m.i) | 2 | 22 | UK | -0.27139 | 0.01831 |
| IACSP95-5000 72 hpi (m.i) | 2 | 23 | UK | -0.25621 | 0.03381 |
| IACSP95-5000 72 hpi (m.i) | 2 | 24 | UK | -0.22581 | 0.064528 |
| IACSP95-5000 72 hpi (m.i) | 2 | 25 | UK | -0.16393 | 0.126719 |
| IACSP95-5000 72 hpi (m.i) | 2 | 26 | UK | -0.05016 | 0.240806 |
| IACSP95-5000 72 hpi (m.i) | 2 | 27 | UK | 0.158182 | 0.449463 |
| IACSP95-5000 72 hpi (m.i) | 2 | 28 | UK | 0.540837 | 0.832433 |
| IACSP95-5000 72 hpi (m.i) | 2 | 29 | UK | 1.146702 | 1.438613 |
| IACSP95-5000 72 hpi (m.i) | 2 | 30 | UK | 1.961413 | 2.25364 |
| IACSP95-5000 72 hpi (m.i) | 2 | 31 | UK | 2.790537 | 3.083079 |
| IACSP95-5000 72 hpi (m.i) | 2 | 32 | UK | 3.519605 | 3.812462 |
| IACSP95-5000 72 hpi (m.i) | 2 | 33 | UK | 4.173478 | 4.46665 |
| IACSP95-5000 72 hpi (m.i) | 2 | 34 | UK | 4.726494 | 5.019981 |
| IACSP95-5000 72 hpi (m.i) | 2 | 35 | UK | 5.206018 | 5.499821 |
| IACSP95-5000 72 hpi (m.i) | 2 | 36 | UK | 5.595877 | 5.889995 |
| IACSP95-5000 72 hpi (m.i) | 2 | 37 | UK | 5.92186 | 6.216293 |
| IACSP95-5000 72 hpi (m.i) | 2 | 38 | UK | 6.216816 | 6.511564 |
| IACSP95-5000 72 hpi (m.i) | 2 | 39 | UK | 6.444695 | 6.739758 |
| IACSP95-5000 72 hpi (m.i) | 2 | 40 | UK | 6.638191 | 6.93357 |
| IACSP95-5000 72 hpi (m.i) | 3 | 1 | UK | -0.28888 | -0.00713 |
| IACSP95-5000 72 hpi (m.i) | 3 | 2 | UK | -0.28366 | -0.00212 |
| IACSP95-5000 72 hpi (m.i) | 3 | 3 | UK | -0.27973 | 0.001599 |
| IACSP95-5000 72 hpi (m.i) | 3 | 4 | UK | -0.27848 | 0.002631 |
| IACSP95-5000 72 hpi (m.i) | 3 | 5 | UK | -0.27699 | 0.003915 |
| IACSP95-5000 72 hpi (m.i) | 3 | 6 | UK | -0.27692 | 0.003775 |
| IACSP95-5000 72 hpi (m.i) | 3 | 7 | UK | -0.28 | 0.000482 |
| IACSP95-5000 72 hpi (m.i) | 3 | 8 | UK | -0.28145 | -0.00118 |
| IACSP95-5000 72 hpi (m.i) | 3 | 9 | UK | -0.28061 | -0.00056 |
| IACSP95-5000 72 hpi (m.i) | 3 | 10 | UK | -0.28309 | -0.00325 |
| IACSP95-5000 72 hpi (m.i) | 3 | 11 | UK | -0.2815 | -0.00187 |
| IACSP95-5000 72 hpi (m.i) | 3 | 12 | UK | -0.28404 | -0.00462 |
| IACSP95-5000 72 hpi (m.i) | 3 | 13 | UK | -0.28189 | -0.00268 |
| IACSP95-5000 72 hpi (m.i) | 3 | 14 | UK | -0.28195 | -0.00296 |
| IACSP95-5000 72 hpi (m.i) | 3 | 15 | UK | -0.28243 | -0.00364 |
| IACSP95-5000 72 hpi (m.i) | 3 | 16 | UK | -0.28131 | -0.00274 |
| IACSP95-5000 72 hpi (m.i) | 3 | 17 | UK | -0.28017 | -0.00181 |
| IACSP95-5000 72 hpi (m.i) | 3 | 18 | UK | -0.27793 | 0.000218 |
| IACSP95-5000 72 hpi (m.i) | 3 | 19 | UK | -0.27837 | -0.00043 |
| IACSP95-5000 72 hpi (m.i) | 3 | 20 | UK | -0.27413 | 0.003601 |
| IACSP95-5000 72 hpi (m.i) | 3 | 21 | UK | -0.26801 | 0.009508 |
| IACSP95-5000 72 hpi (m.i) | 3 | 22 | UK | -0.25815 | 0.019157 |
| IACSP95-5000 72 hpi (m.i) | 3 | 23 | UK | -0.238 | 0.039095 |
| IACSP95-5000 72 hpi (m.i) | 3 | 24 | UK | -0.20099 | 0.075894 |
| IACSP95-5000 72 hpi (m.i) | 3 | 25 | UK | -0.12793 | 0.148744 |
| IACSP95-5000 72 hpi (m.i) | 3 | 26 | UK | 0.013336 | 0.289795 |
| IACSP95-5000 72 hpi (m.i) | 3 | 27 | UK | 0.27207 | 0.548317 |
| IACSP95-5000 72 hpi (m.i) | 3 | 28 | UK | 0.738794 | 1.01483 |
| IACSP95-5000 72 hpi (m.i) | 3 | 29 | UK | 1.475559 | 1.751383 |
| IACSP95-5000 72 hpi (m.i) | 3 | 30 | UK | 2.458264 | 2.733876 |
| IACSP95-5000 72 hpi (m.i) | 3 | 31 | UK | 3.440664 | 3.716065 |
| IACSP95-5000 72 hpi (m.i) | 3 | 32 | UK | 4.274845 | 4.550035 |
| IACSP95-5000 72 hpi (m.i) | 3 | 33 | UK | 5.004295 | 5.279274 |
| IACSP95-5000 72 hpi (m.i) | 3 | 34 | UK | 5.611076 | 5.885842 |
| IACSP95-5000 72 hpi (m.i) | 3 | 35 | UK | 6.130046 | 6.404601 |
| IACSP95-5000 72 hpi (m.i) | 3 | 36 | UK | 6.541438 | 6.815782 |
| IACSP95-5000 72 hpi (m.i) | 3 | 37 | UK | 6.873897 | 7.148029 |
| IACSP95-5000 72 hpi (m.i) | 3 | 38 | UK | 7.184937 | 7.458858 |
| IACSP95-5000 72 hpi (m.i) | 3 | 39 | UK | 7.397099 | 7.670807 |
| IACSP95-5000 72 hpi (m.i) | 3 | 40 | UK | 7.590456 | 7.863953 |
| IACSP95-5000 72 hpi (s.i) | 1 | 1 | UK | -0.35548 | -0.00814 |
| IACSP95-5000 72 hpi (s.i) | 1 | 2 | UK | -0.35246 | -0.0051 |
| IACSP95-5000 72 hpi (s.i) | 1 | 3 | UK | -0.34922 | -0.00185 |
| IACSP95-5000 72 hpi (s.i) | 1 | 4 | UK | -0.34659 | 0.000784 |
| IACSP95-5000 72 hpi (s.i) | 1 | 5 | UK | -0.34452 | 0.002863 |
| IACSP95-5000 72 hpi (s.i) | 1 | 6 | UK | -0.34595 | 0.001449 |
| IACSP95-5000 72 hpi (s.i) | 1 | 7 | UK | -0.34764 | -0.00023 |
| IACSP95-5000 72 hpi (s.i) | 1 | 8 | UK | -0.34683 | 0.00059 |
| IACSP95-5000 72 hpi (s.i) | 1 | 9 | UK | -0.34702 | 0.00041 |
| IACSP95-5000 72 hpi (s.i) | 1 | 10 | UK | -0.34605 | 0.001388 |
| IACSP95-5000 72 hpi (s.i) | 1 | 11 | UK | -0.34732 | 0.000132 |
| IACSP95-5000 72 hpi (s.i) | 1 | 12 | UK | -0.34779 | -0.00033 |
| IACSP95-5000 72 hpi (s.i) | 1 | 13 | UK | -0.34852 | -0.00105 |
| IACSP95-5000 72 hpi (s.i) | 1 | 14 | UK | -0.34828 | -0.0008 |
| IACSP95-5000 72 hpi (s.i) | 1 | 15 | UK | -0.35148 | -0.00399 |
| IACSP95-5000 72 hpi (s.i) | 1 | 16 | UK | -0.35042 | -0.00292 |
| IACSP95-5000 72 hpi (s.i) | 1 | 17 | UK | -0.35121 | -0.0037 |
| IACSP95-5000 72 hpi (s.i) | 1 | 18 | UK | -0.3486 | -0.00108 |
| IACSP95-5000 72 hpi (s.i) | 1 | 19 | UK | -0.3481 | -0.00057 |
| IACSP95-5000 72 hpi (s.i) | 1 | 20 | UK | -0.34632 | 0.001223 |
| IACSP95-5000 72 hpi (s.i) | 1 | 21 | UK | -0.33986 | 0.00769 |
| IACSP95-5000 72 hpi (s.i) | 1 | 22 | UK | -0.33486 | 0.012699 |
| IACSP95-5000 72 hpi (s.i) | 1 | 23 | UK | -0.31968 | 0.027893 |
| IACSP95-5000 72 hpi (s.i) | 1 | 24 | UK | -0.29013 | 0.057456 |
| IACSP95-5000 72 hpi (s.i) | 1 | 25 | UK | -0.22991 | 0.117684 |
| IACSP95-5000 72 hpi (s.i) | 1 | 26 | UK | -0.1167 | 0.2309 |
| IACSP95-5000 72 hpi (s.i) | 1 | 27 | UK | 0.091928 | 0.43954 |
| IACSP95-5000 72 hpi (s.i) | 1 | 28 | UK | 0.478912 | 0.826534 |
| IACSP95-5000 72 hpi (s.i) | 1 | 29 | UK | 1.120138 | 1.467771 |
| IACSP95-5000 72 hpi (s.i) | 1 | 30 | UK | 2.042653 | 2.390295 |
| IACSP95-5000 72 hpi (s.i) | 1 | 31 | UK | 3.048853 | 3.396506 |
| IACSP95-5000 72 hpi (s.i) | 1 | 32 | UK | 3.8929 | 4.240563 |
| IACSP95-5000 72 hpi (s.i) | 1 | 33 | UK | 4.618987 | 4.96666 |
| IACSP95-5000 72 hpi (s.i) | 1 | 34 | UK | 5.234649 | 5.582333 |
| IACSP95-5000 72 hpi (s.i) | 1 | 35 | UK | 5.740205 | 6.087899 |
| IACSP95-5000 72 hpi (s.i) | 1 | 36 | UK | 6.135635 | 6.483339 |
| IACSP95-5000 72 hpi (s.i) | 1 | 37 | UK | 6.473534 | 6.821248 |
| IACSP95-5000 72 hpi (s.i) | 1 | 38 | UK | 6.75967 | 7.107394 |
| IACSP95-5000 72 hpi (s.i) | 1 | 39 | UK | 6.968937 | 7.316672 |
| IACSP95-5000 72 hpi (s.i) | 1 | 40 | UK | 7.174966 | 7.52271 |
| IACSP95-5000 72 hpi (s.i) | 2 | 1 | UK | -0.37164 | -0.02318 |
| IACSP95-5000 72 hpi (s.i) | 2 | 2 | UK | -0.36471 | -0.01586 |
| IACSP95-5000 72 hpi (s.i) | 2 | 3 | UK | -0.35507 | -0.00584 |
| IACSP95-5000 72 hpi (s.i) | 2 | 4 | UK | -0.35034 | -0.00072 |
| IACSP95-5000 72 hpi (s.i) | 2 | 5 | UK | -0.35171 | -0.0017 |
| IACSP95-5000 72 hpi (s.i) | 2 | 6 | UK | -0.35033 | 6.22E-05 |
| IACSP95-5000 72 hpi (s.i) | 2 | 7 | UK | -0.34719 | 0.003584 |
| IACSP95-5000 72 hpi (s.i) | 2 | 8 | UK | -0.34875 | 0.00241 |
| IACSP95-5000 72 hpi (s.i) | 2 | 9 | UK | -0.34658 | 0.004969 |
| IACSP95-5000 72 hpi (s.i) | 2 | 10 | UK | -0.35061 | 0.001322 |
| IACSP95-5000 72 hpi (s.i) | 2 | 11 | UK | -0.35075 | 0.001574 |
| IACSP95-5000 72 hpi (s.i) | 2 | 12 | UK | -0.35199 | 0.000722 |
| IACSP95-5000 72 hpi (s.i) | 2 | 13 | UK | -0.35003 | 0.003071 |
| IACSP95-5000 72 hpi (s.i) | 2 | 14 | UK | -0.35319 | 0.000294 |
| IACSP95-5000 72 hpi (s.i) | 2 | 15 | UK | -0.35813 | -0.00426 |
| IACSP95-5000 72 hpi (s.i) | 2 | 16 | UK | -0.35889 | -0.00463 |
| IACSP95-5000 72 hpi (s.i) | 2 | 17 | UK | -0.35705 | -0.00241 |
| IACSP95-5000 72 hpi (s.i) | 2 | 18 | UK | -0.35718 | -0.00215 |
| IACSP95-5000 72 hpi (s.i) | 2 | 19 | UK | -0.35515 | 0.000263 |
| IACSP95-5000 72 hpi (s.i) | 2 | 20 | UK | -0.35236 | 0.003446 |
| IACSP95-5000 72 hpi (s.i) | 2 | 21 | UK | -0.35417 | 0.002024 |
| IACSP95-5000 72 hpi (s.i) | 2 | 22 | UK | -0.34298 | 0.013596 |
| IACSP95-5000 72 hpi (s.i) | 2 | 23 | UK | -0.32631 | 0.030657 |
| IACSP95-5000 72 hpi (s.i) | 2 | 24 | UK | -0.2991 | 0.058252 |
| IACSP95-5000 72 hpi (s.i) | 2 | 25 | UK | -0.24537 | 0.112364 |
| IACSP95-5000 72 hpi (s.i) | 2 | 26 | UK | -0.13316 | 0.224966 |
| IACSP95-5000 72 hpi (s.i) | 2 | 27 | UK | 0.068438 | 0.426947 |
| IACSP95-5000 72 hpi (s.i) | 2 | 28 | UK | 0.443188 | 0.802084 |
| IACSP95-5000 72 hpi (s.i) | 2 | 29 | UK | 1.066801 | 1.426084 |
| IACSP95-5000 72 hpi (s.i) | 2 | 30 | UK | 1.982286 | 2.341956 |
| IACSP95-5000 72 hpi (s.i) | 2 | 31 | UK | 3.007595 | 3.367652 |
| IACSP95-5000 72 hpi (s.i) | 2 | 32 | UK | 3.881227 | 4.24167 |
| IACSP95-5000 72 hpi (s.i) | 2 | 33 | UK | 4.62635 | 4.98718 |
| IACSP95-5000 72 hpi (s.i) | 2 | 34 | UK | 5.250389 | 5.611605 |
| IACSP95-5000 72 hpi (s.i) | 2 | 35 | UK | 5.782673 | 6.144276 |
| IACSP95-5000 72 hpi (s.i) | 2 | 36 | UK | 6.196136 | 6.558125 |
| IACSP95-5000 72 hpi (s.i) | 2 | 37 | UK | 6.533612 | 6.895988 |
| IACSP95-5000 72 hpi (s.i) | 2 | 38 | UK | 6.850749 | 7.213512 |
| IACSP95-5000 72 hpi (s.i) | 2 | 39 | UK | 7.084733 | 7.447882 |
| IACSP95-5000 72 hpi (s.i) | 2 | 40 | UK | 7.282176 | 7.645712 |
| IACSP95-5000 72 hpi (s.i) | 3 | 1 | UK | -0.35653 | -0.01461 |
| IACSP95-5000 72 hpi (s.i) | 3 | 2 | UK | -0.3539 | -0.01164 |
| IACSP95-5000 72 hpi (s.i) | 3 | 3 | UK | -0.34631 | -0.00372 |
| IACSP95-5000 72 hpi (s.i) | 3 | 4 | UK | -0.3428 | 0.000128 |
| IACSP95-5000 72 hpi (s.i) | 3 | 5 | UK | -0.34477 | -0.00151 |
| IACSP95-5000 72 hpi (s.i) | 3 | 6 | UK | -0.34284 | 0.000752 |
| IACSP95-5000 72 hpi (s.i) | 3 | 7 | UK | -0.34158 | 0.002343 |
| IACSP95-5000 72 hpi (s.i) | 3 | 8 | UK | -0.34068 | 0.003572 |
| IACSP95-5000 72 hpi (s.i) | 3 | 9 | UK | -0.34143 | 0.003158 |
| IACSP95-5000 72 hpi (s.i) | 3 | 10 | UK | -0.34461 | 0.000303 |
| IACSP95-5000 72 hpi (s.i) | 3 | 11 | UK | -0.347 | -0.00175 |
| IACSP95-5000 72 hpi (s.i) | 3 | 12 | UK | -0.34621 | -0.00063 |
| IACSP95-5000 72 hpi (s.i) | 3 | 13 | UK | -0.34183 | 0.004085 |
| IACSP95-5000 72 hpi (s.i) | 3 | 14 | UK | -0.34475 | 0.001489 |
| IACSP95-5000 72 hpi (s.i) | 3 | 15 | UK | -0.34871 | -0.00214 |
| IACSP95-5000 72 hpi (s.i) | 3 | 16 | UK | -0.35401 | -0.00711 |
| IACSP95-5000 72 hpi (s.i) | 3 | 17 | UK | -0.35004 | -0.0028 |
| IACSP95-5000 72 hpi (s.i) | 3 | 18 | UK | -0.34894 | -0.00137 |
| IACSP95-5000 72 hpi (s.i) | 3 | 19 | UK | -0.34799 | -8.9E-05 |
| IACSP95-5000 72 hpi (s.i) | 3 | 20 | UK | -0.34704 | 0.001196 |
| IACSP95-5000 72 hpi (s.i) | 3 | 21 | UK | -0.34447 | 0.004098 |
| IACSP95-5000 72 hpi (s.i) | 3 | 22 | UK | -0.33688 | 0.012023 |
| IACSP95-5000 72 hpi (s.i) | 3 | 23 | UK | -0.31966 | 0.029567 |
| IACSP95-5000 72 hpi (s.i) | 3 | 24 | UK | -0.29185 | 0.05771 |
| IACSP95-5000 72 hpi (s.i) | 3 | 25 | UK | -0.2333 | 0.116597 |
| IACSP95-5000 72 hpi (s.i) | 3 | 26 | UK | -0.11865 | 0.231578 |
| IACSP95-5000 72 hpi (s.i) | 3 | 27 | UK | 0.089602 | 0.44016 |
| IACSP95-5000 72 hpi (s.i) | 3 | 28 | UK | 0.477813 | 0.828703 |
| IACSP95-5000 72 hpi (s.i) | 3 | 29 | UK | 1.120921 | 1.472143 |
| IACSP95-5000 72 hpi (s.i) | 3 | 30 | UK | 2.076843 | 2.428396 |
| IACSP95-5000 72 hpi (s.i) | 3 | 31 | UK | 3.150166 | 3.502051 |
| IACSP95-5000 72 hpi (s.i) | 3 | 32 | UK | 4.065973 | 4.41819 |
| IACSP95-5000 72 hpi (s.i) | 3 | 33 | UK | 4.846574 | 5.199122 |
| IACSP95-5000 72 hpi (s.i) | 3 | 34 | UK | 5.489919 | 5.8428 |
| IACSP95-5000 72 hpi (s.i) | 3 | 35 | UK | 6.036863 | 6.390076 |
| IACSP95-5000 72 hpi (s.i) | 3 | 36 | UK | 6.46353 | 6.817074 |
| IACSP95-5000 72 hpi (s.i) | 3 | 37 | UK | 6.817079 | 7.170955 |
| IACSP95-5000 72 hpi (s.i) | 3 | 38 | UK | 7.118461 | 7.472669 |
| IACSP95-5000 72 hpi (s.i) | 3 | 39 | UK | 7.361595 | 7.716135 |
| IACSP95-5000 72 hpi (s.i) | 3 | 40 | UK | 7.563751 | 7.918622 |
| IAC91-1099 24 hpi (m.i) | 1 | 1 | ACT | -0.11085 | 0.014555 |
| IAC91-1099 24 hpi (m.i) | 1 | 2 | ACT | -0.12031 | 0.006488 |
| IAC91-1099 24 hpi (m.i) | 1 | 3 | ACT | -0.12715 | 0.001044 |
| IAC91-1099 24 hpi (m.i) | 1 | 4 | ACT | -0.13076 | -0.00117 |
| IAC91-1099 24 hpi (m.i) | 1 | 5 | ACT | -0.13319 | -0.0022 |
| IAC91-1099 24 hpi (m.i) | 1 | 6 | ACT | -0.13273 | -0.00034 |
| IAC91-1099 24 hpi (m.i) | 1 | 7 | ACT | -0.13538 | -0.00159 |
| IAC91-1099 24 hpi (m.i) | 1 | 8 | ACT | -0.13321 | 0.00198 |
| IAC91-1099 24 hpi (m.i) | 1 | 9 | ACT | -0.13431 | 0.002275 |
| IAC91-1099 24 hpi (m.i) | 1 | 10 | ACT | -0.1347 | 0.003288 |
| IAC91-1099 24 hpi (m.i) | 1 | 11 | ACT | -0.13911 | 0.000271 |
| IAC91-1099 24 hpi (m.i) | 1 | 12 | ACT | -0.13827 | 0.002507 |
| IAC91-1099 24 hpi (m.i) | 1 | 13 | ACT | -0.14252 | -0.00034 |
| IAC91-1099 24 hpi (m.i) | 1 | 14 | ACT | -0.14256 | 0.001014 |
| IAC91-1099 24 hpi (m.i) | 1 | 15 | ACT | -0.14467 | 0.000301 |
| IAC91-1099 24 hpi (m.i) | 1 | 16 | ACT | -0.14769 | -0.00132 |
| IAC91-1099 24 hpi (m.i) | 1 | 17 | ACT | -0.14998 | -0.00221 |
| IAC91-1099 24 hpi (m.i) | 1 | 18 | ACT | -0.1506 | -0.00143 |
| IAC91-1099 24 hpi (m.i) | 1 | 19 | ACT | -0.15231 | -0.00174 |
| IAC91-1099 24 hpi (m.i) | 1 | 20 | ACT | -0.15525 | -0.00328 |
| IAC91-1099 24 hpi (m.i) | 1 | 21 | ACT | -0.15506 | -0.00169 |
| IAC91-1099 24 hpi (m.i) | 1 | 22 | ACT | -0.15847 | -0.00371 |
| IAC91-1099 24 hpi (m.i) | 1 | 23 | ACT | -0.15773 | -0.00157 |
| IAC91-1099 24 hpi (m.i) | 1 | 24 | ACT | -0.15533 | 0.002226 |
| IAC91-1099 24 hpi (m.i) | 1 | 25 | ACT | -0.15126 | 0.007692 |
| IAC91-1099 24 hpi (m.i) | 1 | 26 | ACT | -0.14633 | 0.014023 |
| IAC91-1099 24 hpi (m.i) | 1 | 27 | ACT | -0.12889 | 0.032866 |
| IAC91-1099 24 hpi (m.i) | 1 | 28 | ACT | -0.09487 | 0.068277 |
| IAC91-1099 24 hpi (m.i) | 1 | 29 | ACT | -0.0278 | 0.136746 |
| IAC91-1099 24 hpi (m.i) | 1 | 30 | ACT | 0.100817 | 0.266764 |
| IAC91-1099 24 hpi (m.i) | 1 | 31 | ACT | 0.332298 | 0.499643 |
| IAC91-1099 24 hpi (m.i) | 1 | 32 | ACT | 0.723359 | 0.892102 |
| IAC91-1099 24 hpi (m.i) | 1 | 33 | ACT | 1.282289 | 1.45243 |
| IAC91-1099 24 hpi (m.i) | 1 | 34 | ACT | 1.940357 | 2.111896 |
| IAC91-1099 24 hpi (m.i) | 1 | 35 | ACT | 2.583981 | 2.756917 |
| IAC91-1099 24 hpi (m.i) | 1 | 36 | ACT | 3.226234 | 3.400569 |
| IAC91-1099 24 hpi (m.i) | 1 | 37 | ACT | 3.855246 | 4.030979 |
| IAC91-1099 24 hpi (m.i) | 1 | 38 | ACT | 4.347792 | 4.524923 |
| IAC91-1099 24 hpi (m.i) | 1 | 39 | ACT | 4.799221 | 4.97775 |
| IAC91-1099 24 hpi (m.i) | 1 | 40 | ACT | 5.176066 | 5.355994 |
| IAC91-1099 24 hpi (m.i) | 2 | 1 | ACT | -0.06624 | 0.016667 |
| IAC91-1099 24 hpi (m.i) | 2 | 2 | ACT | -0.0805 | 0.003846 |
| IAC91-1099 24 hpi (m.i) | 2 | 3 | ACT | -0.08552 | 0.000262 |
| IAC91-1099 24 hpi (m.i) | 2 | 4 | ACT | -0.08679 | 0.000437 |
| IAC91-1099 24 hpi (m.i) | 2 | 5 | ACT | -0.08747 | 0.001192 |
| IAC91-1099 24 hpi (m.i) | 2 | 6 | ACT | -0.08757 | 0.002532 |
| IAC91-1099 24 hpi (m.i) | 2 | 7 | ACT | -0.09039 | 0.001149 |
| IAC91-1099 24 hpi (m.i) | 2 | 8 | ACT | -0.09202 | 0.00095 |
| IAC91-1099 24 hpi (m.i) | 2 | 9 | ACT | -0.09474 | -0.00033 |
| IAC91-1099 24 hpi (m.i) | 2 | 10 | ACT | -0.09486 | 0.000985 |
| IAC91-1099 24 hpi (m.i) | 2 | 11 | ACT | -0.09888 | -0.00159 |
| IAC91-1099 24 hpi (m.i) | 2 | 12 | ACT | -0.0986 | 0.000122 |
| IAC91-1099 24 hpi (m.i) | 2 | 13 | ACT | -0.09991 | 0.00025 |
| IAC91-1099 24 hpi (m.i) | 2 | 14 | ACT | -0.10387 | -0.00227 |
| IAC91-1099 24 hpi (m.i) | 2 | 15 | ACT | -0.10426 | -0.00122 |
| IAC91-1099 24 hpi (m.i) | 2 | 16 | ACT | -0.10688 | -0.00241 |
| IAC91-1099 24 hpi (m.i) | 2 | 17 | ACT | -0.10788 | -0.00197 |
| IAC91-1099 24 hpi (m.i) | 2 | 18 | ACT | -0.10758 | -0.00023 |
| IAC91-1099 24 hpi (m.i) | 2 | 19 | ACT | -0.11116 | -0.00237 |
| IAC91-1099 24 hpi (m.i) | 2 | 20 | ACT | -0.11289 | -0.00266 |
| IAC91-1099 24 hpi (m.i) | 2 | 21 | ACT | -0.11354 | -0.00188 |
| IAC91-1099 24 hpi (m.i) | 2 | 22 | ACT | -0.11283 | 0.000271 |
| IAC91-1099 24 hpi (m.i) | 2 | 23 | ACT | -0.11453 | 1.3E-05 |
| IAC91-1099 24 hpi (m.i) | 2 | 24 | ACT | -0.11529 | 0.00069 |
| IAC91-1099 24 hpi (m.i) | 2 | 25 | ACT | -0.11507 | 0.002341 |
| IAC91-1099 24 hpi (m.i) | 2 | 26 | ACT | -0.11311 | 0.005742 |
| IAC91-1099 24 hpi (m.i) | 2 | 27 | ACT | -0.10494 | 0.015354 |
| IAC91-1099 24 hpi (m.i) | 2 | 28 | ACT | -0.09201 | 0.02972 |
| IAC91-1099 24 hpi (m.i) | 2 | 29 | ACT | -0.0599 | 0.063264 |
| IAC91-1099 24 hpi (m.i) | 2 | 30 | ACT | 0.001016 | 0.125618 |
| IAC91-1099 24 hpi (m.i) | 2 | 31 | ACT | 0.113505 | 0.239545 |
| IAC91-1099 24 hpi (m.i) | 2 | 32 | ACT | 0.326608 | 0.454085 |
| IAC91-1099 24 hpi (m.i) | 2 | 33 | ACT | 0.696629 | 0.825545 |
| IAC91-1099 24 hpi (m.i) | 2 | 34 | ACT | 1.286395 | 1.416748 |
| IAC91-1099 24 hpi (m.i) | 2 | 35 | ACT | 1.998585 | 2.130376 |
| IAC91-1099 24 hpi (m.i) | 2 | 36 | ACT | 2.715542 | 2.848771 |
| IAC91-1099 24 hpi (m.i) | 2 | 37 | ACT | 3.470454 | 3.60512 |
| IAC91-1099 24 hpi (m.i) | 2 | 38 | ACT | 4.117904 | 4.254007 |
| IAC91-1099 24 hpi (m.i) | 2 | 39 | ACT | 4.709262 | 4.846803 |
| IAC91-1099 24 hpi (m.i) | 2 | 40 | ACT | 5.224518 | 5.363497 |
| IAC91-1099 24 hpi (m.i) | 3 | 1 | ACT | -0.00403 | 0.02811 |
| IAC91-1099 24 hpi (m.i) | 3 | 2 | ACT | -0.01933 | 0.013364 |
| IAC91-1099 24 hpi (m.i) | 3 | 3 | ACT | -0.02601 | 0.007236 |
| IAC91-1099 24 hpi (m.i) | 3 | 4 | ACT | -0.02822 | 0.005573 |
| IAC91-1099 24 hpi (m.i) | 3 | 5 | ACT | -0.03262 | 0.001728 |
| IAC91-1099 24 hpi (m.i) | 3 | 6 | ACT | -0.03336 | 0.001542 |
| IAC91-1099 24 hpi (m.i) | 3 | 7 | ACT | -0.03743 | -0.00199 |
| IAC91-1099 24 hpi (m.i) | 3 | 8 | ACT | -0.03639 | -0.00039 |
| IAC91-1099 24 hpi (m.i) | 3 | 9 | ACT | -0.03552 | 0.001025 |
| IAC91-1099 24 hpi (m.i) | 3 | 10 | ACT | -0.03769 | -0.00059 |
| IAC91-1099 24 hpi (m.i) | 3 | 11 | ACT | -0.04172 | -0.00407 |
| IAC91-1099 24 hpi (m.i) | 3 | 12 | ACT | -0.0407 | -0.0025 |
| IAC91-1099 24 hpi (m.i) | 3 | 13 | ACT | -0.04428 | -0.00552 |
| IAC91-1099 24 hpi (m.i) | 3 | 14 | ACT | -0.04196 | -0.00266 |
| IAC91-1099 24 hpi (m.i) | 3 | 15 | ACT | -0.0431 | -0.00325 |
| IAC91-1099 24 hpi (m.i) | 3 | 16 | ACT | -0.0431 | -0.00269 |
| IAC91-1099 24 hpi (m.i) | 3 | 17 | ACT | -0.04461 | -0.00365 |
| IAC91-1099 24 hpi (m.i) | 3 | 18 | ACT | -0.04328 | -0.00177 |
| IAC91-1099 24 hpi (m.i) | 3 | 19 | ACT | -0.04391 | -0.00185 |
| IAC91-1099 24 hpi (m.i) | 3 | 20 | ACT | -0.04576 | -0.00315 |
| IAC91-1099 24 hpi (m.i) | 3 | 21 | ACT | -0.04322 | -5.7E-05 |
| IAC91-1099 24 hpi (m.i) | 3 | 22 | ACT | -0.04406 | -0.00035 |
| IAC91-1099 24 hpi (m.i) | 3 | 23 | ACT | -0.04411 | 0.000147 |
| IAC91-1099 24 hpi (m.i) | 3 | 24 | ACT | -0.03884 | 0.005971 |
| IAC91-1099 24 hpi (m.i) | 3 | 25 | ACT | -0.03409 | 0.011275 |
| IAC91-1099 24 hpi (m.i) | 3 | 26 | ACT | -0.02646 | 0.019452 |
| IAC91-1099 24 hpi (m.i) | 3 | 27 | ACT | -0.001 | 0.045467 |
| IAC91-1099 24 hpi (m.i) | 3 | 28 | ACT | 0.03723 | 0.084247 |
| IAC91-1099 24 hpi (m.i) | 3 | 29 | ACT | 0.11197 | 0.159537 |
| IAC91-1099 24 hpi (m.i) | 3 | 30 | ACT | 0.253163 | 0.301281 |
| IAC91-1099 24 hpi (m.i) | 3 | 31 | ACT | 0.514852 | 0.563521 |
| IAC91-1099 24 hpi (m.i) | 3 | 32 | ACT | 0.952704 | 1.001924 |
| IAC91-1099 24 hpi (m.i) | 3 | 33 | ACT | 1.605103 | 1.654873 |
| IAC91-1099 24 hpi (m.i) | 3 | 34 | ACT | 2.386044 | 2.436365 |
| IAC91-1099 24 hpi (m.i) | 3 | 35 | ACT | 3.126098 | 3.176971 |
| IAC91-1099 24 hpi (m.i) | 3 | 36 | ACT | 3.834133 | 3.885556 |
| IAC91-1099 24 hpi (m.i) | 3 | 37 | ACT | 4.517784 | 4.569758 |
| IAC91-1099 24 hpi (m.i) | 3 | 38 | ACT | 5.043056 | 5.095581 |
| IAC91-1099 24 hpi (m.i) | 3 | 39 | ACT | 5.518281 | 5.571357 |
| IAC91-1099 24 hpi (m.i) | 3 | 40 | ACT | 5.907942 | 5.961569 |
| IAC91-1099 24 hpi (s.i) | 1 | 1 | ACT | -0.29196 | -0.00984 |
| IAC91-1099 24 hpi (s.i) | 1 | 2 | ACT | -0.2916 | -0.00951 |
| IAC91-1099 24 hpi (s.i) | 1 | 3 | ACT | -0.28609 | -0.00403 |
| IAC91-1099 24 hpi (s.i) | 1 | 4 | ACT | -0.28538 | -0.00336 |
| IAC91-1099 24 hpi (s.i) | 1 | 5 | ACT | -0.28276 | -0.00076 |
| IAC91-1099 24 hpi (s.i) | 1 | 6 | ACT | -0.28065 | 0.001314 |
| IAC91-1099 24 hpi (s.i) | 1 | 7 | ACT | -0.2798 | 0.002135 |
| IAC91-1099 24 hpi (s.i) | 1 | 8 | ACT | -0.28003 | 0.00187 |
| IAC91-1099 24 hpi (s.i) | 1 | 9 | ACT | -0.27973 | 0.002143 |
| IAC91-1099 24 hpi (s.i) | 1 | 10 | ACT | -0.28093 | 0.00091 |
| IAC91-1099 24 hpi (s.i) | 1 | 11 | ACT | -0.28061 | 0.001198 |
| IAC91-1099 24 hpi (s.i) | 1 | 12 | ACT | -0.2803 | 0.001481 |
| IAC91-1099 24 hpi (s.i) | 1 | 13 | ACT | -0.28051 | 0.001231 |
| IAC91-1099 24 hpi (s.i) | 1 | 14 | ACT | -0.28043 | 0.001285 |
| IAC91-1099 24 hpi (s.i) | 1 | 15 | ACT | -0.28257 | -0.00089 |
| IAC91-1099 24 hpi (s.i) | 1 | 16 | ACT | -0.28175 | -9.6E-05 |
| IAC91-1099 24 hpi (s.i) | 1 | 17 | ACT | -0.28197 | -0.00035 |
| IAC91-1099 24 hpi (s.i) | 1 | 18 | ACT | -0.28173 | -0.00014 |
| IAC91-1099 24 hpi (s.i) | 1 | 19 | ACT | -0.28311 | -0.00155 |
| IAC91-1099 24 hpi (s.i) | 1 | 20 | ACT | -0.28268 | -0.00115 |
| IAC91-1099 24 hpi (s.i) | 1 | 21 | ACT | -0.28218 | -0.00069 |
| IAC91-1099 24 hpi (s.i) | 1 | 22 | ACT | -0.2827 | -0.00124 |
| IAC91-1099 24 hpi (s.i) | 1 | 23 | ACT | -0.28075 | 0.000687 |
| IAC91-1099 24 hpi (s.i) | 1 | 24 | ACT | -0.27563 | 0.00577 |
| IAC91-1099 24 hpi (s.i) | 1 | 25 | ACT | -0.26549 | 0.01588 |
| IAC91-1099 24 hpi (s.i) | 1 | 26 | ACT | -0.24471 | 0.036627 |
| IAC91-1099 24 hpi (s.i) | 1 | 27 | ACT | -0.2006 | 0.080709 |
| IAC91-1099 24 hpi (s.i) | 1 | 28 | ACT | -0.11784 | 0.163439 |
| IAC91-1099 24 hpi (s.i) | 1 | 29 | ACT | 0.038299 | 0.319544 |
| IAC91-1099 24 hpi (s.i) | 1 | 30 | ACT | 0.325978 | 0.607192 |
| IAC91-1099 24 hpi (s.i) | 1 | 31 | ACT | 0.798825 | 1.080008 |
| IAC91-1099 24 hpi (s.i) | 1 | 32 | ACT | 1.481329 | 1.76248 |
| IAC91-1099 24 hpi (s.i) | 1 | 33 | ACT | 2.239762 | 2.520882 |
| IAC91-1099 24 hpi (s.i) | 1 | 34 | ACT | 2.999982 | 3.281071 |
| IAC91-1099 24 hpi (s.i) | 1 | 35 | ACT | 3.702888 | 3.983946 |
| IAC91-1099 24 hpi (s.i) | 1 | 36 | ACT | 4.343896 | 4.624922 |
| IAC91-1099 24 hpi (s.i) | 1 | 37 | ACT | 4.925743 | 5.206738 |
| IAC91-1099 24 hpi (s.i) | 1 | 38 | ACT | 5.360191 | 5.641154 |
| IAC91-1099 24 hpi (s.i) | 1 | 39 | ACT | 5.749349 | 6.030282 |
| IAC91-1099 24 hpi (s.i) | 1 | 40 | ACT | 6.085677 | 6.366578 |
| IAC91-1099 24 hpi (s.i) | 2 | 1 | ACT | -0.29148 | -0.00532 |
| IAC91-1099 24 hpi (s.i) | 2 | 2 | ACT | -0.29318 | -0.00638 |
| IAC91-1099 24 hpi (s.i) | 2 | 3 | ACT | -0.29081 | -0.00337 |
| IAC91-1099 24 hpi (s.i) | 2 | 4 | ACT | -0.28865 | -0.00057 |
| IAC91-1099 24 hpi (s.i) | 2 | 5 | ACT | -0.28977 | -0.00105 |
| IAC91-1099 24 hpi (s.i) | 2 | 6 | ACT | -0.28769 | 0.001659 |
| IAC91-1099 24 hpi (s.i) | 2 | 7 | ACT | -0.28855 | 0.00144 |
| IAC91-1099 24 hpi (s.i) | 2 | 8 | ACT | -0.29005 | 0.000571 |
| IAC91-1099 24 hpi (s.i) | 2 | 9 | ACT | -0.28912 | 0.002142 |
| IAC91-1099 24 hpi (s.i) | 2 | 10 | ACT | -0.29094 | 0.000961 |
| IAC91-1099 24 hpi (s.i) | 2 | 11 | ACT | -0.29233 | 0.000207 |
| IAC91-1099 24 hpi (s.i) | 2 | 12 | ACT | -0.29183 | 0.001342 |
| IAC91-1099 24 hpi (s.i) | 2 | 13 | ACT | -0.2934 | 0.000406 |
| IAC91-1099 24 hpi (s.i) | 2 | 14 | ACT | -0.29399 | 0.000454 |
| IAC91-1099 24 hpi (s.i) | 2 | 15 | ACT | -0.29583 | -0.00075 |
| IAC91-1099 24 hpi (s.i) | 2 | 16 | ACT | -0.29637 | -0.00065 |
| IAC91-1099 24 hpi (s.i) | 2 | 17 | ACT | -0.29653 | -0.00018 |
| IAC91-1099 24 hpi (s.i) | 2 | 18 | ACT | -0.29808 | -0.00109 |
| IAC91-1099 24 hpi (s.i) | 2 | 19 | ACT | -0.2993 | -0.00168 |
| IAC91-1099 24 hpi (s.i) | 2 | 20 | ACT | -0.29971 | -0.00145 |
| IAC91-1099 24 hpi (s.i) | 2 | 21 | ACT | -0.30004 | -0.00114 |
| IAC91-1099 24 hpi (s.i) | 2 | 22 | ACT | -0.29952 | 1.36E-05 |
| IAC91-1099 24 hpi (s.i) | 2 | 23 | ACT | -0.29743 | 0.002738 |
| IAC91-1099 24 hpi (s.i) | 2 | 24 | ACT | -0.29374 | 0.007063 |
| IAC91-1099 24 hpi (s.i) | 2 | 25 | ACT | -0.28345 | 0.017989 |
| IAC91-1099 24 hpi (s.i) | 2 | 26 | ACT | -0.26413 | 0.037946 |
| IAC91-1099 24 hpi (s.i) | 2 | 27 | ACT | -0.2228 | 0.079917 |
| IAC91-1099 24 hpi (s.i) | 2 | 28 | ACT | -0.14742 | 0.155928 |
| IAC91-1099 24 hpi (s.i) | 2 | 29 | ACT | -0.00067 | 0.303324 |
| IAC91-1099 24 hpi (s.i) | 2 | 30 | ACT | 0.270305 | 0.574931 |
| IAC91-1099 24 hpi (s.i) | 2 | 31 | ACT | 0.726577 | 1.031839 |
| IAC91-1099 24 hpi (s.i) | 2 | 32 | ACT | 1.402921 | 1.70882 |
| IAC91-1099 24 hpi (s.i) | 2 | 33 | ACT | 2.182005 | 2.488541 |
| IAC91-1099 24 hpi (s.i) | 2 | 34 | ACT | 2.968575 | 3.275747 |
| IAC91-1099 24 hpi (s.i) | 2 | 35 | ACT | 3.696868 | 4.004676 |
| IAC91-1099 24 hpi (s.i) | 2 | 36 | ACT | 4.366066 | 4.674511 |
| IAC91-1099 24 hpi (s.i) | 2 | 37 | ACT | 4.97012 | 5.279202 |
| IAC91-1099 24 hpi (s.i) | 2 | 38 | ACT | 5.429615 | 5.739333 |
| IAC91-1099 24 hpi (s.i) | 2 | 39 | ACT | 5.838683 | 6.149037 |
| IAC91-1099 24 hpi (s.i) | 2 | 40 | ACT | 6.184183 | 6.495173 |
| IAC91-1099 24 hpi (s.i) | 3 | 1 | ACT | -0.27569 | -0.00935 |
| IAC91-1099 24 hpi (s.i) | 3 | 2 | ACT | -0.27055 | -0.00385 |
| IAC91-1099 24 hpi (s.i) | 3 | 3 | ACT | -0.26832 | -0.00126 |
| IAC91-1099 24 hpi (s.i) | 3 | 4 | ACT | -0.26693 | 0.000488 |
| IAC91-1099 24 hpi (s.i) | 3 | 5 | ACT | -0.26852 | -0.00074 |
| IAC91-1099 24 hpi (s.i) | 3 | 6 | ACT | -0.26777 | 0.000372 |
| IAC91-1099 24 hpi (s.i) | 3 | 7 | ACT | -0.26697 | 0.001535 |
| IAC91-1099 24 hpi (s.i) | 3 | 8 | ACT | -0.2667 | 0.002168 |
| IAC91-1099 24 hpi (s.i) | 3 | 9 | ACT | -0.26817 | 0.001066 |
| IAC91-1099 24 hpi (s.i) | 3 | 10 | ACT | -0.26882 | 0.000774 |
| IAC91-1099 24 hpi (s.i) | 3 | 11 | ACT | -0.26945 | 0.000506 |
| IAC91-1099 24 hpi (s.i) | 3 | 12 | ACT | -0.26962 | 0.000702 |
| IAC91-1099 24 hpi (s.i) | 3 | 13 | ACT | -0.27217 | -0.00148 |
| IAC91-1099 24 hpi (s.i) | 3 | 14 | ACT | -0.27242 | -0.00138 |
| IAC91-1099 24 hpi (s.i) | 3 | 15 | ACT | -0.27232 | -0.00092 |
| IAC91-1099 24 hpi (s.i) | 3 | 16 | ACT | -0.27279 | -0.00103 |
| IAC91-1099 24 hpi (s.i) | 3 | 17 | ACT | -0.27361 | -0.00148 |
| IAC91-1099 24 hpi (s.i) | 3 | 18 | ACT | -0.27398 | -0.00149 |
| IAC91-1099 24 hpi (s.i) | 3 | 19 | ACT | -0.27469 | -0.00184 |
| IAC91-1099 24 hpi (s.i) | 3 | 20 | ACT | -0.27372 | -0.0005 |
| IAC91-1099 24 hpi (s.i) | 3 | 21 | ACT | -0.27395 | -0.00037 |
| IAC91-1099 24 hpi (s.i) | 3 | 22 | ACT | -0.27472 | -0.00078 |
| IAC91-1099 24 hpi (s.i) | 3 | 23 | ACT | -0.27329 | 0.001012 |
| IAC91-1099 24 hpi (s.i) | 3 | 24 | ACT | -0.27004 | 0.004624 |
| IAC91-1099 24 hpi (s.i) | 3 | 25 | ACT | -0.26134 | 0.013686 |
| IAC91-1099 24 hpi (s.i) | 3 | 26 | ACT | -0.24605 | 0.02934 |
| IAC91-1099 24 hpi (s.i) | 3 | 27 | ACT | -0.21578 | 0.05997 |
| IAC91-1099 24 hpi (s.i) | 3 | 28 | ACT | -0.15398 | 0.122133 |
| IAC91-1099 24 hpi (s.i) | 3 | 29 | ACT | -0.04216 | 0.234313 |
| IAC91-1099 24 hpi (s.i) | 3 | 30 | ACT | 0.173968 | 0.450807 |
| IAC91-1099 24 hpi (s.i) | 3 | 31 | ACT | 0.546564 | 0.823765 |
| IAC91-1099 24 hpi (s.i) | 3 | 32 | ACT | 1.149175 | 1.426738 |
| IAC91-1099 24 hpi (s.i) | 3 | 33 | ACT | 1.932492 | 2.210417 |
| IAC91-1099 24 hpi (s.i) | 3 | 34 | ACT | 2.762426 | 3.040713 |
| IAC91-1099 24 hpi (s.i) | 3 | 35 | ACT | 3.521916 | 3.800565 |
| IAC91-1099 24 hpi (s.i) | 3 | 36 | ACT | 4.23385 | 4.512862 |
| IAC91-1099 24 hpi (s.i) | 3 | 37 | ACT | 4.880948 | 5.160321 |
| IAC91-1099 24 hpi (s.i) | 3 | 38 | ACT | 5.382754 | 5.66249 |
| IAC91-1099 24 hpi (s.i) | 3 | 39 | ACT | 5.816358 | 6.096456 |
| IAC91-1099 24 hpi (s.i) | 3 | 40 | ACT | 6.182602 | 6.463063 |
| IAC91-1099 72 hpi (m.i) | 1 | 1 | ACT | -0.32905 | -0.01274 |
| IAC91-1099 72 hpi (m.i) | 1 | 2 | ACT | -0.32642 | -0.00999 |
| IAC91-1099 72 hpi (m.i) | 1 | 3 | ACT | -0.32143 | -0.00487 |
| IAC91-1099 72 hpi (m.i) | 1 | 4 | ACT | -0.31889 | -0.0022 |
| IAC91-1099 72 hpi (m.i) | 1 | 5 | ACT | -0.31675 | 7.63E-05 |
| IAC91-1099 72 hpi (m.i) | 1 | 6 | ACT | -0.31735 | -0.0004 |
| IAC91-1099 72 hpi (m.i) | 1 | 7 | ACT | -0.31598 | 0.001103 |
| IAC91-1099 72 hpi (m.i) | 1 | 8 | ACT | -0.31597 | 0.001241 |
| IAC91-1099 72 hpi (m.i) | 1 | 9 | ACT | -0.31632 | 0.001018 |
| IAC91-1099 72 hpi (m.i) | 1 | 10 | ACT | -0.31515 | 0.002319 |
| IAC91-1099 72 hpi (m.i) | 1 | 11 | ACT | -0.3161 | 0.001506 |
| IAC91-1099 72 hpi (m.i) | 1 | 12 | ACT | -0.31571 | 0.002017 |
| IAC91-1099 72 hpi (m.i) | 1 | 13 | ACT | -0.31572 | 0.002138 |
| IAC91-1099 72 hpi (m.i) | 1 | 14 | ACT | -0.31761 | 0.000379 |
| IAC91-1099 72 hpi (m.i) | 1 | 15 | ACT | -0.31685 | 0.001273 |
| IAC91-1099 72 hpi (m.i) | 1 | 16 | ACT | -0.31761 | 0.000644 |
| IAC91-1099 72 hpi (m.i) | 1 | 17 | ACT | -0.31796 | 0.000422 |
| IAC91-1099 72 hpi (m.i) | 1 | 18 | ACT | -0.31946 | -0.00095 |
| IAC91-1099 72 hpi (m.i) | 1 | 19 | ACT | -0.31936 | -0.00072 |
| IAC91-1099 72 hpi (m.i) | 1 | 20 | ACT | -0.31906 | -0.00029 |
| IAC91-1099 72 hpi (m.i) | 1 | 21 | ACT | -0.32042 | -0.00152 |
| IAC91-1099 72 hpi (m.i) | 1 | 22 | ACT | -0.32164 | -0.00261 |
| IAC91-1099 72 hpi (m.i) | 1 | 23 | ACT | -0.32123 | -0.00208 |
| IAC91-1099 72 hpi (m.i) | 1 | 24 | ACT | -0.32065 | -0.00136 |
| IAC91-1099 72 hpi (m.i) | 1 | 25 | ACT | -0.31656 | 0.002853 |
| IAC91-1099 72 hpi (m.i) | 1 | 26 | ACT | -0.31016 | 0.009384 |
| IAC91-1099 72 hpi (m.i) | 1 | 27 | ACT | -0.29887 | 0.020806 |
| IAC91-1099 72 hpi (m.i) | 1 | 28 | ACT | -0.27421 | 0.045595 |
| IAC91-1099 72 hpi (m.i) | 1 | 29 | ACT | -0.22721 | 0.092724 |
| IAC91-1099 72 hpi (m.i) | 1 | 30 | ACT | -0.13177 | 0.188294 |
| IAC91-1099 72 hpi (m.i) | 1 | 31 | ACT | 0.04191 | 0.362104 |
| IAC91-1099 72 hpi (m.i) | 1 | 32 | ACT | 0.356516 | 0.676839 |
| IAC91-1099 72 hpi (m.i) | 1 | 33 | ACT | 0.874856 | 1.195308 |
| IAC91-1099 72 hpi (m.i) | 1 | 34 | ACT | 1.600738 | 1.921321 |
| IAC91-1099 72 hpi (m.i) | 1 | 35 | ACT | 2.377791 | 2.698503 |
| IAC91-1099 72 hpi (m.i) | 1 | 36 | ACT | 3.118893 | 3.439734 |
| IAC91-1099 72 hpi (m.i) | 1 | 37 | ACT | 3.844338 | 4.165309 |
| IAC91-1099 72 hpi (m.i) | 1 | 38 | ACT | 4.445265 | 4.766366 |
| IAC91-1099 72 hpi (m.i) | 1 | 39 | ACT | 4.980628 | 5.301858 |
| IAC91-1099 72 hpi (m.i) | 1 | 40 | ACT | 5.429208 | 5.750568 |
| IAC91-1099 72 hpi (m.i) | 2 | 1 | ACT | -0.30902 | -0.00768 |
| IAC91-1099 72 hpi (m.i) | 2 | 2 | ACT | -0.30363 | -0.00237 |
| IAC91-1099 72 hpi (m.i) | 2 | 3 | ACT | -0.30298 | -0.00181 |
| IAC91-1099 72 hpi (m.i) | 2 | 4 | ACT | -0.29999 | 0.001107 |
| IAC91-1099 72 hpi (m.i) | 2 | 5 | ACT | -0.3002 | 0.000813 |
| IAC91-1099 72 hpi (m.i) | 2 | 6 | ACT | -0.29981 | 0.001118 |
| IAC91-1099 72 hpi (m.i) | 2 | 7 | ACT | -0.29922 | 0.00163 |
| IAC91-1099 72 hpi (m.i) | 2 | 8 | ACT | -0.30033 | 0.000435 |
| IAC91-1099 72 hpi (m.i) | 2 | 9 | ACT | -0.29946 | 0.001229 |
| IAC91-1099 72 hpi (m.i) | 2 | 10 | ACT | -0.2998 | 0.000798 |
| IAC91-1099 72 hpi (m.i) | 2 | 11 | ACT | -0.30115 | -0.00063 |
| IAC91-1099 72 hpi (m.i) | 2 | 12 | ACT | -0.30162 | -0.00118 |
| IAC91-1099 72 hpi (m.i) | 2 | 13 | ACT | -0.30217 | -0.00181 |
| IAC91-1099 72 hpi (m.i) | 2 | 14 | ACT | -0.29931 | 0.000965 |
| IAC91-1099 72 hpi (m.i) | 2 | 15 | ACT | -0.30106 | -0.00087 |
| IAC91-1099 72 hpi (m.i) | 2 | 16 | ACT | -0.30129 | -0.00118 |
| IAC91-1099 72 hpi (m.i) | 2 | 17 | ACT | -0.30271 | -0.00268 |
| IAC91-1099 72 hpi (m.i) | 2 | 18 | ACT | -0.3001 | -0.00015 |
| IAC91-1099 72 hpi (m.i) | 2 | 19 | ACT | -0.29949 | 0.000375 |
| IAC91-1099 72 hpi (m.i) | 2 | 20 | ACT | -0.30183 | -0.00204 |
| IAC91-1099 72 hpi (m.i) | 2 | 21 | ACT | -0.30081 | -0.0011 |
| IAC91-1099 72 hpi (m.i) | 2 | 22 | ACT | -0.29967 | -4.5E-05 |
| IAC91-1099 72 hpi (m.i) | 2 | 23 | ACT | -0.30054 | -0.00099 |
| IAC91-1099 72 hpi (m.i) | 2 | 24 | ACT | -0.29728 | 0.002177 |
| IAC91-1099 72 hpi (m.i) | 2 | 25 | ACT | -0.29554 | 0.003839 |
| IAC91-1099 72 hpi (m.i) | 2 | 26 | ACT | -0.28668 | 0.012617 |
| IAC91-1099 72 hpi (m.i) | 2 | 27 | ACT | -0.27119 | 0.028025 |
| IAC91-1099 72 hpi (m.i) | 2 | 28 | ACT | -0.23673 | 0.0624 |
| IAC91-1099 72 hpi (m.i) | 2 | 29 | ACT | -0.17415 | 0.124904 |
| IAC91-1099 72 hpi (m.i) | 2 | 30 | ACT | -0.05231 | 0.246659 |
| IAC91-1099 72 hpi (m.i) | 2 | 31 | ACT | 0.170809 | 0.469699 |
| IAC91-1099 72 hpi (m.i) | 2 | 32 | ACT | 0.569887 | 0.868696 |
| IAC91-1099 72 hpi (m.i) | 2 | 33 | ACT | 1.196339 | 1.495066 |
| IAC91-1099 72 hpi (m.i) | 2 | 34 | ACT | 2.016489 | 2.315134 |
| IAC91-1099 72 hpi (m.i) | 2 | 35 | ACT | 2.825617 | 3.124181 |
| IAC91-1099 72 hpi (m.i) | 2 | 36 | ACT | 3.596373 | 3.894855 |
| IAC91-1099 72 hpi (m.i) | 2 | 37 | ACT | 4.327312 | 4.625711 |
| IAC91-1099 72 hpi (m.i) | 2 | 38 | ACT | 4.920958 | 5.219276 |
| IAC91-1099 72 hpi (m.i) | 2 | 39 | ACT | 5.423435 | 5.721672 |
| IAC91-1099 72 hpi (m.i) | 2 | 40 | ACT | 5.868036 | 6.166192 |
| IAC91-1099 72 hpi (m.i) | 3 | 1 | ACT | -0.34019 | -0.00984 |
| IAC91-1099 72 hpi (m.i) | 3 | 2 | ACT | -0.33623 | -0.00586 |
| IAC91-1099 72 hpi (m.i) | 3 | 3 | ACT | -0.33067 | -0.00028 |
| IAC91-1099 72 hpi (m.i) | 3 | 4 | ACT | -0.33192 | -0.00152 |
| IAC91-1099 72 hpi (m.i) | 3 | 5 | ACT | -0.33076 | -0.00035 |
| IAC91-1099 72 hpi (m.i) | 3 | 6 | ACT | -0.33087 | -0.00043 |
| IAC91-1099 72 hpi (m.i) | 3 | 7 | ACT | -0.32919 | 0.001256 |
| IAC91-1099 72 hpi (m.i) | 3 | 8 | ACT | -0.32881 | 0.001652 |
| IAC91-1099 72 hpi (m.i) | 3 | 9 | ACT | -0.32954 | 0.000942 |
| IAC91-1099 72 hpi (m.i) | 3 | 10 | ACT | -0.33098 | -0.00048 |
| IAC91-1099 72 hpi (m.i) | 3 | 11 | ACT | -0.3272 | 0.003317 |
| IAC91-1099 72 hpi (m.i) | 3 | 12 | ACT | -0.33068 | -0.00015 |
| IAC91-1099 72 hpi (m.i) | 3 | 13 | ACT | -0.3279 | 0.002643 |
| IAC91-1099 72 hpi (m.i) | 3 | 14 | ACT | -0.33103 | -0.00047 |
| IAC91-1099 72 hpi (m.i) | 3 | 15 | ACT | -0.33032 | 0.000255 |
| IAC91-1099 72 hpi (m.i) | 3 | 16 | ACT | -0.33087 | -0.00028 |
| IAC91-1099 72 hpi (m.i) | 3 | 17 | ACT | -0.33359 | -0.00298 |
| IAC91-1099 72 hpi (m.i) | 3 | 18 | ACT | -0.33293 | -0.00231 |
| IAC91-1099 72 hpi (m.i) | 3 | 19 | ACT | -0.33157 | -0.00093 |
| IAC91-1099 72 hpi (m.i) | 3 | 20 | ACT | -0.33364 | -0.00299 |
| IAC91-1099 72 hpi (m.i) | 3 | 21 | ACT | -0.33367 | -0.003 |
| IAC91-1099 72 hpi (m.i) | 3 | 22 | ACT | -0.3318 | -0.00111 |
| IAC91-1099 72 hpi (m.i) | 3 | 23 | ACT | -0.33173 | -0.00103 |
| IAC91-1099 72 hpi (m.i) | 3 | 24 | ACT | -0.32921 | 0.001509 |
| IAC91-1099 72 hpi (m.i) | 3 | 25 | ACT | -0.324 | 0.006731 |
| IAC91-1099 72 hpi (m.i) | 3 | 26 | ACT | -0.31447 | 0.016276 |
| IAC91-1099 72 hpi (m.i) | 3 | 27 | ACT | -0.29804 | 0.032726 |
| IAC91-1099 72 hpi (m.i) | 3 | 28 | ACT | -0.26905 | 0.061725 |
| IAC91-1099 72 hpi (m.i) | 3 | 29 | ACT | -0.21085 | 0.11994 |
| IAC91-1099 72 hpi (m.i) | 3 | 30 | ACT | -0.09203 | 0.238783 |
| IAC91-1099 72 hpi (m.i) | 3 | 31 | ACT | 0.121265 | 0.452091 |
| IAC91-1099 72 hpi (m.i) | 3 | 32 | ACT | 0.509388 | 0.840231 |
| IAC91-1099 72 hpi (m.i) | 3 | 33 | ACT | 1.12911 | 1.459968 |
| IAC91-1099 72 hpi (m.i) | 3 | 34 | ACT | 1.969164 | 2.300039 |
| IAC91-1099 72 hpi (m.i) | 3 | 35 | ACT | 2.828778 | 3.159668 |
| IAC91-1099 72 hpi (m.i) | 3 | 36 | ACT | 3.649412 | 3.980318 |
| IAC91-1099 72 hpi (m.i) | 3 | 37 | ACT | 4.423836 | 4.754757 |
| IAC91-1099 72 hpi (m.i) | 3 | 38 | ACT | 5.056046 | 5.386982 |
| IAC91-1099 72 hpi (m.i) | 3 | 39 | ACT | 5.629018 | 5.959971 |
| IAC91-1099 72 hpi (m.i) | 3 | 40 | ACT | 6.097223 | 6.428192 |
| IAC91-1099 72 hpi (s.i) | 1 | 1 | ACT | -0.36495 | -0.01614 |
| IAC91-1099 72 hpi (s.i) | 1 | 2 | ACT | -0.35796 | -0.0088 |
| IAC91-1099 72 hpi (s.i) | 1 | 3 | ACT | -0.35239 | -0.00288 |
| IAC91-1099 72 hpi (s.i) | 1 | 4 | ACT | -0.35108 | -0.00121 |
| IAC91-1099 72 hpi (s.i) | 1 | 5 | ACT | -0.35067 | -0.00045 |
| IAC91-1099 72 hpi (s.i) | 1 | 6 | ACT | -0.35153 | -0.00095 |
| IAC91-1099 72 hpi (s.i) | 1 | 7 | ACT | -0.35083 | 9.4E-05 |
| IAC91-1099 72 hpi (s.i) | 1 | 8 | ACT | -0.34518 | 0.006098 |
| IAC91-1099 72 hpi (s.i) | 1 | 9 | ACT | -0.35205 | -0.00042 |
| IAC91-1099 72 hpi (s.i) | 1 | 10 | ACT | -0.35291 | -0.00093 |
| IAC91-1099 72 hpi (s.i) | 1 | 11 | ACT | -0.35161 | 0.000727 |
| IAC91-1099 72 hpi (s.i) | 1 | 12 | ACT | -0.35178 | 0.000907 |
| IAC91-1099 72 hpi (s.i) | 1 | 13 | ACT | -0.35095 | 0.002091 |
| IAC91-1099 72 hpi (s.i) | 1 | 14 | ACT | -0.3527 | 0.000686 |
| IAC91-1099 72 hpi (s.i) | 1 | 15 | ACT | -0.35293 | 0.000809 |
| IAC91-1099 72 hpi (s.i) | 1 | 16 | ACT | -0.35355 | 0.000542 |
| IAC91-1099 72 hpi (s.i) | 1 | 17 | ACT | -0.35438 | 6.29E-05 |
| IAC91-1099 72 hpi (s.i) | 1 | 18 | ACT | -0.35557 | -0.00077 |
| IAC91-1099 72 hpi (s.i) | 1 | 19 | ACT | -0.35435 | 0.000805 |
| IAC91-1099 72 hpi (s.i) | 1 | 20 | ACT | -0.35806 | -0.00255 |
| IAC91-1099 72 hpi (s.i) | 1 | 21 | ACT | -0.3555 | 0.000351 |
| IAC91-1099 72 hpi (s.i) | 1 | 22 | ACT | -0.35884 | -0.00263 |
| IAC91-1099 72 hpi (s.i) | 1 | 23 | ACT | -0.35795 | -0.00139 |
| IAC91-1099 72 hpi (s.i) | 1 | 24 | ACT | -0.35961 | -0.0027 |
| IAC91-1099 72 hpi (s.i) | 1 | 25 | ACT | -0.35928 | -0.00202 |
| IAC91-1099 72 hpi (s.i) | 1 | 26 | ACT | -0.358 | -0.00039 |
| IAC91-1099 72 hpi (s.i) | 1 | 27 | ACT | -0.35186 | 0.006107 |
| IAC91-1099 72 hpi (s.i) | 1 | 28 | ACT | -0.34258 | 0.015746 |
| IAC91-1099 72 hpi (s.i) | 1 | 29 | ACT | -0.32491 | 0.033765 |
| IAC91-1099 72 hpi (s.i) | 1 | 30 | ACT | -0.28524 | 0.073787 |
| IAC91-1099 72 hpi (s.i) | 1 | 31 | ACT | -0.21521 | 0.144169 |
| IAC91-1099 72 hpi (s.i) | 1 | 32 | ACT | -0.07834 | 0.281391 |
| IAC91-1099 72 hpi (s.i) | 1 | 33 | ACT | 0.173811 | 0.533893 |
| IAC91-1099 72 hpi (s.i) | 1 | 34 | ACT | 0.609043 | 0.969478 |
| IAC91-1099 72 hpi (s.i) | 1 | 35 | ACT | 1.257151 | 1.617937 |
| IAC91-1099 72 hpi (s.i) | 1 | 36 | ACT | 2.043489 | 2.404628 |
| IAC91-1099 72 hpi (s.i) | 1 | 37 | ACT | 2.846386 | 3.207877 |
| IAC91-1099 72 hpi (s.i) | 1 | 38 | ACT | 3.560384 | 3.922227 |
| IAC91-1099 72 hpi (s.i) | 1 | 39 | ACT | 4.252596 | 4.614792 |
| IAC91-1099 72 hpi (s.i) | 1 | 40 | ACT | 4.8748 | 5.237348 |
| IAC91-1099 72 hpi (s.i) | 2 | 1 | ACT | -0.36799 | -0.01403 |
| IAC91-1099 72 hpi (s.i) | 2 | 2 | ACT | -0.36088 | -0.00687 |
| IAC91-1099 72 hpi (s.i) | 2 | 3 | ACT | -0.3623 | -0.00825 |
| IAC91-1099 72 hpi (s.i) | 2 | 4 | ACT | -0.35469 | -0.00059 |
| IAC91-1099 72 hpi (s.i) | 2 | 5 | ACT | -0.3528 | 0.001342 |
| IAC91-1099 72 hpi (s.i) | 2 | 6 | ACT | -0.34866 | 0.005525 |
| IAC91-1099 72 hpi (s.i) | 2 | 7 | ACT | -0.34726 | 0.006968 |
| IAC91-1099 72 hpi (s.i) | 2 | 8 | ACT | -0.36143 | -0.00717 |
| IAC91-1099 72 hpi (s.i) | 2 | 9 | ACT | -0.34699 | 0.007319 |
| IAC91-1099 72 hpi (s.i) | 2 | 10 | ACT | -0.34987 | 0.004482 |
| IAC91-1099 72 hpi (s.i) | 2 | 11 | ACT | -0.35248 | 0.001917 |
| IAC91-1099 72 hpi (s.i) | 2 | 12 | ACT | -0.35087 | 0.003577 |
| IAC91-1099 72 hpi (s.i) | 2 | 13 | ACT | -0.35572 | -0.00123 |
| IAC91-1099 72 hpi (s.i) | 2 | 14 | ACT | -0.35376 | 0.000771 |
| IAC91-1099 72 hpi (s.i) | 2 | 15 | ACT | -0.35971 | -0.00513 |
| IAC91-1099 72 hpi (s.i) | 2 | 16 | ACT | -0.35838 | -0.00376 |
| IAC91-1099 72 hpi (s.i) | 2 | 17 | ACT | -0.35738 | -0.00272 |
| IAC91-1099 72 hpi (s.i) | 2 | 18 | ACT | -0.35452 | 0.000179 |
| IAC91-1099 72 hpi (s.i) | 2 | 19 | ACT | -0.36212 | -0.00738 |
| IAC91-1099 72 hpi (s.i) | 2 | 20 | ACT | -0.35417 | 0.000616 |
| IAC91-1099 72 hpi (s.i) | 2 | 21 | ACT | -0.36078 | -0.00595 |
| IAC91-1099 72 hpi (s.i) | 2 | 22 | ACT | -0.35405 | 0.000832 |
| IAC91-1099 72 hpi (s.i) | 2 | 23 | ACT | -0.35971 | -0.00479 |
| IAC91-1099 72 hpi (s.i) | 2 | 24 | ACT | -0.35359 | 0.001372 |
| IAC91-1099 72 hpi (s.i) | 2 | 25 | ACT | -0.35347 | 0.001541 |
| IAC91-1099 72 hpi (s.i) | 2 | 26 | ACT | -0.35051 | 0.004541 |
| IAC91-1099 72 hpi (s.i) | 2 | 27 | ACT | -0.3491 | 0.005994 |
| IAC91-1099 72 hpi (s.i) | 2 | 28 | ACT | -0.34149 | 0.01365 |
| IAC91-1099 72 hpi (s.i) | 2 | 29 | ACT | -0.32861 | 0.026574 |
| IAC91-1099 72 hpi (s.i) | 2 | 30 | ACT | -0.30028 | 0.054943 |
| IAC91-1099 72 hpi (s.i) | 2 | 31 | ACT | -0.25091 | 0.104356 |
| IAC91-1099 72 hpi (s.i) | 2 | 32 | ACT | -0.15537 | 0.199943 |
| IAC91-1099 72 hpi (s.i) | 2 | 33 | ACT | 0.027228 | 0.382583 |
| IAC91-1099 72 hpi (s.i) | 2 | 34 | ACT | 0.359061 | 0.714459 |
| IAC91-1099 72 hpi (s.i) | 2 | 35 | ACT | 0.899857 | 1.2553 |
| IAC91-1099 72 hpi (s.i) | 2 | 36 | ACT | 1.677027 | 2.032512 |
| IAC91-1099 72 hpi (s.i) | 2 | 37 | ACT | 2.551157 | 2.906686 |
| IAC91-1099 72 hpi (s.i) | 2 | 38 | ACT | 3.338648 | 3.69422 |
| IAC91-1099 72 hpi (s.i) | 2 | 39 | ACT | 4.092749 | 4.448365 |
| IAC91-1099 72 hpi (s.i) | 2 | 40 | ACT | 4.784309 | 5.139969 |
| IAC91-1099 72 hpi (s.i) | 3 | 1 | ACT | -0.2842 | -0.00944 |
| IAC91-1099 72 hpi (s.i) | 3 | 2 | ACT | -0.28039 | -0.00488 |
| IAC91-1099 72 hpi (s.i) | 3 | 3 | ACT | -0.27337 | 0.002877 |
| IAC91-1099 72 hpi (s.i) | 3 | 4 | ACT | -0.27628 | 0.000715 |
| IAC91-1099 72 hpi (s.i) | 3 | 5 | ACT | -0.27827 | -0.00053 |
| IAC91-1099 72 hpi (s.i) | 3 | 6 | ACT | -0.28053 | -0.00205 |
| IAC91-1099 72 hpi (s.i) | 3 | 7 | ACT | -0.28076 | -0.00153 |
| IAC91-1099 72 hpi (s.i) | 3 | 8 | ACT | -0.26715 | 0.012827 |
| IAC91-1099 72 hpi (s.i) | 3 | 9 | ACT | -0.28324 | -0.00252 |
| IAC91-1099 72 hpi (s.i) | 3 | 10 | ACT | -0.28357 | -0.00211 |
| IAC91-1099 72 hpi (s.i) | 3 | 11 | ACT | -0.28657 | -0.00436 |
| IAC91-1099 72 hpi (s.i) | 3 | 12 | ACT | -0.28419 | -0.00124 |
| IAC91-1099 72 hpi (s.i) | 3 | 13 | ACT | -0.28329 | 0.000406 |
| IAC91-1099 72 hpi (s.i) | 3 | 14 | ACT | -0.28772 | -0.00328 |
| IAC91-1099 72 hpi (s.i) | 3 | 15 | ACT | -0.28441 | 0.000776 |
| IAC91-1099 72 hpi (s.i) | 3 | 16 | ACT | -0.28629 | -0.00035 |
| IAC91-1099 72 hpi (s.i) | 3 | 17 | ACT | -0.28793 | -0.00125 |
| IAC91-1099 72 hpi (s.i) | 3 | 18 | ACT | -0.29038 | -0.00296 |
| IAC91-1099 72 hpi (s.i) | 3 | 19 | ACT | -0.28841 | -0.00024 |
| IAC91-1099 72 hpi (s.i) | 3 | 20 | ACT | -0.29333 | -0.00441 |
| IAC91-1099 72 hpi (s.i) | 3 | 21 | ACT | -0.28755 | 0.002108 |
| IAC91-1099 72 hpi (s.i) | 3 | 22 | ACT | -0.29153 | -0.00112 |
| IAC91-1099 72 hpi (s.i) | 3 | 23 | ACT | -0.28788 | 0.003274 |
| IAC91-1099 72 hpi (s.i) | 3 | 24 | ACT | -0.29075 | 0.001141 |
| IAC91-1099 72 hpi (s.i) | 3 | 25 | ACT | -0.29101 | 0.001629 |
| IAC91-1099 72 hpi (s.i) | 3 | 26 | ACT | -0.29119 | 0.00219 |
| IAC91-1099 72 hpi (s.i) | 3 | 27 | ACT | -0.28221 | 0.011918 |
| IAC91-1099 72 hpi (s.i) | 3 | 28 | ACT | -0.27166 | 0.023212 |
| IAC91-1099 72 hpi (s.i) | 3 | 29 | ACT | -0.24404 | 0.051582 |
| IAC91-1099 72 hpi (s.i) | 3 | 30 | ACT | -0.19477 | 0.101599 |
| IAC91-1099 72 hpi (s.i) | 3 | 31 | ACT | -0.1013 | 0.195808 |
| IAC91-1099 72 hpi (s.i) | 3 | 32 | ACT | 0.077623 | 0.375479 |
| IAC91-1099 72 hpi (s.i) | 3 | 33 | ACT | 0.401831 | 0.700432 |
| IAC91-1099 72 hpi (s.i) | 3 | 34 | ACT | 0.941503 | 1.240848 |
| IAC91-1099 72 hpi (s.i) | 3 | 35 | ACT | 1.714153 | 2.014244 |
| IAC91-1099 72 hpi (s.i) | 3 | 36 | ACT | 2.582967 | 2.883803 |
| IAC91-1099 72 hpi (s.i) | 3 | 37 | ACT | 3.412744 | 3.714325 |
| IAC91-1099 72 hpi (s.i) | 3 | 38 | ACT | 4.120954 | 4.42328 |
| IAC91-1099 72 hpi (s.i) | 3 | 39 | ACT | 4.779355 | 5.082426 |
| IAC91-1099 72 hpi (s.i) | 3 | 40 | ACT | 5.345292 | 5.649108 |
| IACSP95-5000 24 hpi (m.i) | 1 | 1 | ACT | -0.32066 | -0.00256 |
| IACSP95-5000 24 hpi (m.i) | 1 | 2 | ACT | -0.32084 | -0.00275 |
| IACSP95-5000 24 hpi (m.i) | 1 | 3 | ACT | -0.31966 | -0.00158 |
| IACSP95-5000 24 hpi (m.i) | 1 | 4 | ACT | -0.32052 | -0.00245 |
| IACSP95-5000 24 hpi (m.i) | 1 | 5 | ACT | -0.31407 | 0.003995 |
| IACSP95-5000 24 hpi (m.i) | 1 | 6 | ACT | -0.31548 | 0.002581 |
| IACSP95-5000 24 hpi (m.i) | 1 | 7 | ACT | -0.31501 | 0.003037 |
| IACSP95-5000 24 hpi (m.i) | 1 | 8 | ACT | -0.32024 | -0.0022 |
| IACSP95-5000 24 hpi (m.i) | 1 | 9 | ACT | -0.31773 | 0.000305 |
| IACSP95-5000 24 hpi (m.i) | 1 | 10 | ACT | -0.31615 | 0.001878 |
| IACSP95-5000 24 hpi (m.i) | 1 | 11 | ACT | -0.31556 | 0.002463 |
| IACSP95-5000 24 hpi (m.i) | 1 | 12 | ACT | -0.31852 | -0.00051 |
| IACSP95-5000 24 hpi (m.i) | 1 | 13 | ACT | -0.32127 | -0.00327 |
| IACSP95-5000 24 hpi (m.i) | 1 | 14 | ACT | -0.32048 | -0.00248 |
| IACSP95-5000 24 hpi (m.i) | 1 | 15 | ACT | -0.3177 | 0.000296 |
| IACSP95-5000 24 hpi (m.i) | 1 | 16 | ACT | -0.31827 | -0.00028 |
| IACSP95-5000 24 hpi (m.i) | 1 | 17 | ACT | -0.32067 | -0.00269 |
| IACSP95-5000 24 hpi (m.i) | 1 | 18 | ACT | -0.31769 | 0.00028 |
| IACSP95-5000 24 hpi (m.i) | 1 | 19 | ACT | -0.32292 | -0.00496 |
| IACSP95-5000 24 hpi (m.i) | 1 | 20 | ACT | -0.31825 | -0.00029 |
| IACSP95-5000 24 hpi (m.i) | 1 | 21 | ACT | -0.32035 | -0.0024 |
| IACSP95-5000 24 hpi (m.i) | 1 | 22 | ACT | -0.31658 | 0.001361 |
| IACSP95-5000 24 hpi (m.i) | 1 | 23 | ACT | -0.31774 | 0.000194 |
| IACSP95-5000 24 hpi (m.i) | 1 | 24 | ACT | -0.31696 | 0.000964 |
| IACSP95-5000 24 hpi (m.i) | 1 | 25 | ACT | -0.31216 | 0.005756 |
| IACSP95-5000 24 hpi (m.i) | 1 | 26 | ACT | -0.30443 | 0.013487 |
| IACSP95-5000 24 hpi (m.i) | 1 | 27 | ACT | -0.28772 | 0.030184 |
| IACSP95-5000 24 hpi (m.i) | 1 | 28 | ACT | -0.25721 | 0.060693 |
| IACSP95-5000 24 hpi (m.i) | 1 | 29 | ACT | -0.19646 | 0.121428 |
| IACSP95-5000 24 hpi (m.i) | 1 | 30 | ACT | -0.07878 | 0.2391 |
| IACSP95-5000 24 hpi (m.i) | 1 | 31 | ACT | 0.14024 | 0.458117 |
| IACSP95-5000 24 hpi (m.i) | 1 | 32 | ACT | 0.528476 | 0.846346 |
| IACSP95-5000 24 hpi (m.i) | 1 | 33 | ACT | 1.144747 | 1.46261 |
| IACSP95-5000 24 hpi (m.i) | 1 | 34 | ACT | 1.965657 | 2.283512 |
| IACSP95-5000 24 hpi (m.i) | 1 | 35 | ACT | 2.805874 | 3.123722 |
| IACSP95-5000 24 hpi (m.i) | 1 | 36 | ACT | 3.571191 | 3.889032 |
| IACSP95-5000 24 hpi (m.i) | 1 | 37 | ACT | 4.285092 | 4.602926 |
| IACSP95-5000 24 hpi (m.i) | 1 | 38 | ACT | 4.88071 | 5.198537 |
| IACSP95-5000 24 hpi (m.i) | 1 | 39 | ACT | 5.359756 | 5.677576 |
| IACSP95-5000 24 hpi (m.i) | 1 | 40 | ACT | 5.774852 | 6.092664 |
| IACSP95-5000 24 hpi (m.i) | 2 | 1 | ACT | -0.25715 | -0.00308 |
| IACSP95-5000 24 hpi (m.i) | 2 | 2 | ACT | -0.25725 | -0.00274 |
| IACSP95-5000 24 hpi (m.i) | 2 | 3 | ACT | -0.25483 | 0.00012 |
| IACSP95-5000 24 hpi (m.i) | 2 | 4 | ACT | -0.25454 | 0.000842 |
| IACSP95-5000 24 hpi (m.i) | 2 | 5 | ACT | -0.25348 | 0.00233 |
| IACSP95-5000 24 hpi (m.i) | 2 | 6 | ACT | -0.2559 | 0.00035 |
| IACSP95-5000 24 hpi (m.i) | 2 | 7 | ACT | -0.25513 | 0.001553 |
| IACSP95-5000 24 hpi (m.i) | 2 | 8 | ACT | -0.25747 | -0.00035 |
| IACSP95-5000 24 hpi (m.i) | 2 | 9 | ACT | -0.25623 | 0.001316 |
| IACSP95-5000 24 hpi (m.i) | 2 | 10 | ACT | -0.25778 | 0.000201 |
| IACSP95-5000 24 hpi (m.i) | 2 | 11 | ACT | -0.25921 | -0.00079 |
| IACSP95-5000 24 hpi (m.i) | 2 | 12 | ACT | -0.26067 | -0.00182 |
| IACSP95-5000 24 hpi (m.i) | 2 | 13 | ACT | -0.26 | -0.00072 |
| IACSP95-5000 24 hpi (m.i) | 2 | 14 | ACT | -0.26194 | -0.00222 |
| IACSP95-5000 24 hpi (m.i) | 2 | 15 | ACT | -0.26074 | -0.00058 |
| IACSP95-5000 24 hpi (m.i) | 2 | 16 | ACT | -0.26422 | -0.00363 |
| IACSP95-5000 24 hpi (m.i) | 2 | 17 | ACT | -0.26399 | -0.00297 |
| IACSP95-5000 24 hpi (m.i) | 2 | 18 | ACT | -0.26131 | 0.000146 |
| IACSP95-5000 24 hpi (m.i) | 2 | 19 | ACT | -0.26211 | -0.00022 |
| IACSP95-5000 24 hpi (m.i) | 2 | 20 | ACT | -0.26216 | 0.000165 |
| IACSP95-5000 24 hpi (m.i) | 2 | 21 | ACT | -0.26262 | 0.000136 |
| IACSP95-5000 24 hpi (m.i) | 2 | 22 | ACT | -0.26201 | 0.001183 |
| IACSP95-5000 24 hpi (m.i) | 2 | 23 | ACT | -0.26293 | 0.000695 |
| IACSP95-5000 24 hpi (m.i) | 2 | 24 | ACT | -0.2598 | 0.004257 |
| IACSP95-5000 24 hpi (m.i) | 2 | 25 | ACT | -0.25743 | 0.007069 |
| IACSP95-5000 24 hpi (m.i) | 2 | 26 | ACT | -0.24541 | 0.019514 |
| IACSP95-5000 24 hpi (m.i) | 2 | 27 | ACT | -0.22984 | 0.035525 |
| IACSP95-5000 24 hpi (m.i) | 2 | 28 | ACT | -0.19529 | 0.070503 |
| IACSP95-5000 24 hpi (m.i) | 2 | 29 | ACT | -0.12354 | 0.142686 |
| IACSP95-5000 24 hpi (m.i) | 2 | 30 | ACT | 0.010163 | 0.276828 |
| IACSP95-5000 24 hpi (m.i) | 2 | 31 | ACT | 0.25381 | 0.520909 |
| IACSP95-5000 24 hpi (m.i) | 2 | 32 | ACT | 0.679941 | 0.947475 |
| IACSP95-5000 24 hpi (m.i) | 2 | 33 | ACT | 1.332215 | 1.600182 |
| IACSP95-5000 24 hpi (m.i) | 2 | 34 | ACT | 2.157193 | 2.425594 |
| IACSP95-5000 24 hpi (m.i) | 2 | 35 | ACT | 2.972873 | 3.241708 |
| IACSP95-5000 24 hpi (m.i) | 2 | 36 | ACT | 3.718561 | 3.987831 |
| IACSP95-5000 24 hpi (m.i) | 2 | 37 | ACT | 4.406278 | 4.675982 |
| IACSP95-5000 24 hpi (m.i) | 2 | 38 | ACT | 4.976149 | 5.246286 |
| IACSP95-5000 24 hpi (m.i) | 2 | 39 | ACT | 5.435522 | 5.706093 |
| IACSP95-5000 24 hpi (m.i) | 2 | 40 | ACT | 5.844144 | 6.115149 |
| IACSP95-5000 24 hpi (m.i) | 3 | 1 | ACT | -0.3217 | -0.00898 |
| IACSP95-5000 24 hpi (m.i) | 3 | 2 | ACT | -0.3151 | -0.00232 |
| IACSP95-5000 24 hpi (m.i) | 3 | 3 | ACT | -0.31474 | -0.0019 |
| IACSP95-5000 24 hpi (m.i) | 3 | 4 | ACT | -0.31666 | -0.00377 |
| IACSP95-5000 24 hpi (m.i) | 3 | 5 | ACT | -0.31325 | -0.00031 |
| IACSP95-5000 24 hpi (m.i) | 3 | 6 | ACT | -0.31216 | 0.000843 |
| IACSP95-5000 24 hpi (m.i) | 3 | 7 | ACT | -0.31041 | 0.00265 |
| IACSP95-5000 24 hpi (m.i) | 3 | 8 | ACT | -0.31418 | -0.00106 |
| IACSP95-5000 24 hpi (m.i) | 3 | 9 | ACT | -0.31234 | 0.000828 |
| IACSP95-5000 24 hpi (m.i) | 3 | 10 | ACT | -0.30846 | 0.004772 |
| IACSP95-5000 24 hpi (m.i) | 3 | 11 | ACT | -0.31281 | 0.000473 |
| IACSP95-5000 24 hpi (m.i) | 3 | 12 | ACT | -0.30939 | 0.003953 |
| IACSP95-5000 24 hpi (m.i) | 3 | 13 | ACT | -0.31497 | -0.00157 |
| IACSP95-5000 24 hpi (m.i) | 3 | 14 | ACT | -0.31329 | 0.000166 |
| IACSP95-5000 24 hpi (m.i) | 3 | 15 | ACT | -0.31397 | -0.00045 |
| IACSP95-5000 24 hpi (m.i) | 3 | 16 | ACT | -0.30951 | 0.004062 |
| IACSP95-5000 24 hpi (m.i) | 3 | 17 | ACT | -0.31535 | -0.00173 |
| IACSP95-5000 24 hpi (m.i) | 3 | 18 | ACT | -0.31637 | -0.00268 |
| IACSP95-5000 24 hpi (m.i) | 3 | 19 | ACT | -0.31723 | -0.00349 |
| IACSP95-5000 24 hpi (m.i) | 3 | 20 | ACT | -0.31481 | -0.00102 |
| IACSP95-5000 24 hpi (m.i) | 3 | 21 | ACT | -0.31574 | -0.00189 |
| IACSP95-5000 24 hpi (m.i) | 3 | 22 | ACT | -0.31685 | -0.00294 |
| IACSP95-5000 24 hpi (m.i) | 3 | 23 | ACT | -0.31416 | -0.00019 |
| IACSP95-5000 24 hpi (m.i) | 3 | 24 | ACT | -0.31511 | -0.00109 |
| IACSP95-5000 24 hpi (m.i) | 3 | 25 | ACT | -0.30775 | 0.006331 |
| IACSP95-5000 24 hpi (m.i) | 3 | 26 | ACT | -0.30288 | 0.01126 |
| IACSP95-5000 24 hpi (m.i) | 3 | 27 | ACT | -0.28491 | 0.029284 |
| IACSP95-5000 24 hpi (m.i) | 3 | 28 | ACT | -0.25735 | 0.056898 |
| IACSP95-5000 24 hpi (m.i) | 3 | 29 | ACT | -0.20289 | 0.111415 |
| IACSP95-5000 24 hpi (m.i) | 3 | 30 | ACT | -0.09738 | 0.216985 |
| IACSP95-5000 24 hpi (m.i) | 3 | 31 | ACT | 0.099514 | 0.413933 |
| IACSP95-5000 24 hpi (m.i) | 3 | 32 | ACT | 0.458924 | 0.773401 |
| IACSP95-5000 24 hpi (m.i) | 3 | 33 | ACT | 1.050537 | 1.36507 |
| IACSP95-5000 24 hpi (m.i) | 3 | 34 | ACT | 1.890913 | 2.205502 |
| IACSP95-5000 24 hpi (m.i) | 3 | 35 | ACT | 2.80757 | 3.122216 |
| IACSP95-5000 24 hpi (m.i) | 3 | 36 | ACT | 3.652572 | 3.967275 |
| IACSP95-5000 24 hpi (m.i) | 3 | 37 | ACT | 4.440834 | 4.755593 |
| IACSP95-5000 24 hpi (m.i) | 3 | 38 | ACT | 5.050702 | 5.365518 |
| IACSP95-5000 24 hpi (m.i) | 3 | 39 | ACT | 5.611827 | 5.9267 |
| IACSP95-5000 24 hpi (m.i) | 3 | 40 | ACT | 6.101176 | 6.416106 |
| IACSP95-5000 24 hpi (s.i) | 1 | 1 | ACT | -0.29333 | -0.00792 |
| IACSP95-5000 24 hpi (s.i) | 1 | 2 | ACT | -0.29094 | -0.00521 |
| IACSP95-5000 24 hpi (s.i) | 1 | 3 | ACT | -0.28851 | -0.00246 |
| IACSP95-5000 24 hpi (s.i) | 1 | 4 | ACT | -0.2882 | -0.00183 |
| IACSP95-5000 24 hpi (s.i) | 1 | 5 | ACT | -0.28418 | 0.002522 |
| IACSP95-5000 24 hpi (s.i) | 1 | 6 | ACT | -0.28562 | 0.001397 |
| IACSP95-5000 24 hpi (s.i) | 1 | 7 | ACT | -0.28476 | 0.002585 |
| IACSP95-5000 24 hpi (s.i) | 1 | 8 | ACT | -0.28765 | 1.71E-05 |
| IACSP95-5000 24 hpi (s.i) | 1 | 9 | ACT | -0.28821 | -0.00022 |
| IACSP95-5000 24 hpi (s.i) | 1 | 10 | ACT | -0.28817 | 0.000147 |
| IACSP95-5000 24 hpi (s.i) | 1 | 11 | ACT | -0.28804 | 0.000596 |
| IACSP95-5000 24 hpi (s.i) | 1 | 12 | ACT | -0.288 | 0.000957 |
| IACSP95-5000 24 hpi (s.i) | 1 | 13 | ACT | -0.28961 | -0.00033 |
| IACSP95-5000 24 hpi (s.i) | 1 | 14 | ACT | -0.29078 | -0.00118 |
| IACSP95-5000 24 hpi (s.i) | 1 | 15 | ACT | -0.28948 | 0.000446 |
| IACSP95-5000 24 hpi (s.i) | 1 | 16 | ACT | -0.29094 | -0.00069 |
| IACSP95-5000 24 hpi (s.i) | 1 | 17 | ACT | -0.29181 | -0.00124 |
| IACSP95-5000 24 hpi (s.i) | 1 | 18 | ACT | -0.2904 | 0.000494 |
| IACSP95-5000 24 hpi (s.i) | 1 | 19 | ACT | -0.29341 | -0.00219 |
| IACSP95-5000 24 hpi (s.i) | 1 | 20 | ACT | -0.29299 | -0.00145 |
| IACSP95-5000 24 hpi (s.i) | 1 | 21 | ACT | -0.29263 | -0.00077 |
| IACSP95-5000 24 hpi (s.i) | 1 | 22 | ACT | -0.29227 | -8.1E-05 |
| IACSP95-5000 24 hpi (s.i) | 1 | 23 | ACT | -0.29387 | -0.00136 |
| IACSP95-5000 24 hpi (s.i) | 1 | 24 | ACT | -0.29204 | 0.000789 |
| IACSP95-5000 24 hpi (s.i) | 1 | 25 | ACT | -0.2893 | 0.00385 |
| IACSP95-5000 24 hpi (s.i) | 1 | 26 | ACT | -0.28555 | 0.007924 |
| IACSP95-5000 24 hpi (s.i) | 1 | 27 | ACT | -0.27485 | 0.018949 |
| IACSP95-5000 24 hpi (s.i) | 1 | 28 | ACT | -0.25912 | 0.035002 |
| IACSP95-5000 24 hpi (s.i) | 1 | 29 | ACT | -0.22304 | 0.071405 |
| IACSP95-5000 24 hpi (s.i) | 1 | 30 | ACT | -0.15584 | 0.138927 |
| IACSP95-5000 24 hpi (s.i) | 1 | 31 | ACT | -0.02566 | 0.269432 |
| IACSP95-5000 24 hpi (s.i) | 1 | 32 | ACT | 0.21629 | 0.511705 |
| IACSP95-5000 24 hpi (s.i) | 1 | 33 | ACT | 0.642765 | 0.938503 |
| IACSP95-5000 24 hpi (s.i) | 1 | 34 | ACT | 1.31828 | 1.614341 |
| IACSP95-5000 24 hpi (s.i) | 1 | 35 | ACT | 2.168687 | 2.46507 |
| IACSP95-5000 24 hpi (s.i) | 1 | 36 | ACT | 2.990372 | 3.287079 |
| IACSP95-5000 24 hpi (s.i) | 1 | 37 | ACT | 3.747004 | 4.044033 |
| IACSP95-5000 24 hpi (s.i) | 1 | 38 | ACT | 4.375139 | 4.672491 |
| IACSP95-5000 24 hpi (s.i) | 1 | 39 | ACT | 4.903433 | 5.201108 |
| IACSP95-5000 24 hpi (s.i) | 1 | 40 | ACT | 5.356763 | 5.654761 |
| IACSP95-5000 24 hpi (s.i) | 2 | 1 | ACT | -0.27596 | -0.00117 |
| IACSP95-5000 24 hpi (s.i) | 2 | 2 | ACT | -0.2759 | -0.00071 |
| IACSP95-5000 24 hpi (s.i) | 2 | 3 | ACT | -0.27418 | 0.001423 |
| IACSP95-5000 24 hpi (s.i) | 2 | 4 | ACT | -0.27477 | 0.00124 |
| IACSP95-5000 24 hpi (s.i) | 2 | 5 | ACT | -0.27422 | 0.002205 |
| IACSP95-5000 24 hpi (s.i) | 2 | 6 | ACT | -0.27455 | 0.002276 |
| IACSP95-5000 24 hpi (s.i) | 2 | 7 | ACT | -0.27497 | 0.002262 |
| IACSP95-5000 24 hpi (s.i) | 2 | 8 | ACT | -0.27796 | -0.00031 |
| IACSP95-5000 24 hpi (s.i) | 2 | 9 | ACT | -0.27876 | -0.00071 |
| IACSP95-5000 24 hpi (s.i) | 2 | 10 | ACT | -0.27814 | 0.000314 |
| IACSP95-5000 24 hpi (s.i) | 2 | 11 | ACT | -0.27979 | -0.00092 |
| IACSP95-5000 24 hpi (s.i) | 2 | 12 | ACT | -0.28126 | -0.00198 |
| IACSP95-5000 24 hpi (s.i) | 2 | 13 | ACT | -0.28295 | -0.00327 |
| IACSP95-5000 24 hpi (s.i) | 2 | 14 | ACT | -0.2842 | -0.00412 |
| IACSP95-5000 24 hpi (s.i) | 2 | 15 | ACT | -0.28259 | -0.00209 |
| IACSP95-5000 24 hpi (s.i) | 2 | 16 | ACT | -0.28216 | -0.00126 |
| IACSP95-5000 24 hpi (s.i) | 2 | 17 | ACT | -0.28351 | -0.0022 |
| IACSP95-5000 24 hpi (s.i) | 2 | 18 | ACT | -0.28199 | -0.00027 |
| IACSP95-5000 24 hpi (s.i) | 2 | 19 | ACT | -0.28362 | -0.00149 |
| IACSP95-5000 24 hpi (s.i) | 2 | 20 | ACT | -0.28201 | 0.000523 |
| IACSP95-5000 24 hpi (s.i) | 2 | 21 | ACT | -0.28356 | -0.00062 |
| IACSP95-5000 24 hpi (s.i) | 2 | 22 | ACT | -0.28153 | 0.001814 |
| IACSP95-5000 24 hpi (s.i) | 2 | 23 | ACT | -0.28205 | 0.001704 |
| IACSP95-5000 24 hpi (s.i) | 2 | 24 | ACT | -0.28275 | 0.001417 |
| IACSP95-5000 24 hpi (s.i) | 2 | 25 | ACT | -0.2805 | 0.004067 |
| IACSP95-5000 24 hpi (s.i) | 2 | 26 | ACT | -0.27479 | 0.010187 |
| IACSP95-5000 24 hpi (s.i) | 2 | 27 | ACT | -0.26212 | 0.023264 |
| IACSP95-5000 24 hpi (s.i) | 2 | 28 | ACT | -0.2433 | 0.042496 |
| IACSP95-5000 24 hpi (s.i) | 2 | 29 | ACT | -0.20223 | 0.083973 |
| IACSP95-5000 24 hpi (s.i) | 2 | 30 | ACT | -0.1222 | 0.16441 |
| IACSP95-5000 24 hpi (s.i) | 2 | 31 | ACT | 0.027973 | 0.314988 |
| IACSP95-5000 24 hpi (s.i) | 2 | 32 | ACT | 0.304402 | 0.591824 |
| IACSP95-5000 24 hpi (s.i) | 2 | 33 | ACT | 0.781234 | 1.069064 |
| IACSP95-5000 24 hpi (s.i) | 2 | 34 | ACT | 1.517966 | 1.806203 |
| IACSP95-5000 24 hpi (s.i) | 2 | 35 | ACT | 2.414482 | 2.703126 |
| IACSP95-5000 24 hpi (s.i) | 2 | 36 | ACT | 3.283363 | 3.572415 |
| IACSP95-5000 24 hpi (s.i) | 2 | 37 | ACT | 4.082886 | 4.372345 |
| IACSP95-5000 24 hpi (s.i) | 2 | 38 | ACT | 4.75165 | 5.041517 |
| IACSP95-5000 24 hpi (s.i) | 2 | 39 | ACT | 5.326415 | 5.61669 |
| IACSP95-5000 24 hpi (s.i) | 2 | 40 | ACT | 5.813374 | 6.104056 |
| IACSP95-5000 24 hpi (s.i) | 3 | 1 | ACT | -0.24839 | -0.00153 |
| IACSP95-5000 24 hpi (s.i) | 3 | 2 | ACT | -0.24639 | 0.000351 |
| IACSP95-5000 24 hpi (s.i) | 3 | 3 | ACT | -0.24565 | 0.000979 |
| IACSP95-5000 24 hpi (s.i) | 3 | 4 | ACT | -0.24621 | 0.000296 |
| IACSP95-5000 24 hpi (s.i) | 3 | 5 | ACT | -0.24408 | 0.002316 |
| IACSP95-5000 24 hpi (s.i) | 3 | 6 | ACT | -0.24502 | 0.001253 |
| IACSP95-5000 24 hpi (s.i) | 3 | 7 | ACT | -0.24422 | 0.001938 |
| IACSP95-5000 24 hpi (s.i) | 3 | 8 | ACT | -0.24643 | -0.00039 |
| IACSP95-5000 24 hpi (s.i) | 3 | 9 | ACT | -0.24531 | 0.000612 |
| IACSP95-5000 24 hpi (s.i) | 3 | 10 | ACT | -0.24679 | -0.00098 |
| IACSP95-5000 24 hpi (s.i) | 3 | 11 | ACT | -0.24716 | -0.00147 |
| IACSP95-5000 24 hpi (s.i) | 3 | 12 | ACT | -0.24648 | -0.0009 |
| IACSP95-5000 24 hpi (s.i) | 3 | 13 | ACT | -0.24618 | -0.00072 |
| IACSP95-5000 24 hpi (s.i) | 3 | 14 | ACT | -0.24672 | -0.00137 |
| IACSP95-5000 24 hpi (s.i) | 3 | 15 | ACT | -0.24596 | -0.00073 |
| IACSP95-5000 24 hpi (s.i) | 3 | 16 | ACT | -0.24638 | -0.00127 |
| IACSP95-5000 24 hpi (s.i) | 3 | 17 | ACT | -0.24665 | -0.00166 |
| IACSP95-5000 24 hpi (s.i) | 3 | 18 | ACT | -0.24438 | 0.000493 |
| IACSP95-5000 24 hpi (s.i) | 3 | 19 | ACT | -0.24646 | -0.0017 |
| IACSP95-5000 24 hpi (s.i) | 3 | 20 | ACT | -0.24621 | -0.00157 |
| IACSP95-5000 24 hpi (s.i) | 3 | 21 | ACT | -0.24626 | -0.00173 |
| IACSP95-5000 24 hpi (s.i) | 3 | 22 | ACT | -0.24505 | -0.00064 |
| IACSP95-5000 24 hpi (s.i) | 3 | 23 | ACT | -0.24565 | -0.00135 |
| IACSP95-5000 24 hpi (s.i) | 3 | 24 | ACT | -0.24471 | -0.00053 |
| IACSP95-5000 24 hpi (s.i) | 3 | 25 | ACT | -0.24169 | 0.002376 |
| IACSP95-5000 24 hpi (s.i) | 3 | 26 | ACT | -0.23721 | 0.006738 |
| IACSP95-5000 24 hpi (s.i) | 3 | 27 | ACT | -0.22771 | 0.016118 |
| IACSP95-5000 24 hpi (s.i) | 3 | 28 | ACT | -0.21083 | 0.032887 |
| IACSP95-5000 24 hpi (s.i) | 3 | 29 | ACT | -0.17316 | 0.070432 |
| IACSP95-5000 24 hpi (s.i) | 3 | 30 | ACT | -0.10626 | 0.137216 |
| IACSP95-5000 24 hpi (s.i) | 3 | 31 | ACT | 0.026451 | 0.269814 |
| IACSP95-5000 24 hpi (s.i) | 3 | 32 | ACT | 0.268354 | 0.5116 |
| IACSP95-5000 24 hpi (s.i) | 3 | 33 | ACT | 0.694127 | 0.937257 |
| IACSP95-5000 24 hpi (s.i) | 3 | 34 | ACT | 1.378581 | 1.621595 |
| IACSP95-5000 24 hpi (s.i) | 3 | 35 | ACT | 2.287117 | 2.530015 |
| IACSP95-5000 24 hpi (s.i) | 3 | 36 | ACT | 3.219457 | 3.462237 |
| IACSP95-5000 24 hpi (s.i) | 3 | 37 | ACT | 4.077604 | 4.320268 |
| IACSP95-5000 24 hpi (s.i) | 3 | 38 | ACT | 4.800513 | 5.043061 |
| IACSP95-5000 24 hpi (s.i) | 3 | 39 | ACT | 5.424869 | 5.6673 |
| IACSP95-5000 24 hpi (s.i) | 3 | 40 | ACT | 5.969747 | 6.212062 |
| IACSP95-5000 72 hpi (m.i) | 1 | 1 | ACT | -0.30251 | -0.00874 |
| IACSP95-5000 72 hpi (m.i) | 1 | 2 | ACT | -0.29922 | -0.00528 |
| IACSP95-5000 72 hpi (m.i) | 1 | 3 | ACT | -0.2957 | -0.00159 |
| IACSP95-5000 72 hpi (m.i) | 1 | 4 | ACT | -0.29423 | 4.92E-05 |
| IACSP95-5000 72 hpi (m.i) | 1 | 5 | ACT | -0.29395 | 0.000502 |
| IACSP95-5000 72 hpi (m.i) | 1 | 6 | ACT | -0.29333 | 0.001288 |
| IACSP95-5000 72 hpi (m.i) | 1 | 7 | ACT | -0.29401 | 0.000783 |
| IACSP95-5000 72 hpi (m.i) | 1 | 8 | ACT | -0.29296 | 0.002003 |
| IACSP95-5000 72 hpi (m.i) | 1 | 9 | ACT | -0.29379 | 0.001342 |
| IACSP95-5000 72 hpi (m.i) | 1 | 10 | ACT | -0.29409 | 0.001217 |
| IACSP95-5000 72 hpi (m.i) | 1 | 11 | ACT | -0.29466 | 0.000808 |
| IACSP95-5000 72 hpi (m.i) | 1 | 12 | ACT | -0.29451 | 0.001138 |
| IACSP95-5000 72 hpi (m.i) | 1 | 13 | ACT | -0.29716 | -0.00134 |
| IACSP95-5000 72 hpi (m.i) | 1 | 14 | ACT | -0.29681 | -0.00083 |
| IACSP95-5000 72 hpi (m.i) | 1 | 15 | ACT | -0.29654 | -0.00039 |
| IACSP95-5000 72 hpi (m.i) | 1 | 16 | ACT | -0.29794 | -0.00161 |
| IACSP95-5000 72 hpi (m.i) | 1 | 17 | ACT | -0.2974 | -0.00091 |
| IACSP95-5000 72 hpi (m.i) | 1 | 18 | ACT | -0.29696 | -0.00029 |
| IACSP95-5000 72 hpi (m.i) | 1 | 19 | ACT | -0.29959 | -0.00275 |
| IACSP95-5000 72 hpi (m.i) | 1 | 20 | ACT | -0.29783 | -0.00083 |
| IACSP95-5000 72 hpi (m.i) | 1 | 21 | ACT | -0.3 | -0.00283 |
| IACSP95-5000 72 hpi (m.i) | 1 | 22 | ACT | -0.2993 | -0.00196 |
| IACSP95-5000 72 hpi (m.i) | 1 | 23 | ACT | -0.29888 | -0.00137 |
| IACSP95-5000 72 hpi (m.i) | 1 | 24 | ACT | -0.30049 | -0.0028 |
| IACSP95-5000 72 hpi (m.i) | 1 | 25 | ACT | -0.29852 | -0.00067 |
| IACSP95-5000 72 hpi (m.i) | 1 | 26 | ACT | -0.29428 | 0.003743 |
| IACSP95-5000 72 hpi (m.i) | 1 | 27 | ACT | -0.2909 | 0.007292 |
| IACSP95-5000 72 hpi (m.i) | 1 | 28 | ACT | -0.28104 | 0.017326 |
| IACSP95-5000 72 hpi (m.i) | 1 | 29 | ACT | -0.26146 | 0.037075 |
| IACSP95-5000 72 hpi (m.i) | 1 | 30 | ACT | -0.22514 | 0.073562 |
| IACSP95-5000 72 hpi (m.i) | 1 | 31 | ACT | -0.15578 | 0.143093 |
| IACSP95-5000 72 hpi (m.i) | 1 | 32 | ACT | -0.01894 | 0.280101 |
| IACSP95-5000 72 hpi (m.i) | 1 | 33 | ACT | 0.228905 | 0.528121 |
| IACSP95-5000 72 hpi (m.i) | 1 | 34 | ACT | 0.665772 | 0.965158 |
| IACSP95-5000 72 hpi (m.i) | 1 | 35 | ACT | 1.339119 | 1.638675 |
| IACSP95-5000 72 hpi (m.i) | 1 | 36 | ACT | 2.193392 | 2.493118 |
| IACSP95-5000 72 hpi (m.i) | 1 | 37 | ACT | 3.055437 | 3.355333 |
| IACSP95-5000 72 hpi (m.i) | 1 | 38 | ACT | 3.79533 | 4.095397 |
| IACSP95-5000 72 hpi (m.i) | 1 | 39 | ACT | 4.456792 | 4.757029 |
| IACSP95-5000 72 hpi (m.i) | 1 | 40 | ACT | 5.038671 | 5.339078 |
| IACSP95-5000 72 hpi (m.i) | 2 | 1 | ACT | -0.32192 | -0.00852 |
| IACSP95-5000 72 hpi (m.i) | 2 | 2 | ACT | -0.31727 | -0.00414 |
| IACSP95-5000 72 hpi (m.i) | 2 | 3 | ACT | -0.31499 | -0.00213 |
| IACSP95-5000 72 hpi (m.i) | 2 | 4 | ACT | -0.31433 | -0.00174 |
| IACSP95-5000 72 hpi (m.i) | 2 | 5 | ACT | -0.31269 | -0.00036 |
| IACSP95-5000 72 hpi (m.i) | 2 | 6 | ACT | -0.31104 | 0.001014 |
| IACSP95-5000 72 hpi (m.i) | 2 | 7 | ACT | -0.31053 | 0.00126 |
| IACSP95-5000 72 hpi (m.i) | 2 | 8 | ACT | -0.30941 | 0.002103 |
| IACSP95-5000 72 hpi (m.i) | 2 | 9 | ACT | -0.30921 | 0.002035 |
| IACSP95-5000 72 hpi (m.i) | 2 | 10 | ACT | -0.30885 | 0.00213 |
| IACSP95-5000 72 hpi (m.i) | 2 | 11 | ACT | -0.30916 | 0.001551 |
| IACSP95-5000 72 hpi (m.i) | 2 | 12 | ACT | -0.30794 | 0.002505 |
| IACSP95-5000 72 hpi (m.i) | 2 | 13 | ACT | -0.31095 | -0.00077 |
| IACSP95-5000 72 hpi (m.i) | 2 | 14 | ACT | -0.30847 | 0.001441 |
| IACSP95-5000 72 hpi (m.i) | 2 | 15 | ACT | -0.3113 | -0.00166 |
| IACSP95-5000 72 hpi (m.i) | 2 | 16 | ACT | -0.31087 | -0.0015 |
| IACSP95-5000 72 hpi (m.i) | 2 | 17 | ACT | -0.31147 | -0.00237 |
| IACSP95-5000 72 hpi (m.i) | 2 | 18 | ACT | -0.31146 | -0.00263 |
| IACSP95-5000 72 hpi (m.i) | 2 | 19 | ACT | -0.31033 | -0.00176 |
| IACSP95-5000 72 hpi (m.i) | 2 | 20 | ACT | -0.30869 | -0.00039 |
| IACSP95-5000 72 hpi (m.i) | 2 | 21 | ACT | -0.31132 | -0.00328 |
| IACSP95-5000 72 hpi (m.i) | 2 | 22 | ACT | -0.3101 | -0.00234 |
| IACSP95-5000 72 hpi (m.i) | 2 | 23 | ACT | -0.30743 | 6.45E-05 |
| IACSP95-5000 72 hpi (m.i) | 2 | 24 | ACT | -0.30732 | -9.1E-05 |
| IACSP95-5000 72 hpi (m.i) | 2 | 25 | ACT | -0.30707 | -0.00011 |
| IACSP95-5000 72 hpi (m.i) | 2 | 26 | ACT | -0.305 | 0.001696 |
| IACSP95-5000 72 hpi (m.i) | 2 | 27 | ACT | -0.30108 | 0.005339 |
| IACSP95-5000 72 hpi (m.i) | 2 | 28 | ACT | -0.29434 | 0.011816 |
| IACSP95-5000 72 hpi (m.i) | 2 | 29 | ACT | -0.2797 | 0.026186 |
| IACSP95-5000 72 hpi (m.i) | 2 | 30 | ACT | -0.25319 | 0.052433 |
| IACSP95-5000 72 hpi (m.i) | 2 | 31 | ACT | -0.19838 | 0.10697 |
| IACSP95-5000 72 hpi (m.i) | 2 | 32 | ACT | -0.09513 | 0.209951 |
| IACSP95-5000 72 hpi (m.i) | 2 | 33 | ACT | 0.094854 | 0.399669 |
| IACSP95-5000 72 hpi (m.i) | 2 | 34 | ACT | 0.445463 | 0.75001 |
| IACSP95-5000 72 hpi (m.i) | 2 | 35 | ACT | 1.024215 | 1.328495 |
| IACSP95-5000 72 hpi (m.i) | 2 | 36 | ACT | 1.852781 | 2.156792 |
| IACSP95-5000 72 hpi (m.i) | 2 | 37 | ACT | 2.770921 | 3.074663 |
| IACSP95-5000 72 hpi (m.i) | 2 | 38 | ACT | 3.556633 | 3.860108 |
| IACSP95-5000 72 hpi (m.i) | 2 | 39 | ACT | 4.293618 | 4.596825 |
| IACSP95-5000 72 hpi (m.i) | 2 | 40 | ACT | 4.979211 | 5.282149 |
| IACSP95-5000 72 hpi (m.i) | 3 | 1 | ACT | -0.39095 | -0.00353 |
| IACSP95-5000 72 hpi (m.i) | 3 | 2 | ACT | -0.3896 | -0.00222 |
| IACSP95-5000 72 hpi (m.i) | 3 | 3 | ACT | -0.38707 | 0.000263 |
| IACSP95-5000 72 hpi (m.i) | 3 | 4 | ACT | -0.38494 | 0.002345 |
| IACSP95-5000 72 hpi (m.i) | 3 | 5 | ACT | -0.38506 | 0.002182 |
| IACSP95-5000 72 hpi (m.i) | 3 | 6 | ACT | -0.38697 | 0.000233 |
| IACSP95-5000 72 hpi (m.i) | 3 | 7 | ACT | -0.38429 | 0.002868 |
| IACSP95-5000 72 hpi (m.i) | 3 | 8 | ACT | -0.3861 | 0.001008 |
| IACSP95-5000 72 hpi (m.i) | 3 | 9 | ACT | -0.3898 | -0.00273 |
| IACSP95-5000 72 hpi (m.i) | 3 | 10 | ACT | -0.38687 | 0.000153 |
| IACSP95-5000 72 hpi (m.i) | 3 | 11 | ACT | -0.3905 | -0.00352 |
| IACSP95-5000 72 hpi (m.i) | 3 | 12 | ACT | -0.3875 | -0.00056 |
| IACSP95-5000 72 hpi (m.i) | 3 | 13 | ACT | -0.38318 | 0.003717 |
| IACSP95-5000 72 hpi (m.i) | 3 | 14 | ACT | -0.38931 | -0.00246 |
| IACSP95-5000 72 hpi (m.i) | 3 | 15 | ACT | -0.38643 | 0.000382 |
| IACSP95-5000 72 hpi (m.i) | 3 | 16 | ACT | -0.38803 | -0.00127 |
| IACSP95-5000 72 hpi (m.i) | 3 | 17 | ACT | -0.38791 | -0.00119 |
| IACSP95-5000 72 hpi (m.i) | 3 | 18 | ACT | -0.386 | 0.000681 |
| IACSP95-5000 72 hpi (m.i) | 3 | 19 | ACT | -0.3907 | -0.00407 |
| IACSP95-5000 72 hpi (m.i) | 3 | 20 | ACT | -0.38868 | -0.00209 |
| IACSP95-5000 72 hpi (m.i) | 3 | 21 | ACT | -0.38775 | -0.0012 |
| IACSP95-5000 72 hpi (m.i) | 3 | 22 | ACT | -0.38879 | -0.00229 |
| IACSP95-5000 72 hpi (m.i) | 3 | 23 | ACT | -0.38812 | -0.00166 |
| IACSP95-5000 72 hpi (m.i) | 3 | 24 | ACT | -0.38982 | -0.00341 |
| IACSP95-5000 72 hpi (m.i) | 3 | 25 | ACT | -0.3884 | -0.00203 |
| IACSP95-5000 72 hpi (m.i) | 3 | 26 | ACT | -0.38273 | 0.003593 |
| IACSP95-5000 72 hpi (m.i) | 3 | 27 | ACT | -0.37522 | 0.011068 |
| IACSP95-5000 72 hpi (m.i) | 3 | 28 | ACT | -0.36615 | 0.02009 |
| IACSP95-5000 72 hpi (m.i) | 3 | 29 | ACT | -0.3444 | 0.041792 |
| IACSP95-5000 72 hpi (m.i) | 3 | 30 | ACT | -0.301 | 0.085154 |
| IACSP95-5000 72 hpi (m.i) | 3 | 31 | ACT | -0.22065 | 0.165462 |
| IACSP95-5000 72 hpi (m.i) | 3 | 32 | ACT | -0.07235 | 0.313712 |
| IACSP95-5000 72 hpi (m.i) | 3 | 33 | ACT | 0.207898 | 0.59392 |
| IACSP95-5000 72 hpi (m.i) | 3 | 34 | ACT | 0.689321 | 1.075298 |
| IACSP95-5000 72 hpi (m.i) | 3 | 35 | ACT | 1.422206 | 1.80814 |
| IACSP95-5000 72 hpi (m.i) | 3 | 36 | ACT | 2.330934 | 2.716825 |
| IACSP95-5000 72 hpi (m.i) | 3 | 37 | ACT | 3.230111 | 3.615958 |
| IACSP95-5000 72 hpi (m.i) | 3 | 38 | ACT | 4.01239 | 4.398193 |
| IACSP95-5000 72 hpi (m.i) | 3 | 39 | ACT | 4.710487 | 5.096247 |
| IACSP95-5000 72 hpi (m.i) | 3 | 40 | ACT | 5.293506 | 5.679222 |
| IACSP95-5000 72 hpi (s.i) | 1 | 1 | ACT | -0.3248 | -0.01187 |
| IACSP95-5000 72 hpi (s.i) | 1 | 2 | ACT | -0.32099 | -0.0077 |
| IACSP95-5000 72 hpi (s.i) | 1 | 3 | ACT | -0.31491 | -0.00127 |
| IACSP95-5000 72 hpi (s.i) | 1 | 4 | ACT | -0.3141 | -0.00011 |
| IACSP95-5000 72 hpi (s.i) | 1 | 5 | ACT | -0.31551 | -0.00117 |
| IACSP95-5000 72 hpi (s.i) | 1 | 6 | ACT | -0.31503 | -0.00035 |
| IACSP95-5000 72 hpi (s.i) | 1 | 7 | ACT | -0.31411 | 0.000926 |
| IACSP95-5000 72 hpi (s.i) | 1 | 8 | ACT | -0.31374 | 0.001638 |
| IACSP95-5000 72 hpi (s.i) | 1 | 9 | ACT | -0.31382 | 0.001913 |
| IACSP95-5000 72 hpi (s.i) | 1 | 10 | ACT | -0.3144 | 0.001686 |
| IACSP95-5000 72 hpi (s.i) | 1 | 11 | ACT | -0.31643 | 8.8E-07 |
| IACSP95-5000 72 hpi (s.i) | 1 | 12 | ACT | -0.31662 | 0.00016 |
| IACSP95-5000 72 hpi (s.i) | 1 | 13 | ACT | -0.31793 | -0.0008 |
| IACSP95-5000 72 hpi (s.i) | 1 | 14 | ACT | -0.31801 | -0.00053 |
| IACSP95-5000 72 hpi (s.i) | 1 | 15 | ACT | -0.31446 | 0.003367 |
| IACSP95-5000 72 hpi (s.i) | 1 | 16 | ACT | -0.31822 | -4.6E-05 |
| IACSP95-5000 72 hpi (s.i) | 1 | 17 | ACT | -0.31973 | -0.0012 |
| IACSP95-5000 72 hpi (s.i) | 1 | 18 | ACT | -0.32065 | -0.00177 |
| IACSP95-5000 72 hpi (s.i) | 1 | 19 | ACT | -0.31768 | 0.001543 |
| IACSP95-5000 72 hpi (s.i) | 1 | 20 | ACT | -0.32129 | -0.00171 |
| IACSP95-5000 72 hpi (s.i) | 1 | 21 | ACT | -0.32162 | -0.00169 |
| IACSP95-5000 72 hpi (s.i) | 1 | 22 | ACT | -0.32243 | -0.00215 |
| IACSP95-5000 72 hpi (s.i) | 1 | 23 | ACT | -0.32254 | -0.00192 |
| IACSP95-5000 72 hpi (s.i) | 1 | 24 | ACT | -0.32296 | -0.00199 |
| IACSP95-5000 72 hpi (s.i) | 1 | 25 | ACT | -0.32297 | -0.00165 |
| IACSP95-5000 72 hpi (s.i) | 1 | 26 | ACT | -0.32203 | -0.00036 |
| IACSP95-5000 72 hpi (s.i) | 1 | 27 | ACT | -0.31953 | 0.002486 |
| IACSP95-5000 72 hpi (s.i) | 1 | 28 | ACT | -0.31736 | 0.005005 |
| IACSP95-5000 72 hpi (s.i) | 1 | 29 | ACT | -0.30747 | 0.015247 |
| IACSP95-5000 72 hpi (s.i) | 1 | 30 | ACT | -0.29166 | 0.03141 |
| IACSP95-5000 72 hpi (s.i) | 1 | 31 | ACT | -0.25829 | 0.065131 |
| IACSP95-5000 72 hpi (s.i) | 1 | 32 | ACT | -0.19493 | 0.128837 |
| IACSP95-5000 72 hpi (s.i) | 1 | 33 | ACT | -0.07214 | 0.251974 |
| IACSP95-5000 72 hpi (s.i) | 1 | 34 | ACT | 0.160208 | 0.484674 |
| IACSP95-5000 72 hpi (s.i) | 1 | 35 | ACT | 0.561138 | 0.885953 |
| IACSP95-5000 72 hpi (s.i) | 1 | 36 | ACT | 1.199995 | 1.525159 |
| IACSP95-5000 72 hpi (s.i) | 1 | 37 | ACT | 2.04594 | 2.371454 |
| IACSP95-5000 72 hpi (s.i) | 1 | 38 | ACT | 2.906765 | 3.232627 |
| IACSP95-5000 72 hpi (s.i) | 1 | 39 | ACT | 3.67558 | 4.001792 |
| IACSP95-5000 72 hpi (s.i) | 1 | 40 | ACT | 4.36364 | 4.690201 |
| IACSP95-5000 72 hpi (s.i) | 2 | 1 | ACT | -0.35402 | -0.01063 |
| IACSP95-5000 72 hpi (s.i) | 2 | 2 | ACT | -0.3502 | -0.00628 |
| IACSP95-5000 72 hpi (s.i) | 2 | 3 | ACT | -0.34726 | -0.0028 |
| IACSP95-5000 72 hpi (s.i) | 2 | 4 | ACT | -0.34484 | 0.000144 |
| IACSP95-5000 72 hpi (s.i) | 2 | 5 | ACT | -0.34331 | 0.002212 |
| IACSP95-5000 72 hpi (s.i) | 2 | 6 | ACT | -0.34434 | 0.001714 |
| IACSP95-5000 72 hpi (s.i) | 2 | 7 | ACT | -0.34557 | 0.001014 |
| IACSP95-5000 72 hpi (s.i) | 2 | 8 | ACT | -0.34637 | 0.000749 |
| IACSP95-5000 72 hpi (s.i) | 2 | 9 | ACT | -0.34623 | 0.001426 |
| IACSP95-5000 72 hpi (s.i) | 2 | 10 | ACT | -0.34659 | 0.001599 |
| IACSP95-5000 72 hpi (s.i) | 2 | 11 | ACT | -0.3499 | -0.00118 |
| IACSP95-5000 72 hpi (s.i) | 2 | 12 | ACT | -0.35 | -0.00075 |
| IACSP95-5000 72 hpi (s.i) | 2 | 13 | ACT | -0.34994 | -0.00015 |
| IACSP95-5000 72 hpi (s.i) | 2 | 14 | ACT | -0.35075 | -0.00043 |
| IACSP95-5000 72 hpi (s.i) | 2 | 15 | ACT | -0.35208 | -0.00123 |
| IACSP95-5000 72 hpi (s.i) | 2 | 16 | ACT | -0.35137 | 1.67E-05 |
| IACSP95-5000 72 hpi (s.i) | 2 | 17 | ACT | -0.35247 | -0.00055 |
| IACSP95-5000 72 hpi (s.i) | 2 | 18 | ACT | -0.35173 | 0.000722 |
| IACSP95-5000 72 hpi (s.i) | 2 | 19 | ACT | -0.35425 | -0.00127 |
| IACSP95-5000 72 hpi (s.i) | 2 | 20 | ACT | -0.35356 | -3.9E-05 |
| IACSP95-5000 72 hpi (s.i) | 2 | 21 | ACT | -0.35526 | -0.00121 |
| IACSP95-5000 72 hpi (s.i) | 2 | 22 | ACT | -0.3564 | -0.00182 |
| IACSP95-5000 72 hpi (s.i) | 2 | 23 | ACT | -0.35765 | -0.00253 |
| IACSP95-5000 72 hpi (s.i) | 2 | 24 | ACT | -0.35798 | -0.00233 |
| IACSP95-5000 72 hpi (s.i) | 2 | 25 | ACT | -0.35843 | -0.00225 |
| IACSP95-5000 72 hpi (s.i) | 2 | 26 | ACT | -0.35731 | -0.0006 |
| IACSP95-5000 72 hpi (s.i) | 2 | 27 | ACT | -0.35546 | 0.001792 |
| IACSP95-5000 72 hpi (s.i) | 2 | 28 | ACT | -0.35003 | 0.007747 |
| IACSP95-5000 72 hpi (s.i) | 2 | 29 | ACT | -0.34035 | 0.017968 |
| IACSP95-5000 72 hpi (s.i) | 2 | 30 | ACT | -0.31736 | 0.04149 |
| IACSP95-5000 72 hpi (s.i) | 2 | 31 | ACT | -0.27784 | 0.081544 |
| IACSP95-5000 72 hpi (s.i) | 2 | 32 | ACT | -0.19671 | 0.163207 |
| IACSP95-5000 72 hpi (s.i) | 2 | 33 | ACT | -0.04372 | 0.316728 |
| IACSP95-5000 72 hpi (s.i) | 2 | 34 | ACT | 0.242221 | 0.603202 |
| IACSP95-5000 72 hpi (s.i) | 2 | 35 | ACT | 0.740601 | 1.102115 |
| IACSP95-5000 72 hpi (s.i) | 2 | 36 | ACT | 1.503436 | 1.865483 |
| IACSP95-5000 72 hpi (s.i) | 2 | 37 | ACT | 2.429355 | 2.791934 |
| IACSP95-5000 72 hpi (s.i) | 2 | 38 | ACT | 3.251801 | 3.614913 |
| IACSP95-5000 72 hpi (s.i) | 2 | 39 | ACT | 3.967067 | 4.330713 |
| IACSP95-5000 72 hpi (s.i) | 2 | 40 | ACT | 4.602568 | 4.966747 |
| IACSP95-5000 72 hpi (s.i) | 3 | 1 | ACT | -0.33701 | -0.01303 |
| IACSP95-5000 72 hpi (s.i) | 3 | 2 | ACT | -0.33266 | -0.00863 |
| IACSP95-5000 72 hpi (s.i) | 3 | 3 | ACT | -0.32785 | -0.00376 |
| IACSP95-5000 72 hpi (s.i) | 3 | 4 | ACT | -0.32591 | -0.00177 |
| IACSP95-5000 72 hpi (s.i) | 3 | 5 | ACT | -0.32629 | -0.00209 |
| IACSP95-5000 72 hpi (s.i) | 3 | 6 | ACT | -0.32361 | 0.000639 |
| IACSP95-5000 72 hpi (s.i) | 3 | 7 | ACT | -0.32524 | -0.00094 |
| IACSP95-5000 72 hpi (s.i) | 3 | 8 | ACT | -0.32426 | 0.000101 |
| IACSP95-5000 72 hpi (s.i) | 3 | 9 | ACT | -0.32353 | 0.000884 |
| IACSP95-5000 72 hpi (s.i) | 3 | 10 | ACT | -0.32225 | 0.002214 |
| IACSP95-5000 72 hpi (s.i) | 3 | 11 | ACT | -0.32292 | 0.001603 |
| IACSP95-5000 72 hpi (s.i) | 3 | 12 | ACT | -0.32375 | 0.000826 |
| IACSP95-5000 72 hpi (s.i) | 3 | 13 | ACT | -0.32242 | 0.002204 |
| IACSP95-5000 72 hpi (s.i) | 3 | 14 | ACT | -0.32309 | 0.001595 |
| IACSP95-5000 72 hpi (s.i) | 3 | 15 | ACT | -0.32022 | 0.004517 |
| IACSP95-5000 72 hpi (s.i) | 3 | 16 | ACT | -0.32219 | 0.002599 |
| IACSP95-5000 72 hpi (s.i) | 3 | 17 | ACT | -0.32521 | -0.00037 |
| IACSP95-5000 72 hpi (s.i) | 3 | 18 | ACT | -0.32509 | -0.0002 |
| IACSP95-5000 72 hpi (s.i) | 3 | 19 | ACT | -0.32301 | 0.00194 |
| IACSP95-5000 72 hpi (s.i) | 3 | 20 | ACT | -0.32653 | -0.00153 |
| IACSP95-5000 72 hpi (s.i) | 3 | 21 | ACT | -0.32755 | -0.00249 |
| IACSP95-5000 72 hpi (s.i) | 3 | 22 | ACT | -0.32642 | -0.0013 |
| IACSP95-5000 72 hpi (s.i) | 3 | 23 | ACT | -0.32744 | -0.00227 |
| IACSP95-5000 72 hpi (s.i) | 3 | 24 | ACT | -0.328 | -0.00277 |
| IACSP95-5000 72 hpi (s.i) | 3 | 25 | ACT | -0.32811 | -0.00283 |
| IACSP95-5000 72 hpi (s.i) | 3 | 26 | ACT | -0.3255 | -0.00017 |
| IACSP95-5000 72 hpi (s.i) | 3 | 27 | ACT | -0.322 | 0.003383 |
| IACSP95-5000 72 hpi (s.i) | 3 | 28 | ACT | -0.31868 | 0.006761 |
| IACSP95-5000 72 hpi (s.i) | 3 | 29 | ACT | -0.30765 | 0.017845 |
| IACSP95-5000 72 hpi (s.i) | 3 | 30 | ACT | -0.2857 | 0.039848 |
| IACSP95-5000 72 hpi (s.i) | 3 | 31 | ACT | -0.24483 | 0.080768 |
| IACSP95-5000 72 hpi (s.i) | 3 | 32 | ACT | -0.16549 | 0.16017 |
| IACSP95-5000 72 hpi (s.i) | 3 | 33 | ACT | -0.01544 | 0.310269 |
| IACSP95-5000 72 hpi (s.i) | 3 | 34 | ACT | 0.266956 | 0.592719 |
| IACSP95-5000 72 hpi (s.i) | 3 | 35 | ACT | 0.753076 | 1.078893 |
| IACSP95-5000 72 hpi (s.i) | 3 | 36 | ACT | 1.512518 | 1.838389 |
| IACSP95-5000 72 hpi (s.i) | 3 | 37 | ACT | 2.493699 | 2.819624 |
| IACSP95-5000 72 hpi (s.i) | 3 | 38 | ACT | 3.456522 | 3.782501 |
| IACSP95-5000 72 hpi (s.i) | 3 | 39 | ACT | 4.311901 | 4.637934 |
| IACSP95-5000 72 hpi (s.i) | 3 | 40 | ACT | 5.068434 | 5.394521 |
| IAC91-1099 24 hpi (m.i) | 1 | 1 | GAPDH | -0.00013 | 0.018156 |
| IAC91-1099 24 hpi (m.i) | 1 | 2 | GAPDH | -0.01501 | 0.004858 |
| IAC91-1099 24 hpi (m.i) | 1 | 3 | GAPDH | -0.01913 | 0.002326 |
| IAC91-1099 24 hpi (m.i) | 1 | 4 | GAPDH | -0.02296 | 8.09E-05 |
| IAC91-1099 24 hpi (m.i) | 1 | 5 | GAPDH | -0.02454 | 7.91E-05 |
| IAC91-1099 24 hpi (m.i) | 1 | 6 | GAPDH | -0.02543 | 0.000774 |
| IAC91-1099 24 hpi (m.i) | 1 | 7 | GAPDH | -0.02615 | 0.001637 |
| IAC91-1099 24 hpi (m.i) | 1 | 8 | GAPDH | -0.02945 | -7.5E-05 |
| IAC91-1099 24 hpi (m.i) | 1 | 9 | GAPDH | -0.03249 | -0.00153 |
| IAC91-1099 24 hpi (m.i) | 1 | 10 | GAPDH | -0.03245 | 8.83E-05 |
| IAC91-1099 24 hpi (m.i) | 1 | 11 | GAPDH | -0.03674 | -0.00262 |
| IAC91-1099 24 hpi (m.i) | 1 | 12 | GAPDH | -0.03818 | -0.00247 |
| IAC91-1099 24 hpi (m.i) | 1 | 13 | GAPDH | -0.03875 | -0.00146 |
| IAC91-1099 24 hpi (m.i) | 1 | 14 | GAPDH | -0.04059 | -0.00172 |
| IAC91-1099 24 hpi (m.i) | 1 | 15 | GAPDH | -0.04118 | -0.00072 |
| IAC91-1099 24 hpi (m.i) | 1 | 16 | GAPDH | -0.04275 | -0.00072 |
| IAC91-1099 24 hpi (m.i) | 1 | 17 | GAPDH | -0.04317 | 0.000451 |
| IAC91-1099 24 hpi (m.i) | 1 | 18 | GAPDH | -0.03932 | 0.005883 |
| IAC91-1099 24 hpi (m.i) | 1 | 19 | GAPDH | -0.03235 | 0.014434 |
| IAC91-1099 24 hpi (m.i) | 1 | 20 | GAPDH | -0.0146 | 0.033765 |
| IAC91-1099 24 hpi (m.i) | 1 | 21 | GAPDH | 0.020922 | 0.070875 |
| IAC91-1099 24 hpi (m.i) | 1 | 22 | GAPDH | 0.090117 | 0.141653 |
| IAC91-1099 24 hpi (m.i) | 1 | 23 | GAPDH | 0.223751 | 0.27687 |
| IAC91-1099 24 hpi (m.i) | 1 | 24 | GAPDH | 0.471809 | 0.526511 |
| IAC91-1099 24 hpi (m.i) | 1 | 25 | GAPDH | 0.895235 | 0.95152 |
| IAC91-1099 24 hpi (m.i) | 1 | 26 | GAPDH | 1.480892 | 1.538761 |
| IAC91-1099 24 hpi (m.i) | 1 | 27 | GAPDH | 2.08568 | 2.145132 |
| IAC91-1099 24 hpi (m.i) | 1 | 28 | GAPDH | 2.632706 | 2.693741 |
| IAC91-1099 24 hpi (m.i) | 1 | 29 | GAPDH | 3.148687 | 3.211305 |
| IAC91-1099 24 hpi (m.i) | 1 | 30 | GAPDH | 3.660711 | 3.724912 |
| IAC91-1099 24 hpi (m.i) | 1 | 31 | GAPDH | 4.097876 | 4.16366 |
| IAC91-1099 24 hpi (m.i) | 1 | 32 | GAPDH | 4.514784 | 4.582152 |
| IAC91-1099 24 hpi (m.i) | 1 | 33 | GAPDH | 4.870904 | 4.939855 |
| IAC91-1099 24 hpi (m.i) | 1 | 34 | GAPDH | 5.216309 | 5.286843 |
| IAC91-1099 24 hpi (m.i) | 1 | 35 | GAPDH | 5.501719 | 5.573836 |
| IAC91-1099 24 hpi (m.i) | 1 | 36 | GAPDH | 5.757589 | 5.831289 |
| IAC91-1099 24 hpi (m.i) | 1 | 37 | GAPDH | 6.013861 | 6.089145 |
| IAC91-1099 24 hpi (m.i) | 1 | 38 | GAPDH | 6.210086 | 6.286952 |
| IAC91-1099 24 hpi (m.i) | 1 | 39 | GAPDH | 6.391893 | 6.470343 |
| IAC91-1099 24 hpi (m.i) | 1 | 40 | GAPDH | 6.580232 | 6.660264 |
| IAC91-1099 24 hpi (m.i) | 2 | 1 | GAPDH | 0.028659 | 0.024401 |
| IAC91-1099 24 hpi (m.i) | 2 | 2 | GAPDH | 0.013091 | 0.010221 |
| IAC91-1099 24 hpi (m.i) | 2 | 3 | GAPDH | 0.004266 | 0.002785 |
| IAC91-1099 24 hpi (m.i) | 2 | 4 | GAPDH | 0.002929 | 0.002836 |
| IAC91-1099 24 hpi (m.i) | 2 | 5 | GAPDH | -0.00095 | 0.000343 |
| IAC91-1099 24 hpi (m.i) | 2 | 6 | GAPDH | -0.00155 | 0.001132 |
| IAC91-1099 24 hpi (m.i) | 2 | 7 | GAPDH | -0.00349 | 0.000587 |
| IAC91-1099 24 hpi (m.i) | 2 | 8 | GAPDH | -0.00577 | -0.00031 |
| IAC91-1099 24 hpi (m.i) | 2 | 9 | GAPDH | -0.00892 | -0.00208 |
| IAC91-1099 24 hpi (m.i) | 2 | 10 | GAPDH | -0.00907 | -0.00083 |
| IAC91-1099 24 hpi (m.i) | 2 | 11 | GAPDH | -0.01315 | -0.00352 |
| IAC91-1099 24 hpi (m.i) | 2 | 12 | GAPDH | -0.01438 | -0.00336 |
| IAC91-1099 24 hpi (m.i) | 2 | 13 | GAPDH | -0.01628 | -0.00388 |
| IAC91-1099 24 hpi (m.i) | 2 | 14 | GAPDH | -0.01605 | -0.00226 |
| IAC91-1099 24 hpi (m.i) | 2 | 15 | GAPDH | -0.01714 | -0.00196 |
| IAC91-1099 24 hpi (m.i) | 2 | 16 | GAPDH | -0.01719 | -0.00062 |
| IAC91-1099 24 hpi (m.i) | 2 | 17 | GAPDH | -0.01514 | 0.00282 |
| IAC91-1099 24 hpi (m.i) | 2 | 18 | GAPDH | -0.01103 | 0.008319 |
| IAC91-1099 24 hpi (m.i) | 2 | 19 | GAPDH | -0.00403 | 0.016701 |
| IAC91-1099 24 hpi (m.i) | 2 | 20 | GAPDH | 0.016555 | 0.038676 |
| IAC91-1099 24 hpi (m.i) | 2 | 21 | GAPDH | 0.053856 | 0.077366 |
| IAC91-1099 24 hpi (m.i) | 2 | 22 | GAPDH | 0.12908 | 0.153978 |
| IAC91-1099 24 hpi (m.i) | 2 | 23 | GAPDH | 0.271446 | 0.297732 |
| IAC91-1099 24 hpi (m.i) | 2 | 24 | GAPDH | 0.533968 | 0.561642 |
| IAC91-1099 24 hpi (m.i) | 2 | 25 | GAPDH | 0.983981 | 1.013043 |
| IAC91-1099 24 hpi (m.i) | 2 | 26 | GAPDH | 1.588503 | 1.618953 |
| IAC91-1099 24 hpi (m.i) | 2 | 27 | GAPDH | 2.207226 | 2.239065 |
| IAC91-1099 24 hpi (m.i) | 2 | 28 | GAPDH | 2.760228 | 2.793455 |
| IAC91-1099 24 hpi (m.i) | 2 | 29 | GAPDH | 3.2863 | 3.320916 |
| IAC91-1099 24 hpi (m.i) | 2 | 30 | GAPDH | 3.788905 | 3.824909 |
| IAC91-1099 24 hpi (m.i) | 2 | 31 | GAPDH | 4.235491 | 4.272883 |
| IAC91-1099 24 hpi (m.i) | 2 | 32 | GAPDH | 4.662046 | 4.700827 |
| IAC91-1099 24 hpi (m.i) | 2 | 33 | GAPDH | 5.00921 | 5.049379 |
| IAC91-1099 24 hpi (m.i) | 2 | 34 | GAPDH | 5.347572 | 5.38913 |
| IAC91-1099 24 hpi (m.i) | 2 | 35 | GAPDH | 5.636744 | 5.67969 |
| IAC91-1099 24 hpi (m.i) | 2 | 36 | GAPDH | 5.890235 | 5.934569 |
| IAC91-1099 24 hpi (m.i) | 2 | 37 | GAPDH | 6.12716 | 6.172883 |
| IAC91-1099 24 hpi (m.i) | 2 | 38 | GAPDH | 6.333432 | 6.380543 |
| IAC91-1099 24 hpi (m.i) | 2 | 39 | GAPDH | 6.518561 | 6.56706 |
| IAC91-1099 24 hpi (m.i) | 2 | 40 | GAPDH | 6.691981 | 6.741868 |
| IAC91-1099 24 hpi (m.i) | 3 | 1 | GAPDH | 0.034827 | 0.027149 |
| IAC91-1099 24 hpi (m.i) | 3 | 2 | GAPDH | 0.017911 | 0.011136 |
| IAC91-1099 24 hpi (m.i) | 3 | 3 | GAPDH | 0.010688 | 0.004818 |
| IAC91-1099 24 hpi (m.i) | 3 | 4 | GAPDH | 0.007907 | 0.002941 |
| IAC91-1099 24 hpi (m.i) | 3 | 5 | GAPDH | 0.004236 | 0.000173 |
| IAC91-1099 24 hpi (m.i) | 3 | 6 | GAPDH | 0.00314 | -1.8E-05 |
| IAC91-1099 24 hpi (m.i) | 3 | 7 | GAPDH | 0.00246 | 0.000206 |
| IAC91-1099 24 hpi (m.i) | 3 | 8 | GAPDH | 0.000671 | -0.00068 |
| IAC91-1099 24 hpi (m.i) | 3 | 9 | GAPDH | -0.00159 | -0.00204 |
| IAC91-1099 24 hpi (m.i) | 3 | 10 | GAPDH | -0.00337 | -0.00291 |
| IAC91-1099 24 hpi (m.i) | 3 | 11 | GAPDH | -0.00435 | -0.00299 |
| IAC91-1099 24 hpi (m.i) | 3 | 12 | GAPDH | -0.00462 | -0.00235 |
| IAC91-1099 24 hpi (m.i) | 3 | 13 | GAPDH | -0.00677 | -0.0036 |
| IAC91-1099 24 hpi (m.i) | 3 | 14 | GAPDH | -0.00773 | -0.00366 |
| IAC91-1099 24 hpi (m.i) | 3 | 15 | GAPDH | -0.00666 | -0.00168 |
| IAC91-1099 24 hpi (m.i) | 3 | 16 | GAPDH | -0.00515 | 0.000731 |
| IAC91-1099 24 hpi (m.i) | 3 | 17 | GAPDH | -0.00484 | 0.001949 |
| IAC91-1099 24 hpi (m.i) | 3 | 18 | GAPDH | 0.001423 | 0.009113 |
| IAC91-1099 24 hpi (m.i) | 3 | 19 | GAPDH | 0.009067 | 0.017661 |
| IAC91-1099 24 hpi (m.i) | 3 | 20 | GAPDH | 0.029213 | 0.038711 |
| IAC91-1099 24 hpi (m.i) | 3 | 21 | GAPDH | 0.068007 | 0.078409 |
| IAC91-1099 24 hpi (m.i) | 3 | 22 | GAPDH | 0.142913 | 0.154219 |
| IAC91-1099 24 hpi (m.i) | 3 | 23 | GAPDH | 0.285497 | 0.297707 |
| IAC91-1099 24 hpi (m.i) | 3 | 24 | GAPDH | 0.54898 | 0.562094 |
| IAC91-1099 24 hpi (m.i) | 3 | 25 | GAPDH | 1.00107 | 1.015088 |
| IAC91-1099 24 hpi (m.i) | 3 | 26 | GAPDH | 1.609997 | 1.624919 |
| IAC91-1099 24 hpi (m.i) | 3 | 27 | GAPDH | 2.224253 | 2.240079 |
| IAC91-1099 24 hpi (m.i) | 3 | 28 | GAPDH | 2.781044 | 2.797775 |
| IAC91-1099 24 hpi (m.i) | 3 | 29 | GAPDH | 3.294825 | 3.312459 |
| IAC91-1099 24 hpi (m.i) | 3 | 30 | GAPDH | 3.794221 | 3.812759 |
| IAC91-1099 24 hpi (m.i) | 3 | 31 | GAPDH | 4.22955 | 4.248992 |
| IAC91-1099 24 hpi (m.i) | 3 | 32 | GAPDH | 4.63262 | 4.652966 |
| IAC91-1099 24 hpi (m.i) | 3 | 33 | GAPDH | 4.981186 | 5.002437 |
| IAC91-1099 24 hpi (m.i) | 3 | 34 | GAPDH | 5.301836 | 5.323991 |
| IAC91-1099 24 hpi (m.i) | 3 | 35 | GAPDH | 5.584188 | 5.607246 |
| IAC91-1099 24 hpi (m.i) | 3 | 36 | GAPDH | 5.832624 | 5.856586 |
| IAC91-1099 24 hpi (m.i) | 3 | 37 | GAPDH | 6.06977 | 6.094636 |
| IAC91-1099 24 hpi (m.i) | 3 | 38 | GAPDH | 6.259374 | 6.285144 |
| IAC91-1099 24 hpi (m.i) | 3 | 39 | GAPDH | 6.427544 | 6.454218 |
| IAC91-1099 24 hpi (m.i) | 3 | 40 | GAPDH | 6.606223 | 6.633801 |
| IAC91-1099 24 hpi (s.i) | 1 | 1 | GAPDH | -0.28445 | -0.00453 |
| IAC91-1099 24 hpi (s.i) | 1 | 2 | GAPDH | -0.27964 | 0.000507 |
| IAC91-1099 24 hpi (s.i) | 1 | 3 | GAPDH | -0.27755 | 0.002825 |
| IAC91-1099 24 hpi (s.i) | 1 | 4 | GAPDH | -0.27842 | 0.002184 |
| IAC91-1099 24 hpi (s.i) | 1 | 5 | GAPDH | -0.28075 | 7.42E-05 |
| IAC91-1099 24 hpi (s.i) | 1 | 6 | GAPDH | -0.28064 | 0.000412 |
| IAC91-1099 24 hpi (s.i) | 1 | 7 | GAPDH | -0.28106 | 0.000222 |
| IAC91-1099 24 hpi (s.i) | 1 | 8 | GAPDH | -0.28222 | -0.00071 |
| IAC91-1099 24 hpi (s.i) | 1 | 9 | GAPDH | -0.28343 | -0.00169 |
| IAC91-1099 24 hpi (s.i) | 1 | 10 | GAPDH | -0.28526 | -0.00329 |
| IAC91-1099 24 hpi (s.i) | 1 | 11 | GAPDH | -0.28489 | -0.0027 |
| IAC91-1099 24 hpi (s.i) | 1 | 12 | GAPDH | -0.28419 | -0.00177 |
| IAC91-1099 24 hpi (s.i) | 1 | 13 | GAPDH | -0.28389 | -0.00124 |
| IAC91-1099 24 hpi (s.i) | 1 | 14 | GAPDH | -0.28386 | -0.00098 |
| IAC91-1099 24 hpi (s.i) | 1 | 15 | GAPDH | -0.28383 | -0.00073 |
| IAC91-1099 24 hpi (s.i) | 1 | 16 | GAPDH | -0.28143 | 0.001893 |
| IAC91-1099 24 hpi (s.i) | 1 | 17 | GAPDH | -0.27803 | 0.005524 |
| IAC91-1099 24 hpi (s.i) | 1 | 18 | GAPDH | -0.27169 | 0.012092 |
| IAC91-1099 24 hpi (s.i) | 1 | 19 | GAPDH | -0.2559 | 0.028109 |
| IAC91-1099 24 hpi (s.i) | 1 | 20 | GAPDH | -0.22143 | 0.0628 |
| IAC91-1099 24 hpi (s.i) | 1 | 21 | GAPDH | -0.16115 | 0.123308 |
| IAC91-1099 24 hpi (s.i) | 1 | 22 | GAPDH | -0.04275 | 0.241934 |
| IAC91-1099 24 hpi (s.i) | 1 | 23 | GAPDH | 0.183315 | 0.468231 |
| IAC91-1099 24 hpi (s.i) | 1 | 24 | GAPDH | 0.582803 | 0.867947 |
| IAC91-1099 24 hpi (s.i) | 1 | 25 | GAPDH | 1.200252 | 1.485622 |
| IAC91-1099 24 hpi (s.i) | 1 | 26 | GAPDH | 1.863961 | 2.149558 |
| IAC91-1099 24 hpi (s.i) | 1 | 27 | GAPDH | 2.492076 | 2.777901 |
| IAC91-1099 24 hpi (s.i) | 1 | 28 | GAPDH | 3.037807 | 3.323858 |
| IAC91-1099 24 hpi (s.i) | 1 | 29 | GAPDH | 3.543802 | 3.830081 |
| IAC91-1099 24 hpi (s.i) | 1 | 30 | GAPDH | 4.033279 | 4.319786 |
| IAC91-1099 24 hpi (s.i) | 1 | 31 | GAPDH | 4.448318 | 4.735051 |
| IAC91-1099 24 hpi (s.i) | 1 | 32 | GAPDH | 4.838976 | 5.125936 |
| IAC91-1099 24 hpi (s.i) | 1 | 33 | GAPDH | 5.171582 | 5.45877 |
| IAC91-1099 24 hpi (s.i) | 1 | 34 | GAPDH | 5.466448 | 5.753862 |
| IAC91-1099 24 hpi (s.i) | 1 | 35 | GAPDH | 5.729013 | 6.016654 |
| IAC91-1099 24 hpi (s.i) | 1 | 36 | GAPDH | 5.960675 | 6.248544 |
| IAC91-1099 24 hpi (s.i) | 1 | 37 | GAPDH | 6.175382 | 6.463478 |
| IAC91-1099 24 hpi (s.i) | 1 | 38 | GAPDH | 6.360595 | 6.648918 |
| IAC91-1099 24 hpi (s.i) | 1 | 39 | GAPDH | 6.524786 | 6.813335 |
| IAC91-1099 24 hpi (s.i) | 1 | 40 | GAPDH | 6.669657 | 6.958434 |
| IAC91-1099 24 hpi (s.i) | 2 | 1 | GAPDH | -0.27681 | 0.00118 |
| IAC91-1099 24 hpi (s.i) | 2 | 2 | GAPDH | -0.27452 | 0.003312 |
| IAC91-1099 24 hpi (s.i) | 2 | 3 | GAPDH | -0.27456 | 0.003113 |
| IAC91-1099 24 hpi (s.i) | 2 | 4 | GAPDH | -0.275 | 0.002517 |
| IAC91-1099 24 hpi (s.i) | 2 | 5 | GAPDH | -0.27558 | 0.001777 |
| IAC91-1099 24 hpi (s.i) | 2 | 6 | GAPDH | -0.27745 | -0.00025 |
| IAC91-1099 24 hpi (s.i) | 2 | 7 | GAPDH | -0.27629 | 0.000744 |
| IAC91-1099 24 hpi (s.i) | 2 | 8 | GAPDH | -0.27984 | -0.00296 |
| IAC91-1099 24 hpi (s.i) | 2 | 9 | GAPDH | -0.2779 | -0.00119 |
| IAC91-1099 24 hpi (s.i) | 2 | 10 | GAPDH | -0.279 | -0.00244 |
| IAC91-1099 24 hpi (s.i) | 2 | 11 | GAPDH | -0.28124 | -0.00483 |
| IAC91-1099 24 hpi (s.i) | 2 | 12 | GAPDH | -0.27875 | -0.0025 |
| IAC91-1099 24 hpi (s.i) | 2 | 13 | GAPDH | -0.27723 | -0.00114 |
| IAC91-1099 24 hpi (s.i) | 2 | 14 | GAPDH | -0.27803 | -0.00211 |
| IAC91-1099 24 hpi (s.i) | 2 | 15 | GAPDH | -0.2749 | 0.000868 |
| IAC91-1099 24 hpi (s.i) | 2 | 16 | GAPDH | -0.27395 | 0.001656 |
| IAC91-1099 24 hpi (s.i) | 2 | 17 | GAPDH | -0.26869 | 0.006761 |
| IAC91-1099 24 hpi (s.i) | 2 | 18 | GAPDH | -0.26134 | 0.013951 |
| IAC91-1099 24 hpi (s.i) | 2 | 19 | GAPDH | -0.24477 | 0.030362 |
| IAC91-1099 24 hpi (s.i) | 2 | 20 | GAPDH | -0.2101 | 0.064866 |
| IAC91-1099 24 hpi (s.i) | 2 | 21 | GAPDH | -0.14793 | 0.126879 |
| IAC91-1099 24 hpi (s.i) | 2 | 22 | GAPDH | -0.02483 | 0.249821 |
| IAC91-1099 24 hpi (s.i) | 2 | 23 | GAPDH | 0.207354 | 0.481847 |
| IAC91-1099 24 hpi (s.i) | 2 | 24 | GAPDH | 0.620187 | 0.894521 |
| IAC91-1099 24 hpi (s.i) | 2 | 25 | GAPDH | 1.252248 | 1.526423 |
| IAC91-1099 24 hpi (s.i) | 2 | 26 | GAPDH | 1.934933 | 2.208949 |
| IAC91-1099 24 hpi (s.i) | 2 | 27 | GAPDH | 2.572482 | 2.846338 |
| IAC91-1099 24 hpi (s.i) | 2 | 28 | GAPDH | 3.138906 | 3.412603 |
| IAC91-1099 24 hpi (s.i) | 2 | 29 | GAPDH | 3.671549 | 3.945088 |
| IAC91-1099 24 hpi (s.i) | 2 | 30 | GAPDH | 4.176793 | 4.450172 |
| IAC91-1099 24 hpi (s.i) | 2 | 31 | GAPDH | 4.616438 | 4.889659 |
| IAC91-1099 24 hpi (s.i) | 2 | 32 | GAPDH | 5.025848 | 5.298909 |
| IAC91-1099 24 hpi (s.i) | 2 | 33 | GAPDH | 5.384216 | 5.657119 |
| IAC91-1099 24 hpi (s.i) | 2 | 34 | GAPDH | 5.69633 | 5.969073 |
| IAC91-1099 24 hpi (s.i) | 2 | 35 | GAPDH | 5.972146 | 6.24473 |
| IAC91-1099 24 hpi (s.i) | 2 | 36 | GAPDH | 6.221296 | 6.493721 |
| IAC91-1099 24 hpi (s.i) | 2 | 37 | GAPDH | 6.457398 | 6.729665 |
| IAC91-1099 24 hpi (s.i) | 2 | 38 | GAPDH | 6.647171 | 6.919279 |
| IAC91-1099 24 hpi (s.i) | 2 | 39 | GAPDH | 6.821576 | 7.093524 |
| IAC91-1099 24 hpi (s.i) | 2 | 40 | GAPDH | 6.992263 | 7.264052 |
| IAC91-1099 24 hpi (s.i) | 3 | 1 | GAPDH | -0.28625 | -0.00921 |
| IAC91-1099 24 hpi (s.i) | 3 | 2 | GAPDH | -0.28142 | -0.00402 |
| IAC91-1099 24 hpi (s.i) | 3 | 3 | GAPDH | -0.28061 | -0.00283 |
| IAC91-1099 24 hpi (s.i) | 3 | 4 | GAPDH | -0.27617 | 0.00197 |
| IAC91-1099 24 hpi (s.i) | 3 | 5 | GAPDH | -0.27778 | 0.000735 |
| IAC91-1099 24 hpi (s.i) | 3 | 6 | GAPDH | -0.27907 | -0.00019 |
| IAC91-1099 24 hpi (s.i) | 3 | 7 | GAPDH | -0.27848 | 0.00077 |
| IAC91-1099 24 hpi (s.i) | 3 | 8 | GAPDH | -0.27931 | 0.000311 |
| IAC91-1099 24 hpi (s.i) | 3 | 9 | GAPDH | -0.27927 | 0.000721 |
| IAC91-1099 24 hpi (s.i) | 3 | 10 | GAPDH | -0.28033 | 2.91E-05 |
| IAC91-1099 24 hpi (s.i) | 3 | 11 | GAPDH | -0.28143 | -0.0007 |
| IAC91-1099 24 hpi (s.i) | 3 | 12 | GAPDH | -0.28018 | 0.00091 |
| IAC91-1099 24 hpi (s.i) | 3 | 13 | GAPDH | -0.28219 | -0.00073 |
| IAC91-1099 24 hpi (s.i) | 3 | 14 | GAPDH | -0.28255 | -0.00072 |
| IAC91-1099 24 hpi (s.i) | 3 | 15 | GAPDH | -0.28436 | -0.00216 |
| IAC91-1099 24 hpi (s.i) | 3 | 16 | GAPDH | -0.28263 | -6.8E-05 |
| IAC91-1099 24 hpi (s.i) | 3 | 17 | GAPDH | -0.28097 | 0.001966 |
| IAC91-1099 24 hpi (s.i) | 3 | 18 | GAPDH | -0.27266 | 0.010643 |
| IAC91-1099 24 hpi (s.i) | 3 | 19 | GAPDH | -0.25409 | 0.029581 |
| IAC91-1099 24 hpi (s.i) | 3 | 20 | GAPDH | -0.22062 | 0.063418 |
| IAC91-1099 24 hpi (s.i) | 3 | 21 | GAPDH | -0.1536 | 0.130805 |
| IAC91-1099 24 hpi (s.i) | 3 | 22 | GAPDH | -0.02538 | 0.259398 |
| IAC91-1099 24 hpi (s.i) | 3 | 23 | GAPDH | 0.216097 | 0.501244 |
| IAC91-1099 24 hpi (s.i) | 3 | 24 | GAPDH | 0.64086 | 0.926374 |
| IAC91-1099 24 hpi (s.i) | 3 | 25 | GAPDH | 1.292706 | 1.57859 |
| IAC91-1099 24 hpi (s.i) | 3 | 26 | GAPDH | 1.998134 | 2.284386 |
| IAC91-1099 24 hpi (s.i) | 3 | 27 | GAPDH | 2.657098 | 2.943719 |
| IAC91-1099 24 hpi (s.i) | 3 | 28 | GAPDH | 3.231966 | 3.518955 |
| IAC91-1099 24 hpi (s.i) | 3 | 29 | GAPDH | 3.761659 | 4.049016 |
| IAC91-1099 24 hpi (s.i) | 3 | 30 | GAPDH | 4.278125 | 4.565851 |
| IAC91-1099 24 hpi (s.i) | 3 | 31 | GAPDH | 4.716429 | 5.004524 |
| IAC91-1099 24 hpi (s.i) | 3 | 32 | GAPDH | 5.123722 | 5.412185 |
| IAC91-1099 24 hpi (s.i) | 3 | 33 | GAPDH | 5.470405 | 5.759237 |
| IAC91-1099 24 hpi (s.i) | 3 | 34 | GAPDH | 5.799456 | 6.088656 |
| IAC91-1099 24 hpi (s.i) | 3 | 35 | GAPDH | 6.072129 | 6.361698 |
| IAC91-1099 24 hpi (s.i) | 3 | 36 | GAPDH | 6.326695 | 6.616632 |
| IAC91-1099 24 hpi (s.i) | 3 | 37 | GAPDH | 6.562493 | 6.852798 |
| IAC91-1099 24 hpi (s.i) | 3 | 38 | GAPDH | 6.739717 | 7.030392 |
| IAC91-1099 24 hpi (s.i) | 3 | 39 | GAPDH | 6.935575 | 7.226618 |
| IAC91-1099 24 hpi (s.i) | 3 | 40 | GAPDH | 7.103823 | 7.395234 |
| IAC91-1099 72 hpi (m.i) | 1 | 1 | GAPDH | -0.31971 | -0.01032 |
| IAC91-1099 72 hpi (m.i) | 1 | 2 | GAPDH | -0.31195 | -0.00221 |
| IAC91-1099 72 hpi (m.i) | 1 | 3 | GAPDH | -0.31228 | -0.0022 |
| IAC91-1099 72 hpi (m.i) | 1 | 4 | GAPDH | -0.31064 | -0.00021 |
| IAC91-1099 72 hpi (m.i) | 1 | 5 | GAPDH | -0.30982 | 0.000962 |
| IAC91-1099 72 hpi (m.i) | 1 | 6 | GAPDH | -0.30775 | 0.003374 |
| IAC91-1099 72 hpi (m.i) | 1 | 7 | GAPDH | -0.30939 | 0.00208 |
| IAC91-1099 72 hpi (m.i) | 1 | 8 | GAPDH | -0.31032 | 0.001501 |
| IAC91-1099 72 hpi (m.i) | 1 | 9 | GAPDH | -0.3107 | 0.001469 |
| IAC91-1099 72 hpi (m.i) | 1 | 10 | GAPDH | -0.31318 | -0.00066 |
| IAC91-1099 72 hpi (m.i) | 1 | 11 | GAPDH | -0.31471 | -0.00184 |
| IAC91-1099 72 hpi (m.i) | 1 | 12 | GAPDH | -0.31483 | -0.00162 |
| IAC91-1099 72 hpi (m.i) | 1 | 13 | GAPDH | -0.3162 | -0.00264 |
| IAC91-1099 72 hpi (m.i) | 1 | 14 | GAPDH | -0.31709 | -0.00318 |
| IAC91-1099 72 hpi (m.i) | 1 | 15 | GAPDH | -0.31728 | -0.00303 |
| IAC91-1099 72 hpi (m.i) | 1 | 16 | GAPDH | -0.31741 | -0.00281 |
| IAC91-1099 72 hpi (m.i) | 1 | 17 | GAPDH | -0.31472 | 0.000232 |
| IAC91-1099 72 hpi (m.i) | 1 | 18 | GAPDH | -0.30675 | 0.008556 |
| IAC91-1099 72 hpi (m.i) | 1 | 19 | GAPDH | -0.29684 | 0.01881 |
| IAC91-1099 72 hpi (m.i) | 1 | 20 | GAPDH | -0.27006 | 0.045937 |
| IAC91-1099 72 hpi (m.i) | 1 | 21 | GAPDH | -0.22159 | 0.094753 |
| IAC91-1099 72 hpi (m.i) | 1 | 22 | GAPDH | -0.12392 | 0.192776 |
| IAC91-1099 72 hpi (m.i) | 1 | 23 | GAPDH | 0.062674 | 0.379716 |
| IAC91-1099 72 hpi (m.i) | 1 | 24 | GAPDH | 0.396688 | 0.714078 |
| IAC91-1099 72 hpi (m.i) | 1 | 25 | GAPDH | 0.957051 | 1.274789 |
| IAC91-1099 72 hpi (m.i) | 1 | 26 | GAPDH | 1.664092 | 1.982178 |
| IAC91-1099 72 hpi (m.i) | 1 | 27 | GAPDH | 2.335603 | 2.654036 |
| IAC91-1099 72 hpi (m.i) | 1 | 28 | GAPDH | 2.922573 | 3.241355 |
| IAC91-1099 72 hpi (m.i) | 1 | 29 | GAPDH | 3.461167 | 3.780297 |
| IAC91-1099 72 hpi (m.i) | 1 | 30 | GAPDH | 3.979502 | 4.29898 |
| IAC91-1099 72 hpi (m.i) | 1 | 31 | GAPDH | 4.433375 | 4.753201 |
| IAC91-1099 72 hpi (m.i) | 1 | 32 | GAPDH | 4.853151 | 5.173325 |
| IAC91-1099 72 hpi (m.i) | 1 | 33 | GAPDH | 5.218916 | 5.539438 |
| IAC91-1099 72 hpi (m.i) | 1 | 34 | GAPDH | 5.554097 | 5.874966 |
| IAC91-1099 72 hpi (m.i) | 1 | 35 | GAPDH | 5.839809 | 6.161026 |
| IAC91-1099 72 hpi (m.i) | 1 | 36 | GAPDH | 6.103299 | 6.424864 |
| IAC91-1099 72 hpi (m.i) | 1 | 37 | GAPDH | 6.346848 | 6.66876 |
| IAC91-1099 72 hpi (m.i) | 1 | 38 | GAPDH | 6.539371 | 6.861632 |
| IAC91-1099 72 hpi (m.i) | 1 | 39 | GAPDH | 6.729243 | 7.051852 |
| IAC91-1099 72 hpi (m.i) | 1 | 40 | GAPDH | 6.900296 | 7.223253 |
| IAC91-1099 72 hpi (m.i) | 2 | 1 | GAPDH | -0.31024 | -0.01016 |
| IAC91-1099 72 hpi (m.i) | 2 | 2 | GAPDH | -0.3052 | -0.00499 |
| IAC91-1099 72 hpi (m.i) | 2 | 3 | GAPDH | -0.30199 | -0.00164 |
| IAC91-1099 72 hpi (m.i) | 2 | 4 | GAPDH | -0.30099 | -0.00051 |
| IAC91-1099 72 hpi (m.i) | 2 | 5 | GAPDH | -0.29958 | 0.001039 |
| IAC91-1099 72 hpi (m.i) | 2 | 6 | GAPDH | -0.29939 | 0.001366 |
| IAC91-1099 72 hpi (m.i) | 2 | 7 | GAPDH | -0.29905 | 0.001838 |
| IAC91-1099 72 hpi (m.i) | 2 | 8 | GAPDH | -0.29902 | 0.002008 |
| IAC91-1099 72 hpi (m.i) | 2 | 9 | GAPDH | -0.30023 | 0.000931 |
| IAC91-1099 72 hpi (m.i) | 2 | 10 | GAPDH | -0.302 | -0.0007 |
| IAC91-1099 72 hpi (m.i) | 2 | 11 | GAPDH | -0.30396 | -0.00252 |
| IAC91-1099 72 hpi (m.i) | 2 | 12 | GAPDH | -0.3026 | -0.00102 |
| IAC91-1099 72 hpi (m.i) | 2 | 13 | GAPDH | -0.30414 | -0.00243 |
| IAC91-1099 72 hpi (m.i) | 2 | 14 | GAPDH | -0.30349 | -0.00164 |
| IAC91-1099 72 hpi (m.i) | 2 | 15 | GAPDH | -0.30328 | -0.0013 |
| IAC91-1099 72 hpi (m.i) | 2 | 16 | GAPDH | -0.30151 | 0.000604 |
| IAC91-1099 72 hpi (m.i) | 2 | 17 | GAPDH | -0.29827 | 0.003984 |
| IAC91-1099 72 hpi (m.i) | 2 | 18 | GAPDH | -0.29264 | 0.00975 |
| IAC91-1099 72 hpi (m.i) | 2 | 19 | GAPDH | -0.27868 | 0.023846 |
| IAC91-1099 72 hpi (m.i) | 2 | 20 | GAPDH | -0.25099 | 0.051676 |
| IAC91-1099 72 hpi (m.i) | 2 | 21 | GAPDH | -0.20072 | 0.102078 |
| IAC91-1099 72 hpi (m.i) | 2 | 22 | GAPDH | -0.10174 | 0.201193 |
| IAC91-1099 72 hpi (m.i) | 2 | 23 | GAPDH | 0.089325 | 0.392396 |
| IAC91-1099 72 hpi (m.i) | 2 | 24 | GAPDH | 0.43126 | 0.734467 |
| IAC91-1099 72 hpi (m.i) | 2 | 25 | GAPDH | 0.999423 | 1.302767 |
| IAC91-1099 72 hpi (m.i) | 2 | 26 | GAPDH | 1.689703 | 1.993183 |
| IAC91-1099 72 hpi (m.i) | 2 | 27 | GAPDH | 2.351913 | 2.655529 |
| IAC91-1099 72 hpi (m.i) | 2 | 28 | GAPDH | 2.913947 | 3.217699 |
| IAC91-1099 72 hpi (m.i) | 2 | 29 | GAPDH | 3.442291 | 3.746179 |
| IAC91-1099 72 hpi (m.i) | 2 | 30 | GAPDH | 3.949319 | 4.253344 |
| IAC91-1099 72 hpi (m.i) | 2 | 31 | GAPDH | 4.381937 | 4.686098 |
| IAC91-1099 72 hpi (m.i) | 2 | 32 | GAPDH | 4.796073 | 5.10037 |
| IAC91-1099 72 hpi (m.i) | 2 | 33 | GAPDH | 5.142963 | 5.447397 |
| IAC91-1099 72 hpi (m.i) | 2 | 34 | GAPDH | 5.462494 | 5.767064 |
| IAC91-1099 72 hpi (m.i) | 2 | 35 | GAPDH | 5.737475 | 6.042181 |
| IAC91-1099 72 hpi (m.i) | 2 | 36 | GAPDH | 5.986212 | 6.291054 |
| IAC91-1099 72 hpi (m.i) | 2 | 37 | GAPDH | 6.228358 | 6.533336 |
| IAC91-1099 72 hpi (m.i) | 2 | 38 | GAPDH | 6.410208 | 6.715322 |
| IAC91-1099 72 hpi (m.i) | 2 | 39 | GAPDH | 6.591515 | 6.896765 |
| IAC91-1099 72 hpi (m.i) | 2 | 40 | GAPDH | 6.756515 | 7.061901 |
| IAC91-1099 72 hpi (m.i) | 3 | 1 | GAPDH | -0.36591 | -0.00607 |
| IAC91-1099 72 hpi (m.i) | 3 | 2 | GAPDH | -0.36213 | -0.00228 |
| IAC91-1099 72 hpi (m.i) | 3 | 3 | GAPDH | -0.35972 | 0.000148 |
| IAC91-1099 72 hpi (m.i) | 3 | 4 | GAPDH | -0.35889 | 0.000996 |
| IAC91-1099 72 hpi (m.i) | 3 | 5 | GAPDH | -0.35793 | 0.001967 |
| IAC91-1099 72 hpi (m.i) | 3 | 6 | GAPDH | -0.35884 | 0.001074 |
| IAC91-1099 72 hpi (m.i) | 3 | 7 | GAPDH | -0.36028 | -0.00035 |
| IAC91-1099 72 hpi (m.i) | 3 | 8 | GAPDH | -0.36058 | -0.00064 |
| IAC91-1099 72 hpi (m.i) | 3 | 9 | GAPDH | -0.36067 | -0.00072 |
| IAC91-1099 72 hpi (m.i) | 3 | 10 | GAPDH | -0.36136 | -0.0014 |
| IAC91-1099 72 hpi (m.i) | 3 | 11 | GAPDH | -0.36139 | -0.00141 |
| IAC91-1099 72 hpi (m.i) | 3 | 12 | GAPDH | -0.36209 | -0.00209 |
| IAC91-1099 72 hpi (m.i) | 3 | 13 | GAPDH | -0.36113 | -0.00113 |
| IAC91-1099 72 hpi (m.i) | 3 | 14 | GAPDH | -0.3609 | -0.00088 |
| IAC91-1099 72 hpi (m.i) | 3 | 15 | GAPDH | -0.36016 | -0.00013 |
| IAC91-1099 72 hpi (m.i) | 3 | 16 | GAPDH | -0.35924 | 0.000804 |
| IAC91-1099 72 hpi (m.i) | 3 | 17 | GAPDH | -0.35629 | 0.003769 |
| IAC91-1099 72 hpi (m.i) | 3 | 18 | GAPDH | -0.35043 | 0.009643 |
| IAC91-1099 72 hpi (m.i) | 3 | 19 | GAPDH | -0.33569 | 0.024393 |
| IAC91-1099 72 hpi (m.i) | 3 | 20 | GAPDH | -0.3076 | 0.052501 |
| IAC91-1099 72 hpi (m.i) | 3 | 21 | GAPDH | -0.25473 | 0.105381 |
| IAC91-1099 72 hpi (m.i) | 3 | 22 | GAPDH | -0.15135 | 0.208778 |
| IAC91-1099 72 hpi (m.i) | 3 | 23 | GAPDH | 0.048009 | 0.40815 |
| IAC91-1099 72 hpi (m.i) | 3 | 24 | GAPDH | 0.408935 | 0.76909 |
| IAC91-1099 72 hpi (m.i) | 3 | 25 | GAPDH | 1.008438 | 1.368606 |
| IAC91-1099 72 hpi (m.i) | 3 | 26 | GAPDH | 1.740702 | 2.100884 |
| IAC91-1099 72 hpi (m.i) | 3 | 27 | GAPDH | 2.435567 | 2.795763 |
| IAC91-1099 72 hpi (m.i) | 3 | 28 | GAPDH | 3.031168 | 3.391377 |
| IAC91-1099 72 hpi (m.i) | 3 | 29 | GAPDH | 3.592808 | 3.953031 |
| IAC91-1099 72 hpi (m.i) | 3 | 30 | GAPDH | 4.120546 | 4.480783 |
| IAC91-1099 72 hpi (m.i) | 3 | 31 | GAPDH | 4.579411 | 4.939662 |
| IAC91-1099 72 hpi (m.i) | 3 | 32 | GAPDH | 5.013102 | 5.373366 |
| IAC91-1099 72 hpi (m.i) | 3 | 33 | GAPDH | 5.386952 | 5.74723 |
| IAC91-1099 72 hpi (m.i) | 3 | 34 | GAPDH | 5.721762 | 6.082053 |
| IAC91-1099 72 hpi (m.i) | 3 | 35 | GAPDH | 6.02123 | 6.381535 |
| IAC91-1099 72 hpi (m.i) | 3 | 36 | GAPDH | 6.288016 | 6.648335 |
| IAC91-1099 72 hpi (m.i) | 3 | 37 | GAPDH | 6.542732 | 6.903064 |
| IAC91-1099 72 hpi (m.i) | 3 | 38 | GAPDH | 6.734414 | 7.09476 |
| IAC91-1099 72 hpi (m.i) | 3 | 39 | GAPDH | 6.929109 | 7.289469 |
| IAC91-1099 72 hpi (m.i) | 3 | 40 | GAPDH | 7.099476 | 7.459849 |
| IAC91-1099 72 hpi (s.i) | 1 | 1 | GAPDH | -0.31441 | -0.01655 |
| IAC91-1099 72 hpi (s.i) | 1 | 2 | GAPDH | -0.30592 | -0.00765 |
| IAC91-1099 72 hpi (s.i) | 1 | 3 | GAPDH | -0.30311 | -0.00443 |
| IAC91-1099 72 hpi (s.i) | 1 | 4 | GAPDH | -0.30039 | -0.00129 |
| IAC91-1099 72 hpi (s.i) | 1 | 5 | GAPDH | -0.29609 | 0.003428 |
| IAC91-1099 72 hpi (s.i) | 1 | 6 | GAPDH | -0.29664 | 0.003297 |
| IAC91-1099 72 hpi (s.i) | 1 | 7 | GAPDH | -0.29705 | 0.003309 |
| IAC91-1099 72 hpi (s.i) | 1 | 8 | GAPDH | -0.30457 | -0.0038 |
| IAC91-1099 72 hpi (s.i) | 1 | 9 | GAPDH | -0.29942 | 0.001767 |
| IAC91-1099 72 hpi (s.i) | 1 | 10 | GAPDH | -0.30161 | -8.2E-06 |
| IAC91-1099 72 hpi (s.i) | 1 | 11 | GAPDH | -0.30253 | -0.00051 |
| IAC91-1099 72 hpi (s.i) | 1 | 12 | GAPDH | -0.30376 | -0.00132 |
| IAC91-1099 72 hpi (s.i) | 1 | 13 | GAPDH | -0.30287 | -1.8E-05 |
| IAC91-1099 72 hpi (s.i) | 1 | 14 | GAPDH | -0.3037 | -0.00043 |
| IAC91-1099 72 hpi (s.i) | 1 | 15 | GAPDH | -0.3043 | -0.00061 |
| IAC91-1099 72 hpi (s.i) | 1 | 16 | GAPDH | -0.30348 | 0.000617 |
| IAC91-1099 72 hpi (s.i) | 1 | 17 | GAPDH | -0.30312 | 0.0014 |
| IAC91-1099 72 hpi (s.i) | 1 | 18 | GAPDH | -0.29292 | 0.012012 |
| IAC91-1099 72 hpi (s.i) | 1 | 19 | GAPDH | -0.28264 | 0.02271 |
| IAC91-1099 72 hpi (s.i) | 1 | 20 | GAPDH | -0.25618 | 0.049585 |
| IAC91-1099 72 hpi (s.i) | 1 | 21 | GAPDH | -0.20824 | 0.097942 |
| IAC91-1099 72 hpi (s.i) | 1 | 22 | GAPDH | -0.11263 | 0.193966 |
| IAC91-1099 72 hpi (s.i) | 1 | 23 | GAPDH | 0.068927 | 0.375943 |
| IAC91-1099 72 hpi (s.i) | 1 | 24 | GAPDH | 0.396773 | 0.704205 |
| IAC91-1099 72 hpi (s.i) | 1 | 25 | GAPDH | 0.958245 | 1.266092 |
| IAC91-1099 72 hpi (s.i) | 1 | 26 | GAPDH | 1.688635 | 1.996899 |
| IAC91-1099 72 hpi (s.i) | 1 | 27 | GAPDH | 2.40076 | 2.70944 |
| IAC91-1099 72 hpi (s.i) | 1 | 28 | GAPDH | 3.004832 | 3.313928 |
| IAC91-1099 72 hpi (s.i) | 1 | 29 | GAPDH | 3.566491 | 3.876004 |
| IAC91-1099 72 hpi (s.i) | 1 | 30 | GAPDH | 4.110207 | 4.420136 |
| IAC91-1099 72 hpi (s.i) | 1 | 31 | GAPDH | 4.58197 | 4.892316 |
| IAC91-1099 72 hpi (s.i) | 1 | 32 | GAPDH | 5.025374 | 5.336136 |
| IAC91-1099 72 hpi (s.i) | 1 | 33 | GAPDH | 5.402689 | 5.713867 |
| IAC91-1099 72 hpi (s.i) | 1 | 34 | GAPDH | 5.744541 | 6.056136 |
| IAC91-1099 72 hpi (s.i) | 1 | 35 | GAPDH | 6.0741 | 6.386111 |
| IAC91-1099 72 hpi (s.i) | 1 | 36 | GAPDH | 6.324719 | 6.637146 |
| IAC91-1099 72 hpi (s.i) | 1 | 37 | GAPDH | 6.561076 | 6.873919 |
| IAC91-1099 72 hpi (s.i) | 1 | 38 | GAPDH | 6.784311 | 7.097571 |
| IAC91-1099 72 hpi (s.i) | 1 | 39 | GAPDH | 6.96919 | 7.282866 |
| IAC91-1099 72 hpi (s.i) | 1 | 40 | GAPDH | 7.14603 | 7.460123 |
| IAC91-1099 72 hpi (s.i) | 2 | 1 | GAPDH | -0.35502 | -0.01128 |
| IAC91-1099 72 hpi (s.i) | 2 | 2 | GAPDH | -0.34643 | -0.00302 |
| IAC91-1099 72 hpi (s.i) | 2 | 3 | GAPDH | -0.34708 | -0.00399 |
| IAC91-1099 72 hpi (s.i) | 2 | 4 | GAPDH | -0.34232 | 0.00044 |
| IAC91-1099 72 hpi (s.i) | 2 | 5 | GAPDH | -0.33821 | 0.004221 |
| IAC91-1099 72 hpi (s.i) | 2 | 6 | GAPDH | -0.33644 | 0.005655 |
| IAC91-1099 72 hpi (s.i) | 2 | 7 | GAPDH | -0.33721 | 0.004566 |
| IAC91-1099 72 hpi (s.i) | 2 | 8 | GAPDH | -0.34709 | -0.00565 |
| IAC91-1099 72 hpi (s.i) | 2 | 9 | GAPDH | -0.33831 | 0.002802 |
| IAC91-1099 72 hpi (s.i) | 2 | 10 | GAPDH | -0.34173 | -0.00094 |
| IAC91-1099 72 hpi (s.i) | 2 | 11 | GAPDH | -0.34193 | -0.00147 |
| IAC91-1099 72 hpi (s.i) | 2 | 12 | GAPDH | -0.34228 | -0.00215 |
| IAC91-1099 72 hpi (s.i) | 2 | 13 | GAPDH | -0.34373 | -0.00392 |
| IAC91-1099 72 hpi (s.i) | 2 | 14 | GAPDH | -0.34408 | -0.0046 |
| IAC91-1099 72 hpi (s.i) | 2 | 15 | GAPDH | -0.3437 | -0.00455 |
| IAC91-1099 72 hpi (s.i) | 2 | 16 | GAPDH | -0.34058 | -0.00176 |
| IAC91-1099 72 hpi (s.i) | 2 | 17 | GAPDH | -0.34052 | -0.00203 |
| IAC91-1099 72 hpi (s.i) | 2 | 18 | GAPDH | -0.32478 | 0.013388 |
| IAC91-1099 72 hpi (s.i) | 2 | 19 | GAPDH | -0.32195 | 0.015886 |
| IAC91-1099 72 hpi (s.i) | 2 | 20 | GAPDH | -0.29179 | 0.045721 |
| IAC91-1099 72 hpi (s.i) | 2 | 21 | GAPDH | -0.25063 | 0.086551 |
| IAC91-1099 72 hpi (s.i) | 2 | 22 | GAPDH | -0.15649 | 0.180367 |
| IAC91-1099 72 hpi (s.i) | 2 | 23 | GAPDH | 0.013703 | 0.350229 |
| IAC91-1099 72 hpi (s.i) | 2 | 24 | GAPDH | 0.330911 | 0.667109 |
| IAC91-1099 72 hpi (s.i) | 2 | 25 | GAPDH | 0.876092 | 1.211963 |
| IAC91-1099 72 hpi (s.i) | 2 | 26 | GAPDH | 1.616323 | 1.951866 |
| IAC91-1099 72 hpi (s.i) | 2 | 27 | GAPDH | 2.3431 | 2.678315 |
| IAC91-1099 72 hpi (s.i) | 2 | 28 | GAPDH | 2.967792 | 3.302679 |
| IAC91-1099 72 hpi (s.i) | 2 | 29 | GAPDH | 3.541152 | 3.875711 |
| IAC91-1099 72 hpi (s.i) | 2 | 30 | GAPDH | 4.094634 | 4.428865 |
| IAC91-1099 72 hpi (s.i) | 2 | 31 | GAPDH | 4.587123 | 4.921027 |
| IAC91-1099 72 hpi (s.i) | 2 | 32 | GAPDH | 5.040104 | 5.37368 |
| IAC91-1099 72 hpi (s.i) | 2 | 33 | GAPDH | 5.422972 | 5.75622 |
| IAC91-1099 72 hpi (s.i) | 2 | 34 | GAPDH | 5.795666 | 6.128586 |
| IAC91-1099 72 hpi (s.i) | 2 | 35 | GAPDH | 6.109104 | 6.441696 |
| IAC91-1099 72 hpi (s.i) | 2 | 36 | GAPDH | 6.383104 | 6.715368 |
| IAC91-1099 72 hpi (s.i) | 2 | 37 | GAPDH | 6.643781 | 6.975718 |
| IAC91-1099 72 hpi (s.i) | 2 | 38 | GAPDH | 6.857714 | 7.189322 |
| IAC91-1099 72 hpi (s.i) | 2 | 39 | GAPDH | 7.061962 | 7.393242 |
| IAC91-1099 72 hpi (s.i) | 2 | 40 | GAPDH | 7.25411 | 7.585063 |
| IAC91-1099 72 hpi (s.i) | 3 | 1 | GAPDH | -0.291 | -0.00845 |
| IAC91-1099 72 hpi (s.i) | 3 | 2 | GAPDH | -0.2843 | -0.00131 |
| IAC91-1099 72 hpi (s.i) | 3 | 3 | GAPDH | -0.27905 | 0.004371 |
| IAC91-1099 72 hpi (s.i) | 3 | 4 | GAPDH | -0.28247 | 0.001392 |
| IAC91-1099 72 hpi (s.i) | 3 | 5 | GAPDH | -0.28554 | -0.00124 |
| IAC91-1099 72 hpi (s.i) | 3 | 6 | GAPDH | -0.28622 | -0.00148 |
| IAC91-1099 72 hpi (s.i) | 3 | 7 | GAPDH | -0.28859 | -0.00341 |
| IAC91-1099 72 hpi (s.i) | 3 | 8 | GAPDH | -0.27886 | 0.006755 |
| IAC91-1099 72 hpi (s.i) | 3 | 9 | GAPDH | -0.28848 | -0.00243 |
| IAC91-1099 72 hpi (s.i) | 3 | 10 | GAPDH | -0.28949 | -0.003 |
| IAC91-1099 72 hpi (s.i) | 3 | 11 | GAPDH | -0.29122 | -0.00429 |
| IAC91-1099 72 hpi (s.i) | 3 | 12 | GAPDH | -0.28868 | -0.00132 |
| IAC91-1099 72 hpi (s.i) | 3 | 13 | GAPDH | -0.28888 | -0.00108 |
| IAC91-1099 72 hpi (s.i) | 3 | 14 | GAPDH | -0.28886 | -0.00062 |
| IAC91-1099 72 hpi (s.i) | 3 | 15 | GAPDH | -0.28723 | 0.001447 |
| IAC91-1099 72 hpi (s.i) | 3 | 16 | GAPDH | -0.28786 | 0.001253 |
| IAC91-1099 72 hpi (s.i) | 3 | 17 | GAPDH | -0.2859 | 0.003651 |
| IAC91-1099 72 hpi (s.i) | 3 | 18 | GAPDH | -0.2819 | 0.008094 |
| IAC91-1099 72 hpi (s.i) | 3 | 19 | GAPDH | -0.26989 | 0.020538 |
| IAC91-1099 72 hpi (s.i) | 3 | 20 | GAPDH | -0.25223 | 0.038634 |
| IAC91-1099 72 hpi (s.i) | 3 | 21 | GAPDH | -0.20753 | 0.083773 |
| IAC91-1099 72 hpi (s.i) | 3 | 22 | GAPDH | -0.12888 | 0.162857 |
| IAC91-1099 72 hpi (s.i) | 3 | 23 | GAPDH | 0.031633 | 0.323811 |
| IAC91-1099 72 hpi (s.i) | 3 | 24 | GAPDH | 0.314266 | 0.606882 |
| IAC91-1099 72 hpi (s.i) | 3 | 25 | GAPDH | 0.811382 | 1.104435 |
| IAC91-1099 72 hpi (s.i) | 3 | 26 | GAPDH | 1.50372 | 1.79721 |
| IAC91-1099 72 hpi (s.i) | 3 | 27 | GAPDH | 2.198195 | 2.492122 |
| IAC91-1099 72 hpi (s.i) | 3 | 28 | GAPDH | 2.7994 | 3.093766 |
| IAC91-1099 72 hpi (s.i) | 3 | 29 | GAPDH | 3.342899 | 3.637702 |
| IAC91-1099 72 hpi (s.i) | 3 | 30 | GAPDH | 3.866529 | 4.16177 |
| IAC91-1099 72 hpi (s.i) | 3 | 31 | GAPDH | 4.327819 | 4.623497 |
| IAC91-1099 72 hpi (s.i) | 3 | 32 | GAPDH | 4.747705 | 5.043821 |
| IAC91-1099 72 hpi (s.i) | 3 | 33 | GAPDH | 5.110262 | 5.406816 |
| IAC91-1099 72 hpi (s.i) | 3 | 34 | GAPDH | 5.459841 | 5.756832 |
| IAC91-1099 72 hpi (s.i) | 3 | 35 | GAPDH | 5.741461 | 6.03889 |
| IAC91-1099 72 hpi (s.i) | 3 | 36 | GAPDH | 6.007911 | 6.305777 |
| IAC91-1099 72 hpi (s.i) | 3 | 37 | GAPDH | 6.258675 | 6.556978 |
| IAC91-1099 72 hpi (s.i) | 3 | 38 | GAPDH | 6.447201 | 6.745942 |
| IAC91-1099 72 hpi (s.i) | 3 | 39 | GAPDH | 6.632194 | 6.931372 |
| IAC91-1099 72 hpi (s.i) | 3 | 40 | GAPDH | 6.814332 | 7.113949 |
| IACSP95-5000 24 hpi (m.i) | 1 | 1 | GAPDH | -0.29883 | -0.00947 |
| IACSP95-5000 24 hpi (m.i) | 1 | 2 | GAPDH | -0.29268 | -0.0031 |
| IACSP95-5000 24 hpi (m.i) | 1 | 3 | GAPDH | -0.28979 | 1.75E-05 |
| IACSP95-5000 24 hpi (m.i) | 1 | 4 | GAPDH | -0.28697 | 0.003053 |
| IACSP95-5000 24 hpi (m.i) | 1 | 5 | GAPDH | -0.29098 | -0.00073 |
| IACSP95-5000 24 hpi (m.i) | 1 | 6 | GAPDH | -0.29225 | -0.00177 |
| IACSP95-5000 24 hpi (m.i) | 1 | 7 | GAPDH | -0.2925 | -0.00181 |
| IACSP95-5000 24 hpi (m.i) | 1 | 8 | GAPDH | -0.28759 | 0.003327 |
| IACSP95-5000 24 hpi (m.i) | 1 | 9 | GAPDH | -0.28891 | 0.002226 |
| IACSP95-5000 24 hpi (m.i) | 1 | 10 | GAPDH | -0.2933 | -0.00194 |
| IACSP95-5000 24 hpi (m.i) | 1 | 11 | GAPDH | -0.29586 | -0.00428 |
| IACSP95-5000 24 hpi (m.i) | 1 | 12 | GAPDH | -0.29457 | -0.00277 |
| IACSP95-5000 24 hpi (m.i) | 1 | 13 | GAPDH | -0.28951 | 0.002514 |
| IACSP95-5000 24 hpi (m.i) | 1 | 14 | GAPDH | -0.29109 | 0.001156 |
| IACSP95-5000 24 hpi (m.i) | 1 | 15 | GAPDH | -0.2933 | -0.00084 |
| IACSP95-5000 24 hpi (m.i) | 1 | 16 | GAPDH | -0.29273 | -4.3E-05 |
| IACSP95-5000 24 hpi (m.i) | 1 | 17 | GAPDH | -0.29102 | 0.001889 |
| IACSP95-5000 24 hpi (m.i) | 1 | 18 | GAPDH | -0.28615 | 0.00698 |
| IACSP95-5000 24 hpi (m.i) | 1 | 19 | GAPDH | -0.27226 | 0.021093 |
| IACSP95-5000 24 hpi (m.i) | 1 | 20 | GAPDH | -0.25314 | 0.04044 |
| IACSP95-5000 24 hpi (m.i) | 1 | 21 | GAPDH | -0.21318 | 0.080615 |
| IACSP95-5000 24 hpi (m.i) | 1 | 22 | GAPDH | -0.14104 | 0.152979 |
| IACSP95-5000 24 hpi (m.i) | 1 | 23 | GAPDH | 0.008581 | 0.302825 |
| IACSP95-5000 24 hpi (m.i) | 1 | 24 | GAPDH | 0.284769 | 0.579235 |
| IACSP95-5000 24 hpi (m.i) | 1 | 25 | GAPDH | 0.762102 | 1.056789 |
| IACSP95-5000 24 hpi (m.i) | 1 | 26 | GAPDH | 1.454616 | 1.749525 |
| IACSP95-5000 24 hpi (m.i) | 1 | 27 | GAPDH | 2.163481 | 2.458612 |
| IACSP95-5000 24 hpi (m.i) | 1 | 28 | GAPDH | 2.789255 | 3.084608 |
| IACSP95-5000 24 hpi (m.i) | 1 | 29 | GAPDH | 3.335232 | 3.630806 |
| IACSP95-5000 24 hpi (m.i) | 1 | 30 | GAPDH | 3.865083 | 4.16088 |
| IACSP95-5000 24 hpi (m.i) | 1 | 31 | GAPDH | 4.336895 | 4.632914 |
| IACSP95-5000 24 hpi (m.i) | 1 | 32 | GAPDH | 4.768048 | 5.064288 |
| IACSP95-5000 24 hpi (m.i) | 1 | 33 | GAPDH | 5.132091 | 5.428553 |
| IACSP95-5000 24 hpi (m.i) | 1 | 34 | GAPDH | 5.476429 | 5.773113 |
| IACSP95-5000 24 hpi (m.i) | 1 | 35 | GAPDH | 5.770898 | 6.067804 |
| IACSP95-5000 24 hpi (m.i) | 1 | 36 | GAPDH | 6.033861 | 6.330988 |
| IACSP95-5000 24 hpi (m.i) | 1 | 37 | GAPDH | 6.297476 | 6.594826 |
| IACSP95-5000 24 hpi (m.i) | 1 | 38 | GAPDH | 6.47879 | 6.776361 |
| IACSP95-5000 24 hpi (m.i) | 1 | 39 | GAPDH | 6.695136 | 6.992929 |
| IACSP95-5000 24 hpi (m.i) | 1 | 40 | GAPDH | 6.861334 | 7.159349 |
| IACSP95-5000 24 hpi (m.i) | 2 | 1 | GAPDH | -0.2774 | -0.00531 |
| IACSP95-5000 24 hpi (m.i) | 2 | 2 | GAPDH | -0.2761 | -0.00369 |
| IACSP95-5000 24 hpi (m.i) | 2 | 3 | GAPDH | -0.27362 | -0.00089 |
| IACSP95-5000 24 hpi (m.i) | 2 | 4 | GAPDH | -0.2737 | -0.00066 |
| IACSP95-5000 24 hpi (m.i) | 2 | 5 | GAPDH | -0.27168 | 0.001672 |
| IACSP95-5000 24 hpi (m.i) | 2 | 6 | GAPDH | -0.27139 | 0.002269 |
| IACSP95-5000 24 hpi (m.i) | 2 | 7 | GAPDH | -0.27245 | 0.001526 |
| IACSP95-5000 24 hpi (m.i) | 2 | 8 | GAPDH | -0.27418 | 0.000112 |
| IACSP95-5000 24 hpi (m.i) | 2 | 9 | GAPDH | -0.27529 | -0.00068 |
| IACSP95-5000 24 hpi (m.i) | 2 | 10 | GAPDH | -0.27693 | -0.00201 |
| IACSP95-5000 24 hpi (m.i) | 2 | 11 | GAPDH | -0.27631 | -0.00108 |
| IACSP95-5000 24 hpi (m.i) | 2 | 12 | GAPDH | -0.27542 | 0.000124 |
| IACSP95-5000 24 hpi (m.i) | 2 | 13 | GAPDH | -0.2774 | -0.00154 |
| IACSP95-5000 24 hpi (m.i) | 2 | 14 | GAPDH | -0.27869 | -0.00252 |
| IACSP95-5000 24 hpi (m.i) | 2 | 15 | GAPDH | -0.27692 | -0.00043 |
| IACSP95-5000 24 hpi (m.i) | 2 | 16 | GAPDH | -0.27699 | -0.0002 |
| IACSP95-5000 24 hpi (m.i) | 2 | 17 | GAPDH | -0.27279 | 0.004319 |
| IACSP95-5000 24 hpi (m.i) | 2 | 18 | GAPDH | -0.26819 | 0.009232 |
| IACSP95-5000 24 hpi (m.i) | 2 | 19 | GAPDH | -0.25754 | 0.020194 |
| IACSP95-5000 24 hpi (m.i) | 2 | 20 | GAPDH | -0.23506 | 0.042987 |
| IACSP95-5000 24 hpi (m.i) | 2 | 21 | GAPDH | -0.19207 | 0.086296 |
| IACSP95-5000 24 hpi (m.i) | 2 | 22 | GAPDH | -0.11129 | 0.16739 |
| IACSP95-5000 24 hpi (m.i) | 2 | 23 | GAPDH | 0.044462 | 0.323454 |
| IACSP95-5000 24 hpi (m.i) | 2 | 24 | GAPDH | 0.331581 | 0.610887 |
| IACSP95-5000 24 hpi (m.i) | 2 | 25 | GAPDH | 0.827414 | 1.107033 |
| IACSP95-5000 24 hpi (m.i) | 2 | 26 | GAPDH | 1.53339 | 1.813323 |
| IACSP95-5000 24 hpi (m.i) | 2 | 27 | GAPDH | 2.239722 | 2.519969 |
| IACSP95-5000 24 hpi (m.i) | 2 | 28 | GAPDH | 2.854131 | 3.134691 |
| IACSP95-5000 24 hpi (m.i) | 2 | 29 | GAPDH | 3.3936 | 3.674474 |
| IACSP95-5000 24 hpi (m.i) | 2 | 30 | GAPDH | 3.915306 | 4.196493 |
| IACSP95-5000 24 hpi (m.i) | 2 | 31 | GAPDH | 4.369863 | 4.651363 |
| IACSP95-5000 24 hpi (m.i) | 2 | 32 | GAPDH | 4.795269 | 5.077084 |
| IACSP95-5000 24 hpi (m.i) | 2 | 33 | GAPDH | 5.15464 | 5.436768 |
| IACSP95-5000 24 hpi (m.i) | 2 | 34 | GAPDH | 5.495911 | 5.778353 |
| IACSP95-5000 24 hpi (m.i) | 2 | 35 | GAPDH | 5.781857 | 6.064612 |
| IACSP95-5000 24 hpi (m.i) | 2 | 36 | GAPDH | 6.043608 | 6.326676 |
| IACSP95-5000 24 hpi (m.i) | 2 | 37 | GAPDH | 6.28109 | 6.564472 |
| IACSP95-5000 24 hpi (m.i) | 2 | 38 | GAPDH | 6.508387 | 6.792082 |
| IACSP95-5000 24 hpi (m.i) | 2 | 39 | GAPDH | 6.654103 | 6.938112 |
| IACSP95-5000 24 hpi (m.i) | 2 | 40 | GAPDH | 6.827417 | 7.11174 |
| IACSP95-5000 24 hpi (m.i) | 3 | 1 | GAPDH | -0.31878 | -0.00601 |
| IACSP95-5000 24 hpi (m.i) | 3 | 2 | GAPDH | -0.31097 | 0.001539 |
| IACSP95-5000 24 hpi (m.i) | 3 | 3 | GAPDH | -0.31113 | 0.001107 |
| IACSP95-5000 24 hpi (m.i) | 3 | 4 | GAPDH | -0.31089 | 0.001082 |
| IACSP95-5000 24 hpi (m.i) | 3 | 5 | GAPDH | -0.31128 | 0.000431 |
| IACSP95-5000 24 hpi (m.i) | 3 | 6 | GAPDH | -0.31071 | 0.000741 |
| IACSP95-5000 24 hpi (m.i) | 3 | 7 | GAPDH | -0.3106 | 0.000582 |
| IACSP95-5000 24 hpi (m.i) | 3 | 8 | GAPDH | -0.31042 | 0.0005 |
| IACSP95-5000 24 hpi (m.i) | 3 | 9 | GAPDH | -0.30992 | 0.000736 |
| IACSP95-5000 24 hpi (m.i) | 3 | 10 | GAPDH | -0.30981 | 0.000581 |
| IACSP95-5000 24 hpi (m.i) | 3 | 11 | GAPDH | -0.31288 | -0.00275 |
| IACSP95-5000 24 hpi (m.i) | 3 | 12 | GAPDH | -0.31212 | -0.00225 |
| IACSP95-5000 24 hpi (m.i) | 3 | 13 | GAPDH | -0.31248 | -0.00287 |
| IACSP95-5000 24 hpi (m.i) | 3 | 14 | GAPDH | -0.31323 | -0.00389 |
| IACSP95-5000 24 hpi (m.i) | 3 | 15 | GAPDH | -0.31168 | -0.0026 |
| IACSP95-5000 24 hpi (m.i) | 3 | 16 | GAPDH | -0.30924 | -0.00042 |
| IACSP95-5000 24 hpi (m.i) | 3 | 17 | GAPDH | -0.30614 | 0.002411 |
| IACSP95-5000 24 hpi (m.i) | 3 | 18 | GAPDH | -0.30167 | 0.006619 |
| IACSP95-5000 24 hpi (m.i) | 3 | 19 | GAPDH | -0.29273 | 0.015288 |
| IACSP95-5000 24 hpi (m.i) | 3 | 20 | GAPDH | -0.26812 | 0.039644 |
| IACSP95-5000 24 hpi (m.i) | 3 | 21 | GAPDH | -0.22858 | 0.078915 |
| IACSP95-5000 24 hpi (m.i) | 3 | 22 | GAPDH | -0.14488 | 0.162355 |
| IACSP95-5000 24 hpi (m.i) | 3 | 23 | GAPDH | 0.012329 | 0.319298 |
| IACSP95-5000 24 hpi (m.i) | 3 | 24 | GAPDH | 0.303021 | 0.609725 |
| IACSP95-5000 24 hpi (m.i) | 3 | 25 | GAPDH | 0.811849 | 1.11829 |
| IACSP95-5000 24 hpi (m.i) | 3 | 26 | GAPDH | 1.529919 | 1.836096 |
| IACSP95-5000 24 hpi (m.i) | 3 | 27 | GAPDH | 2.250786 | 2.5567 |
| IACSP95-5000 24 hpi (m.i) | 3 | 28 | GAPDH | 2.867004 | 3.172654 |
| IACSP95-5000 24 hpi (m.i) | 3 | 29 | GAPDH | 3.419492 | 3.724878 |
| IACSP95-5000 24 hpi (m.i) | 3 | 30 | GAPDH | 3.949727 | 4.25485 |
| IACSP95-5000 24 hpi (m.i) | 3 | 31 | GAPDH | 4.410306 | 4.715165 |
| IACSP95-5000 24 hpi (m.i) | 3 | 32 | GAPDH | 4.844888 | 5.149484 |
| IACSP95-5000 24 hpi (m.i) | 3 | 33 | GAPDH | 5.209373 | 5.513705 |
| IACSP95-5000 24 hpi (m.i) | 3 | 34 | GAPDH | 5.557551 | 5.86162 |
| IACSP95-5000 24 hpi (m.i) | 3 | 35 | GAPDH | 5.84648 | 6.150285 |
| IACSP95-5000 24 hpi (m.i) | 3 | 36 | GAPDH | 6.109925 | 6.413466 |
| IACSP95-5000 24 hpi (m.i) | 3 | 37 | GAPDH | 6.35572 | 6.658998 |
| IACSP95-5000 24 hpi (m.i) | 3 | 38 | GAPDH | 6.518756 | 6.82177 |
| IACSP95-5000 24 hpi (m.i) | 3 | 39 | GAPDH | 6.738569 | 7.04132 |
| IACSP95-5000 24 hpi (m.i) | 3 | 40 | GAPDH | 6.909249 | 7.211736 |
| IACSP95-5000 24 hpi (s.i) | 1 | 1 | GAPDH | -0.25195 | -0.00319 |
| IACSP95-5000 24 hpi (s.i) | 1 | 2 | GAPDH | -0.24757 | 0.000738 |
| IACSP95-5000 24 hpi (s.i) | 1 | 3 | GAPDH | -0.24529 | 0.002577 |
| IACSP95-5000 24 hpi (s.i) | 1 | 4 | GAPDH | -0.24648 | 0.000947 |
| IACSP95-5000 24 hpi (s.i) | 1 | 5 | GAPDH | -0.24591 | 0.001075 |
| IACSP95-5000 24 hpi (s.i) | 1 | 6 | GAPDH | -0.24705 | -0.00052 |
| IACSP95-5000 24 hpi (s.i) | 1 | 7 | GAPDH | -0.24626 | -0.00017 |
| IACSP95-5000 24 hpi (s.i) | 1 | 8 | GAPDH | -0.24692 | -0.00127 |
| IACSP95-5000 24 hpi (s.i) | 1 | 9 | GAPDH | -0.24588 | -0.00068 |
| IACSP95-5000 24 hpi (s.i) | 1 | 10 | GAPDH | -0.24778 | -0.00301 |
| IACSP95-5000 24 hpi (s.i) | 1 | 11 | GAPDH | -0.24588 | -0.00155 |
| IACSP95-5000 24 hpi (s.i) | 1 | 12 | GAPDH | -0.24345 | 0.000423 |
| IACSP95-5000 24 hpi (s.i) | 1 | 13 | GAPDH | -0.24412 | -0.00068 |
| IACSP95-5000 24 hpi (s.i) | 1 | 14 | GAPDH | -0.24541 | -0.00242 |
| IACSP95-5000 24 hpi (s.i) | 1 | 15 | GAPDH | -0.24299 | -0.00045 |
| IACSP95-5000 24 hpi (s.i) | 1 | 16 | GAPDH | -0.24 | 0.002108 |
| IACSP95-5000 24 hpi (s.i) | 1 | 17 | GAPDH | -0.23804 | 0.003618 |
| IACSP95-5000 24 hpi (s.i) | 1 | 18 | GAPDH | -0.23155 | 0.009664 |
| IACSP95-5000 24 hpi (s.i) | 1 | 19 | GAPDH | -0.22132 | 0.019453 |
| IACSP95-5000 24 hpi (s.i) | 1 | 20 | GAPDH | -0.19628 | 0.04405 |
| IACSP95-5000 24 hpi (s.i) | 1 | 21 | GAPDH | -0.15412 | 0.085772 |
| IACSP95-5000 24 hpi (s.i) | 1 | 22 | GAPDH | -0.06985 | 0.169596 |
| IACSP95-5000 24 hpi (s.i) | 1 | 23 | GAPDH | 0.0924 | 0.331402 |
| IACSP95-5000 24 hpi (s.i) | 1 | 24 | GAPDH | 0.390816 | 0.629374 |
| IACSP95-5000 24 hpi (s.i) | 1 | 25 | GAPDH | 0.91451 | 1.152624 |
| IACSP95-5000 24 hpi (s.i) | 1 | 26 | GAPDH | 1.662198 | 1.899869 |
| IACSP95-5000 24 hpi (s.i) | 1 | 27 | GAPDH | 2.407587 | 2.644815 |
| IACSP95-5000 24 hpi (s.i) | 1 | 28 | GAPDH | 3.049326 | 3.286111 |
| IACSP95-5000 24 hpi (s.i) | 1 | 29 | GAPDH | 3.615475 | 3.851816 |
| IACSP95-5000 24 hpi (s.i) | 1 | 30 | GAPDH | 4.155085 | 4.390983 |
| IACSP95-5000 24 hpi (s.i) | 1 | 31 | GAPDH | 4.628296 | 4.86375 |
| IACSP95-5000 24 hpi (s.i) | 1 | 32 | GAPDH | 5.070238 | 5.30525 |
| IACSP95-5000 24 hpi (s.i) | 1 | 33 | GAPDH | 5.447761 | 5.682329 |
| IACSP95-5000 24 hpi (s.i) | 1 | 34 | GAPDH | 5.79802 | 6.032145 |
| IACSP95-5000 24 hpi (s.i) | 1 | 35 | GAPDH | 6.104851 | 6.338532 |
| IACSP95-5000 24 hpi (s.i) | 1 | 36 | GAPDH | 6.363269 | 6.596507 |
| IACSP95-5000 24 hpi (s.i) | 1 | 37 | GAPDH | 6.624 | 6.856794 |
| IACSP95-5000 24 hpi (s.i) | 1 | 38 | GAPDH | 6.841178 | 7.073529 |
| IACSP95-5000 24 hpi (s.i) | 1 | 39 | GAPDH | 7.019175 | 7.251082 |
| IACSP95-5000 24 hpi (s.i) | 1 | 40 | GAPDH | 7.198298 | 7.429763 |
| IACSP95-5000 24 hpi (s.i) | 2 | 1 | GAPDH | -0.28743 | -0.00591 |
| IACSP95-5000 24 hpi (s.i) | 2 | 2 | GAPDH | -0.28101 | -0.00031 |
| IACSP95-5000 24 hpi (s.i) | 2 | 3 | GAPDH | -0.28039 | -0.00051 |
| IACSP95-5000 24 hpi (s.i) | 2 | 4 | GAPDH | -0.27711 | 0.001961 |
| IACSP95-5000 24 hpi (s.i) | 2 | 5 | GAPDH | -0.27738 | 0.000878 |
| IACSP95-5000 24 hpi (s.i) | 2 | 6 | GAPDH | -0.27768 | -0.00023 |
| IACSP95-5000 24 hpi (s.i) | 2 | 7 | GAPDH | -0.27778 | -0.00115 |
| IACSP95-5000 24 hpi (s.i) | 2 | 8 | GAPDH | -0.27653 | -0.00071 |
| IACSP95-5000 24 hpi (s.i) | 2 | 9 | GAPDH | -0.27472 | 0.000287 |
| IACSP95-5000 24 hpi (s.i) | 2 | 10 | GAPDH | -0.27289 | 0.001299 |
| IACSP95-5000 24 hpi (s.i) | 2 | 11 | GAPDH | -0.27432 | -0.00094 |
| IACSP95-5000 24 hpi (s.i) | 2 | 12 | GAPDH | -0.27324 | -0.00068 |
| IACSP95-5000 24 hpi (s.i) | 2 | 13 | GAPDH | -0.27412 | -0.00237 |
| IACSP95-5000 24 hpi (s.i) | 2 | 14 | GAPDH | -0.2709 | 3.28E-05 |
| IACSP95-5000 24 hpi (s.i) | 2 | 15 | GAPDH | -0.27083 | -0.00071 |
| IACSP95-5000 24 hpi (s.i) | 2 | 16 | GAPDH | -0.26939 | -7.8E-05 |
| IACSP95-5000 24 hpi (s.i) | 2 | 17 | GAPDH | -0.26558 | 0.002912 |
| IACSP95-5000 24 hpi (s.i) | 2 | 18 | GAPDH | -0.26188 | 0.005806 |
| IACSP95-5000 24 hpi (s.i) | 2 | 19 | GAPDH | -0.24916 | 0.017706 |
| IACSP95-5000 24 hpi (s.i) | 2 | 20 | GAPDH | -0.22292 | 0.043129 |
| IACSP95-5000 24 hpi (s.i) | 2 | 21 | GAPDH | -0.17962 | 0.085619 |
| IACSP95-5000 24 hpi (s.i) | 2 | 22 | GAPDH | -0.0925 | 0.171923 |
| IACSP95-5000 24 hpi (s.i) | 2 | 23 | GAPDH | 0.07551 | 0.339122 |
| IACSP95-5000 24 hpi (s.i) | 2 | 24 | GAPDH | 0.382442 | 0.645241 |
| IACSP95-5000 24 hpi (s.i) | 2 | 25 | GAPDH | 0.917411 | 1.179396 |
| IACSP95-5000 24 hpi (s.i) | 2 | 26 | GAPDH | 1.681634 | 1.942805 |
| IACSP95-5000 24 hpi (s.i) | 2 | 27 | GAPDH | 2.438603 | 2.69896 |
| IACSP95-5000 24 hpi (s.i) | 2 | 28 | GAPDH | 3.082315 | 3.341859 |
| IACSP95-5000 24 hpi (s.i) | 2 | 29 | GAPDH | 3.642264 | 3.900994 |
| IACSP95-5000 24 hpi (s.i) | 2 | 30 | GAPDH | 4.184134 | 4.44205 |
| IACSP95-5000 24 hpi (s.i) | 2 | 31 | GAPDH | 4.661243 | 4.918345 |
| IACSP95-5000 24 hpi (s.i) | 2 | 32 | GAPDH | 5.098135 | 5.354423 |
| IACSP95-5000 24 hpi (s.i) | 2 | 33 | GAPDH | 5.47903 | 5.734505 |
| IACSP95-5000 24 hpi (s.i) | 2 | 34 | GAPDH | 5.830581 | 6.085242 |
| IACSP95-5000 24 hpi (s.i) | 2 | 35 | GAPDH | 6.129285 | 6.383132 |
| IACSP95-5000 24 hpi (s.i) | 2 | 36 | GAPDH | 6.393624 | 6.646657 |
| IACSP95-5000 24 hpi (s.i) | 2 | 37 | GAPDH | 6.645686 | 6.897905 |
| IACSP95-5000 24 hpi (s.i) | 2 | 38 | GAPDH | 6.855097 | 7.106503 |
| IACSP95-5000 24 hpi (s.i) | 2 | 39 | GAPDH | 7.047594 | 7.298186 |
| IACSP95-5000 24 hpi (s.i) | 2 | 40 | GAPDH | 7.227981 | 7.477758 |
| IACSP95-5000 24 hpi (s.i) | 3 | 1 | GAPDH | -0.27565 | -0.00653 |
| IACSP95-5000 24 hpi (s.i) | 3 | 2 | GAPDH | -0.27231 | -0.00321 |
| IACSP95-5000 24 hpi (s.i) | 3 | 3 | GAPDH | -0.2703 | -0.00123 |
| IACSP95-5000 24 hpi (s.i) | 3 | 4 | GAPDH | -0.2691 | -5.5E-05 |
| IACSP95-5000 24 hpi (s.i) | 3 | 5 | GAPDH | -0.26874 | 0.000287 |
| IACSP95-5000 24 hpi (s.i) | 3 | 6 | GAPDH | -0.26803 | 0.000975 |
| IACSP95-5000 24 hpi (s.i) | 3 | 7 | GAPDH | -0.26828 | 0.000702 |
| IACSP95-5000 24 hpi (s.i) | 3 | 8 | GAPDH | -0.267 | 0.001954 |
| IACSP95-5000 24 hpi (s.i) | 3 | 9 | GAPDH | -0.26805 | 0.000882 |
| IACSP95-5000 24 hpi (s.i) | 3 | 10 | GAPDH | -0.26895 | -4.1E-05 |
| IACSP95-5000 24 hpi (s.i) | 3 | 11 | GAPDH | -0.27008 | -0.00119 |
| IACSP95-5000 24 hpi (s.i) | 3 | 12 | GAPDH | -0.27065 | -0.00179 |
| IACSP95-5000 24 hpi (s.i) | 3 | 13 | GAPDH | -0.27024 | -0.0014 |
| IACSP95-5000 24 hpi (s.i) | 3 | 14 | GAPDH | -0.26967 | -0.00085 |
| IACSP95-5000 24 hpi (s.i) | 3 | 15 | GAPDH | -0.27035 | -0.00156 |
| IACSP95-5000 24 hpi (s.i) | 3 | 16 | GAPDH | -0.26898 | -0.00021 |
| IACSP95-5000 24 hpi (s.i) | 3 | 17 | GAPDH | -0.26521 | 0.003533 |
| IACSP95-5000 24 hpi (s.i) | 3 | 18 | GAPDH | -0.26094 | 0.007787 |
| IACSP95-5000 24 hpi (s.i) | 3 | 19 | GAPDH | -0.24927 | 0.019426 |
| IACSP95-5000 24 hpi (s.i) | 3 | 20 | GAPDH | -0.22626 | 0.042417 |
| IACSP95-5000 24 hpi (s.i) | 3 | 21 | GAPDH | -0.18529 | 0.083363 |
| IACSP95-5000 24 hpi (s.i) | 3 | 22 | GAPDH | -0.10657 | 0.162064 |
| IACSP95-5000 24 hpi (s.i) | 3 | 23 | GAPDH | 0.045872 | 0.314479 |
| IACSP95-5000 24 hpi (s.i) | 3 | 24 | GAPDH | 0.329758 | 0.598342 |
| IACSP95-5000 24 hpi (s.i) | 3 | 25 | GAPDH | 0.819436 | 1.087996 |
| IACSP95-5000 24 hpi (s.i) | 3 | 26 | GAPDH | 1.518816 | 1.787353 |
| IACSP95-5000 24 hpi (s.i) | 3 | 27 | GAPDH | 2.219239 | 2.487753 |
| IACSP95-5000 24 hpi (s.i) | 3 | 28 | GAPDH | 2.814885 | 3.083375 |
| IACSP95-5000 24 hpi (s.i) | 3 | 29 | GAPDH | 3.341769 | 3.610236 |
| IACSP95-5000 24 hpi (s.i) | 3 | 30 | GAPDH | 3.843442 | 4.111886 |
| IACSP95-5000 24 hpi (s.i) | 3 | 31 | GAPDH | 4.284374 | 4.552795 |
| IACSP95-5000 24 hpi (s.i) | 3 | 32 | GAPDH | 4.689066 | 4.957463 |
| IACSP95-5000 24 hpi (s.i) | 3 | 33 | GAPDH | 5.037788 | 5.306162 |
| IACSP95-5000 24 hpi (s.i) | 3 | 34 | GAPDH | 5.362493 | 5.630844 |
| IACSP95-5000 24 hpi (s.i) | 3 | 35 | GAPDH | 5.635729 | 5.904057 |
| IACSP95-5000 24 hpi (s.i) | 3 | 36 | GAPDH | 5.88499 | 6.153294 |
| IACSP95-5000 24 hpi (s.i) | 3 | 37 | GAPDH | 6.102845 | 6.371126 |
| IACSP95-5000 24 hpi (s.i) | 3 | 38 | GAPDH | 6.298959 | 6.567217 |
| IACSP95-5000 24 hpi (s.i) | 3 | 39 | GAPDH | 6.46611 | 6.734345 |
| IACSP95-5000 24 hpi (s.i) | 3 | 40 | GAPDH | 6.622809 | 6.891021 |
| IACSP95-5000 72 hpi (m.i) | 1 | 1 | GAPDH | -0.2952 | -0.00392 |
| IACSP95-5000 72 hpi (m.i) | 1 | 2 | GAPDH | -0.29394 | -0.00301 |
| IACSP95-5000 72 hpi (m.i) | 1 | 3 | GAPDH | -0.29054 | 2.01E-05 |
| IACSP95-5000 72 hpi (m.i) | 1 | 4 | GAPDH | -0.29043 | -0.00023 |
| IACSP95-5000 72 hpi (m.i) | 1 | 5 | GAPDH | -0.29 | -0.00016 |
| IACSP95-5000 72 hpi (m.i) | 1 | 6 | GAPDH | -0.28946 | 2.18E-05 |
| IACSP95-5000 72 hpi (m.i) | 1 | 7 | GAPDH | -0.28907 | 4.17E-05 |
| IACSP95-5000 72 hpi (m.i) | 1 | 8 | GAPDH | -0.28755 | 0.001201 |
| IACSP95-5000 72 hpi (m.i) | 1 | 9 | GAPDH | -0.28732 | 0.001073 |
| IACSP95-5000 72 hpi (m.i) | 1 | 10 | GAPDH | -0.287 | 0.001032 |
| IACSP95-5000 72 hpi (m.i) | 1 | 11 | GAPDH | -0.28896 | -0.00129 |
| IACSP95-5000 72 hpi (m.i) | 1 | 12 | GAPDH | -0.28795 | -0.00064 |
| IACSP95-5000 72 hpi (m.i) | 1 | 13 | GAPDH | -0.2869 | 4.97E-05 |
| IACSP95-5000 72 hpi (m.i) | 1 | 14 | GAPDH | -0.28679 | -0.00021 |
| IACSP95-5000 72 hpi (m.i) | 1 | 15 | GAPDH | -0.29001 | -0.00379 |
| IACSP95-5000 72 hpi (m.i) | 1 | 16 | GAPDH | -0.28645 | -0.00059 |
| IACSP95-5000 72 hpi (m.i) | 1 | 17 | GAPDH | -0.28507 | 0.000428 |
| IACSP95-5000 72 hpi (m.i) | 1 | 18 | GAPDH | -0.2821 | 0.003044 |
| IACSP95-5000 72 hpi (m.i) | 1 | 19 | GAPDH | -0.27361 | 0.011166 |
| IACSP95-5000 72 hpi (m.i) | 1 | 20 | GAPDH | -0.25848 | 0.025936 |
| IACSP95-5000 72 hpi (m.i) | 1 | 21 | GAPDH | -0.22522 | 0.058835 |
| IACSP95-5000 72 hpi (m.i) | 1 | 22 | GAPDH | -0.16096 | 0.122729 |
| IACSP95-5000 72 hpi (m.i) | 1 | 23 | GAPDH | -0.03917 | 0.244161 |
| IACSP95-5000 72 hpi (m.i) | 1 | 24 | GAPDH | 0.18951 | 0.472479 |
| IACSP95-5000 72 hpi (m.i) | 1 | 25 | GAPDH | 0.604289 | 0.886897 |
| IACSP95-5000 72 hpi (m.i) | 1 | 26 | GAPDH | 1.273075 | 1.555322 |
| IACSP95-5000 72 hpi (m.i) | 1 | 27 | GAPDH | 2.04231 | 2.324195 |
| IACSP95-5000 72 hpi (m.i) | 1 | 28 | GAPDH | 2.718686 | 3.00021 |
| IACSP95-5000 72 hpi (m.i) | 1 | 29 | GAPDH | 3.283287 | 3.564449 |
| IACSP95-5000 72 hpi (m.i) | 1 | 30 | GAPDH | 3.831731 | 4.112532 |
| IACSP95-5000 72 hpi (m.i) | 1 | 31 | GAPDH | 4.314025 | 4.594465 |
| IACSP95-5000 72 hpi (m.i) | 1 | 32 | GAPDH | 4.773027 | 5.053105 |
| IACSP95-5000 72 hpi (m.i) | 1 | 33 | GAPDH | 5.168631 | 5.448347 |
| IACSP95-5000 72 hpi (m.i) | 1 | 34 | GAPDH | 5.53184 | 5.811194 |
| IACSP95-5000 72 hpi (m.i) | 1 | 35 | GAPDH | 5.842842 | 6.121835 |
| IACSP95-5000 72 hpi (m.i) | 1 | 36 | GAPDH | 6.120298 | 6.39893 |
| IACSP95-5000 72 hpi (m.i) | 1 | 37 | GAPDH | 6.395502 | 6.673772 |
| IACSP95-5000 72 hpi (m.i) | 1 | 38 | GAPDH | 6.611507 | 6.889416 |
| IACSP95-5000 72 hpi (m.i) | 1 | 39 | GAPDH | 6.813202 | 7.090749 |
| IACSP95-5000 72 hpi (m.i) | 1 | 40 | GAPDH | 7.00777 | 7.284956 |
| IACSP95-5000 72 hpi (m.i) | 2 | 1 | GAPDH | -0.28461 | -0.00516 |
| IACSP95-5000 72 hpi (m.i) | 2 | 2 | GAPDH | -0.28156 | -0.00202 |
| IACSP95-5000 72 hpi (m.i) | 2 | 3 | GAPDH | -0.28017 | -0.00053 |
| IACSP95-5000 72 hpi (m.i) | 2 | 4 | GAPDH | -0.27963 | 0.000103 |
| IACSP95-5000 72 hpi (m.i) | 2 | 5 | GAPDH | -0.27934 | 0.000483 |
| IACSP95-5000 72 hpi (m.i) | 2 | 6 | GAPDH | -0.27828 | 0.001641 |
| IACSP95-5000 72 hpi (m.i) | 2 | 7 | GAPDH | -0.27957 | 0.000442 |
| IACSP95-5000 72 hpi (m.i) | 2 | 8 | GAPDH | -0.28061 | -0.00051 |
| IACSP95-5000 72 hpi (m.i) | 2 | 9 | GAPDH | -0.27933 | 0.000865 |
| IACSP95-5000 72 hpi (m.i) | 2 | 10 | GAPDH | -0.27949 | 0.000803 |
| IACSP95-5000 72 hpi (m.i) | 2 | 11 | GAPDH | -0.28278 | -0.0024 |
| IACSP95-5000 72 hpi (m.i) | 2 | 12 | GAPDH | -0.2804 | 7.77E-05 |
| IACSP95-5000 72 hpi (m.i) | 2 | 13 | GAPDH | -0.28213 | -0.00156 |
| IACSP95-5000 72 hpi (m.i) | 2 | 14 | GAPDH | -0.2817 | -0.00104 |
| IACSP95-5000 72 hpi (m.i) | 2 | 15 | GAPDH | -0.28194 | -0.00119 |
| IACSP95-5000 72 hpi (m.i) | 2 | 16 | GAPDH | -0.28208 | -0.00123 |
| IACSP95-5000 72 hpi (m.i) | 2 | 17 | GAPDH | -0.2801 | 0.000835 |
| IACSP95-5000 72 hpi (m.i) | 2 | 18 | GAPDH | -0.27782 | 0.003211 |
| IACSP95-5000 72 hpi (m.i) | 2 | 19 | GAPDH | -0.26992 | 0.011201 |
| IACSP95-5000 72 hpi (m.i) | 2 | 20 | GAPDH | -0.2526 | 0.028618 |
| IACSP95-5000 72 hpi (m.i) | 2 | 21 | GAPDH | -0.22324 | 0.058068 |
| IACSP95-5000 72 hpi (m.i) | 2 | 22 | GAPDH | -0.16577 | 0.115628 |
| IACSP95-5000 72 hpi (m.i) | 2 | 23 | GAPDH | -0.05326 | 0.228236 |
| IACSP95-5000 72 hpi (m.i) | 2 | 24 | GAPDH | 0.158243 | 0.439829 |
| IACSP95-5000 72 hpi (m.i) | 2 | 25 | GAPDH | 0.543096 | 0.824774 |
| IACSP95-5000 72 hpi (m.i) | 2 | 26 | GAPDH | 1.174067 | 1.455838 |
| IACSP95-5000 72 hpi (m.i) | 2 | 27 | GAPDH | 1.936865 | 2.218729 |
| IACSP95-5000 72 hpi (m.i) | 2 | 28 | GAPDH | 2.609974 | 2.891931 |
| IACSP95-5000 72 hpi (m.i) | 2 | 29 | GAPDH | 3.175683 | 3.457732 |
| IACSP95-5000 72 hpi (m.i) | 2 | 30 | GAPDH | 3.713758 | 3.995899 |
| IACSP95-5000 72 hpi (m.i) | 2 | 31 | GAPDH | 4.193295 | 4.47553 |
| IACSP95-5000 72 hpi (m.i) | 2 | 32 | GAPDH | 4.637362 | 4.919689 |
| IACSP95-5000 72 hpi (m.i) | 2 | 33 | GAPDH | 5.041822 | 5.324242 |
| IACSP95-5000 72 hpi (m.i) | 2 | 34 | GAPDH | 5.395878 | 5.678391 |
| IACSP95-5000 72 hpi (m.i) | 2 | 35 | GAPDH | 5.681462 | 5.964067 |
| IACSP95-5000 72 hpi (m.i) | 2 | 36 | GAPDH | 5.987772 | 6.270469 |
| IACSP95-5000 72 hpi (m.i) | 2 | 37 | GAPDH | 6.235291 | 6.518081 |
| IACSP95-5000 72 hpi (m.i) | 2 | 38 | GAPDH | 6.436336 | 6.719219 |
| IACSP95-5000 72 hpi (m.i) | 2 | 39 | GAPDH | 6.641691 | 6.924667 |
| IACSP95-5000 72 hpi (m.i) | 2 | 40 | GAPDH | 6.844138 | 7.127207 |
| IACSP95-5000 72 hpi (m.i) | 3 | 1 | GAPDH | -0.36643 | -0.00176 |
| IACSP95-5000 72 hpi (m.i) | 3 | 2 | GAPDH | -0.36635 | -0.00156 |
| IACSP95-5000 72 hpi (m.i) | 3 | 3 | GAPDH | -0.36457 | 0.000349 |
| IACSP95-5000 72 hpi (m.i) | 3 | 4 | GAPDH | -0.36299 | 0.002039 |
| IACSP95-5000 72 hpi (m.i) | 3 | 5 | GAPDH | -0.36313 | 0.00202 |
| IACSP95-5000 72 hpi (m.i) | 3 | 6 | GAPDH | -0.36439 | 0.000877 |
| IACSP95-5000 72 hpi (m.i) | 3 | 7 | GAPDH | -0.3664 | -0.00101 |
| IACSP95-5000 72 hpi (m.i) | 3 | 8 | GAPDH | -0.36574 | -0.00024 |
| IACSP95-5000 72 hpi (m.i) | 3 | 9 | GAPDH | -0.36584 | -0.00022 |
| IACSP95-5000 72 hpi (m.i) | 3 | 10 | GAPDH | -0.36478 | 0.000963 |
| IACSP95-5000 72 hpi (m.i) | 3 | 11 | GAPDH | -0.37044 | -0.00458 |
| IACSP95-5000 72 hpi (m.i) | 3 | 12 | GAPDH | -0.36675 | -0.00078 |
| IACSP95-5000 72 hpi (m.i) | 3 | 13 | GAPDH | -0.36836 | -0.00226 |
| IACSP95-5000 72 hpi (m.i) | 3 | 14 | GAPDH | -0.36925 | -0.00303 |
| IACSP95-5000 72 hpi (m.i) | 3 | 15 | GAPDH | -0.3676 | -0.00127 |
| IACSP95-5000 72 hpi (m.i) | 3 | 16 | GAPDH | -0.36665 | -0.0002 |
| IACSP95-5000 72 hpi (m.i) | 3 | 17 | GAPDH | -0.36502 | 0.001545 |
| IACSP95-5000 72 hpi (m.i) | 3 | 18 | GAPDH | -0.3609 | 0.00579 |
| IACSP95-5000 72 hpi (m.i) | 3 | 19 | GAPDH | -0.35301 | 0.013797 |
| IACSP95-5000 72 hpi (m.i) | 3 | 20 | GAPDH | -0.3362 | 0.030726 |
| IACSP95-5000 72 hpi (m.i) | 3 | 21 | GAPDH | -0.30484 | 0.0622 |
| IACSP95-5000 72 hpi (m.i) | 3 | 22 | GAPDH | -0.24422 | 0.122934 |
| IACSP95-5000 72 hpi (m.i) | 3 | 23 | GAPDH | -0.12727 | 0.240008 |
| IACSP95-5000 72 hpi (m.i) | 3 | 24 | GAPDH | 0.096408 | 0.463803 |
| IACSP95-5000 72 hpi (m.i) | 3 | 25 | GAPDH | 0.497686 | 0.865199 |
| IACSP95-5000 72 hpi (m.i) | 3 | 26 | GAPDH | 1.15018 | 1.517811 |
| IACSP95-5000 72 hpi (m.i) | 3 | 27 | GAPDH | 1.91749 | 2.285239 |
| IACSP95-5000 72 hpi (m.i) | 3 | 28 | GAPDH | 2.580914 | 2.948782 |
| IACSP95-5000 72 hpi (m.i) | 3 | 29 | GAPDH | 3.140566 | 3.508552 |
| IACSP95-5000 72 hpi (m.i) | 3 | 30 | GAPDH | 3.663764 | 4.031868 |
| IACSP95-5000 72 hpi (m.i) | 3 | 31 | GAPDH | 4.139152 | 4.507374 |
| IACSP95-5000 72 hpi (m.i) | 3 | 32 | GAPDH | 4.571714 | 4.940054 |
| IACSP95-5000 72 hpi (m.i) | 3 | 33 | GAPDH | 4.943764 | 5.312222 |
| IACSP95-5000 72 hpi (m.i) | 3 | 34 | GAPDH | 5.296745 | 5.665321 |
| IACSP95-5000 72 hpi (m.i) | 3 | 35 | GAPDH | 5.601409 | 5.970103 |
| IACSP95-5000 72 hpi (m.i) | 3 | 36 | GAPDH | 5.860965 | 6.229777 |
| IACSP95-5000 72 hpi (m.i) | 3 | 37 | GAPDH | 6.106797 | 6.475728 |
| IACSP95-5000 72 hpi (m.i) | 3 | 38 | GAPDH | 6.323674 | 6.692722 |
| IACSP95-5000 72 hpi (m.i) | 3 | 39 | GAPDH | 6.507753 | 6.876919 |
| IACSP95-5000 72 hpi (m.i) | 3 | 40 | GAPDH | 6.682838 | 7.052123 |
| IACSP95-5000 72 hpi (s.i) | 1 | 1 | GAPDH | -0.39344 | -0.01155 |
| IACSP95-5000 72 hpi (s.i) | 1 | 2 | GAPDH | -0.38529 | -0.00345 |
| IACSP95-5000 72 hpi (s.i) | 1 | 3 | GAPDH | -0.38455 | -0.00275 |
| IACSP95-5000 72 hpi (s.i) | 1 | 4 | GAPDH | -0.38225 | -0.00048 |
| IACSP95-5000 72 hpi (s.i) | 1 | 5 | GAPDH | -0.38136 | 0.000369 |
| IACSP95-5000 72 hpi (s.i) | 1 | 6 | GAPDH | -0.38045 | 0.001248 |
| IACSP95-5000 72 hpi (s.i) | 1 | 7 | GAPDH | -0.38002 | 0.001636 |
| IACSP95-5000 72 hpi (s.i) | 1 | 8 | GAPDH | -0.37924 | 0.002375 |
| IACSP95-5000 72 hpi (s.i) | 1 | 9 | GAPDH | -0.38021 | 0.001372 |
| IACSP95-5000 72 hpi (s.i) | 1 | 10 | GAPDH | -0.38061 | 0.000933 |
| IACSP95-5000 72 hpi (s.i) | 1 | 11 | GAPDH | -0.38314 | -0.00163 |
| IACSP95-5000 72 hpi (s.i) | 1 | 12 | GAPDH | -0.38247 | -0.00101 |
| IACSP95-5000 72 hpi (s.i) | 1 | 13 | GAPDH | -0.3817 | -0.00027 |
| IACSP95-5000 72 hpi (s.i) | 1 | 14 | GAPDH | -0.38347 | -0.00208 |
| IACSP95-5000 72 hpi (s.i) | 1 | 15 | GAPDH | -0.38231 | -0.00096 |
| IACSP95-5000 72 hpi (s.i) | 1 | 16 | GAPDH | -0.38288 | -0.00156 |
| IACSP95-5000 72 hpi (s.i) | 1 | 17 | GAPDH | -0.3815 | -0.00023 |
| IACSP95-5000 72 hpi (s.i) | 1 | 18 | GAPDH | -0.37819 | 0.003042 |
| IACSP95-5000 72 hpi (s.i) | 1 | 19 | GAPDH | -0.3707 | 0.010497 |
| IACSP95-5000 72 hpi (s.i) | 1 | 20 | GAPDH | -0.35664 | 0.024515 |
| IACSP95-5000 72 hpi (s.i) | 1 | 21 | GAPDH | -0.33002 | 0.051097 |
| IACSP95-5000 72 hpi (s.i) | 1 | 22 | GAPDH | -0.27457 | 0.106512 |
| IACSP95-5000 72 hpi (s.i) | 1 | 23 | GAPDH | -0.1636 | 0.217443 |
| IACSP95-5000 72 hpi (s.i) | 1 | 24 | GAPDH | 0.039984 | 0.42099 |
| IACSP95-5000 72 hpi (s.i) | 1 | 25 | GAPDH | 0.412085 | 0.793052 |
| IACSP95-5000 72 hpi (s.i) | 1 | 26 | GAPDH | 1.026862 | 1.407791 |
| IACSP95-5000 72 hpi (s.i) | 1 | 27 | GAPDH | 1.771551 | 2.152443 |
| IACSP95-5000 72 hpi (s.i) | 1 | 28 | GAPDH | 2.425334 | 2.806187 |
| IACSP95-5000 72 hpi (s.i) | 1 | 29 | GAPDH | 2.974505 | 3.35532 |
| IACSP95-5000 72 hpi (s.i) | 1 | 30 | GAPDH | 3.49179 | 3.872567 |
| IACSP95-5000 72 hpi (s.i) | 1 | 31 | GAPDH | 3.958272 | 4.339011 |
| IACSP95-5000 72 hpi (s.i) | 1 | 32 | GAPDH | 4.37802 | 4.75872 |
| IACSP95-5000 72 hpi (s.i) | 1 | 33 | GAPDH | 4.748891 | 5.129554 |
| IACSP95-5000 72 hpi (s.i) | 1 | 34 | GAPDH | 5.088728 | 5.469352 |
| IACSP95-5000 72 hpi (s.i) | 1 | 35 | GAPDH | 5.378431 | 5.759017 |
| IACSP95-5000 72 hpi (s.i) | 1 | 36 | GAPDH | 5.644638 | 6.025186 |
| IACSP95-5000 72 hpi (s.i) | 1 | 37 | GAPDH | 5.886219 | 6.266728 |
| IACSP95-5000 72 hpi (s.i) | 1 | 38 | GAPDH | 6.085526 | 6.465997 |
| IACSP95-5000 72 hpi (s.i) | 1 | 39 | GAPDH | 6.274483 | 6.654916 |
| IACSP95-5000 72 hpi (s.i) | 1 | 40 | GAPDH | 6.45848 | 6.838875 |
| IACSP95-5000 72 hpi (s.i) | 2 | 1 | GAPDH | -0.38182 | -0.01392 |
| IACSP95-5000 72 hpi (s.i) | 2 | 2 | GAPDH | -0.37279 | -0.00445 |
| IACSP95-5000 72 hpi (s.i) | 2 | 3 | GAPDH | -0.36931 | -0.00054 |
| IACSP95-5000 72 hpi (s.i) | 2 | 4 | GAPDH | -0.36938 | -0.00018 |
| IACSP95-5000 72 hpi (s.i) | 2 | 5 | GAPDH | -0.37039 | -0.00076 |
| IACSP95-5000 72 hpi (s.i) | 2 | 6 | GAPDH | -0.36859 | 0.001477 |
| IACSP95-5000 72 hpi (s.i) | 2 | 7 | GAPDH | -0.37148 | -0.00098 |
| IACSP95-5000 72 hpi (s.i) | 2 | 8 | GAPDH | -0.36909 | 0.001836 |
| IACSP95-5000 72 hpi (s.i) | 2 | 9 | GAPDH | -0.3692 | 0.002168 |
| IACSP95-5000 72 hpi (s.i) | 2 | 10 | GAPDH | -0.37094 | 0.000853 |
| IACSP95-5000 72 hpi (s.i) | 2 | 11 | GAPDH | -0.3732 | -0.00098 |
| IACSP95-5000 72 hpi (s.i) | 2 | 12 | GAPDH | -0.37267 | -8.7E-06 |
| IACSP95-5000 72 hpi (s.i) | 2 | 13 | GAPDH | -0.37429 | -0.0012 |
| IACSP95-5000 72 hpi (s.i) | 2 | 14 | GAPDH | -0.37583 | -0.0023 |
| IACSP95-5000 72 hpi (s.i) | 2 | 15 | GAPDH | -0.37517 | -0.0012 |
| IACSP95-5000 72 hpi (s.i) | 2 | 16 | GAPDH | -0.3764 | -0.00201 |
| IACSP95-5000 72 hpi (s.i) | 2 | 17 | GAPDH | -0.37626 | -0.00143 |
| IACSP95-5000 72 hpi (s.i) | 2 | 18 | GAPDH | -0.37 | 0.005261 |
| IACSP95-5000 72 hpi (s.i) | 2 | 19 | GAPDH | -0.36242 | 0.013272 |
| IACSP95-5000 72 hpi (s.i) | 2 | 20 | GAPDH | -0.34802 | 0.028103 |
| IACSP95-5000 72 hpi (s.i) | 2 | 21 | GAPDH | -0.31563 | 0.06093 |
| IACSP95-5000 72 hpi (s.i) | 2 | 22 | GAPDH | -0.25528 | 0.121713 |
| IACSP95-5000 72 hpi (s.i) | 2 | 23 | GAPDH | -0.13629 | 0.241134 |
| IACSP95-5000 72 hpi (s.i) | 2 | 24 | GAPDH | 0.089235 | 0.46709 |
| IACSP95-5000 72 hpi (s.i) | 2 | 25 | GAPDH | 0.49859 | 0.876878 |
| IACSP95-5000 72 hpi (s.i) | 2 | 26 | GAPDH | 1.169587 | 1.548308 |
| IACSP95-5000 72 hpi (s.i) | 2 | 27 | GAPDH | 1.96986 | 2.349014 |
| IACSP95-5000 72 hpi (s.i) | 2 | 28 | GAPDH | 2.661178 | 3.040765 |
| IACSP95-5000 72 hpi (s.i) | 2 | 29 | GAPDH | 3.235648 | 3.615668 |
| IACSP95-5000 72 hpi (s.i) | 2 | 30 | GAPDH | 3.777927 | 4.15838 |
| IACSP95-5000 72 hpi (s.i) | 2 | 31 | GAPDH | 4.259075 | 4.63996 |
| IACSP95-5000 72 hpi (s.i) | 2 | 32 | GAPDH | 4.711005 | 5.092323 |
| IACSP95-5000 72 hpi (s.i) | 2 | 33 | GAPDH | 5.100215 | 5.481966 |
| IACSP95-5000 72 hpi (s.i) | 2 | 34 | GAPDH | 5.452277 | 5.83446 |
| IACSP95-5000 72 hpi (s.i) | 2 | 35 | GAPDH | 5.762311 | 6.144928 |
| IACSP95-5000 72 hpi (s.i) | 2 | 36 | GAPDH | 6.031668 | 6.414717 |
| IACSP95-5000 72 hpi (s.i) | 2 | 37 | GAPDH | 6.281893 | 6.665375 |
| IACSP95-5000 72 hpi (s.i) | 2 | 38 | GAPDH | 6.503045 | 6.88696 |
| IACSP95-5000 72 hpi (s.i) | 2 | 39 | GAPDH | 6.688679 | 7.073027 |
| IACSP95-5000 72 hpi (s.i) | 2 | 40 | GAPDH | 6.879293 | 7.264074 |
| IACSP95-5000 72 hpi (s.i) | 3 | 1 | GAPDH | -0.33112 | -0.01886 |
| IACSP95-5000 72 hpi (s.i) | 3 | 2 | GAPDH | -0.32003 | -0.0076 |
| IACSP95-5000 72 hpi (s.i) | 3 | 3 | GAPDH | -0.31483 | -0.00224 |
| IACSP95-5000 72 hpi (s.i) | 3 | 4 | GAPDH | -0.31232 | 0.000441 |
| IACSP95-5000 72 hpi (s.i) | 3 | 5 | GAPDH | -0.31104 | 0.001882 |
| IACSP95-5000 72 hpi (s.i) | 3 | 6 | GAPDH | -0.31188 | 0.001214 |
| IACSP95-5000 72 hpi (s.i) | 3 | 7 | GAPDH | -0.31216 | 0.001095 |
| IACSP95-5000 72 hpi (s.i) | 3 | 8 | GAPDH | -0.31204 | 0.001377 |
| IACSP95-5000 72 hpi (s.i) | 3 | 9 | GAPDH | -0.31293 | 0.000652 |
| IACSP95-5000 72 hpi (s.i) | 3 | 10 | GAPDH | -0.31378 | -3E-05 |
| IACSP95-5000 72 hpi (s.i) | 3 | 11 | GAPDH | -0.31577 | -0.00186 |
| IACSP95-5000 72 hpi (s.i) | 3 | 12 | GAPDH | -0.31453 | -0.00045 |
| IACSP95-5000 72 hpi (s.i) | 3 | 13 | GAPDH | -0.31596 | -0.00171 |
| IACSP95-5000 72 hpi (s.i) | 3 | 14 | GAPDH | -0.31785 | -0.00344 |
| IACSP95-5000 72 hpi (s.i) | 3 | 15 | GAPDH | -0.31512 | -0.00055 |
| IACSP95-5000 72 hpi (s.i) | 3 | 16 | GAPDH | -0.31626 | -0.00152 |
| IACSP95-5000 72 hpi (s.i) | 3 | 17 | GAPDH | -0.31531 | -0.00041 |
| IACSP95-5000 72 hpi (s.i) | 3 | 18 | GAPDH | -0.30954 | 0.005534 |
| IACSP95-5000 72 hpi (s.i) | 3 | 19 | GAPDH | -0.30351 | 0.011728 |
| IACSP95-5000 72 hpi (s.i) | 3 | 20 | GAPDH | -0.2878 | 0.027599 |
| IACSP95-5000 72 hpi (s.i) | 3 | 21 | GAPDH | -0.25661 | 0.058952 |
| IACSP95-5000 72 hpi (s.i) | 3 | 22 | GAPDH | -0.19925 | 0.116478 |
| IACSP95-5000 72 hpi (s.i) | 3 | 23 | GAPDH | -0.08432 | 0.231572 |
| IACSP95-5000 72 hpi (s.i) | 3 | 24 | GAPDH | 0.13358 | 0.44964 |
| IACSP95-5000 72 hpi (s.i) | 3 | 25 | GAPDH | 0.528344 | 0.844569 |
| IACSP95-5000 72 hpi (s.i) | 3 | 26 | GAPDH | 1.179634 | 1.496025 |
| IACSP95-5000 72 hpi (s.i) | 3 | 27 | GAPDH | 1.971349 | 2.287904 |
| IACSP95-5000 72 hpi (s.i) | 3 | 28 | GAPDH | 2.666367 | 2.983088 |
| IACSP95-5000 72 hpi (s.i) | 3 | 29 | GAPDH | 3.247334 | 3.56422 |
| IACSP95-5000 72 hpi (s.i) | 3 | 30 | GAPDH | 3.794895 | 4.111945 |
| IACSP95-5000 72 hpi (s.i) | 3 | 31 | GAPDH | 4.279673 | 4.596888 |
| IACSP95-5000 72 hpi (s.i) | 3 | 32 | GAPDH | 4.737349 | 5.054729 |
| IACSP95-5000 72 hpi (s.i) | 3 | 33 | GAPDH | 5.129079 | 5.446624 |
| IACSP95-5000 72 hpi (s.i) | 3 | 34 | GAPDH | 5.494891 | 5.812601 |
| IACSP95-5000 72 hpi (s.i) | 3 | 35 | GAPDH | 5.804811 | 6.122686 |
| IACSP95-5000 72 hpi (s.i) | 3 | 36 | GAPDH | 6.0869 | 6.404941 |
| IACSP95-5000 72 hpi (s.i) | 3 | 37 | GAPDH | 6.34596 | 6.664165 |
| IACSP95-5000 72 hpi (s.i) | 3 | 38 | GAPDH | 6.565773 | 6.884144 |
| IACSP95-5000 72 hpi (s.i) | 3 | 39 | GAPDH | 6.759903 | 7.078439 |
| IACSP95-5000 72 hpi (s.i) | 3 | 40 | GAPDH | 6.946866 | 7.265566 |
| IAC91-1099 24 hpi (m.i) | 1 | 1 | RPL1 | 0.085956 | 0.044511 |
| IAC91-1099 24 hpi (m.i) | 1 | 2 | RPL1 | 0.063201 | 0.021102 |
| IAC91-1099 24 hpi (m.i) | 1 | 3 | RPL1 | 0.052756 | 0.010003 |
| IAC91-1099 24 hpi (m.i) | 1 | 4 | RPL1 | 0.050457 | 0.007049 |
| IAC91-1099 24 hpi (m.i) | 1 | 5 | RPL1 | 0.046393 | 0.002331 |
| IAC91-1099 24 hpi (m.i) | 1 | 6 | RPL1 | 0.045223 | 0.000506 |
| IAC91-1099 24 hpi (m.i) | 1 | 7 | RPL1 | 0.042089 | -0.00328 |
| IAC91-1099 24 hpi (m.i) | 1 | 8 | RPL1 | 0.043742 | -0.00228 |
| IAC91-1099 24 hpi (m.i) | 1 | 9 | RPL1 | 0.042109 | -0.00457 |
| IAC91-1099 24 hpi (m.i) | 1 | 10 | RPL1 | 0.042313 | -0.00502 |
| IAC91-1099 24 hpi (m.i) | 1 | 11 | RPL1 | 0.043968 | -0.00402 |
| IAC91-1099 24 hpi (m.i) | 1 | 12 | RPL1 | 0.045673 | -0.00297 |
| IAC91-1099 24 hpi (m.i) | 1 | 13 | RPL1 | 0.046396 | -0.0029 |
| IAC91-1099 24 hpi (m.i) | 1 | 14 | RPL1 | 0.046658 | -0.00329 |
| IAC91-1099 24 hpi (m.i) | 1 | 15 | RPL1 | 0.045411 | -0.00519 |
| IAC91-1099 24 hpi (m.i) | 1 | 16 | RPL1 | 0.048495 | -0.00277 |
| IAC91-1099 24 hpi (m.i) | 1 | 17 | RPL1 | 0.051907 | -8E-06 |
| IAC91-1099 24 hpi (m.i) | 1 | 18 | RPL1 | 0.053768 | 0.001199 |
| IAC91-1099 24 hpi (m.i) | 1 | 19 | RPL1 | 0.05393 | 0.000707 |
| IAC91-1099 24 hpi (m.i) | 1 | 20 | RPL1 | 0.056018 | 0.00214 |
| IAC91-1099 24 hpi (m.i) | 1 | 21 | RPL1 | 0.058936 | 0.004404 |
| IAC91-1099 24 hpi (m.i) | 1 | 22 | RPL1 | 0.06316 | 0.007974 |
| IAC91-1099 24 hpi (m.i) | 1 | 23 | RPL1 | 0.067984 | 0.012143 |
| IAC91-1099 24 hpi (m.i) | 1 | 24 | RPL1 | 0.079347 | 0.022852 |
| IAC91-1099 24 hpi (m.i) | 1 | 25 | RPL1 | 0.093532 | 0.036383 |
| IAC91-1099 24 hpi (m.i) | 1 | 26 | RPL1 | 0.121957 | 0.064154 |
| IAC91-1099 24 hpi (m.i) | 1 | 27 | RPL1 | 0.172991 | 0.114533 |
| IAC91-1099 24 hpi (m.i) | 1 | 28 | RPL1 | 0.265683 | 0.206571 |
| IAC91-1099 24 hpi (m.i) | 1 | 29 | RPL1 | 0.428951 | 0.369184 |
| IAC91-1099 24 hpi (m.i) | 1 | 30 | RPL1 | 0.726805 | 0.666384 |
| IAC91-1099 24 hpi (m.i) | 1 | 31 | RPL1 | 1.128692 | 1.067616 |
| IAC91-1099 24 hpi (m.i) | 1 | 32 | RPL1 | 1.707679 | 1.645949 |
| IAC91-1099 24 hpi (m.i) | 1 | 33 | RPL1 | 2.368193 | 2.305809 |
| IAC91-1099 24 hpi (m.i) | 1 | 34 | RPL1 | 3.154429 | 3.091391 |
| IAC91-1099 24 hpi (m.i) | 1 | 35 | RPL1 | 3.862993 | 3.7993 |
| IAC91-1099 24 hpi (m.i) | 1 | 36 | RPL1 | 4.586332 | 4.521985 |
| IAC91-1099 24 hpi (m.i) | 1 | 37 | RPL1 | 5.150829 | 5.085827 |
| IAC91-1099 24 hpi (m.i) | 1 | 38 | RPL1 | 5.727001 | 5.661345 |
| IAC91-1099 24 hpi (m.i) | 1 | 39 | RPL1 | 6.173812 | 6.107502 |
| IAC91-1099 24 hpi (m.i) | 1 | 40 | RPL1 | 6.576549 | 6.509584 |
| IAC91-1099 24 hpi (m.i) | 2 | 1 | RPL1 | 0.080117 | 0.045008 |
| IAC91-1099 24 hpi (m.i) | 2 | 2 | RPL1 | 0.057353 | 0.021026 |
| IAC91-1099 24 hpi (m.i) | 2 | 3 | RPL1 | 0.047857 | 0.010313 |
| IAC91-1099 24 hpi (m.i) | 2 | 4 | RPL1 | 0.044411 | 0.005649 |
| IAC91-1099 24 hpi (m.i) | 2 | 5 | RPL1 | 0.041746 | 0.001767 |
| IAC91-1099 24 hpi (m.i) | 2 | 6 | RPL1 | 0.041199 | 2.52E-06 |
| IAC91-1099 24 hpi (m.i) | 2 | 7 | RPL1 | 0.038993 | -0.00342 |
| IAC91-1099 24 hpi (m.i) | 2 | 8 | RPL1 | 0.040725 | -0.00291 |
| IAC91-1099 24 hpi (m.i) | 2 | 9 | RPL1 | 0.041891 | -0.00296 |
| IAC91-1099 24 hpi (m.i) | 2 | 10 | RPL1 | 0.042481 | -0.00358 |
| IAC91-1099 24 hpi (m.i) | 2 | 11 | RPL1 | 0.042725 | -0.00456 |
| IAC91-1099 24 hpi (m.i) | 2 | 12 | RPL1 | 0.046618 | -0.00188 |
| IAC91-1099 24 hpi (m.i) | 2 | 13 | RPL1 | 0.047321 | -0.0024 |
| IAC91-1099 24 hpi (m.i) | 2 | 14 | RPL1 | 0.047784 | -0.00315 |
| IAC91-1099 24 hpi (m.i) | 2 | 15 | RPL1 | 0.049077 | -0.00308 |
| IAC91-1099 24 hpi (m.i) | 2 | 16 | RPL1 | 0.0507 | -0.00267 |
| IAC91-1099 24 hpi (m.i) | 2 | 17 | RPL1 | 0.052244 | -0.00234 |
| IAC91-1099 24 hpi (m.i) | 2 | 18 | RPL1 | 0.054759 | -0.00105 |
| IAC91-1099 24 hpi (m.i) | 2 | 19 | RPL1 | 0.058077 | 0.001054 |
| IAC91-1099 24 hpi (m.i) | 2 | 20 | RPL1 | 0.062048 | 0.003808 |
| IAC91-1099 24 hpi (m.i) | 2 | 21 | RPL1 | 0.063662 | 0.004204 |
| IAC91-1099 24 hpi (m.i) | 2 | 22 | RPL1 | 0.067874 | 0.007199 |
| IAC91-1099 24 hpi (m.i) | 2 | 23 | RPL1 | 0.073118 | 0.011225 |
| IAC91-1099 24 hpi (m.i) | 2 | 24 | RPL1 | 0.083241 | 0.020131 |
| IAC91-1099 24 hpi (m.i) | 2 | 25 | RPL1 | 0.100119 | 0.035791 |
| IAC91-1099 24 hpi (m.i) | 2 | 26 | RPL1 | 0.132142 | 0.066597 |
| IAC91-1099 24 hpi (m.i) | 2 | 27 | RPL1 | 0.185483 | 0.118721 |
| IAC91-1099 24 hpi (m.i) | 2 | 28 | RPL1 | 0.283093 | 0.215114 |
| IAC91-1099 24 hpi (m.i) | 2 | 29 | RPL1 | 0.454328 | 0.385132 |
| IAC91-1099 24 hpi (m.i) | 2 | 30 | RPL1 | 0.768389 | 0.697975 |
| IAC91-1099 24 hpi (m.i) | 2 | 31 | RPL1 | 1.183766 | 1.112134 |
| IAC91-1099 24 hpi (m.i) | 2 | 32 | RPL1 | 1.783907 | 1.711058 |
| IAC91-1099 24 hpi (m.i) | 2 | 33 | RPL1 | 2.455293 | 2.381227 |
| IAC91-1099 24 hpi (m.i) | 2 | 34 | RPL1 | 3.235511 | 3.160227 |
| IAC91-1099 24 hpi (m.i) | 2 | 35 | RPL1 | 3.938317 | 3.861816 |
| IAC91-1099 24 hpi (m.i) | 2 | 36 | RPL1 | 4.640734 | 4.563015 |
| IAC91-1099 24 hpi (m.i) | 2 | 37 | RPL1 | 5.176195 | 5.097259 |
| IAC91-1099 24 hpi (m.i) | 2 | 38 | RPL1 | 5.734768 | 5.654615 |
| IAC91-1099 24 hpi (m.i) | 2 | 39 | RPL1 | 6.147404 | 6.066033 |
| IAC91-1099 24 hpi (m.i) | 2 | 40 | RPL1 | 6.514462 | 6.431874 |
| IAC91-1099 24 hpi (m.i) | 3 | 1 | RPL1 | 0.10943 | 0.048562 |
| IAC91-1099 24 hpi (m.i) | 3 | 2 | RPL1 | 0.087613 | 0.024834 |
| IAC91-1099 24 hpi (m.i) | 3 | 3 | RPL1 | 0.0766 | 0.011911 |
| IAC91-1099 24 hpi (m.i) | 3 | 4 | RPL1 | 0.072231 | 0.005631 |
| IAC91-1099 24 hpi (m.i) | 3 | 5 | RPL1 | 0.072003 | 0.003493 |
| IAC91-1099 24 hpi (m.i) | 3 | 6 | RPL1 | 0.071295 | 0.000874 |
| IAC91-1099 24 hpi (m.i) | 3 | 7 | RPL1 | 0.069453 | -0.00288 |
| IAC91-1099 24 hpi (m.i) | 3 | 8 | RPL1 | 0.071406 | -0.00284 |
| IAC91-1099 24 hpi (m.i) | 3 | 9 | RPL1 | 0.072454 | -0.0037 |
| IAC91-1099 24 hpi (m.i) | 3 | 10 | RPL1 | 0.073178 | -0.00488 |
| IAC91-1099 24 hpi (m.i) | 3 | 11 | RPL1 | 0.07608 | -0.00389 |
| IAC91-1099 24 hpi (m.i) | 3 | 12 | RPL1 | 0.076721 | -0.00516 |
| IAC91-1099 24 hpi (m.i) | 3 | 13 | RPL1 | 0.078861 | -0.00493 |
| IAC91-1099 24 hpi (m.i) | 3 | 14 | RPL1 | 0.080747 | -0.00496 |
| IAC91-1099 24 hpi (m.i) | 3 | 15 | RPL1 | 0.083562 | -0.00405 |
| IAC91-1099 24 hpi (m.i) | 3 | 16 | RPL1 | 0.087608 | -0.00192 |
| IAC91-1099 24 hpi (m.i) | 3 | 17 | RPL1 | 0.089541 | -0.0019 |
| IAC91-1099 24 hpi (m.i) | 3 | 18 | RPL1 | 0.093494 | 0.000147 |
| IAC91-1099 24 hpi (m.i) | 3 | 19 | RPL1 | 0.095925 | 0.000668 |
| IAC91-1099 24 hpi (m.i) | 3 | 20 | RPL1 | 0.100623 | 0.003455 |
| IAC91-1099 24 hpi (m.i) | 3 | 21 | RPL1 | 0.105982 | 0.006904 |
| IAC91-1099 24 hpi (m.i) | 3 | 22 | RPL1 | 0.109018 | 0.008028 |
| IAC91-1099 24 hpi (m.i) | 3 | 23 | RPL1 | 0.117457 | 0.014557 |
| IAC91-1099 24 hpi (m.i) | 3 | 24 | RPL1 | 0.131419 | 0.026609 |
| IAC91-1099 24 hpi (m.i) | 3 | 25 | RPL1 | 0.149071 | 0.04235 |
| IAC91-1099 24 hpi (m.i) | 3 | 26 | RPL1 | 0.186965 | 0.078334 |
| IAC91-1099 24 hpi (m.i) | 3 | 27 | RPL1 | 0.250168 | 0.139626 |
| IAC91-1099 24 hpi (m.i) | 3 | 28 | RPL1 | 0.36046 | 0.248008 |
| IAC91-1099 24 hpi (m.i) | 3 | 29 | RPL1 | 0.550748 | 0.436385 |
| IAC91-1099 24 hpi (m.i) | 3 | 30 | RPL1 | 0.898166 | 0.781893 |
| IAC91-1099 24 hpi (m.i) | 3 | 31 | RPL1 | 1.344496 | 1.226312 |
| IAC91-1099 24 hpi (m.i) | 3 | 32 | RPL1 | 1.979371 | 1.859277 |
| IAC91-1099 24 hpi (m.i) | 3 | 33 | RPL1 | 2.678101 | 2.556097 |
| IAC91-1099 24 hpi (m.i) | 3 | 34 | RPL1 | 3.472993 | 3.349078 |
| IAC91-1099 24 hpi (m.i) | 3 | 35 | RPL1 | 4.181962 | 4.056136 |
| IAC91-1099 24 hpi (m.i) | 3 | 36 | RPL1 | 4.881324 | 4.753588 |
| IAC91-1099 24 hpi (m.i) | 3 | 37 | RPL1 | 5.403128 | 5.273481 |
| IAC91-1099 24 hpi (m.i) | 3 | 38 | RPL1 | 5.929062 | 5.797504 |
| IAC91-1099 24 hpi (m.i) | 3 | 39 | RPL1 | 6.336993 | 6.203526 |
| IAC91-1099 24 hpi (m.i) | 3 | 40 | RPL1 | 6.686715 | 6.551337 |
| IAC91-1099 24 hpi (s.i) | 1 | 1 | RPL1 | -0.28491 | -0.00508 |
| IAC91-1099 24 hpi (s.i) | 1 | 2 | RPL1 | -0.28041 | 0.000117 |
| IAC91-1099 24 hpi (s.i) | 1 | 3 | RPL1 | -0.28131 | -8.3E-05 |
| IAC91-1099 24 hpi (s.i) | 1 | 4 | RPL1 | -0.27924 | 0.002695 |
| IAC91-1099 24 hpi (s.i) | 1 | 5 | RPL1 | -0.28126 | 0.001378 |
| IAC91-1099 24 hpi (s.i) | 1 | 6 | RPL1 | -0.28107 | 0.00227 |
| IAC91-1099 24 hpi (s.i) | 1 | 7 | RPL1 | -0.2826 | 0.001441 |
| IAC91-1099 24 hpi (s.i) | 1 | 8 | RPL1 | -0.28403 | 0.000709 |
| IAC91-1099 24 hpi (s.i) | 1 | 9 | RPL1 | -0.28467 | 0.000774 |
| IAC91-1099 24 hpi (s.i) | 1 | 10 | RPL1 | -0.28764 | -0.00149 |
| IAC91-1099 24 hpi (s.i) | 1 | 11 | RPL1 | -0.2877 | -0.00085 |
| IAC91-1099 24 hpi (s.i) | 1 | 12 | RPL1 | -0.28879 | -0.00124 |
| IAC91-1099 24 hpi (s.i) | 1 | 13 | RPL1 | -0.29157 | -0.00331 |
| IAC91-1099 24 hpi (s.i) | 1 | 14 | RPL1 | -0.29122 | -0.00226 |
| IAC91-1099 24 hpi (s.i) | 1 | 15 | RPL1 | -0.29374 | -0.00408 |
| IAC91-1099 24 hpi (s.i) | 1 | 16 | RPL1 | -0.29361 | -0.00325 |
| IAC91-1099 24 hpi (s.i) | 1 | 17 | RPL1 | -0.29189 | -0.00083 |
| IAC91-1099 24 hpi (s.i) | 1 | 18 | RPL1 | -0.29298 | -0.00121 |
| IAC91-1099 24 hpi (s.i) | 1 | 19 | RPL1 | -0.29544 | -0.00297 |
| IAC91-1099 24 hpi (s.i) | 1 | 20 | RPL1 | -0.29205 | 0.00112 |
| IAC91-1099 24 hpi (s.i) | 1 | 21 | RPL1 | -0.29099 | 0.002879 |
| IAC91-1099 24 hpi (s.i) | 1 | 22 | RPL1 | -0.28627 | 0.008302 |
| IAC91-1099 24 hpi (s.i) | 1 | 23 | RPL1 | -0.28278 | 0.012495 |
| IAC91-1099 24 hpi (s.i) | 1 | 24 | RPL1 | -0.26539 | 0.030587 |
| IAC91-1099 24 hpi (s.i) | 1 | 25 | RPL1 | -0.24215 | 0.054531 |
| IAC91-1099 24 hpi (s.i) | 1 | 26 | RPL1 | -0.19733 | 0.100055 |
| IAC91-1099 24 hpi (s.i) | 1 | 27 | RPL1 | -0.10421 | 0.193871 |
| IAC91-1099 24 hpi (s.i) | 1 | 28 | RPL1 | 0.054033 | 0.352821 |
| IAC91-1099 24 hpi (s.i) | 1 | 29 | RPL1 | 0.333626 | 0.633117 |
| IAC91-1099 24 hpi (s.i) | 1 | 30 | RPL1 | 0.807449 | 1.107642 |
| IAC91-1099 24 hpi (s.i) | 1 | 31 | RPL1 | 1.392005 | 1.692899 |
| IAC91-1099 24 hpi (s.i) | 1 | 32 | RPL1 | 2.142566 | 2.444164 |
| IAC91-1099 24 hpi (s.i) | 1 | 33 | RPL1 | 2.908648 | 3.210948 |
| IAC91-1099 24 hpi (s.i) | 1 | 34 | RPL1 | 3.733265 | 4.036266 |
| IAC91-1099 24 hpi (s.i) | 1 | 35 | RPL1 | 4.430209 | 4.733913 |
| IAC91-1099 24 hpi (s.i) | 1 | 36 | RPL1 | 5.072972 | 5.377378 |
| IAC91-1099 24 hpi (s.i) | 1 | 37 | RPL1 | 5.564505 | 5.869613 |
| IAC91-1099 24 hpi (s.i) | 1 | 38 | RPL1 | 6.034962 | 6.340772 |
| IAC91-1099 24 hpi (s.i) | 1 | 39 | RPL1 | 6.387289 | 6.693802 |
| IAC91-1099 24 hpi (s.i) | 1 | 40 | RPL1 | 6.716669 | 7.023884 |
| IAC91-1099 24 hpi (s.i) | 2 | 1 | RPL1 | -0.28141 | 0.000494 |
| IAC91-1099 24 hpi (s.i) | 2 | 2 | RPL1 | -0.28035 | 0.001991 |
| IAC91-1099 24 hpi (s.i) | 2 | 3 | RPL1 | -0.28349 | -0.00071 |
| IAC91-1099 24 hpi (s.i) | 2 | 4 | RPL1 | -0.28102 | 0.002191 |
| IAC91-1099 24 hpi (s.i) | 2 | 5 | RPL1 | -0.28156 | 0.00209 |
| IAC91-1099 24 hpi (s.i) | 2 | 6 | RPL1 | -0.28278 | 0.001296 |
| IAC91-1099 24 hpi (s.i) | 2 | 7 | RPL1 | -0.28223 | 0.002282 |
| IAC91-1099 24 hpi (s.i) | 2 | 8 | RPL1 | -0.28359 | 0.001357 |
| IAC91-1099 24 hpi (s.i) | 2 | 9 | RPL1 | -0.28375 | 0.001634 |
| IAC91-1099 24 hpi (s.i) | 2 | 10 | RPL1 | -0.28729 | -0.00147 |
| IAC91-1099 24 hpi (s.i) | 2 | 11 | RPL1 | -0.28679 | -0.00053 |
| IAC91-1099 24 hpi (s.i) | 2 | 12 | RPL1 | -0.2886 | -0.00191 |
| IAC91-1099 24 hpi (s.i) | 2 | 13 | RPL1 | -0.29063 | -0.00351 |
| IAC91-1099 24 hpi (s.i) | 2 | 14 | RPL1 | -0.2906 | -0.00305 |
| IAC91-1099 24 hpi (s.i) | 2 | 15 | RPL1 | -0.29141 | -0.00342 |
| IAC91-1099 24 hpi (s.i) | 2 | 16 | RPL1 | -0.29085 | -0.00243 |
| IAC91-1099 24 hpi (s.i) | 2 | 17 | RPL1 | -0.29129 | -0.00243 |
| IAC91-1099 24 hpi (s.i) | 2 | 18 | RPL1 | -0.29095 | -0.00165 |
| IAC91-1099 24 hpi (s.i) | 2 | 19 | RPL1 | -0.29168 | -0.00195 |
| IAC91-1099 24 hpi (s.i) | 2 | 20 | RPL1 | -0.28985 | 0.000311 |
| IAC91-1099 24 hpi (s.i) | 2 | 21 | RPL1 | -0.28609 | 0.004506 |
| IAC91-1099 24 hpi (s.i) | 2 | 22 | RPL1 | -0.28363 | 0.007406 |
| IAC91-1099 24 hpi (s.i) | 2 | 23 | RPL1 | -0.27846 | 0.013005 |
| IAC91-1099 24 hpi (s.i) | 2 | 24 | RPL1 | -0.26106 | 0.030837 |
| IAC91-1099 24 hpi (s.i) | 2 | 25 | RPL1 | -0.2373 | 0.055038 |
| IAC91-1099 24 hpi (s.i) | 2 | 26 | RPL1 | -0.19094 | 0.101828 |
| IAC91-1099 24 hpi (s.i) | 2 | 27 | RPL1 | -0.10081 | 0.192398 |
| IAC91-1099 24 hpi (s.i) | 2 | 28 | RPL1 | 0.048391 | 0.34203 |
| IAC91-1099 24 hpi (s.i) | 2 | 29 | RPL1 | 0.321349 | 0.615422 |
| IAC91-1099 24 hpi (s.i) | 2 | 30 | RPL1 | 0.790962 | 1.08547 |
| IAC91-1099 24 hpi (s.i) | 2 | 31 | RPL1 | 1.397574 | 1.692516 |
| IAC91-1099 24 hpi (s.i) | 2 | 32 | RPL1 | 2.195622 | 2.490999 |
| IAC91-1099 24 hpi (s.i) | 2 | 33 | RPL1 | 3.031729 | 3.32754 |
| IAC91-1099 24 hpi (s.i) | 2 | 34 | RPL1 | 3.945318 | 4.241564 |
| IAC91-1099 24 hpi (s.i) | 2 | 35 | RPL1 | 4.732159 | 5.028839 |
| IAC91-1099 24 hpi (s.i) | 2 | 36 | RPL1 | 5.490206 | 5.787321 |
| IAC91-1099 24 hpi (s.i) | 2 | 37 | RPL1 | 6.046672 | 6.344222 |
| IAC91-1099 24 hpi (s.i) | 2 | 38 | RPL1 | 6.600112 | 6.898096 |
| IAC91-1099 24 hpi (s.i) | 2 | 39 | RPL1 | 7.026257 | 7.324675 |
| IAC91-1099 24 hpi (s.i) | 2 | 40 | RPL1 | 7.393036 | 7.691889 |
| IAC91-1099 24 hpi (s.i) | 3 | 1 | RPL1 | -0.22907 | -0.16581 |
| IAC91-1099 24 hpi (s.i) | 3 | 2 | RPL1 | -0.10341 | -0.04577 |
| IAC91-1099 24 hpi (s.i) | 3 | 3 | RPL1 | -0.07662 | -0.0246 |
| IAC91-1099 24 hpi (s.i) | 3 | 4 | RPL1 | -0.06332 | -0.01692 |
| IAC91-1099 24 hpi (s.i) | 3 | 5 | RPL1 | -0.04736 | -0.00657 |
| IAC91-1099 24 hpi (s.i) | 3 | 6 | RPL1 | -0.03586 | -0.00069 |
| IAC91-1099 24 hpi (s.i) | 3 | 7 | RPL1 | -0.02879 | 0.000764 |
| IAC91-1099 24 hpi (s.i) | 3 | 8 | RPL1 | -0.01729 | 0.006644 |
| IAC91-1099 24 hpi (s.i) | 3 | 9 | RPL1 | -0.00905 | 0.009265 |
| IAC91-1099 24 hpi (s.i) | 3 | 10 | RPL1 | 0.000112 | 0.012809 |
| IAC91-1099 24 hpi (s.i) | 3 | 11 | RPL1 | 0.003222 | 0.010301 |
| IAC91-1099 24 hpi (s.i) | 3 | 12 | RPL1 | 0.01201 | 0.013472 |
| IAC91-1099 24 hpi (s.i) | 3 | 13 | RPL1 | 0.015665 | 0.011509 |
| IAC91-1099 24 hpi (s.i) | 3 | 14 | RPL1 | 0.026377 | 0.016603 |
| IAC91-1099 24 hpi (s.i) | 3 | 15 | RPL1 | 0.018759 | 0.003367 |
| IAC91-1099 24 hpi (s.i) | 3 | 16 | RPL1 | 0.024763 | 0.003753 |
| IAC91-1099 24 hpi (s.i) | 3 | 17 | RPL1 | 0.028551 | 0.001923 |
| IAC91-1099 24 hpi (s.i) | 3 | 18 | RPL1 | 0.031752 | -0.00049 |
| IAC91-1099 24 hpi (s.i) | 3 | 19 | RPL1 | 0.033789 | -0.00407 |
| IAC91-1099 24 hpi (s.i) | 3 | 20 | RPL1 | 0.037075 | -0.00641 |
| IAC91-1099 24 hpi (s.i) | 3 | 21 | RPL1 | 0.04105 | -0.00805 |
| IAC91-1099 24 hpi (s.i) | 3 | 22 | RPL1 | 0.043165 | -0.01155 |
| IAC91-1099 24 hpi (s.i) | 3 | 23 | RPL1 | 0.049281 | -0.01105 |
| IAC91-1099 24 hpi (s.i) | 3 | 24 | RPL1 | 0.058264 | -0.00769 |
| IAC91-1099 24 hpi (s.i) | 3 | 25 | RPL1 | 0.072065 | 0.000495 |
| IAC91-1099 24 hpi (s.i) | 3 | 26 | RPL1 | 0.102009 | 0.024821 |
| IAC91-1099 24 hpi (s.i) | 3 | 27 | RPL1 | 0.148997 | 0.066192 |
| IAC91-1099 24 hpi (s.i) | 3 | 28 | RPL1 | 0.242075 | 0.153652 |
| IAC91-1099 24 hpi (s.i) | 3 | 29 | RPL1 | 0.402187 | 0.308146 |
| IAC91-1099 24 hpi (s.i) | 3 | 30 | RPL1 | 0.690936 | 0.591278 |
| IAC91-1099 24 hpi (s.i) | 3 | 31 | RPL1 | 1.069821 | 0.964545 |
| IAC91-1099 24 hpi (s.i) | 3 | 32 | RPL1 | 1.60373 | 1.492836 |
| IAC91-1099 24 hpi (s.i) | 3 | 33 | RPL1 | 2.1987 | 2.082188 |
| IAC91-1099 24 hpi (s.i) | 3 | 34 | RPL1 | 2.889712 | 2.767583 |
| IAC91-1099 24 hpi (s.i) | 3 | 35 | RPL1 | 3.50858 | 3.380833 |
| IAC91-1099 24 hpi (s.i) | 3 | 36 | RPL1 | 4.128233 | 3.994868 |
| IAC91-1099 24 hpi (s.i) | 3 | 37 | RPL1 | 4.62039 | 4.481407 |
| IAC91-1099 24 hpi (s.i) | 3 | 38 | RPL1 | 5.106726 | 4.962126 |
| IAC91-1099 24 hpi (s.i) | 3 | 39 | RPL1 | 5.475331 | 5.325113 |
| IAC91-1099 24 hpi (s.i) | 3 | 40 | RPL1 | 5.810768 | 5.654932 |
| IAC91-1099 72 hpi (m.i) | 1 | 1 | RPL1 | -0.29719 | -0.00546 |
| IAC91-1099 72 hpi (m.i) | 1 | 2 | RPL1 | -0.2928 | -0.0007 |
| IAC91-1099 72 hpi (m.i) | 1 | 3 | RPL1 | -0.29241 | 5.11E-05 |
| IAC91-1099 72 hpi (m.i) | 1 | 4 | RPL1 | -0.29307 | -0.00024 |
| IAC91-1099 72 hpi (m.i) | 1 | 5 | RPL1 | -0.29347 | -0.00028 |
| IAC91-1099 72 hpi (m.i) | 1 | 6 | RPL1 | -0.29146 | 0.002103 |
| IAC91-1099 72 hpi (m.i) | 1 | 7 | RPL1 | -0.29399 | -6.6E-05 |
| IAC91-1099 72 hpi (m.i) | 1 | 8 | RPL1 | -0.29449 | -0.00019 |
| IAC91-1099 72 hpi (m.i) | 1 | 9 | RPL1 | -0.29447 | 0.000187 |
| IAC91-1099 72 hpi (m.i) | 1 | 10 | RPL1 | -0.29431 | 0.000717 |
| IAC91-1099 72 hpi (m.i) | 1 | 11 | RPL1 | -0.29476 | 0.000623 |
| IAC91-1099 72 hpi (m.i) | 1 | 12 | RPL1 | -0.29692 | -0.00117 |
| IAC91-1099 72 hpi (m.i) | 1 | 13 | RPL1 | -0.29546 | 0.000656 |
| IAC91-1099 72 hpi (m.i) | 1 | 14 | RPL1 | -0.2964 | 7.96E-05 |
| IAC91-1099 72 hpi (m.i) | 1 | 15 | RPL1 | -0.29874 | -0.0019 |
| IAC91-1099 72 hpi (m.i) | 1 | 16 | RPL1 | -0.299 | -0.00179 |
| IAC91-1099 72 hpi (m.i) | 1 | 17 | RPL1 | -0.29917 | -0.00159 |
| IAC91-1099 72 hpi (m.i) | 1 | 18 | RPL1 | -0.29713 | 0.000815 |
| IAC91-1099 72 hpi (m.i) | 1 | 19 | RPL1 | -0.29807 | 0.000238 |
| IAC91-1099 72 hpi (m.i) | 1 | 20 | RPL1 | -0.29898 | -0.00031 |
| IAC91-1099 72 hpi (m.i) | 1 | 21 | RPL1 | -0.30041 | -0.00137 |
| IAC91-1099 72 hpi (m.i) | 1 | 22 | RPL1 | -0.30055 | -0.00115 |
| IAC91-1099 72 hpi (m.i) | 1 | 23 | RPL1 | -0.29519 | 0.004577 |
| IAC91-1099 72 hpi (m.i) | 1 | 24 | RPL1 | -0.28712 | 0.013019 |
| IAC91-1099 72 hpi (m.i) | 1 | 25 | RPL1 | -0.27242 | 0.028077 |
| IAC91-1099 72 hpi (m.i) | 1 | 26 | RPL1 | -0.23802 | 0.062844 |
| IAC91-1099 72 hpi (m.i) | 1 | 27 | RPL1 | -0.17911 | 0.122127 |
| IAC91-1099 72 hpi (m.i) | 1 | 28 | RPL1 | -0.06722 | 0.234379 |
| IAC91-1099 72 hpi (m.i) | 1 | 29 | RPL1 | 0.134162 | 0.436126 |
| IAC91-1099 72 hpi (m.i) | 1 | 30 | RPL1 | 0.501048 | 0.803377 |
| IAC91-1099 72 hpi (m.i) | 1 | 31 | RPL1 | 1.008706 | 1.311401 |
| IAC91-1099 72 hpi (m.i) | 1 | 32 | RPL1 | 1.713964 | 2.017023 |
| IAC91-1099 72 hpi (m.i) | 1 | 33 | RPL1 | 2.483587 | 2.787012 |
| IAC91-1099 72 hpi (m.i) | 1 | 34 | RPL1 | 3.346914 | 3.650704 |
| IAC91-1099 72 hpi (m.i) | 1 | 35 | RPL1 | 4.114076 | 4.418231 |
| IAC91-1099 72 hpi (m.i) | 1 | 36 | RPL1 | 4.865133 | 5.169654 |
| IAC91-1099 72 hpi (m.i) | 1 | 37 | RPL1 | 5.444383 | 5.749269 |
| IAC91-1099 72 hpi (m.i) | 1 | 38 | RPL1 | 6.004043 | 6.309295 |
| IAC91-1099 72 hpi (m.i) | 1 | 39 | RPL1 | 6.433341 | 6.738958 |
| IAC91-1099 72 hpi (m.i) | 1 | 40 | RPL1 | 6.819186 | 7.125169 |
| IAC91-1099 72 hpi (m.i) | 2 | 1 | RPL1 | -0.32749 | -0.00925 |
| IAC91-1099 72 hpi (m.i) | 2 | 2 | RPL1 | -0.32292 | -0.00456 |
| IAC91-1099 72 hpi (m.i) | 2 | 3 | RPL1 | -0.31861 | -0.00014 |
| IAC91-1099 72 hpi (m.i) | 2 | 4 | RPL1 | -0.3191 | -0.00052 |
| IAC91-1099 72 hpi (m.i) | 2 | 5 | RPL1 | -0.31822 | 0.000478 |
| IAC91-1099 72 hpi (m.i) | 2 | 6 | RPL1 | -0.31751 | 0.001308 |
| IAC91-1099 72 hpi (m.i) | 2 | 7 | RPL1 | -0.31871 | 0.00022 |
| IAC91-1099 72 hpi (m.i) | 2 | 8 | RPL1 | -0.31966 | -0.00062 |
| IAC91-1099 72 hpi (m.i) | 2 | 9 | RPL1 | -0.3172 | 0.001954 |
| IAC91-1099 72 hpi (m.i) | 2 | 10 | RPL1 | -0.31877 | 0.000495 |
| IAC91-1099 72 hpi (m.i) | 2 | 11 | RPL1 | -0.31919 | 0.000196 |
| IAC91-1099 72 hpi (m.i) | 2 | 12 | RPL1 | -0.31905 | 0.000447 |
| IAC91-1099 72 hpi (m.i) | 2 | 13 | RPL1 | -0.31944 | 0.000174 |
| IAC91-1099 72 hpi (m.i) | 2 | 14 | RPL1 | -0.31946 | 0.000263 |
| IAC91-1099 72 hpi (m.i) | 2 | 15 | RPL1 | -0.32142 | -0.00157 |
| IAC91-1099 72 hpi (m.i) | 2 | 16 | RPL1 | -0.32169 | -0.00173 |
| IAC91-1099 72 hpi (m.i) | 2 | 17 | RPL1 | -0.32125 | -0.00119 |
| IAC91-1099 72 hpi (m.i) | 2 | 18 | RPL1 | -0.32227 | -0.00209 |
| IAC91-1099 72 hpi (m.i) | 2 | 19 | RPL1 | -0.32294 | -0.00264 |
| IAC91-1099 72 hpi (m.i) | 2 | 20 | RPL1 | -0.32192 | -0.00151 |
| IAC91-1099 72 hpi (m.i) | 2 | 21 | RPL1 | -0.32159 | -0.00106 |
| IAC91-1099 72 hpi (m.i) | 2 | 22 | RPL1 | -0.31882 | 0.001822 |
| IAC91-1099 72 hpi (m.i) | 2 | 23 | RPL1 | -0.31505 | 0.005707 |
| IAC91-1099 72 hpi (m.i) | 2 | 24 | RPL1 | -0.30557 | 0.015301 |
| IAC91-1099 72 hpi (m.i) | 2 | 25 | RPL1 | -0.28984 | 0.031142 |
| IAC91-1099 72 hpi (m.i) | 2 | 26 | RPL1 | -0.25352 | 0.067575 |
| IAC91-1099 72 hpi (m.i) | 2 | 27 | RPL1 | -0.1915 | 0.129714 |
| IAC91-1099 72 hpi (m.i) | 2 | 28 | RPL1 | -0.07372 | 0.247604 |
| IAC91-1099 72 hpi (m.i) | 2 | 29 | RPL1 | 0.136514 | 0.457952 |
| IAC91-1099 72 hpi (m.i) | 2 | 30 | RPL1 | 0.516875 | 0.838427 |
| IAC91-1099 72 hpi (m.i) | 2 | 31 | RPL1 | 1.039325 | 1.360991 |
| IAC91-1099 72 hpi (m.i) | 2 | 32 | RPL1 | 1.760603 | 2.082383 |
| IAC91-1099 72 hpi (m.i) | 2 | 33 | RPL1 | 2.539826 | 2.861721 |
| IAC91-1099 72 hpi (m.i) | 2 | 34 | RPL1 | 3.407898 | 3.729907 |
| IAC91-1099 72 hpi (m.i) | 2 | 35 | RPL1 | 4.168326 | 4.490449 |
| IAC91-1099 72 hpi (m.i) | 2 | 36 | RPL1 | 4.903783 | 5.22602 |
| IAC91-1099 72 hpi (m.i) | 2 | 37 | RPL1 | 5.463871 | 5.786222 |
| IAC91-1099 72 hpi (m.i) | 2 | 38 | RPL1 | 6.010415 | 6.33288 |
| IAC91-1099 72 hpi (m.i) | 2 | 39 | RPL1 | 6.42828 | 6.75086 |
| IAC91-1099 72 hpi (m.i) | 2 | 40 | RPL1 | 6.794693 | 7.117386 |
| IAC91-1099 72 hpi (m.i) | 3 | 1 | RPL1 | -0.38082 | -0.00608 |
| IAC91-1099 72 hpi (m.i) | 3 | 2 | RPL1 | -0.37694 | -0.0019 |
| IAC91-1099 72 hpi (m.i) | 3 | 3 | RPL1 | -0.37466 | 0.000687 |
| IAC91-1099 72 hpi (m.i) | 3 | 4 | RPL1 | -0.37364 | 0.002007 |
| IAC91-1099 72 hpi (m.i) | 3 | 5 | RPL1 | -0.37555 | 0.000402 |
| IAC91-1099 72 hpi (m.i) | 3 | 6 | RPL1 | -0.3761 | 0.000156 |
| IAC91-1099 72 hpi (m.i) | 3 | 7 | RPL1 | -0.37642 | 0.00015 |
| IAC91-1099 72 hpi (m.i) | 3 | 8 | RPL1 | -0.37735 | -0.00048 |
| IAC91-1099 72 hpi (m.i) | 3 | 9 | RPL1 | -0.37744 | -0.00027 |
| IAC91-1099 72 hpi (m.i) | 3 | 10 | RPL1 | -0.37793 | -0.00045 |
| IAC91-1099 72 hpi (m.i) | 3 | 11 | RPL1 | -0.37679 | 0.000992 |
| IAC91-1099 72 hpi (m.i) | 3 | 12 | RPL1 | -0.37817 | -8E-05 |
| IAC91-1099 72 hpi (m.i) | 3 | 13 | RPL1 | -0.3797 | -0.00131 |
| IAC91-1099 72 hpi (m.i) | 3 | 14 | RPL1 | -0.37878 | -8E-05 |
| IAC91-1099 72 hpi (m.i) | 3 | 15 | RPL1 | -0.38098 | -0.00197 |
| IAC91-1099 72 hpi (m.i) | 3 | 16 | RPL1 | -0.38071 | -0.0014 |
| IAC91-1099 72 hpi (m.i) | 3 | 17 | RPL1 | -0.38154 | -0.00193 |
| IAC91-1099 72 hpi (m.i) | 3 | 18 | RPL1 | -0.38225 | -0.00233 |
| IAC91-1099 72 hpi (m.i) | 3 | 19 | RPL1 | -0.38173 | -0.00151 |
| IAC91-1099 72 hpi (m.i) | 3 | 20 | RPL1 | -0.38005 | 0.000476 |
| IAC91-1099 72 hpi (m.i) | 3 | 21 | RPL1 | -0.37883 | 0.002001 |
| IAC91-1099 72 hpi (m.i) | 3 | 22 | RPL1 | -0.37619 | 0.004949 |
| IAC91-1099 72 hpi (m.i) | 3 | 23 | RPL1 | -0.37108 | 0.01036 |
| IAC91-1099 72 hpi (m.i) | 3 | 24 | RPL1 | -0.35999 | 0.021749 |
| IAC91-1099 72 hpi (m.i) | 3 | 25 | RPL1 | -0.34038 | 0.041668 |
| IAC91-1099 72 hpi (m.i) | 3 | 26 | RPL1 | -0.29943 | 0.082928 |
| IAC91-1099 72 hpi (m.i) | 3 | 27 | RPL1 | -0.22534 | 0.157318 |
| IAC91-1099 72 hpi (m.i) | 3 | 28 | RPL1 | -0.09243 | 0.290534 |
| IAC91-1099 72 hpi (m.i) | 3 | 29 | RPL1 | 0.150075 | 0.533342 |
| IAC91-1099 72 hpi (m.i) | 3 | 30 | RPL1 | 0.580754 | 0.964326 |
| IAC91-1099 72 hpi (m.i) | 3 | 31 | RPL1 | 1.164893 | 1.548769 |
| IAC91-1099 72 hpi (m.i) | 3 | 32 | RPL1 | 1.958209 | 2.34239 |
| IAC91-1099 72 hpi (m.i) | 3 | 33 | RPL1 | 2.804255 | 3.18874 |
| IAC91-1099 72 hpi (m.i) | 3 | 34 | RPL1 | 3.743011 | 4.127801 |
| IAC91-1099 72 hpi (m.i) | 3 | 35 | RPL1 | 4.562171 | 4.947266 |
| IAC91-1099 72 hpi (m.i) | 3 | 36 | RPL1 | 5.350361 | 5.735761 |
| IAC91-1099 72 hpi (m.i) | 3 | 37 | RPL1 | 5.942051 | 6.327754 |
| IAC91-1099 72 hpi (m.i) | 3 | 38 | RPL1 | 6.524272 | 6.91028 |
| IAC91-1099 72 hpi (m.i) | 3 | 39 | RPL1 | 6.96457 | 7.350883 |
| IAC91-1099 72 hpi (m.i) | 3 | 40 | RPL1 | 7.358346 | 7.744964 |
| IAC91-1099 72 hpi (s.i) | 1 | 1 | RPL1 | -0.32948 | -0.00944 |
| IAC91-1099 72 hpi (s.i) | 1 | 2 | RPL1 | -0.32514 | -0.0048 |
| IAC91-1099 72 hpi (s.i) | 1 | 3 | RPL1 | -0.3196 | 0.001047 |
| IAC91-1099 72 hpi (s.i) | 1 | 4 | RPL1 | -0.32178 | -0.00082 |
| IAC91-1099 72 hpi (s.i) | 1 | 5 | RPL1 | -0.31745 | 0.003816 |
| IAC91-1099 72 hpi (s.i) | 1 | 6 | RPL1 | -0.31807 | 0.003498 |
| IAC91-1099 72 hpi (s.i) | 1 | 7 | RPL1 | -0.32011 | 0.001766 |
| IAC91-1099 72 hpi (s.i) | 1 | 8 | RPL1 | -0.32276 | -0.00058 |
| IAC91-1099 72 hpi (s.i) | 1 | 9 | RPL1 | -0.32528 | -0.00278 |
| IAC91-1099 72 hpi (s.i) | 1 | 10 | RPL1 | -0.32558 | -0.00278 |
| IAC91-1099 72 hpi (s.i) | 1 | 11 | RPL1 | -0.3275 | -0.00439 |
| IAC91-1099 72 hpi (s.i) | 1 | 12 | RPL1 | -0.32184 | 0.001574 |
| IAC91-1099 72 hpi (s.i) | 1 | 13 | RPL1 | -0.32506 | -0.00134 |
| IAC91-1099 72 hpi (s.i) | 1 | 14 | RPL1 | -0.32899 | -0.00496 |
| IAC91-1099 72 hpi (s.i) | 1 | 15 | RPL1 | -0.32337 | 0.00097 |
| IAC91-1099 72 hpi (s.i) | 1 | 16 | RPL1 | -0.32614 | -0.0015 |
| IAC91-1099 72 hpi (s.i) | 1 | 17 | RPL1 | -0.32486 | 8.95E-05 |
| IAC91-1099 72 hpi (s.i) | 1 | 18 | RPL1 | -0.32519 | 7.07E-05 |
| IAC91-1099 72 hpi (s.i) | 1 | 19 | RPL1 | -0.32442 | 0.001143 |
| IAC91-1099 72 hpi (s.i) | 1 | 20 | RPL1 | -0.32357 | 0.002309 |
| IAC91-1099 72 hpi (s.i) | 1 | 21 | RPL1 | -0.32332 | 0.002863 |
| IAC91-1099 72 hpi (s.i) | 1 | 22 | RPL1 | -0.31764 | 0.008847 |
| IAC91-1099 72 hpi (s.i) | 1 | 23 | RPL1 | -0.30631 | 0.020487 |
| IAC91-1099 72 hpi (s.i) | 1 | 24 | RPL1 | -0.27804 | 0.049061 |
| IAC91-1099 72 hpi (s.i) | 1 | 25 | RPL1 | -0.2373 | 0.09011 |
| IAC91-1099 72 hpi (s.i) | 1 | 26 | RPL1 | -0.14708 | 0.180637 |
| IAC91-1099 72 hpi (s.i) | 1 | 27 | RPL1 | 0.015006 | 0.343031 |
| IAC91-1099 72 hpi (s.i) | 1 | 28 | RPL1 | 0.299065 | 0.627397 |
| IAC91-1099 72 hpi (s.i) | 1 | 29 | RPL1 | 0.761074 | 1.089714 |
| IAC91-1099 72 hpi (s.i) | 1 | 30 | RPL1 | 1.469754 | 1.798701 |
| IAC91-1099 72 hpi (s.i) | 1 | 31 | RPL1 | 2.256195 | 2.58545 |
| IAC91-1099 72 hpi (s.i) | 1 | 32 | RPL1 | 3.168575 | 3.498137 |
| IAC91-1099 72 hpi (s.i) | 1 | 33 | RPL1 | 4.034272 | 4.364141 |
| IAC91-1099 72 hpi (s.i) | 1 | 34 | RPL1 | 4.886221 | 5.216398 |
| IAC91-1099 72 hpi (s.i) | 1 | 35 | RPL1 | 5.581926 | 5.91241 |
| IAC91-1099 72 hpi (s.i) | 1 | 36 | RPL1 | 6.235966 | 6.566757 |
| IAC91-1099 72 hpi (s.i) | 1 | 37 | RPL1 | 6.686329 | 7.017428 |
| IAC91-1099 72 hpi (s.i) | 1 | 38 | RPL1 | 7.145289 | 7.476695 |
| IAC91-1099 72 hpi (s.i) | 1 | 39 | RPL1 | 7.493353 | 7.825066 |
| IAC91-1099 72 hpi (s.i) | 1 | 40 | RPL1 | 7.799592 | 8.131613 |
| IAC91-1099 72 hpi (s.i) | 2 | 1 | RPL1 | -0.38455 | -0.01786 |
| IAC91-1099 72 hpi (s.i) | 2 | 2 | RPL1 | -0.37614 | -0.00933 |
| IAC91-1099 72 hpi (s.i) | 2 | 3 | RPL1 | -0.36611 | 0.000829 |
| IAC91-1099 72 hpi (s.i) | 2 | 4 | RPL1 | -0.36872 | -0.00166 |
| IAC91-1099 72 hpi (s.i) | 2 | 5 | RPL1 | -0.36314 | 0.004054 |
| IAC91-1099 72 hpi (s.i) | 2 | 6 | RPL1 | -0.36551 | 0.001808 |
| IAC91-1099 72 hpi (s.i) | 2 | 7 | RPL1 | -0.36331 | 0.004131 |
| IAC91-1099 72 hpi (s.i) | 2 | 8 | RPL1 | -0.36676 | 0.000813 |
| IAC91-1099 72 hpi (s.i) | 2 | 9 | RPL1 | -0.36966 | -0.00196 |
| IAC91-1099 72 hpi (s.i) | 2 | 10 | RPL1 | -0.36879 | -0.00097 |
| IAC91-1099 72 hpi (s.i) | 2 | 11 | RPL1 | -0.37594 | -0.00799 |
| IAC91-1099 72 hpi (s.i) | 2 | 12 | RPL1 | -0.36528 | 0.002793 |
| IAC91-1099 72 hpi (s.i) | 2 | 13 | RPL1 | -0.36977 | -0.00157 |
| IAC91-1099 72 hpi (s.i) | 2 | 14 | RPL1 | -0.37796 | -0.00963 |
| IAC91-1099 72 hpi (s.i) | 2 | 15 | RPL1 | -0.36814 | 0.000316 |
| IAC91-1099 72 hpi (s.i) | 2 | 16 | RPL1 | -0.36568 | 0.002895 |
| IAC91-1099 72 hpi (s.i) | 2 | 17 | RPL1 | -0.36853 | 0.000173 |
| IAC91-1099 72 hpi (s.i) | 2 | 18 | RPL1 | -0.36909 | -0.00026 |
| IAC91-1099 72 hpi (s.i) | 2 | 19 | RPL1 | -0.36731 | 0.001646 |
| IAC91-1099 72 hpi (s.i) | 2 | 20 | RPL1 | -0.3669 | 0.002187 |
| IAC91-1099 72 hpi (s.i) | 2 | 21 | RPL1 | -0.36682 | 0.002388 |
| IAC91-1099 72 hpi (s.i) | 2 | 22 | RPL1 | -0.36433 | 0.005005 |
| IAC91-1099 72 hpi (s.i) | 2 | 23 | RPL1 | -0.34435 | 0.025112 |
| IAC91-1099 72 hpi (s.i) | 2 | 24 | RPL1 | -0.31297 | 0.056619 |
| IAC91-1099 72 hpi (s.i) | 2 | 25 | RPL1 | -0.27175 | 0.097965 |
| IAC91-1099 72 hpi (s.i) | 2 | 26 | RPL1 | -0.17557 | 0.194275 |
| IAC91-1099 72 hpi (s.i) | 2 | 27 | RPL1 | -0.00207 | 0.367895 |
| IAC91-1099 72 hpi (s.i) | 2 | 28 | RPL1 | 0.300054 | 0.670148 |
| IAC91-1099 72 hpi (s.i) | 2 | 29 | RPL1 | 0.790151 | 1.160371 |
| IAC91-1099 72 hpi (s.i) | 2 | 30 | RPL1 | 1.534693 | 1.905039 |
| IAC91-1099 72 hpi (s.i) | 2 | 31 | RPL1 | 2.355442 | 2.725914 |
| IAC91-1099 72 hpi (s.i) | 2 | 32 | RPL1 | 3.299392 | 3.669991 |
| IAC91-1099 72 hpi (s.i) | 2 | 33 | RPL1 | 4.197299 | 4.568024 |
| IAC91-1099 72 hpi (s.i) | 2 | 34 | RPL1 | 5.087389 | 5.45824 |
| IAC91-1099 72 hpi (s.i) | 2 | 35 | RPL1 | 5.803402 | 6.17438 |
| IAC91-1099 72 hpi (s.i) | 2 | 36 | RPL1 | 6.471884 | 6.842987 |
| IAC91-1099 72 hpi (s.i) | 2 | 37 | RPL1 | 6.954882 | 7.326111 |
| IAC91-1099 72 hpi (s.i) | 2 | 38 | RPL1 | 7.441864 | 7.813221 |
| IAC91-1099 72 hpi (s.i) | 2 | 39 | RPL1 | 7.813733 | 8.185215 |
| IAC91-1099 72 hpi (s.i) | 2 | 40 | RPL1 | 8.126509 | 8.498117 |
| IAC91-1099 72 hpi (s.i) | 3 | 1 | RPL1 | -0.28947 | -0.00384 |
| IAC91-1099 72 hpi (s.i) | 3 | 2 | RPL1 | -0.28771 | -0.00094 |
| IAC91-1099 72 hpi (s.i) | 3 | 3 | RPL1 | -0.28806 | -0.00017 |
| IAC91-1099 72 hpi (s.i) | 3 | 4 | RPL1 | -0.28522 | 0.003798 |
| IAC91-1099 72 hpi (s.i) | 3 | 5 | RPL1 | -0.28902 | 0.001123 |
| IAC91-1099 72 hpi (s.i) | 3 | 6 | RPL1 | -0.29025 | 0.001019 |
| IAC91-1099 72 hpi (s.i) | 3 | 7 | RPL1 | -0.29268 | -0.00028 |
| IAC91-1099 72 hpi (s.i) | 3 | 8 | RPL1 | -0.29724 | -0.00371 |
| IAC91-1099 72 hpi (s.i) | 3 | 9 | RPL1 | -0.29259 | 0.002065 |
| IAC91-1099 72 hpi (s.i) | 3 | 10 | RPL1 | -0.29672 | -0.00093 |
| IAC91-1099 72 hpi (s.i) | 3 | 11 | RPL1 | -0.29238 | 0.00454 |
| IAC91-1099 72 hpi (s.i) | 3 | 12 | RPL1 | -0.30152 | -0.00348 |
| IAC91-1099 72 hpi (s.i) | 3 | 13 | RPL1 | -0.30059 | -0.00142 |
| IAC91-1099 72 hpi (s.i) | 3 | 14 | RPL1 | -0.29754 | 0.002764 |
| IAC91-1099 72 hpi (s.i) | 3 | 15 | RPL1 | -0.30512 | -0.00369 |
| IAC91-1099 72 hpi (s.i) | 3 | 16 | RPL1 | -0.3064 | -0.00385 |
| IAC91-1099 72 hpi (s.i) | 3 | 17 | RPL1 | -0.30639 | -0.00271 |
| IAC91-1099 72 hpi (s.i) | 3 | 18 | RPL1 | -0.30728 | -0.00247 |
| IAC91-1099 72 hpi (s.i) | 3 | 19 | RPL1 | -0.30989 | -0.00395 |
| IAC91-1099 72 hpi (s.i) | 3 | 20 | RPL1 | -0.31009 | -0.00302 |
| IAC91-1099 72 hpi (s.i) | 3 | 21 | RPL1 | -0.30262 | 0.005575 |
| IAC91-1099 72 hpi (s.i) | 3 | 22 | RPL1 | -0.30054 | 0.008789 |
| IAC91-1099 72 hpi (s.i) | 3 | 23 | RPL1 | -0.29133 | 0.019119 |
| IAC91-1099 72 hpi (s.i) | 3 | 24 | RPL1 | -0.27364 | 0.037941 |
| IAC91-1099 72 hpi (s.i) | 3 | 25 | RPL1 | -0.23512 | 0.077593 |
| IAC91-1099 72 hpi (s.i) | 3 | 26 | RPL1 | -0.1627 | 0.151141 |
| IAC91-1099 72 hpi (s.i) | 3 | 27 | RPL1 | -0.02748 | 0.287489 |
| IAC91-1099 72 hpi (s.i) | 3 | 28 | RPL1 | 0.209 | 0.525094 |
| IAC91-1099 72 hpi (s.i) | 3 | 29 | RPL1 | 0.615115 | 0.932337 |
| IAC91-1099 72 hpi (s.i) | 3 | 30 | RPL1 | 1.255087 | 1.573437 |
| IAC91-1099 72 hpi (s.i) | 3 | 31 | RPL1 | 1.997283 | 2.316761 |
| IAC91-1099 72 hpi (s.i) | 3 | 32 | RPL1 | 2.872622 | 3.193228 |
| IAC91-1099 72 hpi (s.i) | 3 | 33 | RPL1 | 3.720913 | 4.042647 |
| IAC91-1099 72 hpi (s.i) | 3 | 34 | RPL1 | 4.579475 | 4.902338 |
| IAC91-1099 72 hpi (s.i) | 3 | 35 | RPL1 | 5.271812 | 5.595803 |
| IAC91-1099 72 hpi (s.i) | 3 | 36 | RPL1 | 5.899985 | 6.225104 |
| IAC91-1099 72 hpi (s.i) | 3 | 37 | RPL1 | 6.385069 | 6.711316 |
| IAC91-1099 72 hpi (s.i) | 3 | 38 | RPL1 | 6.860949 | 7.188323 |
| IAC91-1099 72 hpi (s.i) | 3 | 39 | RPL1 | 7.204344 | 7.532847 |
| IAC91-1099 72 hpi (s.i) | 3 | 40 | RPL1 | 7.534717 | 7.864348 |
| IACSP95-5000 24 hpi (m.i) | 1 | 1 | RPL1 | -0.31034 | -0.00316 |
| IACSP95-5000 24 hpi (m.i) | 1 | 2 | RPL1 | -0.30672 | -0.00025 |
| IACSP95-5000 24 hpi (m.i) | 1 | 3 | RPL1 | -0.30532 | 0.000448 |
| IACSP95-5000 24 hpi (m.i) | 1 | 4 | RPL1 | -0.30237 | 0.002693 |
| IACSP95-5000 24 hpi (m.i) | 1 | 5 | RPL1 | -0.30584 | -0.00148 |
| IACSP95-5000 24 hpi (m.i) | 1 | 6 | RPL1 | -0.30134 | 0.002309 |
| IACSP95-5000 24 hpi (m.i) | 1 | 7 | RPL1 | -0.30293 | 1.97E-05 |
| IACSP95-5000 24 hpi (m.i) | 1 | 8 | RPL1 | -0.30235 | -0.00011 |
| IACSP95-5000 24 hpi (m.i) | 1 | 9 | RPL1 | -0.30317 | -0.00163 |
| IACSP95-5000 24 hpi (m.i) | 1 | 10 | RPL1 | -0.30287 | -0.00204 |
| IACSP95-5000 24 hpi (m.i) | 1 | 11 | RPL1 | -0.29666 | 0.003468 |
| IACSP95-5000 24 hpi (m.i) | 1 | 12 | RPL1 | -0.29857 | 0.000853 |
| IACSP95-5000 24 hpi (m.i) | 1 | 13 | RPL1 | -0.29857 | 0.000142 |
| IACSP95-5000 24 hpi (m.i) | 1 | 14 | RPL1 | -0.29672 | 0.001287 |
| IACSP95-5000 24 hpi (m.i) | 1 | 15 | RPL1 | -0.29929 | -0.00199 |
| IACSP95-5000 24 hpi (m.i) | 1 | 16 | RPL1 | -0.30122 | -0.00463 |
| IACSP95-5000 24 hpi (m.i) | 1 | 17 | RPL1 | -0.30109 | -0.0052 |
| IACSP95-5000 24 hpi (m.i) | 1 | 18 | RPL1 | -0.29745 | -0.00227 |
| IACSP95-5000 24 hpi (m.i) | 1 | 19 | RPL1 | -0.29647 | -0.00199 |
| IACSP95-5000 24 hpi (m.i) | 1 | 20 | RPL1 | -0.29543 | -0.00166 |
| IACSP95-5000 24 hpi (m.i) | 1 | 21 | RPL1 | -0.28701 | 0.006056 |
| IACSP95-5000 24 hpi (m.i) | 1 | 22 | RPL1 | -0.28664 | 0.005718 |
| IACSP95-5000 24 hpi (m.i) | 1 | 23 | RPL1 | -0.28249 | 0.009162 |
| IACSP95-5000 24 hpi (m.i) | 1 | 24 | RPL1 | -0.2623 | 0.028647 |
| IACSP95-5000 24 hpi (m.i) | 1 | 25 | RPL1 | -0.23727 | 0.052972 |
| IACSP95-5000 24 hpi (m.i) | 1 | 26 | RPL1 | -0.194 | 0.09554 |
| IACSP95-5000 24 hpi (m.i) | 1 | 27 | RPL1 | -0.11456 | 0.174274 |
| IACSP95-5000 24 hpi (m.i) | 1 | 28 | RPL1 | 0.042202 | 0.330331 |
| IACSP95-5000 24 hpi (m.i) | 1 | 29 | RPL1 | 0.309949 | 0.597372 |
| IACSP95-5000 24 hpi (m.i) | 1 | 30 | RPL1 | 0.77644 | 1.063158 |
| IACSP95-5000 24 hpi (m.i) | 1 | 31 | RPL1 | 1.417695 | 1.703707 |
| IACSP95-5000 24 hpi (m.i) | 1 | 32 | RPL1 | 2.260262 | 2.545568 |
| IACSP95-5000 24 hpi (m.i) | 1 | 33 | RPL1 | 3.147463 | 3.432063 |
| IACSP95-5000 24 hpi (m.i) | 1 | 34 | RPL1 | 4.091425 | 4.37532 |
| IACSP95-5000 24 hpi (m.i) | 1 | 35 | RPL1 | 4.920262 | 5.203452 |
| IACSP95-5000 24 hpi (m.i) | 1 | 36 | RPL1 | 5.681824 | 5.964308 |
| IACSP95-5000 24 hpi (m.i) | 1 | 37 | RPL1 | 6.271871 | 6.553649 |
| IACSP95-5000 24 hpi (m.i) | 1 | 38 | RPL1 | 6.843775 | 7.124848 |
| IACSP95-5000 24 hpi (m.i) | 1 | 39 | RPL1 | 7.262845 | 7.543212 |
| IACSP95-5000 24 hpi (m.i) | 1 | 40 | RPL1 | 7.631447 | 7.911108 |
| IACSP95-5000 24 hpi (m.i) | 2 | 1 | RPL1 | -0.27248 | 0.005733 |
| IACSP95-5000 24 hpi (m.i) | 2 | 2 | RPL1 | -0.27379 | 0.003335 |
| IACSP95-5000 24 hpi (m.i) | 2 | 3 | RPL1 | -0.26884 | 0.0072 |
| IACSP95-5000 24 hpi (m.i) | 2 | 4 | RPL1 | -0.27662 | -0.00166 |
| IACSP95-5000 24 hpi (m.i) | 2 | 5 | RPL1 | -0.27062 | 0.003253 |
| IACSP95-5000 24 hpi (m.i) | 2 | 6 | RPL1 | -0.27354 | -0.00075 |
| IACSP95-5000 24 hpi (m.i) | 2 | 7 | RPL1 | -0.27346 | -0.00175 |
| IACSP95-5000 24 hpi (m.i) | 2 | 8 | RPL1 | -0.27381 | -0.00319 |
| IACSP95-5000 24 hpi (m.i) | 2 | 9 | RPL1 | -0.26815 | 0.001388 |
| IACSP95-5000 24 hpi (m.i) | 2 | 10 | RPL1 | -0.27029 | -0.00184 |
| IACSP95-5000 24 hpi (m.i) | 2 | 11 | RPL1 | -0.26837 | -0.001 |
| IACSP95-5000 24 hpi (m.i) | 2 | 12 | RPL1 | -0.26884 | -0.00255 |
| IACSP95-5000 24 hpi (m.i) | 2 | 13 | RPL1 | -0.26753 | -0.00233 |
| IACSP95-5000 24 hpi (m.i) | 2 | 14 | RPL1 | -0.26401 | 0.000106 |
| IACSP95-5000 24 hpi (m.i) | 2 | 15 | RPL1 | -0.26409 | -0.00106 |
| IACSP95-5000 24 hpi (m.i) | 2 | 16 | RPL1 | -0.26327 | -0.00132 |
| IACSP95-5000 24 hpi (m.i) | 2 | 17 | RPL1 | -0.26168 | -0.00081 |
| IACSP95-5000 24 hpi (m.i) | 2 | 18 | RPL1 | -0.25983 | -4.7E-05 |
| IACSP95-5000 24 hpi (m.i) | 2 | 19 | RPL1 | -0.25856 | 0.000139 |
| IACSP95-5000 24 hpi (m.i) | 2 | 20 | RPL1 | -0.25822 | -0.0006 |
| IACSP95-5000 24 hpi (m.i) | 2 | 21 | RPL1 | -0.25484 | 0.001691 |
| IACSP95-5000 24 hpi (m.i) | 2 | 22 | RPL1 | -0.25032 | 0.005129 |
| IACSP95-5000 24 hpi (m.i) | 2 | 23 | RPL1 | -0.24318 | 0.011178 |
| IACSP95-5000 24 hpi (m.i) | 2 | 24 | RPL1 | -0.23243 | 0.020846 |
| IACSP95-5000 24 hpi (m.i) | 2 | 25 | RPL1 | -0.21248 | 0.03972 |
| IACSP95-5000 24 hpi (m.i) | 2 | 26 | RPL1 | -0.16738 | 0.083733 |
| IACSP95-5000 24 hpi (m.i) | 2 | 27 | RPL1 | -0.08916 | 0.160866 |
| IACSP95-5000 24 hpi (m.i) | 2 | 28 | RPL1 | 0.051586 | 0.300529 |
| IACSP95-5000 24 hpi (m.i) | 2 | 29 | RPL1 | 0.305207 | 0.553066 |
| IACSP95-5000 24 hpi (m.i) | 2 | 30 | RPL1 | 0.748209 | 0.994983 |
| IACSP95-5000 24 hpi (m.i) | 2 | 31 | RPL1 | 1.352017 | 1.597708 |
| IACSP95-5000 24 hpi (m.i) | 2 | 32 | RPL1 | 2.155979 | 2.400586 |
| IACSP95-5000 24 hpi (m.i) | 2 | 33 | RPL1 | 3.004071 | 3.247593 |
| IACSP95-5000 24 hpi (m.i) | 2 | 34 | RPL1 | 3.905661 | 4.148099 |
| IACSP95-5000 24 hpi (m.i) | 2 | 35 | RPL1 | 4.679229 | 4.920584 |
| IACSP95-5000 24 hpi (m.i) | 2 | 36 | RPL1 | 5.408166 | 5.648437 |
| IACSP95-5000 24 hpi (m.i) | 2 | 37 | RPL1 | 5.949573 | 6.188759 |
| IACSP95-5000 24 hpi (m.i) | 2 | 38 | RPL1 | 6.469734 | 6.707836 |
| IACSP95-5000 24 hpi (m.i) | 2 | 39 | RPL1 | 6.866704 | 7.103722 |
| IACSP95-5000 24 hpi (m.i) | 2 | 40 | RPL1 | 7.232562 | 7.468496 |
| IACSP95-5000 24 hpi (m.i) | 3 | 1 | RPL1 | -0.30382 | -0.00385 |
| IACSP95-5000 24 hpi (m.i) | 3 | 2 | RPL1 | -0.2974 | 0.002053 |
| IACSP95-5000 24 hpi (m.i) | 3 | 3 | RPL1 | -0.29919 | -0.00025 |
| IACSP95-5000 24 hpi (m.i) | 3 | 4 | RPL1 | -0.29353 | 0.004898 |
| IACSP95-5000 24 hpi (m.i) | 3 | 5 | RPL1 | -0.2962 | 0.001711 |
| IACSP95-5000 24 hpi (m.i) | 3 | 6 | RPL1 | -0.29627 | 0.001129 |
| IACSP95-5000 24 hpi (m.i) | 3 | 7 | RPL1 | -0.29744 | -0.00055 |
| IACSP95-5000 24 hpi (m.i) | 3 | 8 | RPL1 | -0.29435 | 0.002026 |
| IACSP95-5000 24 hpi (m.i) | 3 | 9 | RPL1 | -0.29744 | -0.00157 |
| IACSP95-5000 24 hpi (m.i) | 3 | 10 | RPL1 | -0.29514 | 0.00022 |
| IACSP95-5000 24 hpi (m.i) | 3 | 11 | RPL1 | -0.29797 | -0.00312 |
| IACSP95-5000 24 hpi (m.i) | 3 | 12 | RPL1 | -0.29612 | -0.00179 |
| IACSP95-5000 24 hpi (m.i) | 3 | 13 | RPL1 | -0.29647 | -0.00265 |
| IACSP95-5000 24 hpi (m.i) | 3 | 14 | RPL1 | -0.29787 | -0.00456 |
| IACSP95-5000 24 hpi (m.i) | 3 | 15 | RPL1 | -0.29448 | -0.00168 |
| IACSP95-5000 24 hpi (m.i) | 3 | 16 | RPL1 | -0.29229 | -8.6E-08 |
| IACSP95-5000 24 hpi (m.i) | 3 | 17 | RPL1 | -0.2935 | -0.00172 |
| IACSP95-5000 24 hpi (m.i) | 3 | 18 | RPL1 | -0.29261 | -0.00135 |
| IACSP95-5000 24 hpi (m.i) | 3 | 19 | RPL1 | -0.29055 | 0.000198 |
| IACSP95-5000 24 hpi (m.i) | 3 | 20 | RPL1 | -0.28888 | 0.001361 |
| IACSP95-5000 24 hpi (m.i) | 3 | 21 | RPL1 | -0.28749 | 0.002239 |
| IACSP95-5000 24 hpi (m.i) | 3 | 22 | RPL1 | -0.28375 | 0.005469 |
| IACSP95-5000 24 hpi (m.i) | 3 | 23 | RPL1 | -0.27742 | 0.011282 |
| IACSP95-5000 24 hpi (m.i) | 3 | 24 | RPL1 | -0.26454 | 0.023656 |
| IACSP95-5000 24 hpi (m.i) | 3 | 25 | RPL1 | -0.24721 | 0.040474 |
| IACSP95-5000 24 hpi (m.i) | 3 | 26 | RPL1 | -0.2084 | 0.078773 |
| IACSP95-5000 24 hpi (m.i) | 3 | 27 | RPL1 | -0.13997 | 0.146692 |
| IACSP95-5000 24 hpi (m.i) | 3 | 28 | RPL1 | -0.01608 | 0.270068 |
| IACSP95-5000 24 hpi (m.i) | 3 | 29 | RPL1 | 0.206785 | 0.49242 |
| IACSP95-5000 24 hpi (m.i) | 3 | 30 | RPL1 | 0.604774 | 0.889897 |
| IACSP95-5000 24 hpi (m.i) | 3 | 31 | RPL1 | 1.169081 | 1.453692 |
| IACSP95-5000 24 hpi (m.i) | 3 | 32 | RPL1 | 1.947757 | 2.231856 |
| IACSP95-5000 24 hpi (m.i) | 3 | 33 | RPL1 | 2.797099 | 3.080686 |
| IACSP95-5000 24 hpi (m.i) | 3 | 34 | RPL1 | 3.715925 | 3.999001 |
| IACSP95-5000 24 hpi (m.i) | 3 | 35 | RPL1 | 4.518422 | 4.800986 |
| IACSP95-5000 24 hpi (m.i) | 3 | 36 | RPL1 | 5.293237 | 5.57529 |
| IACSP95-5000 24 hpi (m.i) | 3 | 37 | RPL1 | 5.882877 | 6.164417 |
| IACSP95-5000 24 hpi (m.i) | 3 | 38 | RPL1 | 6.453454 | 6.734483 |
| IACSP95-5000 24 hpi (m.i) | 3 | 39 | RPL1 | 6.881468 | 7.161985 |
| IACSP95-5000 24 hpi (m.i) | 3 | 40 | RPL1 | 7.255962 | 7.535967 |
| IACSP95-5000 24 hpi (s.i) | 1 | 1 | RPL1 | -0.22949 | 0.002093 |
| IACSP95-5000 24 hpi (s.i) | 1 | 2 | RPL1 | -0.22686 | 0.003596 |
| IACSP95-5000 24 hpi (s.i) | 1 | 3 | RPL1 | -0.22683 | 0.002495 |
| IACSP95-5000 24 hpi (s.i) | 1 | 4 | RPL1 | -0.22712 | 0.001082 |
| IACSP95-5000 24 hpi (s.i) | 1 | 5 | RPL1 | -0.2266 | 0.000475 |
| IACSP95-5000 24 hpi (s.i) | 1 | 6 | RPL1 | -0.2249 | 0.001041 |
| IACSP95-5000 24 hpi (s.i) | 1 | 7 | RPL1 | -0.22709 | -0.00227 |
| IACSP95-5000 24 hpi (s.i) | 1 | 8 | RPL1 | -0.22304 | 0.000647 |
| IACSP95-5000 24 hpi (s.i) | 1 | 9 | RPL1 | -0.22325 | -0.00069 |
| IACSP95-5000 24 hpi (s.i) | 1 | 10 | RPL1 | -0.22085 | 0.000584 |
| IACSP95-5000 24 hpi (s.i) | 1 | 11 | RPL1 | -0.2211 | -0.00079 |
| IACSP95-5000 24 hpi (s.i) | 1 | 12 | RPL1 | -0.21963 | -0.00045 |
| IACSP95-5000 24 hpi (s.i) | 1 | 13 | RPL1 | -0.21915 | -0.00109 |
| IACSP95-5000 24 hpi (s.i) | 1 | 14 | RPL1 | -0.21939 | -0.00246 |
| IACSP95-5000 24 hpi (s.i) | 1 | 15 | RPL1 | -0.21639 | -0.00059 |
| IACSP95-5000 24 hpi (s.i) | 1 | 16 | RPL1 | -0.21545 | -0.00077 |
| IACSP95-5000 24 hpi (s.i) | 1 | 17 | RPL1 | -0.21294 | 0.000608 |
| IACSP95-5000 24 hpi (s.i) | 1 | 18 | RPL1 | -0.21653 | -0.00411 |
| IACSP95-5000 24 hpi (s.i) | 1 | 19 | RPL1 | -0.21197 | -0.00067 |
| IACSP95-5000 24 hpi (s.i) | 1 | 20 | RPL1 | -0.20961 | 0.000557 |
| IACSP95-5000 24 hpi (s.i) | 1 | 21 | RPL1 | -0.20803 | 0.001009 |
| IACSP95-5000 24 hpi (s.i) | 1 | 22 | RPL1 | -0.20253 | 0.005386 |
| IACSP95-5000 24 hpi (s.i) | 1 | 23 | RPL1 | -0.19308 | 0.013709 |
| IACSP95-5000 24 hpi (s.i) | 1 | 24 | RPL1 | -0.18229 | 0.023369 |
| IACSP95-5000 24 hpi (s.i) | 1 | 25 | RPL1 | -0.15726 | 0.047271 |
| IACSP95-5000 24 hpi (s.i) | 1 | 26 | RPL1 | -0.11021 | 0.093193 |
| IACSP95-5000 24 hpi (s.i) | 1 | 27 | RPL1 | -0.03085 | 0.171425 |
| IACSP95-5000 24 hpi (s.i) | 1 | 28 | RPL1 | 0.11625 | 0.317404 |
| IACSP95-5000 24 hpi (s.i) | 1 | 29 | RPL1 | 0.373651 | 0.573677 |
| IACSP95-5000 24 hpi (s.i) | 1 | 30 | RPL1 | 0.825256 | 1.024155 |
| IACSP95-5000 24 hpi (s.i) | 1 | 31 | RPL1 | 1.442218 | 1.639991 |
| IACSP95-5000 24 hpi (s.i) | 1 | 32 | RPL1 | 2.255943 | 2.452589 |
| IACSP95-5000 24 hpi (s.i) | 1 | 33 | RPL1 | 3.119986 | 3.315505 |
| IACSP95-5000 24 hpi (s.i) | 1 | 34 | RPL1 | 4.032196 | 4.226587 |
| IACSP95-5000 24 hpi (s.i) | 1 | 35 | RPL1 | 4.829256 | 5.022521 |
| IACSP95-5000 24 hpi (s.i) | 1 | 36 | RPL1 | 5.577889 | 5.770028 |
| IACSP95-5000 24 hpi (s.i) | 1 | 37 | RPL1 | 6.146705 | 6.337716 |
| IACSP95-5000 24 hpi (s.i) | 1 | 38 | RPL1 | 6.712104 | 6.901989 |
| IACSP95-5000 24 hpi (s.i) | 1 | 39 | RPL1 | 7.122165 | 7.310922 |
| IACSP95-5000 24 hpi (s.i) | 1 | 40 | RPL1 | 7.51298 | 7.70061 |
| IACSP95-5000 24 hpi (s.i) | 2 | 1 | RPL1 | -0.2782 | 0.003026 |
| IACSP95-5000 24 hpi (s.i) | 2 | 2 | RPL1 | -0.27428 | 0.004865 |
| IACSP95-5000 24 hpi (s.i) | 2 | 3 | RPL1 | -0.27121 | 0.005859 |
| IACSP95-5000 24 hpi (s.i) | 2 | 4 | RPL1 | -0.27175 | 0.003232 |
| IACSP95-5000 24 hpi (s.i) | 2 | 5 | RPL1 | -0.27258 | 0.000322 |
| IACSP95-5000 24 hpi (s.i) | 2 | 6 | RPL1 | -0.2716 | -0.00077 |
| IACSP95-5000 24 hpi (s.i) | 2 | 7 | RPL1 | -0.26894 | -0.00019 |
| IACSP95-5000 24 hpi (s.i) | 2 | 8 | RPL1 | -0.26781 | -0.00114 |
| IACSP95-5000 24 hpi (s.i) | 2 | 9 | RPL1 | -0.2646 | -1.3E-05 |
| IACSP95-5000 24 hpi (s.i) | 2 | 10 | RPL1 | -0.26205 | 0.000455 |
| IACSP95-5000 24 hpi (s.i) | 2 | 11 | RPL1 | -0.2631 | -0.00267 |
| IACSP95-5000 24 hpi (s.i) | 2 | 12 | RPL1 | -0.26234 | -0.004 |
| IACSP95-5000 24 hpi (s.i) | 2 | 13 | RPL1 | -0.25776 | -0.00149 |
| IACSP95-5000 24 hpi (s.i) | 2 | 14 | RPL1 | -0.25815 | -0.00396 |
| IACSP95-5000 24 hpi (s.i) | 2 | 15 | RPL1 | -0.25552 | -0.00341 |
| IACSP95-5000 24 hpi (s.i) | 2 | 16 | RPL1 | -0.25004 | -1.2E-05 |
| IACSP95-5000 24 hpi (s.i) | 2 | 17 | RPL1 | -0.24935 | -0.00141 |
| IACSP95-5000 24 hpi (s.i) | 2 | 18 | RPL1 | -0.24664 | -0.00078 |
| IACSP95-5000 24 hpi (s.i) | 2 | 19 | RPL1 | -0.2455 | -0.00172 |
| IACSP95-5000 24 hpi (s.i) | 2 | 20 | RPL1 | -0.24107 | 0.000634 |
| IACSP95-5000 24 hpi (s.i) | 2 | 21 | RPL1 | -0.23619 | 0.003438 |
| IACSP95-5000 24 hpi (s.i) | 2 | 22 | RPL1 | -0.22992 | 0.007627 |
| IACSP95-5000 24 hpi (s.i) | 2 | 23 | RPL1 | -0.2211 | 0.014361 |
| IACSP95-5000 24 hpi (s.i) | 2 | 24 | RPL1 | -0.20145 | 0.031936 |
| IACSP95-5000 24 hpi (s.i) | 2 | 25 | RPL1 | -0.17163 | 0.059678 |
| IACSP95-5000 24 hpi (s.i) | 2 | 26 | RPL1 | -0.11803 | 0.111193 |
| IACSP95-5000 24 hpi (s.i) | 2 | 27 | RPL1 | -0.02069 | 0.20645 |
| IACSP95-5000 24 hpi (s.i) | 2 | 28 | RPL1 | 0.154728 | 0.37979 |
| IACSP95-5000 24 hpi (s.i) | 2 | 29 | RPL1 | 0.467787 | 0.690769 |
| IACSP95-5000 24 hpi (s.i) | 2 | 30 | RPL1 | 0.992982 | 1.213885 |
| IACSP95-5000 24 hpi (s.i) | 2 | 31 | RPL1 | 1.691944 | 1.910767 |
| IACSP95-5000 24 hpi (s.i) | 2 | 32 | RPL1 | 2.580101 | 2.796843 |
| IACSP95-5000 24 hpi (s.i) | 2 | 33 | RPL1 | 3.489497 | 3.704159 |
| IACSP95-5000 24 hpi (s.i) | 2 | 34 | RPL1 | 4.422251 | 4.634833 |
| IACSP95-5000 24 hpi (s.i) | 2 | 35 | RPL1 | 5.213702 | 5.424203 |
| IACSP95-5000 24 hpi (s.i) | 2 | 36 | RPL1 | 5.950209 | 6.15863 |
| IACSP95-5000 24 hpi (s.i) | 2 | 37 | RPL1 | 6.489047 | 6.695388 |
| IACSP95-5000 24 hpi (s.i) | 2 | 38 | RPL1 | 7.020949 | 7.22521 |
| IACSP95-5000 24 hpi (s.i) | 2 | 39 | RPL1 | 7.415429 | 7.61761 |
| IACSP95-5000 24 hpi (s.i) | 2 | 40 | RPL1 | 7.780745 | 7.980845 |
| IACSP95-5000 24 hpi (s.i) | 3 | 1 | RPL1 | -0.24015 | -0.00339 |
| IACSP95-5000 24 hpi (s.i) | 3 | 2 | RPL1 | -0.23592 | -0.00078 |
| IACSP95-5000 24 hpi (s.i) | 3 | 3 | RPL1 | -0.23245 | 0.001074 |
| IACSP95-5000 24 hpi (s.i) | 3 | 4 | RPL1 | -0.23281 | -0.00089 |
| IACSP95-5000 24 hpi (s.i) | 3 | 5 | RPL1 | -0.22963 | 0.000671 |
| IACSP95-5000 24 hpi (s.i) | 3 | 6 | RPL1 | -0.22817 | 0.00052 |
| IACSP95-5000 24 hpi (s.i) | 3 | 7 | RPL1 | -0.22816 | -0.00108 |
| IACSP95-5000 24 hpi (s.i) | 3 | 8 | RPL1 | -0.22648 | -0.00101 |
| IACSP95-5000 24 hpi (s.i) | 3 | 9 | RPL1 | -0.22373 | 0.00012 |
| IACSP95-5000 24 hpi (s.i) | 3 | 10 | RPL1 | -0.2217 | 0.000539 |
| IACSP95-5000 24 hpi (s.i) | 3 | 11 | RPL1 | -0.22053 | 9.44E-05 |
| IACSP95-5000 24 hpi (s.i) | 3 | 12 | RPL1 | -0.21832 | 0.000688 |
| IACSP95-5000 24 hpi (s.i) | 3 | 13 | RPL1 | -0.21688 | 0.000521 |
| IACSP95-5000 24 hpi (s.i) | 3 | 14 | RPL1 | -0.21583 | -4.7E-05 |
| IACSP95-5000 24 hpi (s.i) | 3 | 15 | RPL1 | -0.21337 | 0.000799 |
| IACSP95-5000 24 hpi (s.i) | 3 | 16 | RPL1 | -0.21229 | 0.000272 |
| IACSP95-5000 24 hpi (s.i) | 3 | 17 | RPL1 | -0.21386 | -0.00291 |
| IACSP95-5000 24 hpi (s.i) | 3 | 18 | RPL1 | -0.21054 | -0.00121 |
| IACSP95-5000 24 hpi (s.i) | 3 | 19 | RPL1 | -0.20813 | -0.00041 |
| IACSP95-5000 24 hpi (s.i) | 3 | 20 | RPL1 | -0.20596 | 0.000145 |
| IACSP95-5000 24 hpi (s.i) | 3 | 21 | RPL1 | -0.2053 | -0.00081 |
| IACSP95-5000 24 hpi (s.i) | 3 | 22 | RPL1 | -0.19995 | 0.002933 |
| IACSP95-5000 24 hpi (s.i) | 3 | 23 | RPL1 | -0.19149 | 0.009781 |
| IACSP95-5000 24 hpi (s.i) | 3 | 24 | RPL1 | -0.17746 | 0.022197 |
| IACSP95-5000 24 hpi (s.i) | 3 | 25 | RPL1 | -0.1536 | 0.044437 |
| IACSP95-5000 24 hpi (s.i) | 3 | 26 | RPL1 | -0.10329 | 0.093137 |
| IACSP95-5000 24 hpi (s.i) | 3 | 27 | RPL1 | -0.01614 | 0.178677 |
| IACSP95-5000 24 hpi (s.i) | 3 | 28 | RPL1 | 0.140885 | 0.334086 |
| IACSP95-5000 24 hpi (s.i) | 3 | 29 | RPL1 | 0.415032 | 0.60662 |
| IACSP95-5000 24 hpi (s.i) | 3 | 30 | RPL1 | 0.873961 | 1.063937 |
| IACSP95-5000 24 hpi (s.i) | 3 | 31 | RPL1 | 1.46857 | 1.656932 |
| IACSP95-5000 24 hpi (s.i) | 3 | 32 | RPL1 | 2.213102 | 2.399852 |
| IACSP95-5000 24 hpi (s.i) | 3 | 33 | RPL1 | 2.973804 | 3.158941 |
| IACSP95-5000 24 hpi (s.i) | 3 | 34 | RPL1 | 3.760087 | 3.94361 |
| IACSP95-5000 24 hpi (s.i) | 3 | 35 | RPL1 | 4.417516 | 4.599426 |
| IACSP95-5000 24 hpi (s.i) | 3 | 36 | RPL1 | 5.028548 | 5.208845 |
| IACSP95-5000 24 hpi (s.i) | 3 | 37 | RPL1 | 5.493633 | 5.672318 |
| IACSP95-5000 24 hpi (s.i) | 3 | 38 | RPL1 | 5.930889 | 6.10796 |
| IACSP95-5000 24 hpi (s.i) | 3 | 39 | RPL1 | 6.265701 | 6.441159 |
| IACSP95-5000 24 hpi (s.i) | 3 | 40 | RPL1 | 6.563528 | 6.737373 |
| IACSP95-5000 72 hpi (m.i) | 1 | 1 | RPL1 | -0.27395 | 0.001926 |
| IACSP95-5000 72 hpi (m.i) | 1 | 2 | RPL1 | -0.27384 | 0.000309 |
| IACSP95-5000 72 hpi (m.i) | 1 | 3 | RPL1 | -0.27126 | 0.001153 |
| IACSP95-5000 72 hpi (m.i) | 1 | 4 | RPL1 | -0.26694 | 0.003741 |
| IACSP95-5000 72 hpi (m.i) | 1 | 5 | RPL1 | -0.26852 | 0.000436 |
| IACSP95-5000 72 hpi (m.i) | 1 | 6 | RPL1 | -0.26744 | -0.00022 |
| IACSP95-5000 72 hpi (m.i) | 1 | 7 | RPL1 | -0.26509 | 0.000397 |
| IACSP95-5000 72 hpi (m.i) | 1 | 8 | RPL1 | -0.26394 | -0.00019 |
| IACSP95-5000 72 hpi (m.i) | 1 | 9 | RPL1 | -0.26285 | -0.00083 |
| IACSP95-5000 72 hpi (m.i) | 1 | 10 | RPL1 | -0.26251 | -0.00222 |
| IACSP95-5000 72 hpi (m.i) | 1 | 11 | RPL1 | -0.25939 | -0.00083 |
| IACSP95-5000 72 hpi (m.i) | 1 | 12 | RPL1 | -0.25786 | -0.00104 |
| IACSP95-5000 72 hpi (m.i) | 1 | 13 | RPL1 | -0.25592 | -0.00083 |
| IACSP95-5000 72 hpi (m.i) | 1 | 14 | RPL1 | -0.25549 | -0.00213 |
| IACSP95-5000 72 hpi (m.i) | 1 | 15 | RPL1 | -0.25297 | -0.00135 |
| IACSP95-5000 72 hpi (m.i) | 1 | 16 | RPL1 | -0.25066 | -0.00076 |
| IACSP95-5000 72 hpi (m.i) | 1 | 17 | RPL1 | -0.24993 | -0.00176 |
| IACSP95-5000 72 hpi (m.i) | 1 | 18 | RPL1 | -0.24541 | 0.001023 |
| IACSP95-5000 72 hpi (m.i) | 1 | 19 | RPL1 | -0.24613 | -0.00144 |
| IACSP95-5000 72 hpi (m.i) | 1 | 20 | RPL1 | -0.24178 | 0.00119 |
| IACSP95-5000 72 hpi (m.i) | 1 | 21 | RPL1 | -0.23558 | 0.005653 |
| IACSP95-5000 72 hpi (m.i) | 1 | 22 | RPL1 | -0.22853 | 0.010973 |
| IACSP95-5000 72 hpi (m.i) | 1 | 23 | RPL1 | -0.21316 | 0.024612 |
| IACSP95-5000 72 hpi (m.i) | 1 | 24 | RPL1 | -0.18854 | 0.047497 |
| IACSP95-5000 72 hpi (m.i) | 1 | 25 | RPL1 | -0.14688 | 0.087427 |
| IACSP95-5000 72 hpi (m.i) | 1 | 26 | RPL1 | -0.06908 | 0.163496 |
| IACSP95-5000 72 hpi (m.i) | 1 | 27 | RPL1 | 0.070853 | 0.301693 |
| IACSP95-5000 72 hpi (m.i) | 1 | 28 | RPL1 | 0.322167 | 0.551274 |
| IACSP95-5000 72 hpi (m.i) | 1 | 29 | RPL1 | 0.745321 | 0.972697 |
| IACSP95-5000 72 hpi (m.i) | 1 | 30 | RPL1 | 1.408566 | 1.634209 |
| IACSP95-5000 72 hpi (m.i) | 1 | 31 | RPL1 | 2.208992 | 2.432902 |
| IACSP95-5000 72 hpi (m.i) | 1 | 32 | RPL1 | 3.13603 | 3.358209 |
| IACSP95-5000 72 hpi (m.i) | 1 | 33 | RPL1 | 4.04366 | 4.264106 |
| IACSP95-5000 72 hpi (m.i) | 1 | 34 | RPL1 | 4.941572 | 5.160286 |
| IACSP95-5000 72 hpi (m.i) | 1 | 35 | RPL1 | 5.676908 | 5.893889 |
| IACSP95-5000 72 hpi (m.i) | 1 | 36 | RPL1 | 6.36265 | 6.577899 |
| IACSP95-5000 72 hpi (m.i) | 1 | 37 | RPL1 | 6.858605 | 7.072122 |
| IACSP95-5000 72 hpi (m.i) | 1 | 38 | RPL1 | 7.352272 | 7.564056 |
| IACSP95-5000 72 hpi (m.i) | 1 | 39 | RPL1 | 7.740455 | 7.950507 |
| IACSP95-5000 72 hpi (m.i) | 1 | 40 | RPL1 | 8.067819 | 8.276138 |
| IACSP95-5000 72 hpi (m.i) | 2 | 1 | RPL1 | -0.2611 | 0.007414 |
| IACSP95-5000 72 hpi (m.i) | 2 | 2 | RPL1 | -0.25993 | 0.005806 |
| IACSP95-5000 72 hpi (m.i) | 2 | 3 | RPL1 | -0.25902 | 0.003939 |
| IACSP95-5000 72 hpi (m.i) | 2 | 4 | RPL1 | -0.25649 | 0.003685 |
| IACSP95-5000 72 hpi (m.i) | 2 | 5 | RPL1 | -0.25616 | 0.001247 |
| IACSP95-5000 72 hpi (m.i) | 2 | 6 | RPL1 | -0.25493 | -0.0003 |
| IACSP95-5000 72 hpi (m.i) | 2 | 7 | RPL1 | -0.2526 | -0.00075 |
| IACSP95-5000 72 hpi (m.i) | 2 | 8 | RPL1 | -0.25112 | -0.00205 |
| IACSP95-5000 72 hpi (m.i) | 2 | 9 | RPL1 | -0.24748 | -0.00118 |
| IACSP95-5000 72 hpi (m.i) | 2 | 10 | RPL1 | -0.24527 | -0.00175 |
| IACSP95-5000 72 hpi (m.i) | 2 | 11 | RPL1 | -0.24205 | -0.00131 |
| IACSP95-5000 72 hpi (m.i) | 2 | 12 | RPL1 | -0.24143 | -0.00347 |
| IACSP95-5000 72 hpi (m.i) | 2 | 13 | RPL1 | -0.23705 | -0.00187 |
| IACSP95-5000 72 hpi (m.i) | 2 | 14 | RPL1 | -0.23321 | -0.0008 |
| IACSP95-5000 72 hpi (m.i) | 2 | 15 | RPL1 | -0.23219 | -0.00256 |
| IACSP95-5000 72 hpi (m.i) | 2 | 16 | RPL1 | -0.22922 | -0.00236 |
| IACSP95-5000 72 hpi (m.i) | 2 | 17 | RPL1 | -0.22429 | -0.00021 |
| IACSP95-5000 72 hpi (m.i) | 2 | 18 | RPL1 | -0.2203 | 0.000996 |
| IACSP95-5000 72 hpi (m.i) | 2 | 19 | RPL1 | -0.2158 | 0.002726 |
| IACSP95-5000 72 hpi (m.i) | 2 | 20 | RPL1 | -0.20973 | 0.006017 |
| IACSP95-5000 72 hpi (m.i) | 2 | 21 | RPL1 | -0.2034 | 0.009571 |
| IACSP95-5000 72 hpi (m.i) | 2 | 22 | RPL1 | -0.19331 | 0.01688 |
| IACSP95-5000 72 hpi (m.i) | 2 | 23 | RPL1 | -0.1756 | 0.031812 |
| IACSP95-5000 72 hpi (m.i) | 2 | 24 | RPL1 | -0.14288 | 0.061758 |
| IACSP95-5000 72 hpi (m.i) | 2 | 25 | RPL1 | -0.09433 | 0.107526 |
| IACSP95-5000 72 hpi (m.i) | 2 | 26 | RPL1 | 0.005087 | 0.20417 |
| IACSP95-5000 72 hpi (m.i) | 2 | 27 | RPL1 | 0.180459 | 0.376765 |
| IACSP95-5000 72 hpi (m.i) | 2 | 28 | RPL1 | 0.485782 | 0.679311 |
| IACSP95-5000 72 hpi (m.i) | 2 | 29 | RPL1 | 0.981669 | 1.172421 |
| IACSP95-5000 72 hpi (m.i) | 2 | 30 | RPL1 | 1.729136 | 1.917111 |
| IACSP95-5000 72 hpi (m.i) | 2 | 31 | RPL1 | 2.570673 | 2.755871 |
| IACSP95-5000 72 hpi (m.i) | 2 | 32 | RPL1 | 3.50334 | 3.68576 |
| IACSP95-5000 72 hpi (m.i) | 2 | 33 | RPL1 | 4.384244 | 4.563888 |
| IACSP95-5000 72 hpi (m.i) | 2 | 34 | RPL1 | 5.241201 | 5.418067 |
| IACSP95-5000 72 hpi (m.i) | 2 | 35 | RPL1 | 5.931207 | 6.105296 |
| IACSP95-5000 72 hpi (m.i) | 2 | 36 | RPL1 | 6.534386 | 6.705698 |
| IACSP95-5000 72 hpi (m.i) | 2 | 37 | RPL1 | 6.996771 | 7.165306 |
| IACSP95-5000 72 hpi (m.i) | 2 | 38 | RPL1 | 7.45052 | 7.616277 |
| IACSP95-5000 72 hpi (m.i) | 2 | 39 | RPL1 | 7.766417 | 7.929397 |
| IACSP95-5000 72 hpi (m.i) | 2 | 40 | RPL1 | 8.063565 | 8.223769 |
| IACSP95-5000 72 hpi (m.i) | 3 | 1 | RPL1 | -0.34427 | 0.006725 |
| IACSP95-5000 72 hpi (m.i) | 3 | 2 | RPL1 | -0.34541 | 0.004524 |
| IACSP95-5000 72 hpi (m.i) | 3 | 3 | RPL1 | -0.34537 | 0.003507 |
| IACSP95-5000 72 hpi (m.i) | 3 | 4 | RPL1 | -0.3444 | 0.003428 |
| IACSP95-5000 72 hpi (m.i) | 3 | 5 | RPL1 | -0.3442 | 0.00257 |
| IACSP95-5000 72 hpi (m.i) | 3 | 6 | RPL1 | -0.34481 | 0.000903 |
| IACSP95-5000 72 hpi (m.i) | 3 | 7 | RPL1 | -0.34666 | -0.00199 |
| IACSP95-5000 72 hpi (m.i) | 3 | 8 | RPL1 | -0.3445 | -0.00089 |
| IACSP95-5000 72 hpi (m.i) | 3 | 9 | RPL1 | -0.34476 | -0.00221 |
| IACSP95-5000 72 hpi (m.i) | 3 | 10 | RPL1 | -0.34402 | -0.00252 |
| IACSP95-5000 72 hpi (m.i) | 3 | 11 | RPL1 | -0.3417 | -0.00126 |
| IACSP95-5000 72 hpi (m.i) | 3 | 12 | RPL1 | -0.34156 | -0.00217 |
| IACSP95-5000 72 hpi (m.i) | 3 | 13 | RPL1 | -0.34043 | -0.00209 |
| IACSP95-5000 72 hpi (m.i) | 3 | 14 | RPL1 | -0.33982 | -0.00254 |
| IACSP95-5000 72 hpi (m.i) | 3 | 15 | RPL1 | -0.33818 | -0.00196 |
| IACSP95-5000 72 hpi (m.i) | 3 | 16 | RPL1 | -0.33521 | -3.8E-05 |
| IACSP95-5000 72 hpi (m.i) | 3 | 17 | RPL1 | -0.33626 | -0.00215 |
| IACSP95-5000 72 hpi (m.i) | 3 | 18 | RPL1 | -0.33346 | -0.0004 |
| IACSP95-5000 72 hpi (m.i) | 3 | 19 | RPL1 | -0.33011 | 0.001898 |
| IACSP95-5000 72 hpi (m.i) | 3 | 20 | RPL1 | -0.32872 | 0.002224 |
| IACSP95-5000 72 hpi (m.i) | 3 | 21 | RPL1 | -0.3242 | 0.005691 |
| IACSP95-5000 72 hpi (m.i) | 3 | 22 | RPL1 | -0.31631 | 0.012525 |
| IACSP95-5000 72 hpi (m.i) | 3 | 23 | RPL1 | -0.30451 | 0.023271 |
| IACSP95-5000 72 hpi (m.i) | 3 | 24 | RPL1 | -0.28073 | 0.046002 |
| IACSP95-5000 72 hpi (m.i) | 3 | 25 | RPL1 | -0.24097 | 0.084703 |
| IACSP95-5000 72 hpi (m.i) | 3 | 26 | RPL1 | -0.15939 | 0.165231 |
| IACSP95-5000 72 hpi (m.i) | 3 | 27 | RPL1 | -0.01823 | 0.305338 |
| IACSP95-5000 72 hpi (m.i) | 3 | 28 | RPL1 | 0.23219 | 0.554699 |
| IACSP95-5000 72 hpi (m.i) | 3 | 29 | RPL1 | 0.648486 | 0.96994 |
| IACSP95-5000 72 hpi (m.i) | 3 | 30 | RPL1 | 1.293706 | 1.614106 |
| IACSP95-5000 72 hpi (m.i) | 3 | 31 | RPL1 | 2.053279 | 2.372624 |
| IACSP95-5000 72 hpi (m.i) | 3 | 32 | RPL1 | 2.917982 | 3.236272 |
| IACSP95-5000 72 hpi (m.i) | 3 | 33 | RPL1 | 3.761874 | 4.079108 |
| IACSP95-5000 72 hpi (m.i) | 3 | 34 | RPL1 | 4.572115 | 4.888295 |
| IACSP95-5000 72 hpi (m.i) | 3 | 35 | RPL1 | 5.236543 | 5.551668 |
| IACSP95-5000 72 hpi (m.i) | 3 | 36 | RPL1 | 5.848074 | 6.162144 |
| IACSP95-5000 72 hpi (m.i) | 3 | 37 | RPL1 | 6.291108 | 6.604123 |
| IACSP95-5000 72 hpi (m.i) | 3 | 38 | RPL1 | 6.719951 | 7.03191 |
| IACSP95-5000 72 hpi (m.i) | 3 | 39 | RPL1 | 7.056112 | 7.367017 |
| IACSP95-5000 72 hpi (m.i) | 3 | 40 | RPL1 | 7.343085 | 7.652935 |
| IACSP95-5000 72 hpi (s.i) | 1 | 1 | RPL1 | -0.41172 | -0.01329 |
| IACSP95-5000 72 hpi (s.i) | 1 | 2 | RPL1 | -0.40594 | -0.0068 |
| IACSP95-5000 72 hpi (s.i) | 1 | 3 | RPL1 | -0.40203 | -0.00218 |
| IACSP95-5000 72 hpi (s.i) | 1 | 4 | RPL1 | -0.39988 | 0.000665 |
| IACSP95-5000 72 hpi (s.i) | 1 | 5 | RPL1 | -0.40088 | 0.000373 |
| IACSP95-5000 72 hpi (s.i) | 1 | 6 | RPL1 | -0.40045 | 0.001504 |
| IACSP95-5000 72 hpi (s.i) | 1 | 7 | RPL1 | -0.40351 | -0.00085 |
| IACSP95-5000 72 hpi (s.i) | 1 | 8 | RPL1 | -0.40224 | 0.001125 |
| IACSP95-5000 72 hpi (s.i) | 1 | 9 | RPL1 | -0.40094 | 0.003125 |
| IACSP95-5000 72 hpi (s.i) | 1 | 10 | RPL1 | -0.40517 | -0.0004 |
| IACSP95-5000 72 hpi (s.i) | 1 | 11 | RPL1 | -0.40523 | 0.000243 |
| IACSP95-5000 72 hpi (s.i) | 1 | 12 | RPL1 | -0.40486 | 0.001317 |
| IACSP95-5000 72 hpi (s.i) | 1 | 13 | RPL1 | -0.4068 | 7.63E-05 |
| IACSP95-5000 72 hpi (s.i) | 1 | 14 | RPL1 | -0.40845 | -0.00087 |
| IACSP95-5000 72 hpi (s.i) | 1 | 15 | RPL1 | -0.41193 | -0.00365 |
| IACSP95-5000 72 hpi (s.i) | 1 | 16 | RPL1 | -0.4113 | -0.00231 |
| IACSP95-5000 72 hpi (s.i) | 1 | 17 | RPL1 | -0.41029 | -0.00059 |
| IACSP95-5000 72 hpi (s.i) | 1 | 18 | RPL1 | -0.41385 | -0.00346 |
| IACSP95-5000 72 hpi (s.i) | 1 | 19 | RPL1 | -0.41179 | -0.00069 |
| IACSP95-5000 72 hpi (s.i) | 1 | 20 | RPL1 | -0.40937 | 0.002431 |
| IACSP95-5000 72 hpi (s.i) | 1 | 21 | RPL1 | -0.40837 | 0.004142 |
| IACSP95-5000 72 hpi (s.i) | 1 | 22 | RPL1 | -0.40036 | 0.012849 |
| IACSP95-5000 72 hpi (s.i) | 1 | 23 | RPL1 | -0.3889 | 0.025012 |
| IACSP95-5000 72 hpi (s.i) | 1 | 24 | RPL1 | -0.36597 | 0.048654 |
| IACSP95-5000 72 hpi (s.i) | 1 | 25 | RPL1 | -0.32377 | 0.091552 |
| IACSP95-5000 72 hpi (s.i) | 1 | 26 | RPL1 | -0.24019 | 0.175834 |
| IACSP95-5000 72 hpi (s.i) | 1 | 27 | RPL1 | -0.08602 | 0.330711 |
| IACSP95-5000 72 hpi (s.i) | 1 | 28 | RPL1 | 0.189075 | 0.606509 |
| IACSP95-5000 72 hpi (s.i) | 1 | 29 | RPL1 | 0.649688 | 1.067825 |
| IACSP95-5000 72 hpi (s.i) | 1 | 30 | RPL1 | 1.352968 | 1.771809 |
| IACSP95-5000 72 hpi (s.i) | 1 | 31 | RPL1 | 2.176322 | 2.595866 |
| IACSP95-5000 72 hpi (s.i) | 1 | 32 | RPL1 | 3.096541 | 3.516788 |
| IACSP95-5000 72 hpi (s.i) | 1 | 33 | RPL1 | 3.968568 | 4.38952 |
| IACSP95-5000 72 hpi (s.i) | 1 | 34 | RPL1 | 4.812457 | 5.234111 |
| IACSP95-5000 72 hpi (s.i) | 1 | 35 | RPL1 | 5.487525 | 5.909883 |
| IACSP95-5000 72 hpi (s.i) | 1 | 36 | RPL1 | 6.103032 | 6.526094 |
| IACSP95-5000 72 hpi (s.i) | 1 | 37 | RPL1 | 6.562186 | 6.985951 |
| IACSP95-5000 72 hpi (s.i) | 1 | 38 | RPL1 | 6.998489 | 7.422957 |
| IACSP95-5000 72 hpi (s.i) | 1 | 39 | RPL1 | 7.32874 | 7.753912 |
| IACSP95-5000 72 hpi (s.i) | 1 | 40 | RPL1 | 7.630044 | 8.05592 |
| IACSP95-5000 72 hpi (s.i) | 2 | 1 | RPL1 | -0.40442 | -0.00664 |
| IACSP95-5000 72 hpi (s.i) | 2 | 2 | RPL1 | -0.4012 | -0.00259 |
| IACSP95-5000 72 hpi (s.i) | 2 | 3 | RPL1 | -0.3994 | 1.83E-05 |
| IACSP95-5000 72 hpi (s.i) | 2 | 4 | RPL1 | -0.39791 | 0.002331 |
| IACSP95-5000 72 hpi (s.i) | 2 | 5 | RPL1 | -0.40054 | 0.000532 |
| IACSP95-5000 72 hpi (s.i) | 2 | 6 | RPL1 | -0.40114 | 0.000749 |
| IACSP95-5000 72 hpi (s.i) | 2 | 7 | RPL1 | -0.4036 | -0.00088 |
| IACSP95-5000 72 hpi (s.i) | 2 | 8 | RPL1 | -0.40503 | -0.0015 |
| IACSP95-5000 72 hpi (s.i) | 2 | 9 | RPL1 | -0.40322 | 0.001134 |
| IACSP95-5000 72 hpi (s.i) | 2 | 10 | RPL1 | -0.40516 | 2.06E-05 |
| IACSP95-5000 72 hpi (s.i) | 2 | 11 | RPL1 | -0.40669 | -0.00069 |
| IACSP95-5000 72 hpi (s.i) | 2 | 12 | RPL1 | -0.4082 | -0.00138 |
| IACSP95-5000 72 hpi (s.i) | 2 | 13 | RPL1 | -0.40751 | 0.000136 |
| IACSP95-5000 72 hpi (s.i) | 2 | 14 | RPL1 | -0.40944 | -0.00098 |
| IACSP95-5000 72 hpi (s.i) | 2 | 15 | RPL1 | -0.41001 | -0.00072 |
| IACSP95-5000 72 hpi (s.i) | 2 | 16 | RPL1 | -0.41001 | 9.72E-05 |
| IACSP95-5000 72 hpi (s.i) | 2 | 17 | RPL1 | -0.41148 | -0.00055 |
| IACSP95-5000 72 hpi (s.i) | 2 | 18 | RPL1 | -0.41418 | -0.00243 |
| IACSP95-5000 72 hpi (s.i) | 2 | 19 | RPL1 | -0.41513 | -0.00256 |
| IACSP95-5000 72 hpi (s.i) | 2 | 20 | RPL1 | -0.41157 | 0.001829 |
| IACSP95-5000 72 hpi (s.i) | 2 | 21 | RPL1 | -0.40938 | 0.004843 |
| IACSP95-5000 72 hpi (s.i) | 2 | 22 | RPL1 | -0.40446 | 0.010579 |
| IACSP95-5000 72 hpi (s.i) | 2 | 23 | RPL1 | -0.39383 | 0.022033 |
| IACSP95-5000 72 hpi (s.i) | 2 | 24 | RPL1 | -0.37329 | 0.0434 |
| IACSP95-5000 72 hpi (s.i) | 2 | 25 | RPL1 | -0.33352 | 0.083991 |
| IACSP95-5000 72 hpi (s.i) | 2 | 26 | RPL1 | -0.25324 | 0.165091 |
| IACSP95-5000 72 hpi (s.i) | 2 | 27 | RPL1 | -0.10467 | 0.314483 |
| IACSP95-5000 72 hpi (s.i) | 2 | 28 | RPL1 | 0.161749 | 0.581723 |
| IACSP95-5000 72 hpi (s.i) | 2 | 29 | RPL1 | 0.611967 | 1.032763 |
| IACSP95-5000 72 hpi (s.i) | 2 | 30 | RPL1 | 1.322686 | 1.744304 |
| IACSP95-5000 72 hpi (s.i) | 2 | 31 | RPL1 | 2.183686 | 2.606126 |
| IACSP95-5000 72 hpi (s.i) | 2 | 32 | RPL1 | 3.157078 | 3.58034 |
| IACSP95-5000 72 hpi (s.i) | 2 | 33 | RPL1 | 4.086349 | 4.510433 |
| IACSP95-5000 72 hpi (s.i) | 2 | 34 | RPL1 | 4.98042 | 5.405326 |
| IACSP95-5000 72 hpi (s.i) | 2 | 35 | RPL1 | 5.708896 | 6.134624 |
| IACSP95-5000 72 hpi (s.i) | 2 | 36 | RPL1 | 6.363249 | 6.7898 |
| IACSP95-5000 72 hpi (s.i) | 2 | 37 | RPL1 | 6.852478 | 7.27985 |
| IACSP95-5000 72 hpi (s.i) | 2 | 38 | RPL1 | 7.318366 | 7.74656 |
| IACSP95-5000 72 hpi (s.i) | 2 | 39 | RPL1 | 7.661165 | 8.090181 |
| IACSP95-5000 72 hpi (s.i) | 2 | 40 | RPL1 | 7.982232 | 8.41207 |
| IACSP95-5000 72 hpi (s.i) | 3 | 1 | RPL1 | -0.34945 | -0.01512 |
| IACSP95-5000 72 hpi (s.i) | 3 | 2 | RPL1 | -0.34002 | -0.00565 |
| IACSP95-5000 72 hpi (s.i) | 3 | 3 | RPL1 | -0.33434 | 6.56E-05 |
| IACSP95-5000 72 hpi (s.i) | 3 | 4 | RPL1 | -0.33448 | -2.9E-05 |
| IACSP95-5000 72 hpi (s.i) | 3 | 5 | RPL1 | -0.33397 | 0.000518 |
| IACSP95-5000 72 hpi (s.i) | 3 | 6 | RPL1 | -0.33187 | 0.002663 |
| IACSP95-5000 72 hpi (s.i) | 3 | 7 | RPL1 | -0.33507 | -0.00051 |
| IACSP95-5000 72 hpi (s.i) | 3 | 8 | RPL1 | -0.33525 | -0.00064 |
| IACSP95-5000 72 hpi (s.i) | 3 | 9 | RPL1 | -0.33666 | -0.00201 |
| IACSP95-5000 72 hpi (s.i) | 3 | 10 | RPL1 | -0.33728 | -0.00259 |
| IACSP95-5000 72 hpi (s.i) | 3 | 11 | RPL1 | -0.33604 | -0.00131 |
| IACSP95-5000 72 hpi (s.i) | 3 | 12 | RPL1 | -0.33421 | 0.000559 |
| IACSP95-5000 72 hpi (s.i) | 3 | 13 | RPL1 | -0.32999 | 0.004815 |
| IACSP95-5000 72 hpi (s.i) | 3 | 14 | RPL1 | -0.33297 | 0.001876 |
| IACSP95-5000 72 hpi (s.i) | 3 | 15 | RPL1 | -0.33508 | -0.00019 |
| IACSP95-5000 72 hpi (s.i) | 3 | 16 | RPL1 | -0.33696 | -0.00203 |
| IACSP95-5000 72 hpi (s.i) | 3 | 17 | RPL1 | -0.33659 | -0.00162 |
| IACSP95-5000 72 hpi (s.i) | 3 | 18 | RPL1 | -0.33651 | -0.0015 |
| IACSP95-5000 72 hpi (s.i) | 3 | 19 | RPL1 | -0.33633 | -0.00128 |
| IACSP95-5000 72 hpi (s.i) | 3 | 20 | RPL1 | -0.33462 | 0.000466 |
| IACSP95-5000 72 hpi (s.i) | 3 | 21 | RPL1 | -0.33237 | 0.002762 |
| IACSP95-5000 72 hpi (s.i) | 3 | 22 | RPL1 | -0.32771 | 0.00746 |
| IACSP95-5000 72 hpi (s.i) | 3 | 23 | RPL1 | -0.31725 | 0.017959 |
| IACSP95-5000 72 hpi (s.i) | 3 | 24 | RPL1 | -0.29524 | 0.040004 |
| IACSP95-5000 72 hpi (s.i) | 3 | 25 | RPL1 | -0.25414 | 0.081142 |
| IACSP95-5000 72 hpi (s.i) | 3 | 26 | RPL1 | -0.17271 | 0.16262 |
| IACSP95-5000 72 hpi (s.i) | 3 | 27 | RPL1 | -0.02355 | 0.311813 |
| IACSP95-5000 72 hpi (s.i) | 3 | 28 | RPL1 | 0.242659 | 0.578065 |
| IACSP95-5000 72 hpi (s.i) | 3 | 29 | RPL1 | 0.696695 | 1.032141 |
| IACSP95-5000 72 hpi (s.i) | 3 | 30 | RPL1 | 1.405972 | 1.741458 |
| IACSP95-5000 72 hpi (s.i) | 3 | 31 | RPL1 | 2.260028 | 2.595553 |
| IACSP95-5000 72 hpi (s.i) | 3 | 32 | RPL1 | 3.223307 | 3.558872 |
| IACSP95-5000 72 hpi (s.i) | 3 | 33 | RPL1 | 4.147913 | 4.483519 |
| IACSP95-5000 72 hpi (s.i) | 3 | 34 | RPL1 | 5.035881 | 5.371526 |
| IACSP95-5000 72 hpi (s.i) | 3 | 35 | RPL1 | 5.757024 | 6.09271 |
| IACSP95-5000 72 hpi (s.i) | 3 | 36 | RPL1 | 6.39962 | 6.735345 |
| IACSP95-5000 72 hpi (s.i) | 3 | 37 | RPL1 | 6.880962 | 7.216727 |
| IACSP95-5000 72 hpi (s.i) | 3 | 38 | RPL1 | 7.346456 | 7.68226 |
| IACSP95-5000 72 hpi (s.i) | 3 | 39 | RPL1 | 7.703978 | 8.039823 |
| IACSP95-5000 72 hpi (s.i) | 3 | 40 | RPL1 | 8.013577 | 8.349462 |
| IAC91-1099 24 hpi (m.i) | 1 | 1 | UBQ1 | 0.112875 | 0.064004 |
| IAC91-1099 24 hpi (m.i) | 1 | 2 | UBQ1 | 0.084882 | 0.029034 |
| IAC91-1099 24 hpi (m.i) | 1 | 3 | UBQ1 | 0.078456 | 0.015631 |
| IAC91-1099 24 hpi (m.i) | 1 | 4 | UBQ1 | 0.074322 | 0.00452 |
| IAC91-1099 24 hpi (m.i) | 1 | 5 | UBQ1 | 0.075677 | -0.0011 |
| IAC91-1099 24 hpi (m.i) | 1 | 6 | UBQ1 | 0.081885 | -0.00187 |
| IAC91-1099 24 hpi (m.i) | 1 | 7 | UBQ1 | 0.086615 | -0.00412 |
| IAC91-1099 24 hpi (m.i) | 1 | 8 | UBQ1 | 0.092441 | -0.00527 |
| IAC91-1099 24 hpi (m.i) | 1 | 9 | UBQ1 | 0.100965 | -0.00372 |
| IAC91-1099 24 hpi (m.i) | 1 | 10 | UBQ1 | 0.10791 | -0.00375 |
| IAC91-1099 24 hpi (m.i) | 1 | 11 | UBQ1 | 0.116137 | -0.0025 |
| IAC91-1099 24 hpi (m.i) | 1 | 12 | UBQ1 | 0.126482 | 0.000864 |
| IAC91-1099 24 hpi (m.i) | 1 | 13 | UBQ1 | 0.131542 | -0.00105 |
| IAC91-1099 24 hpi (m.i) | 1 | 14 | UBQ1 | 0.137289 | -0.00228 |
| IAC91-1099 24 hpi (m.i) | 1 | 15 | UBQ1 | 0.144229 | -0.00232 |
| IAC91-1099 24 hpi (m.i) | 1 | 16 | UBQ1 | 0.150675 | -0.00285 |
| IAC91-1099 24 hpi (m.i) | 1 | 17 | UBQ1 | 0.159885 | -0.00062 |
| IAC91-1099 24 hpi (m.i) | 1 | 18 | UBQ1 | 0.165446 | -0.00203 |
| IAC91-1099 24 hpi (m.i) | 1 | 19 | UBQ1 | 0.174098 | -0.00036 |
| IAC91-1099 24 hpi (m.i) | 1 | 20 | UBQ1 | 0.182491 | 0.001056 |
| IAC91-1099 24 hpi (m.i) | 1 | 21 | UBQ1 | 0.190756 | 0.002343 |
| IAC91-1099 24 hpi (m.i) | 1 | 22 | UBQ1 | 0.19652 | 0.001131 |
| IAC91-1099 24 hpi (m.i) | 1 | 23 | UBQ1 | 0.206195 | 0.003828 |
| IAC91-1099 24 hpi (m.i) | 1 | 24 | UBQ1 | 0.213836 | 0.004492 |
| IAC91-1099 24 hpi (m.i) | 1 | 25 | UBQ1 | 0.219931 | 0.00361 |
| IAC91-1099 24 hpi (m.i) | 1 | 26 | UBQ1 | 0.234978 | 0.011681 |
| IAC91-1099 24 hpi (m.i) | 1 | 27 | UBQ1 | 0.253247 | 0.022972 |
| IAC91-1099 24 hpi (m.i) | 1 | 28 | UBQ1 | 0.276392 | 0.03914 |
| IAC91-1099 24 hpi (m.i) | 1 | 29 | UBQ1 | 0.318502 | 0.074273 |
| IAC91-1099 24 hpi (m.i) | 1 | 30 | UBQ1 | 0.392586 | 0.141381 |
| IAC91-1099 24 hpi (m.i) | 1 | 31 | UBQ1 | 0.521782 | 0.263599 |
| IAC91-1099 24 hpi (m.i) | 1 | 32 | UBQ1 | 0.765672 | 0.500512 |
| IAC91-1099 24 hpi (m.i) | 1 | 33 | UBQ1 | 1.199094 | 0.926957 |
| IAC91-1099 24 hpi (m.i) | 1 | 34 | UBQ1 | 1.913238 | 1.634124 |
| IAC91-1099 24 hpi (m.i) | 1 | 35 | UBQ1 | 2.921176 | 2.635085 |
| IAC91-1099 24 hpi (m.i) | 1 | 36 | UBQ1 | 4.049106 | 3.756037 |
| IAC91-1099 24 hpi (m.i) | 1 | 37 | UBQ1 | 5.011274 | 4.711229 |
| IAC91-1099 24 hpi (m.i) | 1 | 38 | UBQ1 | 5.947616 | 5.640594 |
| IAC91-1099 24 hpi (m.i) | 1 | 39 | UBQ1 | 6.718792 | 6.404793 |
| IAC91-1099 24 hpi (m.i) | 1 | 40 | UBQ1 | 7.379855 | 7.058878 |
| IAC91-1099 24 hpi (m.i) | 2 | 1 | UBQ1 | 0.155653 | 0.062892 |
| IAC91-1099 24 hpi (m.i) | 2 | 2 | UBQ1 | 0.130843 | 0.029016 |
| IAC91-1099 24 hpi (m.i) | 2 | 3 | UBQ1 | 0.125213 | 0.014321 |
| IAC91-1099 24 hpi (m.i) | 2 | 4 | UBQ1 | 0.123097 | 0.003139 |
| IAC91-1099 24 hpi (m.i) | 2 | 5 | UBQ1 | 0.129422 | 0.000398 |
| IAC91-1099 24 hpi (m.i) | 2 | 6 | UBQ1 | 0.135256 | -0.00283 |
| IAC91-1099 24 hpi (m.i) | 2 | 7 | UBQ1 | 0.144065 | -0.00309 |
| IAC91-1099 24 hpi (m.i) | 2 | 8 | UBQ1 | 0.149984 | -0.00624 |
| IAC91-1099 24 hpi (m.i) | 2 | 9 | UBQ1 | 0.161933 | -0.00335 |
| IAC91-1099 24 hpi (m.i) | 2 | 10 | UBQ1 | 0.171462 | -0.00289 |
| IAC91-1099 24 hpi (m.i) | 2 | 11 | UBQ1 | 0.17994 | -0.00348 |
| IAC91-1099 24 hpi (m.i) | 2 | 12 | UBQ1 | 0.190278 | -0.00221 |
| IAC91-1099 24 hpi (m.i) | 2 | 13 | UBQ1 | 0.197967 | -0.00358 |
| IAC91-1099 24 hpi (m.i) | 2 | 14 | UBQ1 | 0.209845 | -0.00077 |
| IAC91-1099 24 hpi (m.i) | 2 | 15 | UBQ1 | 0.219098 | -0.00059 |
| IAC91-1099 24 hpi (m.i) | 2 | 16 | UBQ1 | 0.229596 | 0.000847 |
| IAC91-1099 24 hpi (m.i) | 2 | 17 | UBQ1 | 0.237817 | 2.05E-06 |
| IAC91-1099 24 hpi (m.i) | 2 | 18 | UBQ1 | 0.247719 | 0.000838 |
| IAC91-1099 24 hpi (m.i) | 2 | 19 | UBQ1 | 0.257841 | 0.001895 |
| IAC91-1099 24 hpi (m.i) | 2 | 20 | UBQ1 | 0.268147 | 0.003134 |
| IAC91-1099 24 hpi (m.i) | 2 | 21 | UBQ1 | 0.278542 | 0.004463 |
| IAC91-1099 24 hpi (m.i) | 2 | 22 | UBQ1 | 0.286681 | 0.003536 |
| IAC91-1099 24 hpi (m.i) | 2 | 23 | UBQ1 | 0.298662 | 0.006452 |
| IAC91-1099 24 hpi (m.i) | 2 | 24 | UBQ1 | 0.311162 | 0.009885 |
| IAC91-1099 24 hpi (m.i) | 2 | 25 | UBQ1 | 0.324437 | 0.014095 |
| IAC91-1099 24 hpi (m.i) | 2 | 26 | UBQ1 | 0.343538 | 0.02413 |
| IAC91-1099 24 hpi (m.i) | 2 | 27 | UBQ1 | 0.37168 | 0.043206 |
| IAC91-1099 24 hpi (m.i) | 2 | 28 | UBQ1 | 0.408869 | 0.071329 |
| IAC91-1099 24 hpi (m.i) | 2 | 29 | UBQ1 | 0.484832 | 0.138226 |
| IAC91-1099 24 hpi (m.i) | 2 | 30 | UBQ1 | 0.620633 | 0.264962 |
| IAC91-1099 24 hpi (m.i) | 2 | 31 | UBQ1 | 0.863258 | 0.498521 |
| IAC91-1099 24 hpi (m.i) | 2 | 32 | UBQ1 | 1.302163 | 0.928359 |
| IAC91-1099 24 hpi (m.i) | 2 | 33 | UBQ1 | 2.041063 | 1.658193 |
| IAC91-1099 24 hpi (m.i) | 2 | 34 | UBQ1 | 3.128772 | 2.736837 |
| IAC91-1099 24 hpi (m.i) | 2 | 35 | UBQ1 | 4.335816 | 3.934815 |
| IAC91-1099 24 hpi (m.i) | 2 | 36 | UBQ1 | 5.496699 | 5.086632 |
| IAC91-1099 24 hpi (m.i) | 2 | 37 | UBQ1 | 6.450465 | 6.031332 |
| IAC91-1099 24 hpi (m.i) | 2 | 38 | UBQ1 | 7.404943 | 6.976744 |
| IAC91-1099 24 hpi (m.i) | 2 | 39 | UBQ1 | 8.121177 | 7.683912 |
| IAC91-1099 24 hpi (m.i) | 2 | 40 | UBQ1 | 8.790172 | 8.343841 |
| IAC91-1099 24 hpi (m.i) | 3 | 1 | UBQ1 | 0.147566 | 0.054595 |
| IAC91-1099 24 hpi (m.i) | 3 | 2 | UBQ1 | 0.127361 | 0.028443 |
| IAC91-1099 24 hpi (m.i) | 3 | 3 | UBQ1 | 0.12066 | 0.015795 |
| IAC91-1099 24 hpi (m.i) | 3 | 4 | UBQ1 | 0.119509 | 0.008697 |
| IAC91-1099 24 hpi (m.i) | 3 | 5 | UBQ1 | 0.121337 | 0.004578 |
| IAC91-1099 24 hpi (m.i) | 3 | 6 | UBQ1 | 0.122363 | -0.00034 |
| IAC91-1099 24 hpi (m.i) | 3 | 7 | UBQ1 | 0.123387 | -0.00527 |
| IAC91-1099 24 hpi (m.i) | 3 | 8 | UBQ1 | 0.130264 | -0.00434 |
| IAC91-1099 24 hpi (m.i) | 3 | 9 | UBQ1 | 0.135738 | -0.00481 |
| IAC91-1099 24 hpi (m.i) | 3 | 10 | UBQ1 | 0.140474 | -0.00602 |
| IAC91-1099 24 hpi (m.i) | 3 | 11 | UBQ1 | 0.148315 | -0.00413 |
| IAC91-1099 24 hpi (m.i) | 3 | 12 | UBQ1 | 0.154793 | -0.00359 |
| IAC91-1099 24 hpi (m.i) | 3 | 13 | UBQ1 | 0.158517 | -0.00582 |
| IAC91-1099 24 hpi (m.i) | 3 | 14 | UBQ1 | 0.165459 | -0.00482 |
| IAC91-1099 24 hpi (m.i) | 3 | 15 | UBQ1 | 0.174219 | -0.00201 |
| IAC91-1099 24 hpi (m.i) | 3 | 16 | UBQ1 | 0.179059 | -0.00312 |
| IAC91-1099 24 hpi (m.i) | 3 | 17 | UBQ1 | 0.18436 | -0.00376 |
| IAC91-1099 24 hpi (m.i) | 3 | 18 | UBQ1 | 0.191191 | -0.00288 |
| IAC91-1099 24 hpi (m.i) | 3 | 19 | UBQ1 | 0.197854 | -0.00216 |
| IAC91-1099 24 hpi (m.i) | 3 | 20 | UBQ1 | 0.209127 | 0.003165 |
| IAC91-1099 24 hpi (m.i) | 3 | 21 | UBQ1 | 0.215022 | 0.003113 |
| IAC91-1099 24 hpi (m.i) | 3 | 22 | UBQ1 | 0.22227 | 0.004413 |
| IAC91-1099 24 hpi (m.i) | 3 | 23 | UBQ1 | 0.228146 | 0.004343 |
| IAC91-1099 24 hpi (m.i) | 3 | 24 | UBQ1 | 0.238699 | 0.008949 |
| IAC91-1099 24 hpi (m.i) | 3 | 25 | UBQ1 | 0.248464 | 0.012767 |
| IAC91-1099 24 hpi (m.i) | 3 | 26 | UBQ1 | 0.263629 | 0.021985 |
| IAC91-1099 24 hpi (m.i) | 3 | 27 | UBQ1 | 0.283813 | 0.036223 |
| IAC91-1099 24 hpi (m.i) | 3 | 28 | UBQ1 | 0.31498 | 0.061442 |
| IAC91-1099 24 hpi (m.i) | 3 | 29 | UBQ1 | 0.369281 | 0.109796 |
| IAC91-1099 24 hpi (m.i) | 3 | 30 | UBQ1 | 0.470999 | 0.205567 |
| IAC91-1099 24 hpi (m.i) | 3 | 31 | UBQ1 | 0.647339 | 0.375961 |
| IAC91-1099 24 hpi (m.i) | 3 | 32 | UBQ1 | 0.975679 | 0.698354 |
| IAC91-1099 24 hpi (m.i) | 3 | 33 | UBQ1 | 1.541775 | 1.258503 |
| IAC91-1099 24 hpi (m.i) | 3 | 34 | UBQ1 | 2.411237 | 2.122018 |
| IAC91-1099 24 hpi (m.i) | 3 | 35 | UBQ1 | 3.463104 | 3.167938 |
| IAC91-1099 24 hpi (m.i) | 3 | 36 | UBQ1 | 4.501872 | 4.200758 |
| IAC91-1099 24 hpi (m.i) | 3 | 37 | UBQ1 | 5.358941 | 5.051881 |
| IAC91-1099 24 hpi (m.i) | 3 | 38 | UBQ1 | 6.181929 | 5.868922 |
| IAC91-1099 24 hpi (m.i) | 3 | 39 | UBQ1 | 6.835042 | 6.516088 |
| IAC91-1099 24 hpi (m.i) | 3 | 40 | UBQ1 | 7.382633 | 7.057732 |
| IAC91-1099 24 hpi (s.i) | 1 | 1 | UBQ1 | -0.3081 | 0.002947 |
| IAC91-1099 24 hpi (s.i) | 1 | 2 | UBQ1 | -0.31018 | -0.00112 |
| IAC91-1099 24 hpi (s.i) | 1 | 3 | UBQ1 | -0.30688 | 0.000186 |
| IAC91-1099 24 hpi (s.i) | 1 | 4 | UBQ1 | -0.30482 | 0.000255 |
| IAC91-1099 24 hpi (s.i) | 1 | 5 | UBQ1 | -0.30134 | 0.00175 |
| IAC91-1099 24 hpi (s.i) | 1 | 6 | UBQ1 | -0.29929 | 0.001804 |
| IAC91-1099 24 hpi (s.i) | 1 | 7 | UBQ1 | -0.29859 | 0.000509 |
| IAC91-1099 24 hpi (s.i) | 1 | 8 | UBQ1 | -0.29647 | 0.000645 |
| IAC91-1099 24 hpi (s.i) | 1 | 9 | UBQ1 | -0.29392 | 0.001196 |
| IAC91-1099 24 hpi (s.i) | 1 | 10 | UBQ1 | -0.29315 | -1.6E-05 |
| IAC91-1099 24 hpi (s.i) | 1 | 11 | UBQ1 | -0.28956 | 0.001578 |
| IAC91-1099 24 hpi (s.i) | 1 | 12 | UBQ1 | -0.28976 | -0.00061 |
| IAC91-1099 24 hpi (s.i) | 1 | 13 | UBQ1 | -0.28791 | -0.00075 |
| IAC91-1099 24 hpi (s.i) | 1 | 14 | UBQ1 | -0.28843 | -0.00327 |
| IAC91-1099 24 hpi (s.i) | 1 | 15 | UBQ1 | -0.28434 | -0.00117 |
| IAC91-1099 24 hpi (s.i) | 1 | 16 | UBQ1 | -0.28211 | -0.00093 |
| IAC91-1099 24 hpi (s.i) | 1 | 17 | UBQ1 | -0.28086 | -0.00167 |
| IAC91-1099 24 hpi (s.i) | 1 | 18 | UBQ1 | -0.27795 | -0.00075 |
| IAC91-1099 24 hpi (s.i) | 1 | 19 | UBQ1 | -0.27855 | -0.00334 |
| IAC91-1099 24 hpi (s.i) | 1 | 20 | UBQ1 | -0.2742 | -0.00098 |
| IAC91-1099 24 hpi (s.i) | 1 | 21 | UBQ1 | -0.27176 | -0.00053 |
| IAC91-1099 24 hpi (s.i) | 1 | 22 | UBQ1 | -0.27098 | -0.00174 |
| IAC91-1099 24 hpi (s.i) | 1 | 23 | UBQ1 | -0.26875 | -0.0015 |
| IAC91-1099 24 hpi (s.i) | 1 | 24 | UBQ1 | -0.26434 | 0.000913 |
| IAC91-1099 24 hpi (s.i) | 1 | 25 | UBQ1 | -0.26268 | 0.000578 |
| IAC91-1099 24 hpi (s.i) | 1 | 26 | UBQ1 | -0.2595 | 0.001773 |
| IAC91-1099 24 hpi (s.i) | 1 | 27 | UBQ1 | -0.25321 | 0.006074 |
| IAC91-1099 24 hpi (s.i) | 1 | 28 | UBQ1 | -0.24718 | 0.010104 |
| IAC91-1099 24 hpi (s.i) | 1 | 29 | UBQ1 | -0.23713 | 0.018167 |
| IAC91-1099 24 hpi (s.i) | 1 | 30 | UBQ1 | -0.21601 | 0.037302 |
| IAC91-1099 24 hpi (s.i) | 1 | 31 | UBQ1 | -0.1821 | 0.069221 |
| IAC91-1099 24 hpi (s.i) | 1 | 32 | UBQ1 | -0.11607 | 0.133257 |
| IAC91-1099 24 hpi (s.i) | 1 | 33 | UBQ1 | 0.012074 | 0.259408 |
| IAC91-1099 24 hpi (s.i) | 1 | 34 | UBQ1 | 0.251928 | 0.49727 |
| IAC91-1099 24 hpi (s.i) | 1 | 35 | UBQ1 | 0.688908 | 0.932259 |
| IAC91-1099 24 hpi (s.i) | 1 | 36 | UBQ1 | 1.431892 | 1.673252 |
| IAC91-1099 24 hpi (s.i) | 1 | 37 | UBQ1 | 2.538838 | 2.778207 |
| IAC91-1099 24 hpi (s.i) | 1 | 38 | UBQ1 | 3.878427 | 4.115805 |
| IAC91-1099 24 hpi (s.i) | 1 | 39 | UBQ1 | 5.115399 | 5.350786 |
| IAC91-1099 24 hpi (s.i) | 1 | 40 | UBQ1 | 6.238166 | 6.471562 |
| IAC91-1099 24 hpi (s.i) | 2 | 1 | UBQ1 | -0.31634 | 0.002829 |
| IAC91-1099 24 hpi (s.i) | 2 | 2 | UBQ1 | -0.31613 | 0.001242 |
| IAC91-1099 24 hpi (s.i) | 2 | 3 | UBQ1 | -0.31243 | 0.003146 |
| IAC91-1099 24 hpi (s.i) | 2 | 4 | UBQ1 | -0.31085 | 0.002927 |
| IAC91-1099 24 hpi (s.i) | 2 | 5 | UBQ1 | -0.31092 | 0.001057 |
| IAC91-1099 24 hpi (s.i) | 2 | 6 | UBQ1 | -0.30891 | 0.001266 |
| IAC91-1099 24 hpi (s.i) | 2 | 7 | UBQ1 | -0.30725 | 0.00113 |
| IAC91-1099 24 hpi (s.i) | 2 | 8 | UBQ1 | -0.30496 | 0.001626 |
| IAC91-1099 24 hpi (s.i) | 2 | 9 | UBQ1 | -0.3045 | 0.000286 |
| IAC91-1099 24 hpi (s.i) | 2 | 10 | UBQ1 | -0.30271 | 0.000271 |
| IAC91-1099 24 hpi (s.i) | 2 | 11 | UBQ1 | -0.30239 | -0.0012 |
| IAC91-1099 24 hpi (s.i) | 2 | 12 | UBQ1 | -0.30239 | -0.00301 |
| IAC91-1099 24 hpi (s.i) | 2 | 13 | UBQ1 | -0.3028 | -0.00521 |
| IAC91-1099 24 hpi (s.i) | 2 | 14 | UBQ1 | -0.29875 | -0.00296 |
| IAC91-1099 24 hpi (s.i) | 2 | 15 | UBQ1 | -0.29711 | -0.00312 |
| IAC91-1099 24 hpi (s.i) | 2 | 16 | UBQ1 | -0.29498 | -0.00279 |
| IAC91-1099 24 hpi (s.i) | 2 | 17 | UBQ1 | -0.29122 | -0.00083 |
| IAC91-1099 24 hpi (s.i) | 2 | 18 | UBQ1 | -0.29135 | -0.00276 |
| IAC91-1099 24 hpi (s.i) | 2 | 19 | UBQ1 | -0.28797 | -0.00117 |
| IAC91-1099 24 hpi (s.i) | 2 | 20 | UBQ1 | -0.28526 | -0.00026 |
| IAC91-1099 24 hpi (s.i) | 2 | 21 | UBQ1 | -0.28374 | -0.00054 |
| IAC91-1099 24 hpi (s.i) | 2 | 22 | UBQ1 | -0.27905 | 0.002352 |
| IAC91-1099 24 hpi (s.i) | 2 | 23 | UBQ1 | -0.27927 | 0.000335 |
| IAC91-1099 24 hpi (s.i) | 2 | 24 | UBQ1 | -0.27439 | 0.003412 |
| IAC91-1099 24 hpi (s.i) | 2 | 25 | UBQ1 | -0.26998 | 0.006025 |
| IAC91-1099 24 hpi (s.i) | 2 | 26 | UBQ1 | -0.2615 | 0.012705 |
| IAC91-1099 24 hpi (s.i) | 2 | 27 | UBQ1 | -0.25111 | 0.021299 |
| IAC91-1099 24 hpi (s.i) | 2 | 28 | UBQ1 | -0.23261 | 0.038003 |
| IAC91-1099 24 hpi (s.i) | 2 | 29 | UBQ1 | -0.19575 | 0.073063 |
| IAC91-1099 24 hpi (s.i) | 2 | 30 | UBQ1 | -0.12383 | 0.143177 |
| IAC91-1099 24 hpi (s.i) | 2 | 31 | UBQ1 | 0.002408 | 0.267621 |
| IAC91-1099 24 hpi (s.i) | 2 | 32 | UBQ1 | 0.246719 | 0.510134 |
| IAC91-1099 24 hpi (s.i) | 2 | 33 | UBQ1 | 0.692369 | 0.953985 |
| IAC91-1099 24 hpi (s.i) | 2 | 34 | UBQ1 | 1.447501 | 1.707319 |
| IAC91-1099 24 hpi (s.i) | 2 | 35 | UBQ1 | 2.588795 | 2.846813 |
| IAC91-1099 24 hpi (s.i) | 2 | 36 | UBQ1 | 3.968333 | 4.224553 |
| IAC91-1099 24 hpi (s.i) | 2 | 37 | UBQ1 | 5.178526 | 5.432947 |
| IAC91-1099 24 hpi (s.i) | 2 | 38 | UBQ1 | 6.305386 | 6.558009 |
| IAC91-1099 24 hpi (s.i) | 2 | 39 | UBQ1 | 7.207376 | 7.4582 |
| IAC91-1099 24 hpi (s.i) | 2 | 40 | UBQ1 | 7.97799 | 8.227015 |
| IAC91-1099 24 hpi (s.i) | 3 | 1 | UBQ1 | -0.28305 | -0.00217 |
[truncated: 50,730 more chars]
