# Supplementary material for: Reference genes for gene expression studies targeting sugarcane infected with Sugarcane mosaic virus (SCMV)
Source: BMC Res Notes. 2019 Mar 18;12:149. doi: 10.1186/s13104-019-4168-5 (PMC6423880; doi:10.1186/s13104-019-4168-5)
Supplement: Supplementary file 5 — Additional file 5: Figure S3. qRT-PCR amplicon size verification in agarose gel 1% of three newly designed primer pairs in cDNA bulks and genomic DNA from IACSP95-5000 and IAC91-1099 sugarcane cultivars. [file 13104_2019_4168_MOESM5_ESM.docx]

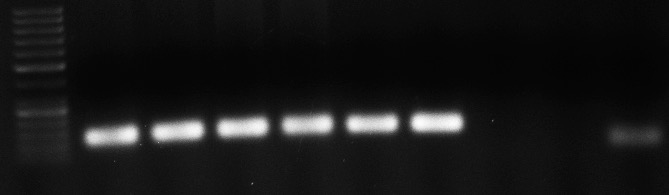


**7**

**9**

**5**

**L**

**3**

**8**

**6**

**4**

**2**

**1**

**a)**

**b)**

200bp

100bp


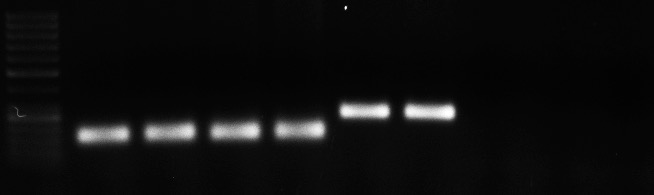


**7**

**4**

**3**

**2**

**1**

**L**

**8**

**9**

**6**

**5**

**c)**

250 bp

150 bp


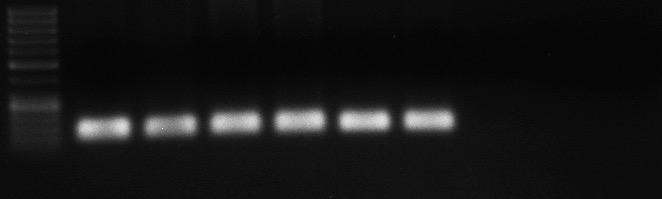


**7**

**5**

**4**

**3**

**2**

**1**

**L**

**8**

**9**

**6**

100 bp

200 bp

Figure S3. Agarose gel 1% of qRT-PCR products of three newly designed primer pairs: a) SAND; b) UK; c) UBC18. L: 50 bp DNA Ladder (GeneRulerTM Fermentas); 1 and 2: two technical replicates of the amplicon from IACSP95-5000 cDNA bulk; 3 and 4: two technical replicates of the amplicon ,from IAC91-1099 cDNA bulk; 5: amplicon from IACSP95-5000 genomic DNA; 6: amplicon from IAC91-1099 genomic DNA; 7: empty lane; 8 and 9: two technical replicates for blank. The band in the no template control (NTC) for gene SAND, a) lane 9, is the result of primer dimer formation. The primer pair selected for gene UK flanks two adjacent exons flanking an intron, resulting in a bigger amplicon when tested in genomic DNA.
